# Screening and identifying of biomarkers in early colorectal cancer and adenoma based on genome-wide methylation profiles

**DOI:** 10.1186/s12957-023-03189-1

**Published:** 2023-10-02

**Authors:** Chungang He, Qinyuan Huang, Shibiao Zhong, Li Sheng Chen, Hewei Xiao, Lei Li

**Affiliations:** 1 Department of Colorectal and Anal Surgery, the People’s Hospital of Guangxi Zhuang Autonomous Region, Tao Yuan Road No.6, Nanning, 530021 Guangxi China; 2 Nursing College of Guangxi Medical University, Nanning, 530021 Guangxi China; 3 Department of Gastrointestinal Surgery, Ruikang Hospital Affiliated to Guangxi University of Traditional Chinese Medicine, Nanning, 530011 Guangxi China; 4 Department of Colorectal and Anal Surgery, the First Affiliated Hospital of Guangxi Medical University, Nanning, 530021 Guangxi China; 5 Office of Academic Research, the People’s Hospital of Guangxi Zhuang Autonomous Region, Nanning, 530021 Guangxi China; 6 Department of Gastrointestinal Surgery, the People’s Hospital of Guangxi Zhuang Autonomous Region, Nanning, 530021 Guangxi China

**Keywords:** Colorectal cancer, Adenoma, Biomarkers, Illumina methylation chip

## Abstract

**Background:**

Colorectal cancer is one of the most common malignant tumors worldwide with high morbidity and mortality. This study aimed to identify different methylation sites as new methylation markers in CRC and colorectal adenoma through tissue detection.

**Methods:**

DNA extraction and bisulfite modification as well as Infinium 450K methylation microarray detection were performed in 46 samples of sporadic colorectal cancer tissue, nine samples of colorectal adenoma, and 20 normal samples, and bioinformatic analysis was conducted involving genes enrichments of GO and KEGG. Pyrosequencing methylation detection was further performed in 68 sporadic colorectal cancer tissues, 31 samples of colorectal adenoma, and 49 normal colorectal mucosae adjacent to carcinoma to investigate the differentially methylated genes obtained from methylation microarray.

**Results:**

There were 65,535 differential methylation marker probes, among which 25,464 were hypermethylated markers and 40,071 were hypomethylated markers in the adenoma compared with the normal group, and 395,571 were differentially methylated markers in patients with sporadic colorectal cancer compared with the normal group, including 21,710 hypermethylated markers and 17,861 hypomethylated markers. Five hypermethylated genes including ZNF471, SND1, SPOCK1, FBLIM1, and OTX1 were detected and confirmed in 68 cases of colorectal cancer, 31 cases of adenoma, and 49 cases of normal control group.

**Conclusions:**

Hypermethylated genes of ZNF471, SND1, SPOCK1, FBLIM1, and OTX1 were obtained from methylation chip detection and further confirm analysis in colorectal cancer and adenoma compared with normal tissue, which may be promising diagnostic markers of colorectal cancer and colorectal adenoma.

**Supplementary Information:**

The online version contains supplementary material available at 10.1186/s12957-023-03189-1.

## Introduction

Colorectal cancer (CRC) is one of the most common malignant tumors worldwide with high morbidity and mortality [1]. Estimated new cancer cases of CRC rank second and deaths rank the fifth of all cancers in 2022 in China [2]. Early diagnosis-involved biomarkers may play a crucial role in improving the prognosis of patients with CRC and remain to be explored currently.

“Normal-adenoma-cancer-cancer metastasis” is the main pathway for the occurrence and development of sporadic CRC, during which a series of changes of cumulative genes and epigenetics might occur [3, 4]. It has been shown in many that abnormal DNA methylation, including hypermethylated and hypomethylated states, is widespread in colorectal adenomas, and many abnormal methylated genes have been found, suggesting that the change of gene epigenetic inheritance, as a frequent early event, may affect the transition of colorectal adenomas to CRC, which may also serve as a biomarker for early diagnosis [3, 5, 6]. However, traditional research methods are mainly limited to the discovery and detection of a single gene, unable to reveal the methylation status of the whole colorectal adenoma and CRC. There are deficiencies in the pathogenesis, early diagnosis, and risk assessment of colorectal adenoma and cancer [7]. Therefore, the adoption of a new genomic methylation detection method is more conducive to a comprehensive analysis of the methylation profile of colorectal adenoma, further revealing its mechanism of action in colorectal adenoma and CRC development and discovering new methylation markers that may be used in the early diagnosis of adenoma and CRC.

Illumina Infinium 450K detection technology with powerful detection function, high throughput, and high sensitivity is a genome-wide methylation detection and analysis method. We aimed to find different methylation sites as new methylation markers in CRC and colorectal adenoma compared with the normal group using Illumina Infinium 450K methylation chip to detect and analyze the methylation spectrum of each group. Our results would provide new ideas and clues for investigating of pathogenesis and early diagnosis of colorectal adenoma and CRC.

## Methods

### Sample collection

All the patients who donated samples for the present study have provided consent following an ethical approval from the ethics committee of the People’s Hospital of Guangxi Zhuang Autonomous Region (No.: 2013–017).

For methylation microarrays (patient characteristics were shown in Table S1), 46 sporadic colorectal cancer tissues were obtained from patients with colorectal surgery in the First Affiliated Hospital of Guangxi Medical University and patients with sporadic colorectal cancer radical resection in the Guangxi Zhuang Autonomous Region People’s Hospital from January 2013 to December 2014. Tumor tissue without apparent necrosis around the tumor site was taken. The specimens of 9 patients with colorectal adenoma (diameter > 2 cm) were collected from the patients who underwent surgery for the first time between January 2013 and December 2014. All of them were confirmed by electronic colonoscopy and pathology before surgery. Colorectal specimens from 6 hemorrhoids, 4 patients with constipation, and 10 adjacent normal mucosa (> 10 cm away from the tumor) were selected as normal controls. Individuals with matched gender and similar age to the patients in the colorectal cancer and adenoma groups were chosen. None of the subjects had familial inherited colorectal disease and a history of smoking.

For confirmation of CpG methylation markers (patient characteristics were shown in Table S2), 68 sporadic colorectal cancer tissues, 31 samples of colorectal adenoma, and 49 normal colorectal mucosa adjacent to carcinoma were collected. All samples were confirmed by pathology. The specimens were frozen in liquid nitrogen within 10 min after surgical resection and then transferred to – 80 °C for storage.

### DNA extraction, bisulfite modification and Infinium 450K methylation microarray and analysis

DNA in tissues was extracted according to the QIAamp DNA Mini Kit (Qiagen, Hidden, Germany) kit instruction (Supplement 1). Quality detection, quantification of extracted DNA, and modification and transformation of DNA bisulfite were performed according to the instructions of the Zymo EZ DNA Methylation Kit (Zymo Research, CA, USA) (Supplement 2).

All bisulfite-modified DNA samples were performed 450K methylation microarray (Illumina, San Diego, Ca, USA) according to the instructions (Supplement 3). The chip was scanned with an Illumina HiScan SQ scanner (Illumina, San Diego, Ca, USA).

The signal value was extracted from the original data (idat file) using genomestudio (genomestudio software 2011.1, Illumina). And results were obtained after importing the original data to genomestudio (genomestudio software 2011.1, Illumina) for normalization. The normalization method selected in the present study is “Background correction.” Background values are the average strength values of negative controls of points on the microbead chip. Since the characteristics of negative control points are the same as those of general probes in terms of thermodynamic properties, but there is no specific binding target in the genome investigation, the negative control signal value can be used for background correction. Negative controls can be used to estimate signal strength levels at points without hybridizing target genes. The strength of the probe is subtracted from the average strength of the negative comparison point. As a result, the strength value of the non-expressed genes should theoretically be 0, so that half of the non-expressed genes may have a negative signal strength value. Beta values were used to calculate the degree of methylation at CpG sites. The Illumina methylation chip designs two probes for each CpG site, using two-color fluorescence signals signalB and signalA to detect methylated and non-methylated alleles. The intensity ratio of signalB and signalA was the methylation level of the CpG site. *β* = Max (SignalB,0)/Max (SignalA,0) + Max (SignalB,0) + 100.

Data obtained from methylation microarray had undergone bioinformatics analysis of gene enrichment by Gene Ontology (GO, http://geneontology.org/) and Kyoto Encyclopedia of Genes and Genomes (KEGG, https://www.kegg.jp/) online.

The hypermethylation and hypomethylation genes with − 3 to 3 change between colorectal cancer and normal as well as adenoma and normal were involved in the cluster analysis. The distance between the pairs of multiple samples is calculated to form the distance matrix according to the expression of the selected differential methylation sites.

### Confirmation methylation markers

The primers of methylation markers selected are shown in Table S8. Bisulfite transformation was performed according to the instruction of the EpiTect Bisulfite Kit (Supplement 4), and then pyrosequencing was performed.

### Statistical analysis

The beta value was used to calculate the methylation degree of CpG sites. The Illumina methylation chip is designed to detect methylated and unmethylated alleles using two-color fluorescent signals signalB and signalA for each CpG site. The intensity ratio of signalB to signalA was the methylation level of the CpG site. *t*-test was used to compare the two groups and ANOVA was used to analyze more than two groups.

## Results

### Distribution of microarray data

**Fig. 1:**
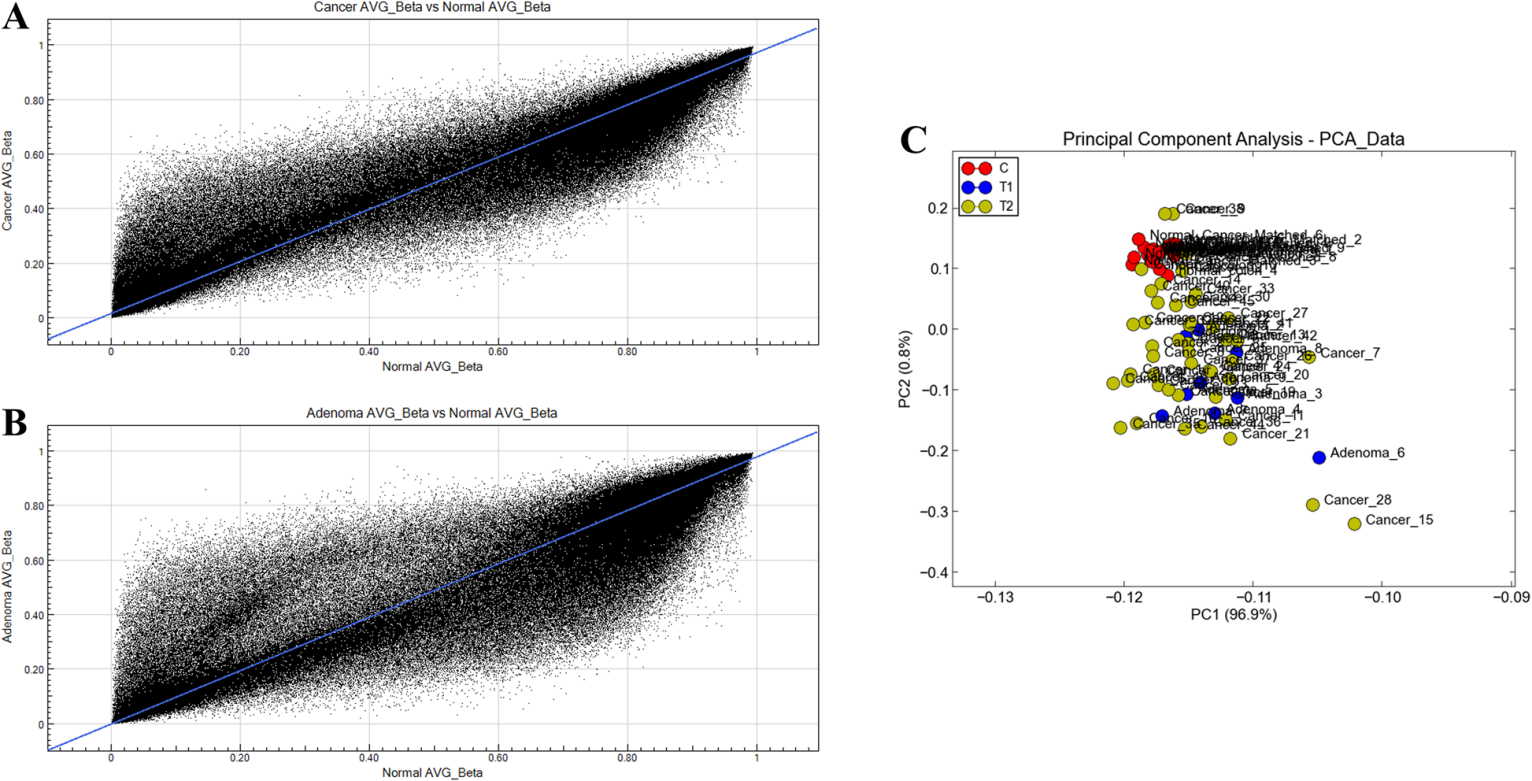
Distribution of microarray data. **A** Scatter plot distribution of colorectal cancer. **B** Scatter plot distribution of the adenoma group. **C** PCA analysis was performed on all probes on the chip to investigate the sample distribution

Scatter plots of chip data are used to evaluate the central tendency of two sets of data. The average beta values of the two groups were used to plot to scatter plots in a two-dimensional cartesian coordinate plane. Figure 1A shows the scatter plot distribution of CRC, and Fig. 1B shows the scatter plot distribution of the adenoma group. PCA analysis was performed on all probes on the chip to investigate the sample distribution. Our results from the principal component analysis indicated that samples of each group are distributed in different areas of two-dimensional or three-dimensional space, and the spatial distribution of samples in the same group is relatively concentrated (Fig. 1C).

### Methylation profile analysis of colorectal cancer and adenoma

**Fig. 2:**
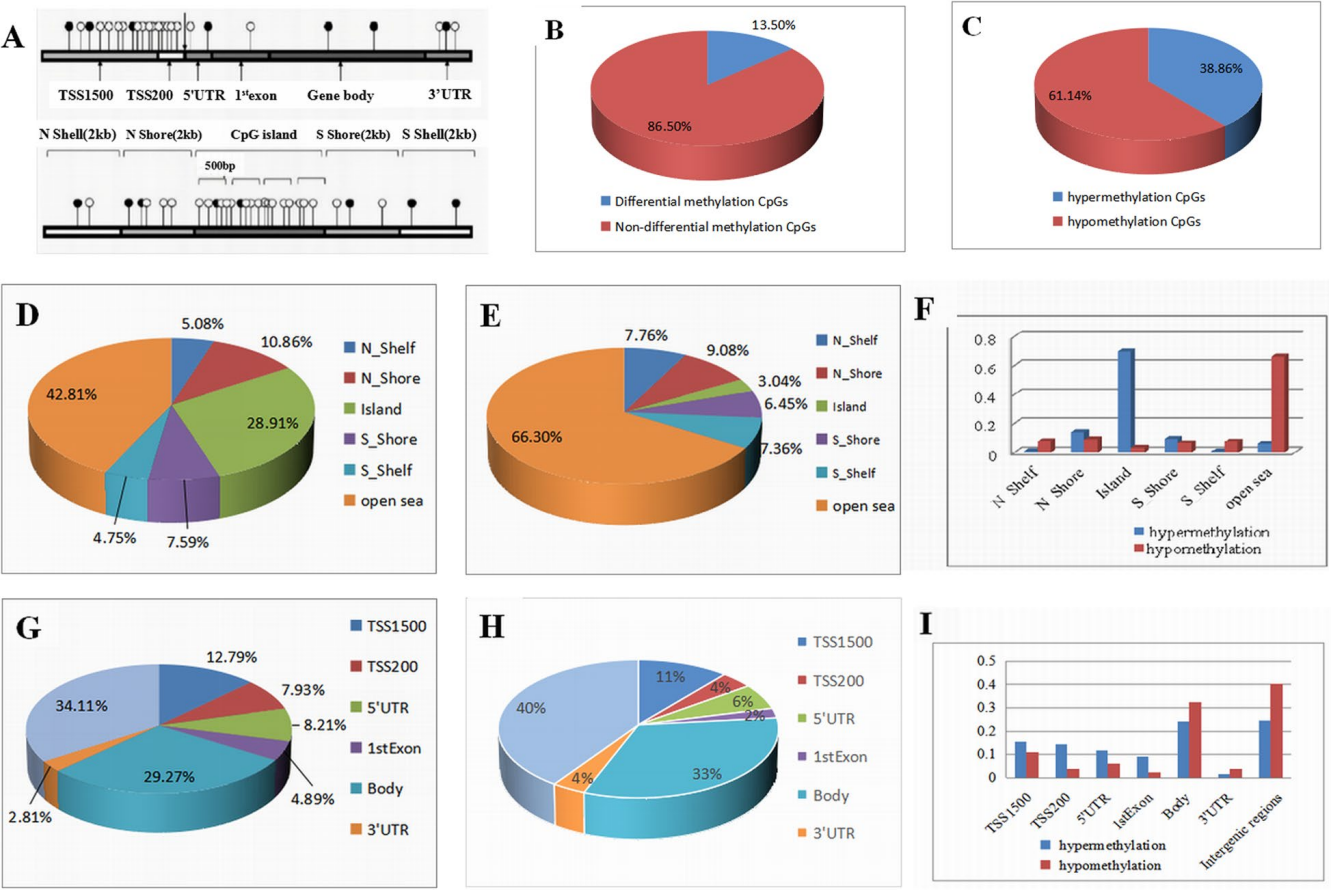
Methylation profile analysis in the adenoma and normal groups. **A** Gene and CpG island structure map. **B** The percentage of differentially methylated sites in all CpGs. **C** The proportion of hypermethylation and hypomethylation CpGs on differentially methylated CpGs. **D** The distribution of differentially methylated CpGs was analyzed on CpG island-related regions. **E** The distribution of differentially hypomethylation CpGs was analyzed on CpG island-related regions. **F** Differentially methylated-CpGs were further divided into hyper/hypo-methylation CpGs to compare the distribution on CpG island-related regions. **G** The distribution of differentially methylated-CpGs was analyzed on the CpG island of the promoter. **H** The distribution of differentially hypomethylation CpGs was analyzed on the CpG island of the promoter. **I** Differentially methylated CpGs were further divided into hyper/hypo-methylation CpGs to compare the distribution on the CpG island of the promoter

HumanMetylation450K methylation chip detection and difference analysis were performed in our study. The chip contains a total of 485,577 methylation markers (probes), among which 485,512 probes are involved in this study. Differential methylation sites common to colorectal cancer, adenoma, colorectal cancer, and adenoma were first screened out, and the methylation profiles of each group were mainly described according to the distribution of differentially high/low methylated CpG sites in gene structure and CpG island-related structures (Fig. 2A).

In the comparison between the adenoma and normal group, there were a total of 65,535 (13.50%) differential methylation marker probes, among which 25,464 (38.86%) were hypermethylated markers and 40,071 (61.14%) were hypomethylated markers. Eight thousand five hundred forty-one differentially methylated genes were among all the differential methylation sites (Table 1, Fig. 2B, C). The distribution of differentially methylated CpGs was analyzed on CpG island-related regions and gene categories which were shown in Fig. 2D, G. 69.63% of hypermethylated markers were located in CpG islands (Fig. 2F). In comparison, 66.30% of hypomethylated markers were located outside CpG islands (open sea regions) (Fig. 2E). 45.33% of highly methylated CpG sites were found in promoter regions (TSS 1500, TSS 200, 5′-UTR and 1st exon) (Fig. 2I), while 40% of hypomethylated markers were found in intergenic regions. Thirty-seven percent and 33% of the hypomethylation sites were located in the intra-gene region (body and 3′UTR) and promoter region (including TSS1500, TSS200, ′UTR, and 1st exon), respectively (Fig. 2H).

**Table 1:** The ratio of differentially methylated CpG sites in all CpGs tested

Group	All CpGs	Differentially methylation CpGs (%)	Non-differentially methylation CpGs (%)
Adenoma	485,512 (100%)	65,535 (13.50%)	419,977 (86.50%)
Colorectal cancer	485,512 (100%)	39,571 (8.15%)	445,941 (91.85%)

**Fig. 3:**
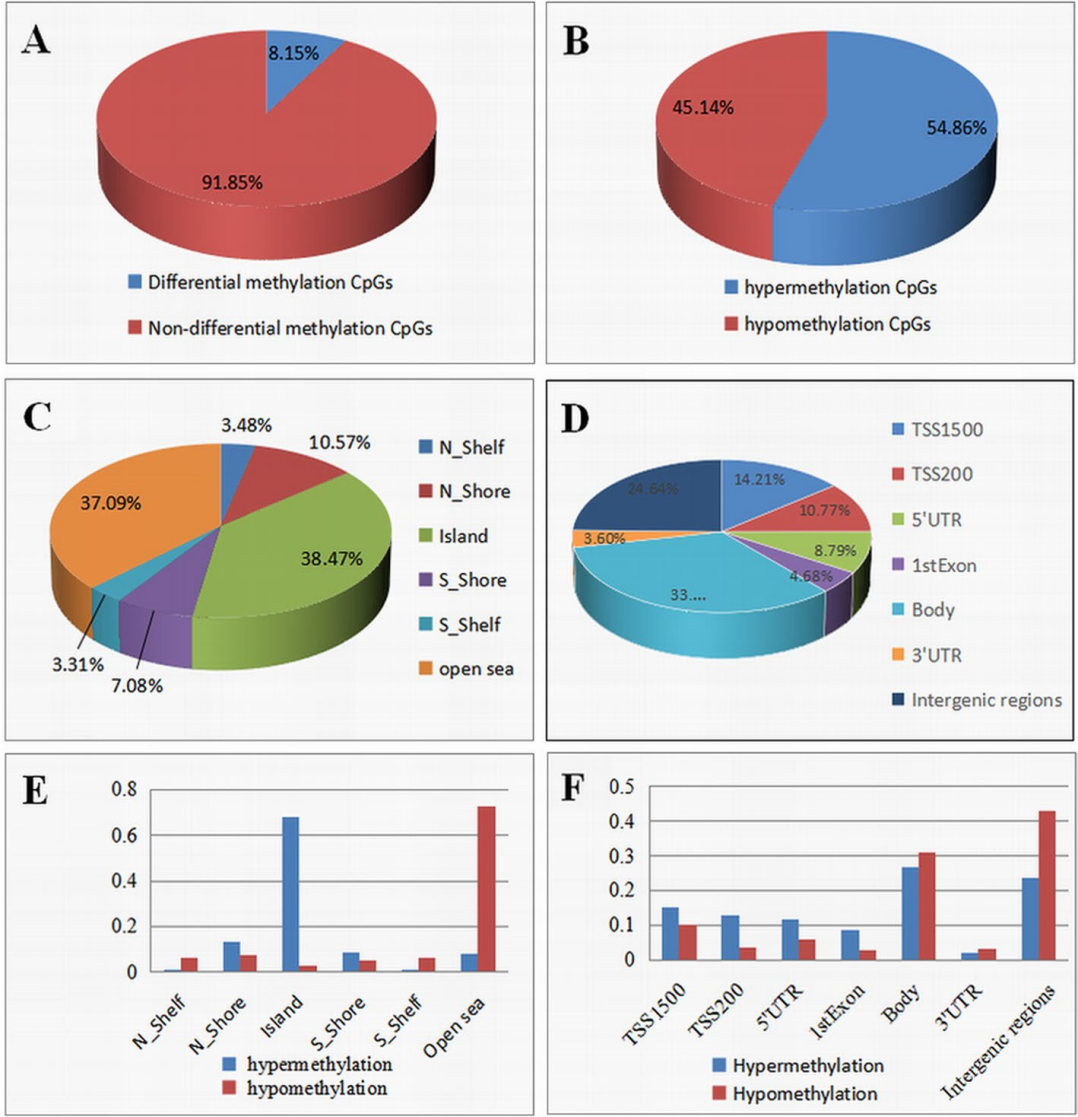
Methylation profile analysis in the colorectal cancer and normal groups. **A** The percentage of differentially methylated sites in all CpGs. **B** The proportion of hypermethylation and hypomethylation CpGs on differentially methylated CpGs. **C** The distribution of differentially methylated CpGs was analyzed on CpG island-related regions. **D** The distribution of differentially methylated-CpGs was analyzed on the CpG island of the promoter. **E** Differentially methylated CpGs were further divided into hyper/hypo-methylation CpGs to compare the distribution on CpG island-related regions. **F** Differentially methylated CpGs were further divided into hyper/hypo-methylation CpGs to compare the distribution on the CpG island of the promoter

There were 395,571 (8.15%) differentially methylated markers in patients with sporadic colorectal cancer compared with the normal group, including 21,710 (54.86%) hypermethylated markers and 17,861 (45.14%) hypomethylated markers. Among all the differentially methylated sites, 3551 differentially methylated genes were included. The results of the subgroup statistical analysis of differential methylation sites in gene structure and CpG island-related regions according to hypermethylation/hypomethylation were shown in Fig. 3. There were 8.15% of differentially methylated genes CpGs in all CpGs, among which there were 45.14% hypomethylation CpGs and 54.86% hypermethylation CpGs on differentially methylated CpGs (Fig. 3A, B). It was shown in Fig. 3C, E, that 38.47% of differentially methylated CpGs were in CpG island and 37.09% in the open sea. Gene categories’ analyzed results showed that 33.3% of hypomethylation CpG sites were in the body (Fig. 3D, F).

**Fig. 4:**
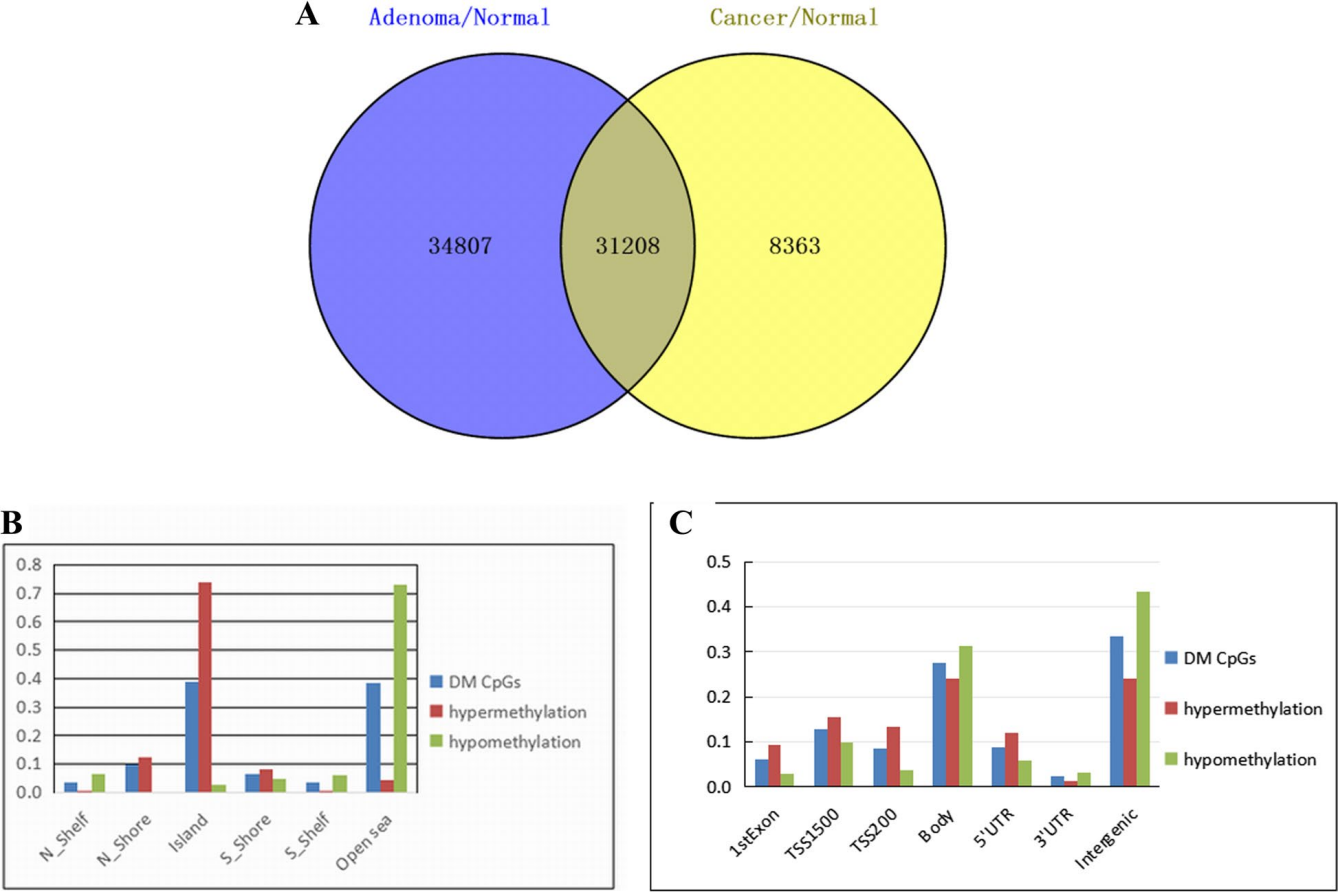
The proportion of distribution for differential methylation CpG markers and descriptive analysis of co-differentially methylated sites in colorectal cancers and adenomas. **A** The proportion of distribution for differential methylation CpG markers in adenoma and colorectal cancer respectively. The middle section represents the co-distribution for differential methylation CpG markers. The blue area represents a unique marker of adenoma, and the yellow portion is a unique marker of colorectal. **B** The distribution of co-differential methylation CpGs and co-hyper/hypo-methylation CpGs was analyzed on CpG island-related regions. **C** The distribution of co-differential methylation CpGs and co-hyper/hypo-methylation CpGs was analyzed on the CpG island of the promoter

Results of the further analysis revealed that there were 31,208 CpG methylation markers in colorectal cancer and adenoma (Fig. 4A), and the distribution of CpG sites in the gene structure and CpG island-related regions was shown in Fig. 4B, C. In this study, the top 20 hypermethylated and hypomethylated markers in adenoma and colorectal cancer were listed for the screened differential methylation markers (Tables 2 and 3).

**Table 2:** List of top 20 candidate hypermethylated/hypomethylated genes in adenomas

Differentially hypermethylated CpG sites						Differentially hypomethylated CpG sites					
CpG ID	Δβ	Chromosome	Gene name	Gene-based region	CpG island-based region	CpG ID	Δβ	Chromosome	Gene name	Gene-based region	CpG island-based region
cg09296001	0.702	7	SND1	Body	Island	cg01372366	− 0.518	11	PTPRJ	Body	Open sea
cg19358877	0.701	19	ZNF471	1stExon	Island	cg24032190	− 0.513	15	SMAD3	Body	Open sea
cg13895235	0.622	7	PRKAR1B	5′UTR	S_Shore	cg26668151	− 0.488	7	DNAJB6	Body	Open sea
cg22474464	0.617	20	NKX2-2	Body	Island	cg11228682	− 0.487	6	ATXN1	Body	Open sea
cg06952671	0.598	2	ITGA4	5′UTR	Island	cg14284630	− 0.483	11	OR5T3	TSS1500	Open sea
cg04292718	0.588	1	CD34	5′UTR	Island	cg06197966	− 0.481	10	SVIL	5′UTR	Open sea
cg25884711	0.652	7	NPY	5′UTR	Island	cg00438740	− 0.470	2	INPP5D	TSS200	N_Shore
cg22434409	0.579	4	KCNIP4	TSS1500	Island	cg02939767	− 0.459	20	TCF15	3′UTR	Open sea
cg12664464	0.578	20	GATA5	1stExon	Island	cg04923875	− 0.459	1	TNR	5′UTR	Open sea
cg16791424	0.575	14	NOVA1	TSS1500	S_Shore	cg02107844	− 0.453	15	SLCO3A1	Body	Open sea
cg16741041	0.575	13	SPG20	5′UTR	Island	cg03301058	− 0.452	6	GABRR2	Body	Open sea
cg05842348	0.565	14	PAX9	Body	S_Shore	cg13942186	− 0.452	9	EXD3	5′UTR	Open sea
cg03976877	0.563	7	VIPR2	1stExon	Island	cg03796321	− 0.452	15	AKAP13	Body	Open sea
cg25784220	0.562	19	ZSCAN18	1stExon	Island	cg06178383	− 0.451	16	SCNN1G	Body	Open sea
cg22065614	0.557	4	GLRB	1stExon	Island	cg27423177	− 0.448	12	C3AR1	TSS1500	Open sea
cg24152605	0.559	19	ZFP28	1stExon	Island	cg08942191	− 0.446	15	ARNT2	Body	Open sea
cg09248054	0.557	1	AGRN	Body	Island	cg23622047	− 0.438	2	RAPGEF4	Body	Open sea
cg24371075	0.553	17	MYH10	5′UTR	Island	cg00370106	− 0.437	6	SYTL3	1stExon	Open sea
cg14270292	0.550	6	EYA4	5′UTR	Island	cg24721964	− 0.433	3	CLRN1	TSS1500	Open sea
cg15139588	0.547	19	ZNF793	5′UTR	Island	cg01437515	− 0.427	1	TNFRSF4	Body	N_Shore

**Table 3:** List of top 20 candidate hypermethylated/hypomethylated genes in colorectal cancers

Differentially hypermethylated/hypomethylated genes					
CpG ID	Δβ	Chromosome	Gene name	Gene-based region	CpG island-based region
cg21101720	0.632	17	ANKRD13B	Body	Island
cg13895235	0.594	7	PRKAR1B	5′UTR	S_Shore
cg07136998	0.540	5	SLIT3	5′UTR	Island
cg12628196	0.527	7	SND1	Body	Island
cg21277995	0.527	6	IRF4	Body	Island
cg13001868	0.521	17	C17orf46	Body	Island
cg13267264	0.518	8	PRDM14	TSS200	Island
cg20219457	0.516	13	DCLK1	1stExon	Island
cg14650610	0.513	5	SPOCK1	5′UTR	Island
cg00421139	0.513	8	GDF6	1stExon	Island
cg21842523	0.511	11	LMO1	Body	Island
cg03394150	0.510	16	GSG1L	Body	Island
cg07974511	0.509	2	OTX1	Body	Island
cg16601494	0.504	1	C1orf70	5′UTR	N_Shore
cg04883903	0.504	12	NOS1	5′UTR	Island
cg04455164	0.501	12	DPY19L2	TSS1500	Island
cg00817367	0.498	12	GRASP	Body	Island
cg17362861	0.498	7	WDR86	TSS200	Island
cg14763548	0.497	20	VSX1	1stExon	Island
cg13484546	0.497	1	FBLIM1	TSS1500	N_Shore
cg09701880	− 0.476	2	LOC541471	Body	Island
cg13324103	− 0.448	10	SVIL	5′UTR	Open sea
cg00863099	− 0.420	2	NCRNA00152	3′UTR	Island
cg11838152	− 0.419	13	ITGBL1	1stExon	Island

### Differential methylation gene GO and KEGG analysis

**Fig. 5:**
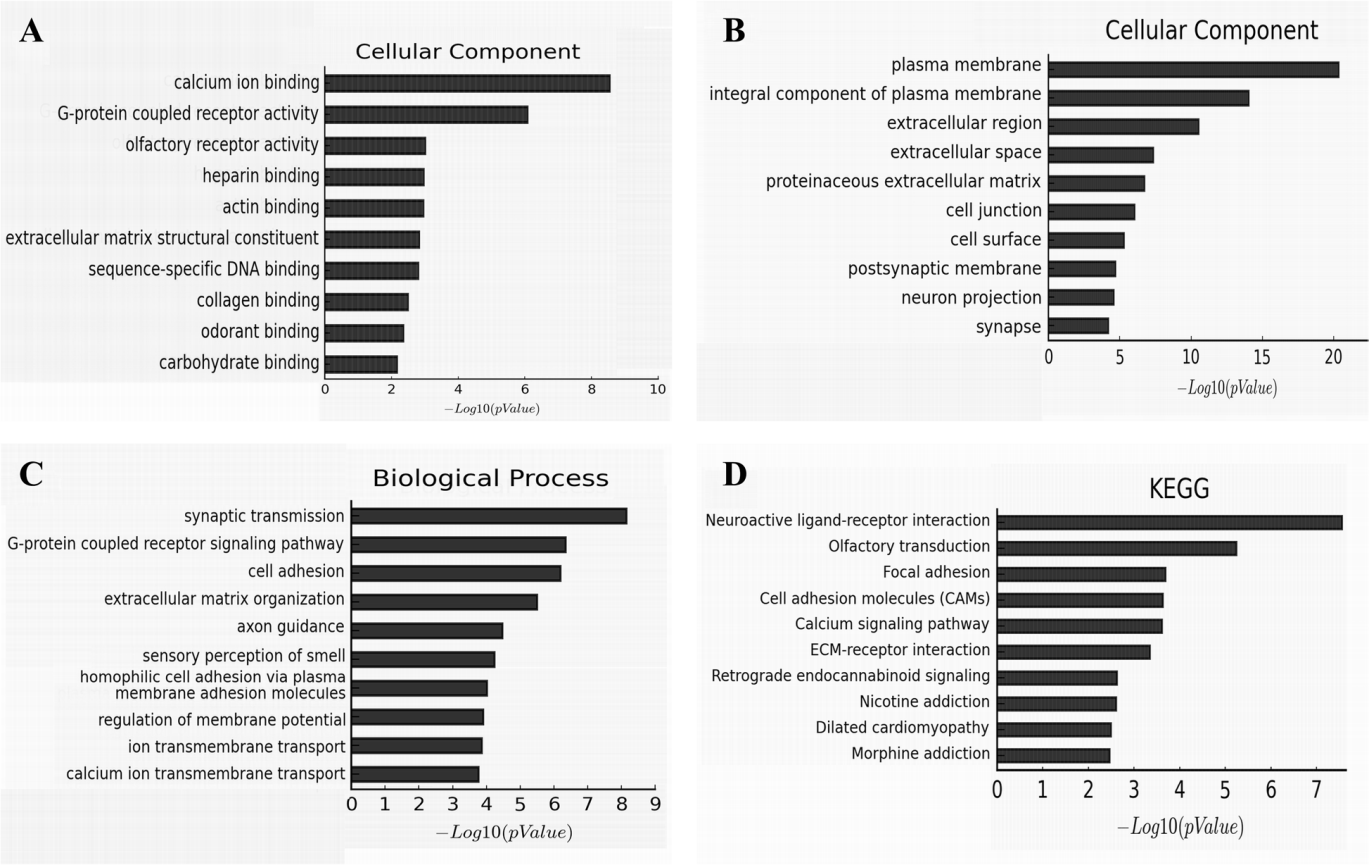
Differential methylation gene GO and KEGG analysis of differentially methylated sites-targets. **A** GO analysis of differentially methylated sites-target genes according to molecular function. **B** GO analysis of differentially methylated sites-target genes according to the cellular component. **C** GO analysis of differentially methylated sites-target genes according to biological process. **D** KEGG pathway-based analysis of differentially methylated sites-target genes

GO analysis was performed on the genes corresponding to differential methylation sites, and the molecular function of the genes was described. In the colorectal cancer group, synaptic transmission, axon guiding, cell adhesion, G-protein-coupled receptor signaling pathway, and extracellular matrix tissue were the five most affected biological processes (Fig. 5C). Calcium binding, G protein-coupled receptor activity, sequence-specific DNA binding, transcriptional activator activity, and sequence-specific binding of the proximal region of the RNA polymerase II core promoter are the five most affected molecular functions (Fig. 5A). According to the analysis of cell components, the overall component of the plasma membrane, cell junction, extracellular region, postsynaptic membrane, and cell components were the five most affected cell components (Fig. 5B). It was found in the adenoma group that synaptic transmission, G-protein-coupled receptor signaling pathway, cell adhesion, extracellular matrix organization, and axonal orientation were the five most affected biological processes, while plasma membrane, integration component of the observed plasma membrane, extracellular membrane, and so on were the most affected cellular components.

Pathway analysis of genes corresponding to differentially methylated sites was conducted using the KEGG database, and the significance of gene enrichment in each pathway entry was calculated by the statistical test. Differentially methylated genes in colorectal cancer were mainly enriched in neuroactive ligand-receptor interaction, morphine addiction, ECM receptor interaction, retrograde endocannabinoid signal transduction, olfactory transduction, and other signal transduction pathways (Table 4). According to KEGG pathway enrichment analysis, abnormally methylated genes were observed to be mainly concentrated in signal transduction pathways such as neuroactive ligand-receptor interaction, olfactory transduction, adhesion plaques, cell adhesion molecules, and calcium signaling pathways in adenomas (Table 5, Fig. 5D). Promoter differential methylation genes are mainly enriched in signal transduction pathways such as neural active ligand-receptor interaction, nicotine addiction, calcium signaling pathway, cholinergic synapses, and ECM receptor interaction. Differentially methylated genes are mainly enriched in signal transduction pathways such as olfactory transduction, retrograde endocannabinoid signaling, and neural active ligand-receptor interaction, as shown in Table 6.

**Table 4:** KEGG pathway analysis of the specific category of genes in colorectal cancers

Term ID	Term description	Number of genes	*P*-value
hsa04080	Neuroactive ligand-receptor interaction	180	1.1E − 10
hsa05032	Morphine addiction	66	1.3E − 05
hsa04512	ECM-receptor interaction	62	2.6E − 05
hsa04723	Retrograde endocannabinoid signaling	69	3.2E − 05
hsa04740	Olfactory transduction	203	3.3E − 05

**Table 5:** KEGG pathway analysis of the specific category of genes in adenomas

Pathway term ID	Term description	Number of genes	*P*-value
hsa04080	Neuroactive ligand-receptor interaction	213	2.73E − 08
hsa04740	Olfactory transduction	270	5.68E − 06
hsa04510	Focal adhesion	144	0.000
hsa04514	Cell adhesion molecules (CAMs)	106	0.000
hsa04020	Calcium signaling Pathway	128	0.000

**Table 6:** KEGG pathway analysis of the specific category of hypomethylated genes in adenomas

Term description	Number of genes	FDR	*P*-value
Olfactory transduction	25	3.8E − 1	3.1E − 4
Retrograde endocannabinoid signals	9	8.8E0	7.3E − 3
Neuroreactive ligand-receptor interactions	16	1.3E1	1.1E − 2

### Cluster analysis

**Fig. 6:**
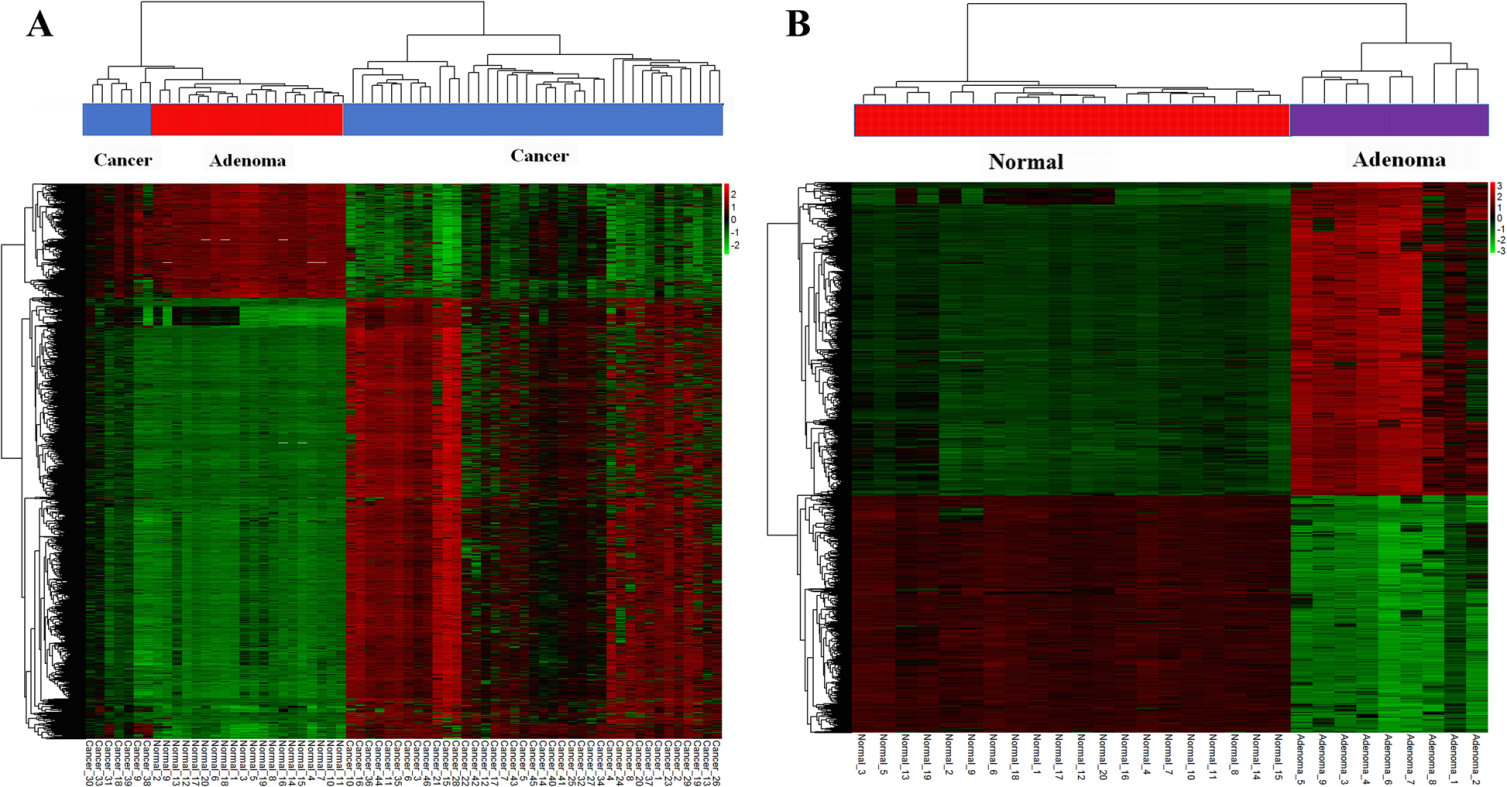
Clustering analysis of differential CpGs methylation marker in colorectal cancer and adenoma tissues. **A** Unsupervised hierarchical clustering and heat map associated with the methylation profile of the colorectal cancer specimens. **B** Unsupervised hierarchical clustering and heat map associated with the methylation profile of the adenoma specimens. The relative methylation level is depicted according to the color scale. Red indicates hypermethylation; green indicates hypo-methylation. − 3, 0, and 3 are fold changes in the corresponding spectrum

Unsupervised hierarchical clustering of differentially methylated genes was performed in the present study. The distance between several samples is calculated to form a distance matrix, and the nearest two classes are combined into a new class. The distance between the new class and the current classes is calculated and then combined and calculated until there is only one class. The direct correlation of the samples is calculated by the expression of the selected differential methylation sites. Genes gathered in the same cluster may have similar biological functions, shown in the heatmap (Fig. 6).

### Screening and detection of methylated gene markers in colorectal cancer and adenoma

For the methylation markers obtained from colorectal cancer, adenoma, and normal controls, due to a large number of differential methylation sites, it is complicated for follow-up studies. To screen the differential hypomethylation genes with repeatability, function, and diagnostic value from a large number of differential methylation sites as markers, we prefer to combine the methylation chip data based on a previous study [7]. Methylated gene markers were screened according to the following conditions: the Δβ absolute value of differential methylation at CpG sites in microarray data was ≥ 0.4, non-gene markers were deleted, and the average methylation level β in hypermethylated markers was ≤ 0.1 in the normal group and ≤ 0.2 in the adenoma group where hypomethylated markers were located. In combination with GO and KEGG, the marker with any of the first five items can be a candidate gene methylation marker. In addition, we restricted the marker gene to one of the first three entries in GO analysis and KEGG pathway analysis to further select the marker. Based on the above criteria, 181 hypermethylated genes and 28 hypomethylated genes were screened out in the adenoma group. In addition, this project specifically screens promoter region and genome hypomethylation markers. First of all, for hypomethylation markers, we further restrict the criteria to a total of 15 genes conforming to one of the first three items of GO and pathway analysis, which are the following: SMAD3, DNAJB6, ATXN1, TCF15, TNR, GABRR2, EXD3, AKAP13, ARNT2, RAPGEF4, IL5RA, SPTBN5, INPP5A, ANK2, PRDM16. According to the gene promoter region, there were 1524/346 high/low methylation sites, including 929 genes, among which the top 15 high/low methylation genes were LRRC4, NPY, DCLK1 CLIP4 PRKAR1B, ZNF542, FLI1, LOC389333, ITGA4, GATA5, KHDRBS2, FAM115A, CNRIP1, PTPRT, GA. OR2M3, CHRM2, C7orf16, MACROD2, POLD3, HPVC1, LRRIQ4, TNR, OR56A1, TCN1, SVIL, ACMSD, SEC23B, SMAD3, and C7orf66.

**Fig. 7:**
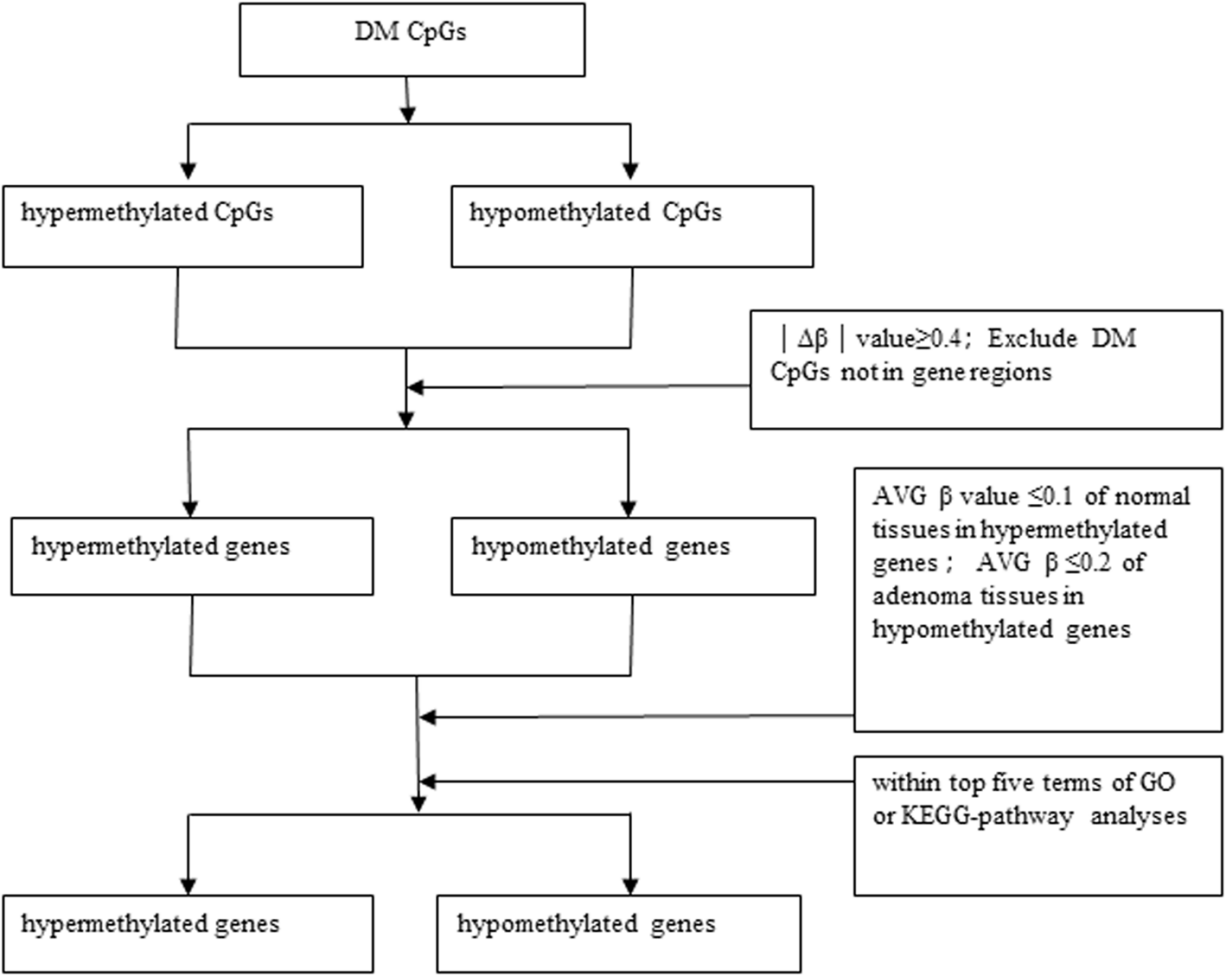
Steps and parameters for differentially candidate biomarker genes selection in adenomas and colorectal cancers

**Fig. 8:**
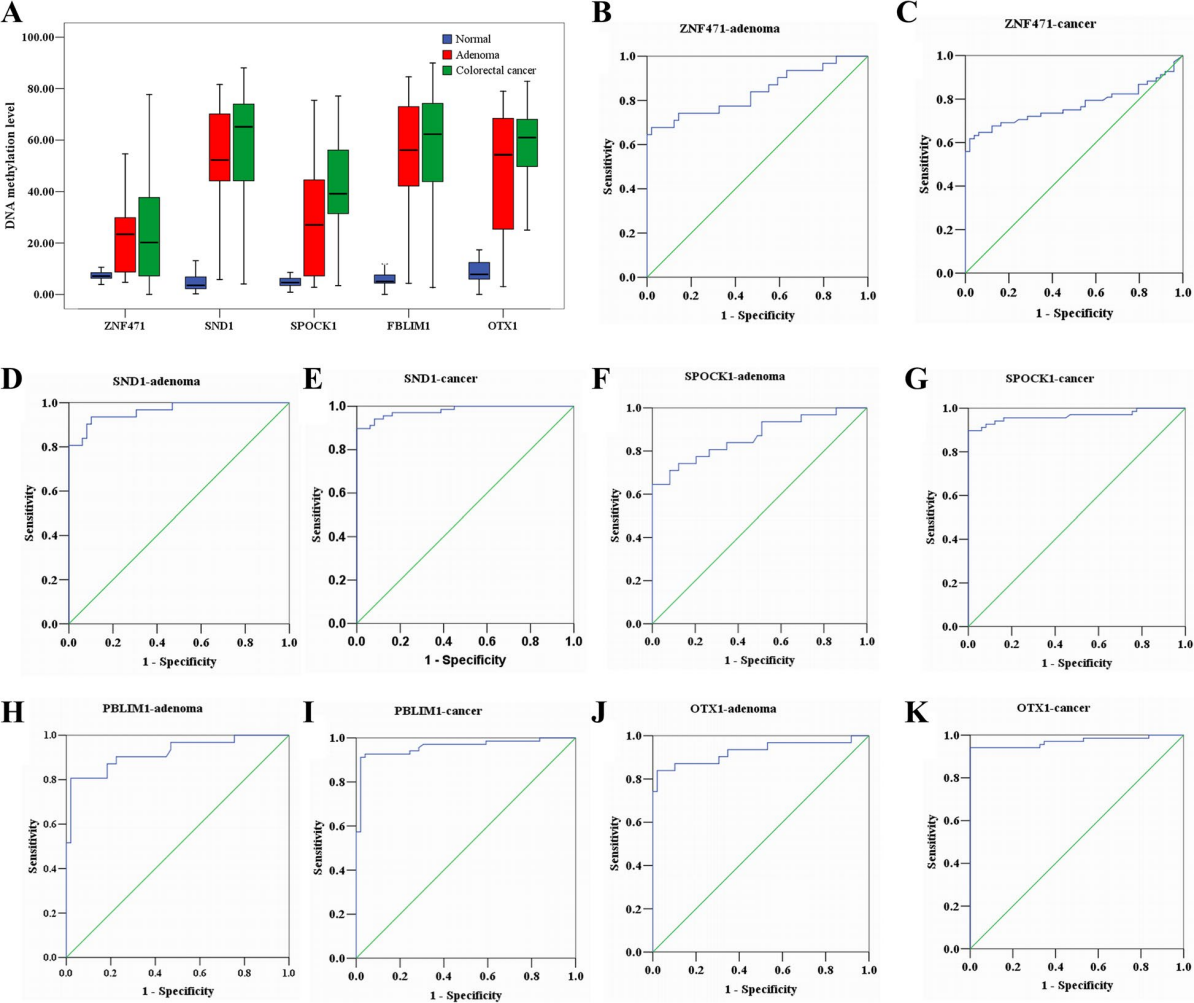
Screening and detection of methylated gene markers in colorectal cancer and adenoma. **A** Box plot of methylation indices for colorectal adenoma and colorectal cancer analyzed by pyrosequencing. The methylation index (MI) of each gene is presented for normal control, adenoma, and colorectal cancer as a boxplot. Whiskers of the boxplot mark the 5 and 95 percentiles; the box marks the 25 (low boundary of box), median, and 75 (upper boundary of box) percentiles and extreme values. **B** Receiver operator curve (ROC) analysis for ZNF471 predicting aberrant methylation in adenoma. **C** ROC analysis for ZNF471 predicting aberrant methylation in colorectal cancer. **D** ROC analysis for SND1 predicting aberrant methylation in adenoma. **E** ROC analysis for SND1 predicting aberrant methylation in colorectal cancer. **F** ROC analysis for SPOCK1 predicting aberrant methylation in adenoma. **G** ROC analysis for SPOCK1 predicting aberrant methylation in colorectal cancer. **H** ROC analysis for FBLIM1 predicting aberrant methylation in adenoma. **I** ROC analysis for FBLIM1 predicting aberrant methylation in colorectal cancer. **J** ROC analysis for OTX1 predicting aberrant methylation in adenoma. **K** ROC analysis for OTX1 predicting aberrant methylation in colorectal cancer

According to the screening conditions of the adenoma group, 151 hypermethylated genes and five hypomethylated genes were screened out from the methylated markers of colon cancer and the normal control group. By further analyzing the markers shared by adenoma and cancer, 61 hypermethylated genes and five hypomethylated genes were screened out. Steps and parameters for differentially candidate biomarker genes selection in adenomas and colorectal cancers were shown in Fig. 7. According to the above data and preliminary analysis of the data, five gene methylation markers (ZNF471, SND1, SPOCK1, FBLIM1, and OTX1) have been detected in the colorectal adenoma, colorectal cancer, and control groups. Pyrosequencing was used to detect the methylation degree of 5 gene methylation markers in 68 cases of colorectal cancer, 31 cases of adenoma, and 49 cases of the normal control group. Correlations of colorectal cancer patient characteristics with methylation of ZNF471, SND1, SPOCK1, FBLIM1, and OTX1 were shown in Tables S3, S4, S5, S6 and S7. The results are shown in Table 7 and Fig. 8A. The results of receiver operator curve (ROC) analysis for each candidate gene predicting aberrant methylation in colorectal adenoma and colorectal cancer are shown in Fig. 8B–K. The methylation degree of ZNF471 was more than 2.9-fold, SND1 was more than 14.5-fold, SPOCK1 was more than 5.8-fold, FBLIM1 was more than 11.1-fold, and OTX1 was more than 6.9-fold in colorectal cancer or colorectal adenoma to that in the normal control. It is essential that the methylation degree of SND1 was more than 18.1-fold and FBLIM1 was more than 12.4-fold in colorectal cancer to that in the normal control.

**Table 7:** Distribution of DNA methylation Index among six genes in colorectal cancers, adenomas and normal controls

	Colorectal cancer (*N* = 68)			Colorectal adenoma (*N* = 31)			Normal control (*N* = 49)			*P* value^a^	*P* value^b^
Gene	Mean ± SD	Median	Range	Mean ± SD	Median	Range	Mean ± SD	Median	Range		
ZNF471	24.32 ± 19.91	20.20	0.00–77.69	22.69 ± 13.28	23.36	4.74–54.63	6.60 ± 2.17	6.91	0.00–11.93	< 0.001	< 0.001
SND1	58.16 ± 22.38	65.18	4.11–88.11	51.20 ± 23.84	52.30	3.83–81.59	5.42 ± 4.62	3.59	0.23–19.77	< 0.001	< 0.001
SPOCK1	41.70 ± 19.54	39.13	3.41–77.11	27.47 ± 20.49	27.04	2.78–75.50	5.32 ± 2.84	4.63	0.87–13.43	< 0.001	< 0.001
FBLIM1	56.94 ± 23.30	62.29	2.71–89.92	50.55 ± 24.49	56.11	5.03–76.27	7.15 ± 8.14	5.02	0.00–76.07	< 0.001	< 0.001
OTX1	57.27 ± 18.10	60.95	5.01–82.83	47.26 ± 24.44	54.29	3.10–78.97	9.02 ± 4.78	7.81	0.00–23.06	< 0.001	< 0.001

^a,b^Summary statistics with a nonparametric Mann–Whitney test comparing colorectal cancers with normal control (a) and adenomas with normal control (b) for each gene

## Discussion

Early diagnosis with markers improves the prognosis of patients with CRC. DNA methylation was concerned to play an essential role in tumors occurrence, development, and metastasis. The characteristic methylation sites are of great significance for the diagnosis, typing, prognosis, and treatment of tumors including CRC. The gene CpG island promoter is often hypermethylated, leading to tumor suppressor gene silencing. DKK2 promoter methylation and related RNA status were suggested as biomarkers of CRC diagnosis [8]. SDC2 and TFPI2 methylation, which was affected by tumor location, patient age, mutation load, and microsatellite instability (MSI), may be considered methylation markers for CRC detection [9]. Promoter methylation-mediated repression of UNC5C and UNC5D correlated with poor progression-free and overall survival, which has good relation to diagnosis and prognosis in CRC [10]. It was reported that many genes in colorectal cancer, such as p14, p16, p53, MGMT, APC, hMLH1, THBS1, E-cadherin, TIMP3, and GSTP1, have the presence of promoter CpG island hypermethylation, which is considered to be an early event in colorectal cancer [11, 12]. Therefore, the methylation profile of CRC to reveal the correlation may be helpful for early diagnosis and prognosis of CRC.

Adopting a new genomic methylation detection method is more conducive to a comprehensive analysis of the methylation profile of CRC to further reveal its mechanism of action in the canceration process and discover new methylation markers for CRC diagnosis and prognosis. The methylation chip of Illumina Infinium 450K detection technology, covering a detection range of more than 485,000 CpG sites, is a genome-wide methylation detection analysis method with powerful detection function, has high throughput, has high sensitivity, and has the advantages of high accuracy and good repeatability [13, 14]. Recently, the Illumina Infinium 450K methylation chip has been used to obtain many new methylation markers in CRC detection [7]. In the present study, the Illumina Infinium 450K methylation chip was used to detect gene methylation in CRC and adenoma compared with the normal group. A large number of different methylation sites were found in CRC, including 65,535 (13.50%) differentially methylated markers in the adenoma group, among which 25,464 (38.86%) were hypermethylated markers and 40,071 (61.14%) were hypomethylated markers, covering a total of 8541 differentially methylated genes as well as 395,571 (8.15%) differential methylation marker probes which were compared between the sporadic colorectal cancer group and the normal group, including 21,710 (54.86%) hypermethylated markers and 17,861 (45.14%) hypomethylated markers, covering a total of 3551 differential methylation genes. 318,208 differential methylation markers were further found in the CRC and adenoma compared with the normal group in our study. The top 20 hypermethylated CpG sites mapping to 15 genes and 20 hypomethylated CpG sites mapping to 20 genes were identified according to Δβ values in the adenoma group, the results of which showed that the hypermethylated CpG sites were mainly located in the CGI region, and the hypomethylated CpG sites were primarily located in the gene body region. Our results suggested that hypermethylation may play a more important role in promoter regions, while hypomethylation may act in gene body regions and CpG off-island regions. Our results were consistent with the previous studies that the majority of methylation including hypomethylation and hypermethylation occurring during adenoma formation may play an essential role in tumorigenesis and progression [15–17]. According to previous studies, some genes as diagnostic markers were selected through screening in colorectal adenoma and colorectal cancer. One hundred eighty-one hypermethylated genes and 28 hypomethylated genes were screened among the methylation markers obtained from the adenoma and normal control, and 151 hypermethylated genes and five hypomethylated genes were screened among the methylation markers of the colon cancer and normal control groups. Moreover, among the typical markers of adenoma and cancer, 61 hypermethylated genes and four hypomethylated genes were screened.

Abnormal methylation of tumor suppressor gene promoter is one of the most essential methylation events; it often leads to the silencing of tumor suppressor genes, which plays a vital role in the occurrence and development of cancer. The distribution of gene promoter structure and CpG island structure were then compared with that of the normal group utilizing microarray data combined with bioinformatics analysis in the present study, the results of which showed that there were a large number of differential methylation markers in colorectal cancer and adenoma. One thousand eight hundred seventy differential methylated markers including 1524 hypermethylated markers and 346 hypomethylated markers were found in the gene promoter region in colorectal adenomas compared with the normal group. Among the top 20 promoter hypermethylated genes, 15 genes have been reported methylated including ten genes detected in bowel cancer and five genes detected in adenoma. Among the top 20 promoter hypomethylated genes, nine genes have been reported methylated, including five genes detected in bowel cancer and four genes detected in adenoma. GO and KEGG pathway analyses of 929 promoter differential methylated genes showed that they covered many different functional communities. GO analysis results showed that promoter differential methylated genes were mainly enriched in chemical synaptic transmission, nervous system development, extracellular matrix organization, extracellular matrix structural components, RNA polymerase II regulatory region sequence-specific DNA binding, sequence-specific DNA binding, plasma membrane, protein extracellular matrix, cell membrane, and other annotation topics. The KEGG pathway analysis showed that promoter differential methylation genes are mainly involved in ligand-receptor interactions, nicotine addiction, calcium signaling, and other signal transduction pathways of neural activity. These results indicate that there are many types of genes involved in the regulation of signal transduction pathways in the occurrence and development of CRC. Still, the specific mechanisms need to be further studied.

Based on the results of the methylation chip of Illumina Infinium 450K detection and bioinformatic analysis, five hypermethylated genes, ZNF471, SND1, SPOCK1, FBLIM1, and OTX1, were selected for further investigation in the colorectal adenoma, CRC, and control normal tissues in our study. Our results showed that all the genes of ZNF471, SND1, SPOCK1, FBLIM1, and OTX1 methylation with the area under receiver operating curve (ROC) more than 0.90 were significantly high in the colorectal adenoma and CRC compared with the normal group. ZNF471 methylation was found in tongue squamous cell carcinoma and may be served as a diagnostic marker in the previous study [18]. In another study, hypermethylation of the ZNF471 gene promoter was inversely correlated with its expression, and overexpression of ZNF471 inhibited EMT and acted as a tumor suppressor with diagnostic and prognostic significance in cervical cancer [19]. Our results similarly indicated that ZNF471 methylation level was higher in CRC and colorectal adenoma than the normal group which suggested that our results for ZNF471 appear consistent with the above previous study and indicate this gene might display good diagnostic value as a tumor suppressor for both colorectal cancer and adenomas. As an N6-methyladenosine (m6A) reader, SND1 is associated with methylation and can modify mRNA and regulate target mRNA stability [20]. SND1 expression was increased in tumors such as bladder cancer [21], glioma [22], and ovarian cancer [23] and correlated with proliferation and metastasis as well as chemoresistance. As previously reported, SND1 hypermethylation was found in CRC, which was consistent with our results of the present study [7]. SPOCK1 was significantly highly expressed in liver cancer [24], pancreatic cancer [25], and colon cancer cells [26] and correlated with tumor immune infiltrates in colorectal cancer as one of the valuable prognostic biomarkers [27]. SPOCK1 was reported to connect with tumor immune infiltrates and may be a useful prognostic biomarker and act a carcinogenic gene in CRC. SPOCK1 hypomethylation was found in the placentas of women with the HbSS genotype. The promoter CpG islands of SPOCK1 were hypermethylated and promoted tumor progress in CRC [26, 28] similar to our research. FBLIM1 was mentioned to enhance oral cancer malignancy in a previous study [29] and may contribute to the diagnosis of hepatocellular cancer [30]. FBLIM1 was hypomethylated and could reduce cell proliferation in non-alcoholic fatty liver disease [31]. In our study, hypermethylated FBLIM1 was first found in CRC and colorectal adenoma and the role of FBLIM1 in tumor development and progress is worth further study. As previously reported [32, 33], OTX1 was hypermethylated in cancers such as lung, squamous cell carcinomas and breast cancer. Hypermethylated OTX1 was detected in our study, which may be associated with its oncogene function in CRC similar to a previous study that OTX1 promotes CRC progression [34]. Our results indicated that ZNF471, SND1, SPOCK1, FBLIM1, and OTX1 methylation may be useful in diagnosing colorectal cancer and adenoma. However, the diagnostic value of each marker has been preliminarily analyzed presently. The relationship between the above markers and clinicopathological factors in colorectal cancer and adenoma and the mechanism in the occurrence and development of tumors is still worthy of investigation in our further study.

## Conclusion

Hypermethylated genes of ZNF471, SND1, SPOCK1, FBLIM1, and OTX1 were obtained from methylation chip detection and further confirm analysis in colorectal cancer and adenoma compared with normal tissue, which may be promising diagnostic markers of CRC and colorectal adenoma.

### Supplementary Information

**Additional file 1: Supplement 1.** QIAamp® DNA Mini and Blood Mini Handbook.

**Additional file 2: Supplement 2.** EZ DNA Methylation™ Kit.

**Additional file 3: Supplement 3.** EpiTect® Bisulfite Handbook.

**Additional file 4: Supplement 4.** EpiTect Bisulfite Kit.

**Additional file 5: Table S1.** Patient characteristics.

**Additional file 6: Table S2.** Patient characteristics.

**Additional file 7: Table S3.** Correlations of clinical characteristics with methylation status of ZNF471 in colorectal cancers.

**Additional file 8: Table S4.** Correlations of clinical characteristics with methylation status of SND1 in colorectal cancers.

**Additional file 9: Table S5.** Correlations of clinical characteristics with methylation status of SPOCK1 in colorectal cancers.

**Additional file 10: Table S6.** Correlations of clinical characteristics with methylation status of FBLIM1 in colorectal cancers.

**Additional file 11: Table S7.** Correlations of clinical characteristics with methylation status of OTX1 in colorectal cancers.

**Additional file 12: Table S8.** The primers of methylation markers.

**Additional file 13.** Infinium HD assay methylation protocol guide.

## Data Availability

All the data would be supplied by the corresponding author if required.
