# Supplementary material for: Screening and identifying of biomarkers in early colorectal cancer and adenoma based on genome-wide methylation profiles
Source: World J Surg Oncol. 2023 Oct 2;21:312. doi: 10.1186/s12957-023-03189-1 (PMC10544418; doi:10.1186/s12957-023-03189-1)
Supplement: Supplementary file 1 — Additional file 1: Supplement 1. QIAamp® DNA Mini and Blood Mini Handbook. [file 12957_2023_3189_MOESM1_ESM.pdf]

Third Edition April 2010

QIAamp® DNA Mini and Blood Mini Handbook

For DNA purification from
- whole blood
- plasma
- serum
- buffy coat
- lymphocytes
- dried blood spots (QIAamp DNA Mini Kit only)
- body fluids
- cultured cells
- swabs
- tissue (QIAamp DNA Mini Kit only)

QIAGEN®

Sample & Assay Technologies

Trademarks: QIAGEN®, QIAamp®, QIAcube®, FlexiGene®, Gentra Puregene®, InhibitEX®, MinElute®, TissueRuptor® (QIAGEN Group); Amicon®, Centricon®, UltraFree® (Millipore Corporation); DACRON® (E. I. du Pont de Nemours and Company); Puritan® (Hardwood Products Company); Triton® (Rohm and Haas Company); Tween® (ICI Americas Inc.).

© 1999–2010 QIAGEN, all rights reserved.

FSC
www.fsc.org
MIX
Papier aus verantwortungsvollen Quellen
FSC® C009997

QIAGEN is a member of the Forest Stewardship Council (FSC). For the production of printed materials, including handbooks, QIAGEN has a policy to select suppliers that comply with FSC standards for printing processes and well-managed forests.

Contents

**Kit Contents**	**5**
**Storage**	**6**
**Quality Control**	**6**
**Product Use Limitations**	**7**
**Product Warranty and Satisfaction Guarantee**	**7**
**Technical Assistance**	**7**
**Safety Information**	**8**
**Introduction**	**9**
Purification of viral RNA and DNA	9
Purification of DNA from urine	10
Purification of DNA from stool	10
Purification of DNA from formalin-fixed, paraffin-embedded tissues	10
Purification of DNA from forensic and human identity samples	11
Purification of high-molecular-weight DNA	11
Processing of large sample volumes	11
High-throughput sample processing	11
Principle and procedure	12
Purification on QIAamp Mini spin columns	12
Automated DNA purification on the QIAcube	15
**Equipment and Reagents to Be Supplied by User**	**16**
**Important Notes**	**17**
Preparation of reagents	17
Amounts of starting material	18
Preparation of buffy coat	19
Handling of QIAamp Mini columns	19
Copurification of RNA	24
Elution mode for maximum yield or concentration	25
**Protocols**	
■ **DNA Purification from Blood or Body Fluids (Spin Protocol)**	**27**
■ **DNA Purification from Blood or Body Fluids (Vacuum Protocol)**	**30**
■ **DNA Purification from Tissues (QIAamp DNA Mini Kit)**	**33**
■ **DNA Purification from Buccal Swabs (Spin Protocol)**	**37**

- DNA Purification from Buccal Swabs (Vacuum Protocol) 40
- DNA Purification from Dried Blood Spots (QIAamp DNA Mini Kit) 43

Troubleshooting Guide 45
Appendix A: Determination of Concentration, Yield, Purity, and Length of DNA 51
Appendix B: Protocol for Cultured Cells 51
Appendix C: Protocols for Fixed Tissues 53
Appendix D: Protocols for Bacteria 55
Appendix E: Protocol for Yeast (e.g., Cultured *Candida* spp.) 57
Appendix F: Protocols for Viral DNA 59
Appendix G: Protocols for Eye, Nasal, or Pharyngeal Swabs 61
Appendix H: Protocol for Mitochondrial DNA from Platelets 62
Appendix I: Protocol for CSF and Bone Marrow on Hematological Slides 63
Appendix J: Protocol for Crude Cell Lysates and Other Samples 64
Appendix K: Protocol for Sample Concentration 66
Ordering Information 67

Kit Contents

QIAamp DNA Kits<br>Catalog no.<br>Number of preps	Blood Mini (50)<br>51104<br>50	Blood Mini (250)<br>51106<br>250	Mini (50)<br>51304<br>50	Mini (250)<br>51306<br>250
QIAamp Mini Spin Columns	50	250	50	250
Collection Tubes (2 ml)	150	750	150	750
Buffer AL*	12 ml	54 ml	12 ml	54 ml
Buffer ATL	–	–	10 ml	50 ml
Buffer AW1* (concentrate)	19 ml	95 ml	19 ml	95 ml
Buffer AW2† (concentrate)	13 ml	66 ml	13 ml	66 ml
Buffer AE	12 ml	60 ml	22 ml	110 ml
QIAGEN® Protease	1 vial‡	1 vial§	–	–
Protease Solvent†	1.2 ml	5.5 ml	–	–
Proteinase K	–	–	1.25 ml	6 ml

* Contains chaotropic salt. Not compatible with disinfecting agents containing bleach; see page 8 for safety information.

† Contains sodium azide as a preservative.

‡ Resuspension volume 1.2 ml.

§ Resuspension volume 5.5 ml.

Storage

QIAamp Mini spin columns and buffers can be stored dry at room temperature (15–25°C) for up to 1 year without showing any reduction in performance.

Lyophilized QIAGEN Protease can be stored at room temperature (15–25°C) for up to 12 months without any decrease in performance. For storage longer than 12 months or if ambient temperatures constantly exceed 25°C, QIAGEN Protease should be stored dry at 2–8°C.

Reconstituted QIAGEN Protease is stable for 2 months when stored at 2–8°C. Keeping the QIAGEN Protease stock solution at room temperature for prolonged periods of time should be avoided. Storage at –20°C will prolong its life, but repeated freezing and thawing should be avoided. Dividing the solution into aliquots and storage at –20°C is recommended.

QIAamp DNA Mini Kits contain ready-to-use proteinase K solution, which is dissolved in a specially formulated storage buffer. The proteinase K is stable for up to 1 year after delivery when stored at room temperature. To prolong the lifetime of Proteinase K, storage at 2–8°C is recommended.

Quality Control

In accordance with QIAGEN's ISO-certified Quality Management System, each lot of QIAamp DNA Mini Kits and QIAamp DNA Blood Mini Kits is tested against predetermined specifications to ensure consistent product quality.

Product Use Limitations

The QIAamp DNA Mini Kit and QIAamp DNA Blood Mini Kit are intended for molecular biology applications. These products are not intended for the diagnosis, prevention, or treatment of a disease.

All due care and attention should be exercised in the handling of the products. We recommend all users of QIAamp Kits to adhere to the NIH guidelines that have been developed for recombinant DNA experiments, or to other applicable guidelines.

Product Warranty and Satisfaction Guarantee

QIAGEN guarantees the performance of all products in the manner described in our product literature. The purchaser must determine the suitability of the product for its particular use. Should any product fail to perform satisfactorily due to any reason other than misuse, QIAGEN will replace it free of charge or refund the purchase price. We reserve the right to change, alter, or modify any product to enhance its performance and design. If a QIAGEN product does not meet your expectations, simply call your local Technical Service Department or distributor. We will credit your account or exchange the product — as you wish. Separate conditions apply to QIAGEN scientific instruments, service products, and to products shipped on dry ice. Please inquire for more information.

A copy of QIAGEN terms and conditions can be obtained on request, and is also provided on the back of our invoices. If you have questions about product specifications or performance, please call QIAGEN Technical Services or your local distributor (see back cover or visit www.qiagen.com).

Technical Assistance

At QIAGEN we pride ourselves on the quality and availability of our technical support. Our Technical Service Departments are staffed by experienced scientists with extensive practical and theoretical expertise in sample and assay technologies and the use of QIAGEN products. If you have any questions or experience any difficulties regarding QIAamp DNA Mini or QIAamp DNA Blood Mini Kits or QIAGEN products in general, please do not hesitate to contact us.

QIAGEN customers are a major source of information regarding advanced or specialized uses of our products. This information is helpful to other scientists as well as to the researchers at QIAGEN. We therefore encourage you to contact us if you have any suggestions about product performance or new applications and techniques.

For technical assistance and more information, please see our Technical Support Center at www.qiagen.com/goto/TechSupportCenter or call one of the QIAGEN Technical Service Departments or local distributors (see back cover or visit www.qiagen.com).

Safety Information

When working with chemicals, always wear a suitable lab coat, disposable gloves, and protective goggles. For more information, please consult the appropriate material safety data sheets (MSDSs). These are available online in convenient and compact PDF format at www.qiagen.com/Support/MSDS.aspx where you can find, view, and print the MSDS for each QIAGEN kit and kit components.

CAUTION: DO NOT add bleach or acidic solutions directly to the sample-preparation waste.

The sample-preparation waste contains guanidine hydrochloride from Buffers AL and AW1, which can form highly reactive compounds when combined with bleach.

If liquid containing these buffers is spilt, clean with suitable laboratory detergent and water. If the spilt liquid contains potentially infectious agents, clean the affected area first with laboratory detergent and water, and then with 1% (v/v) sodium hypochlorite.

The following risk and safety phrases apply to components of the QIAamp DNA Mini and QIAamp DNA Blood Mini Kits.

Buffers AW1 and AL

Contain guanidine hydrochloride: harmful, irritant. Risk and safety phrases:* R22-36/38, S13-26-36-46

Proteinase K (QIAamp DNA Mini Kit only)

Contains proteinase K: Sensitizer, irritant. Risk and safety phrases:* R36/37/38-42/43, S23-24-26-36/37

QIAGEN Protease (QIAamp DNA Blood Mini Kit only)

Contains subtilisin: sensitizer, irritant. Risk and safety phrases:* R37/38-41-42, S22-24-26-36/37/39-46

24-hour emergency information

Emergency medical information can be obtained 24 hours a day in English, French, and German from:

Poison Information Center Mainz, Germany

Tel.: +49-6131-19240

* R22: Harmful if swallowed; R36/38: Irritating to eyes and skin; R36/37/38: Irritating to eyes, respiratory system and skin; R37/38: Irritating to respiratory system and skin; R41: Risk of serious damage to eyes; R42: May cause sensitization by inhalation; R42/43: May cause sensitization by inhalation and skin contact; S13: Keep away from food, drink and animal feedingstuffs; S22: Do not breathe dust; S23: Do not breathe vapor; S24: Avoid contact with skin; S26: In case of contact with eyes, rinse immediately with plenty of water and seek medical advice; S36: Wear suitable protective clothing; S36/37: Wear suitable protective clothing and gloves; S36/37/39: Wear suitable protective clothing, gloves, and eye/face protection; S46: If swallowed, seek medical advice immediately and show container or label.

Introduction

QIAamp DNA Mini and QIAamp DNA Blood Mini Kits provide fast and easy methods for purification of total DNA for reliable PCR and Southern blotting. Total DNA (e.g., genomic, viral, mitochondrial) can be purified from whole blood, plasma, serum, buffy coat, bone marrow, other body fluids, lymphocytes, cultured cells, tissue, and forensic specimens.

The simple QIAamp spin and vacuum procedures, which are ideal for simultaneous processing of multiple samples, yield pure DNA ready for direct amplification in just 20 minutes. The QIAamp spin procedures can be fully automated on the QIAcube® for increased standardization and ease of use (see page 15). The QIAamp procedure is suitable for use with fresh or frozen whole blood and blood which has been treated with citrate, heparin, or EDTA. Prior separation of leukocytes is not necessary. Purification requires no phenol/chloroform extraction or alcohol precipitation, and involves very little handling. DNA is eluted in Buffer AE or water, ready for direct addition to PCR or other enzymatic reactions. Alternatively, it can be safely stored at –20°C for later use. The purified DNA is free of protein, nucleases, and other contaminants or inhibitors.

DNA purified using QIAamp Kits is up to 50 kb in size, with fragments of approximately 20–30 kb predominating. DNA of this length denatures completely during thermal cycling and can be amplified with high efficiency.

For purification of genomic DNA from blood for in vitro diagnostics in Europe, the QIAamp DSP DNA Blood Mini Kit is CE-IVD-marked, compliant with EU Directive 98/79/EC. This kit is not available in the USA and Canada.

Purification of viral RNA and DNA

For purification of viral RNA, the QIAamp Viral RNA Mini Kit is recommended. All buffers and components are guaranteed to be RNase-free. Alternatively, for simultaneous purification of viral DNA and RNA, we recommend using the QIAamp MinElute® Virus Vacuum Kit or the QIAamp MinElute Virus Spin Kit. These kits provide viral nucleic acid purification with minimal elution volumes for higher sensitivity in downstream applications. All buffers and components of these kits are guaranteed to be RNase-free. Viral nucleic acid purification using the QIAamp MinElute Virus Spin Kit or the QIAamp Viral RNA Mini Kit can be fully automated on the QIAcube for increased standardization and ease of use.

Purification of viral DNA is possible with QIAamp DNA Mini or QIAamp DNA Blood Mini Kits. Since cellular DNA copurifies with viral DNA, cell-free samples (e.g., plasma, serum) are necessary to obtain pure viral DNA. For preparation of DNA from free viral particles in fluids or suspensions (other than urine) using the Blood and Body Fluid protocols, see Appendix F, page 59.

For purification of viral nucleic acids for in vitro diagnostics in Europe, the QIAamp DSP Virus Kit is CE-IVD-marked, compliant with EU Directive 98/79/EC. This kit is not available in the USA and Canada.

Purification of DNA from urine

For preparation of cellular, bacterial, or viral DNA from urine, the QIAamp Viral RNA Mini Kit is recommended. Buffer AVL supplied with this kit is optimized to inactivate the numerous PCR inhibitors found in urine.

Purification of DNA from stool

The QIAamp DNA Stool Mini Kit is recommended for preparation of DNA from stool. Stool samples typically contain many compounds that can degrade DNA and inhibit downstream enzymatic reactions. The QIAamp DNA Stool Mini Kit removes these substances through the action of a proprietary reagent that efficiently adsorbs inhibitors, together with a lysis buffer that provides optimized conditions for inhibitor removal. DNA purification using the QIAamp DNA Stool Mini Kit can be fully automated on the QIAcube for increased standardization and ease of use.

QIAamp DNA Mini or QIAamp DNA Blood Mini Kits can also be used to purify viral DNA from stool, but removal of inhibitors is not as effective. See Appendix F, page 59.

Purification of DNA from formalin-fixed, paraffin-embedded tissues

The QIAamp DNA FFPE Tissue Kit is recommended for purification of DNA from formalin-fixed, paraffin-embedded (FFPE) tissues. The kit combines the selective binding properties of a silica-based membrane with flexible elution volumes of between 20 and 100 µl. Specially optimized lysis conditions allow genomic DNA to be efficiently purified from FFPE tissue sections without the need for overnight incubation.

The QIAamp DNA Mini Kit can also be used for purification of DNA from FFPE tissues that have first been processed as described in Appendix C, page 53.

Purification of DNA from forensic and human identity samples

The QIAamp DNA Investigator Kit is recommended for purification of total (genomic and mitochondrial) DNA from a wide range of forensic and human identity samples, such as casework or crime-scene samples, dried blood, bone, and sexual assault samples, swabs, and filters. Purification is fast and efficient, and purified DNA performs well in downstream analyses, such as quantitative PCR and STR analysis, with high signal-to-noise ratios. The procedure is designed to ensure that there is no sample-to-sample cross-contamination. Purification of DNA using the QIAamp DNA Investigator Kit can be automated on the QIAcube.

Purification of high-molecular-weight DNA

To purify high-molecular-weight DNA, larger than the 50 kb achieved with QIAamp Kits, we recommend using QIAGEN Genomic-Tips or Gentra Puregene® Kits. QIAGEN Genomic-Tips are gravity-flow, anion-exchange tips that enable purification of DNA of up to 150 kb from a wide range of sample types. The tips are available separately or, with QIAGEN Protease and buffers, as part of Blood & Cell Culture DNA Kits.

Gentra Puregene Kits use a modified salting-out precipitation method for purification of archive-quality DNA of 100–200 kb. The procedure is scalable for large or small sample volumes, and kits are available for a wide range of sample types. An ongoing study of archived DNA has shown that purified DNA can be stored for at least 14 years without degradation.

Processing large sample volumes

QIAamp DNA Blood Midi and Maxi Kits are available for purification of DNA from up to 2 ml and 10 ml of blood, respectively. These kits use the same silica-membrane technology as the QIAamp DNA Blood Mini Kit.

The FlexiGene® DNA Kit provides scalable purification of genomic DNA from whole blood, buffy coat, or cultured cells in a single tube. The simple, rapid procedure yields pure DNA of up to 150 kb, ready to use in downstream applications such as PCR or Southern blotting.

Gentra Puregene Kits provide a scalable procedure for large or small sample volumes. The kits use a modified salting-out precipitation method for purification of archive-quality DNA, and kits are available for a wide range of sample types.

High-throughput sample processing

Please contact one of the QIAGEN Technical Service Departments (see back cover), or visit www.qiagen.com for detailed information on high-throughput QIAamp systems and automated solutions.

Principle and procedure

QIAamp DNA Mini and QIAamp DNA Blood Mini Kits are designed for rapid purification of an average of 6 µg of total DNA (e.g., genomic, viral, mitochondrial) from 200 µl of whole human blood, and up to 50 µg of DNA from 200 µl of buffy coat, 5 x 10^6 lymphocytes, or cultured cells that have a normal set of chromosomes. The procedure is suitable for use with whole blood treated with citrate, heparin, or EDTA;* buffy coat; lymphocytes; plasma; serum; and body fluids. Samples may be either fresh or frozen. For larger volumes of whole blood or cultured cells, we recommend using QIAamp DNA Blood Midi or Maxi Kits.

The QIAamp DNA Mini Kit performs all the functions of the QIAamp DNA Blood Mini Kit, and also allows rapid purification of DNA from solid tissue. On average, up to 30 µg of DNA can be purified from 25 mg of various human tissues.

Lysis with QIAGEN Protease or proteinase K

QIAamp DNA Blood Mini Kits contain QIAGEN Protease. Intensive research has shown that QIAGEN Protease is the optimal enzyme for use with the lysis buffer provided in the QIAamp DNA Blood Mini Kit. QIAGEN Protease is completely free of DNase and RNase activity.

When using the QIAamp DNA Blood Mini Kit for a sample that requires a modified protocol, please contact our Technical Service Department for advice about whether your lysis conditions are compatible with QIAGEN Protease. When >8 mM EDTA is used in conjunction with >0.5% SDS,* QIAGEN Protease activity decreases. For samples that require an SDS-containing lysis buffer or that contain high levels of EDTA, the QIAamp DNA Mini Kit is recommended. The QIAamp DNA Mini Kit contains proteinase K, which is the enzyme of choice for SDS-containing lysis buffers used in the Tissue Protocol, but which performs equally well in the Blood and Body Fluid Protocol. The activity of the proteinase K solution is 600 mAU/ml solution (or 40 mAU/mg protein). This activity provides optimal results in QIAamp protocols.

Purification on QIAamp Mini spin columns

The QIAamp DNA purification procedure comprises 4 steps and is carried out using QIAamp Mini spin columns in a standard microcentrifuge, on a vacuum manifold, or fully automated on the QIAcube (see page 15). The procedures are designed to ensure that there is no sample-to-sample cross-contamination and allow safe handling of potentially infectious samples.

* When working with chemicals, always wear a suitable lab coat, disposable gloves, and protective goggles. For more information, consult the appropriate material safety data sheets (MSDSs), available from the product supplier.

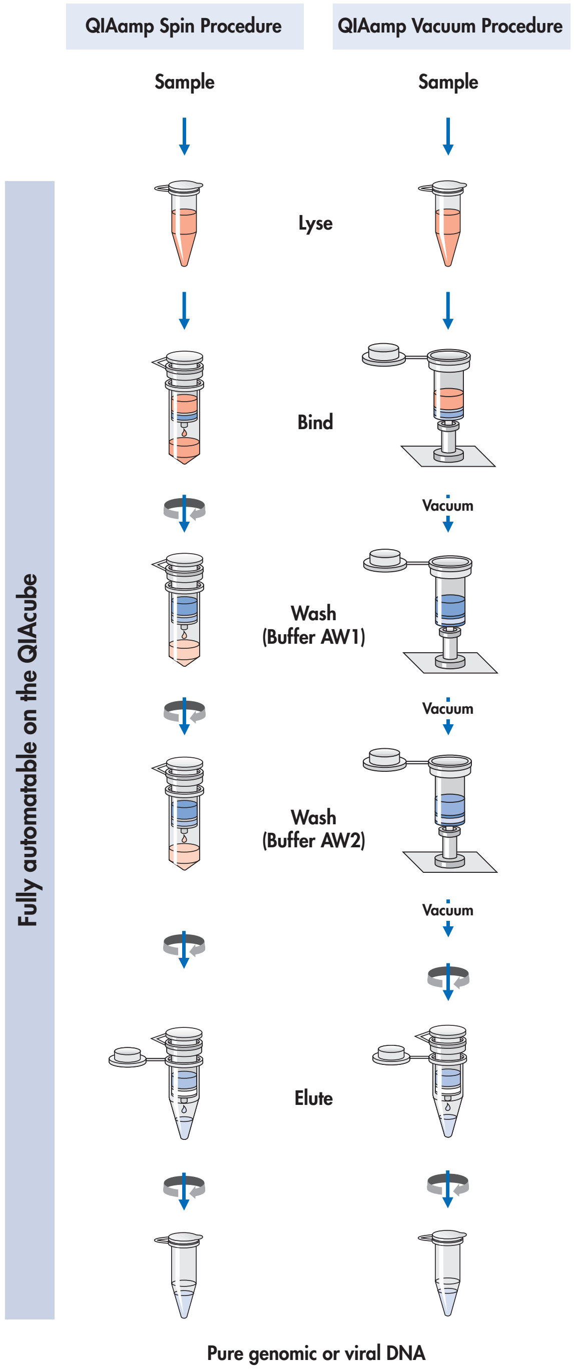
QIAamp Spin Procedure
QIAamp Vacuum Procedure
Sample
Sample
Fully automatable on the QIAcube
Lyse
Bind
Vacuum
Wash
(Buffer AW1)
Vacuum
Wash
(Buffer AW2)
Vacuum
Elute
Pure genomic or viral DNA

QIAamp Mini spin columns fit into most standard microcentrifuge tubes. In the spin protocol, due to the volume of filtrate, 2 ml collection tubes (provided) are required to support the QIAamp Mini spin column during loading and wash steps. For the vacuum protocol, a vacuum manifold (e.g., QIAvac 24 Plus manifold; see Ordering Information, page 68) and a vacuum pump capable of producing a vacuum of –800 to –900 mbar are required. Eluted DNA can be collected in standard 1.5 ml microcentrifuge tubes (not provided).

Adsorption to the QIAamp membrane

The lysate buffering conditions are adjusted to allow optimal binding of the DNA to the QIAamp membrane before the sample is loaded onto the QIAamp Mini spin column. DNA is adsorbed onto the QIAamp silica membrane during a brief centrifugation or vacuum step. Salt and pH conditions in the lysate ensure that protein and other contaminants, which can inhibit PCR and other downstream enzymatic reactions, are not retained on the QIAamp membrane. If the initial sample volume is larger than 200 µl, it will be necessary to load the lysate onto the QIAamp Mini spin column in several steps. If larger sample volumes are required, we suggest using QIAamp DNA Blood Midi or Maxi Kits (Midi: 1–2 ml; Maxi: 5–10 ml starting material).

Removal of residual contaminants

DNA bound to the QIAamp membrane is washed in 2 centrifugation or vacuum steps. The use of 2 different wash buffers, Buffer AW1 and Buffer AW2, significantly improves the purity of the eluted DNA. Wash conditions ensure complete removal of any residual contaminants without affecting DNA binding.

Elution of pure nucleic acids

Purified DNA is eluted from the QIAamp Mini spin column in a concentrated form in either Buffer AE or water. Elution buffer should be equilibrated to room temperature (15–25°C) before it is applied to the column. Yields will be increased if the QIAamp Mini spin column is incubated with the elution buffer at room temperature for 5 minutes before centrifugation. The eluted genomic DNA is up to 50 kb in length (predominantly 20–30 kb) and is suitable for direct use in PCR or Southern-blotting applications.

If the purified DNA is to be stored, elution in Buffer AE (10 mM Tris·Cl; 0.5 mM EDTA; pH 9.0) and storage at –20°C is recommended. If high pH or EDTA affects sensitive downstream applications, use water for elution. However, ensure that the pH of the water is at least 7.0 (deionized water from certain sources can be acidic). DNA stored in water is subject to degradation by acid hydrolysis.

Automated DNA purification on the QIAcube

Purification of DNA from blood, body fluids, tissues, and bacteria using the QIAamp DNA Mini Kit or the QIAamp DNA Blood Mini Kit can be fully automated on the QIAcube. The innovative QIAcube uses advanced technology to process QIAGEN spin columns, enabling seamless integration of automated, low-throughput sample prep into your laboratory workflow. Sample preparation using the QIAcube follows the same steps as the manual procedure (i.e., lyse, bind, wash, and elute), enabling you to continue using the QIAamp DNA Mini Kit and the QIAamp DNA Blood Mini Kit for purification of high-quality DNA. For more information about the automated procedure, see the relevant protocol sheet available at www.qiagen.com/MyQIAcube .

The QIAcube is preinstalled with protocols for purification of plasmid DNA, genomic DNA, RNA, viral nucleic acids, and proteins, plus DNA and RNA cleanup. The range of protocols available is continually expanding, and additional QIAGEN protocols can be downloaded free of charge at www.qiagen.com/MyQIAcube .

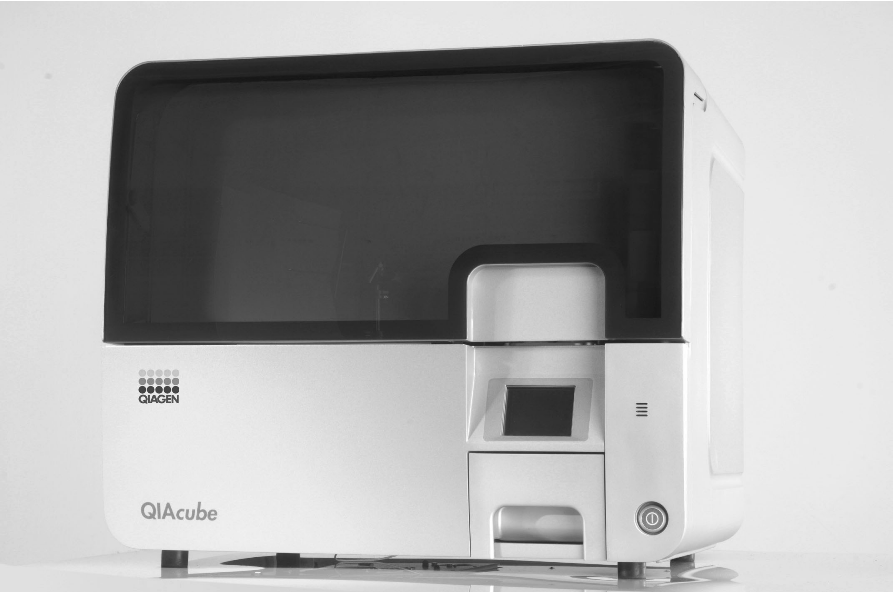

Figure 1. Automated DNA purification. DNA purification using the QIAamp DNA Mini Kit or the QIAamp DNA Blood Mini Kit can be fully automated on the QIAcube.

Equipment and Reagents to Be Supplied by User

When working with chemicals, always wear a suitable lab coat, disposable gloves, and protective goggles. For more information, consult the appropriate material safety data sheets (MSDSs), available from the product supplier.

- Ethanol (96–100%)*
- 1.5 ml microcentrifuge tubes
- Pipet tips with aerosol barrier
- Microcentrifuge (with rotor for 2 ml tubes)
- Vortexer
- Water bath or heating block at 56°C
- Phosphate-buffered saline (PBS) may be required for some samples

For vacuum protocols

- QIAvac 24 Plus (cat. no. 19413) or equivalent
- VacConnectors (cat. no. 19407)
- Vacuum Regulator (cat. 19530) for easy monitoring of vacuum pressures and easy releasing of vacuum
- Vacuum Pump (cat. no. 84010 [USA and Canada], 84000 [Japan], or 84020 [rest of world]) or equivalent pump capable of producing a vacuum of –800 to –900 mbar
- For buccal swabs or large volumes: Extension Tubes (cat. no. 15987)
- Optional: VacValves (cat. no. 19408)
- Optional: QIAvac Connecting System (cat. no. 19419)
- Optional: RNase A (100 mg/ml)

For tissues

- Additional water bath or heating block at 70°C
- Optional: Equipment for mechanical disruption, such as the TissueRuptor® (see page 71 for ordering information) or mortar, pestle, and liquid nitrogen

For buccal swabs

- Additional Buffer AL (cat. no. 19075)
- 2 ml microcentrifuge tubes
- For cotton or DACRON® swabs: Scissors or appropriate cutting device

For dried blood spots

- 3 mm single-hole paper puncher
- Two additional water baths or heating blocks at 85°C and 70°C

* Do not use denatured alcohol, which contains other substances such as methanol or methylethylketone.

Important Notes

Preparation of reagents

QIAGEN Protease stock solution (store at 2–8°C or –20°C)

When using the QIAamp DNA Blood Mini Kit (50), pipet 1.2 ml protease solvent* into the vial containing lyophilized QIAGEN Protease, as indicated on the label. When using the QIAamp DNA Blood Mini Kit (250), pipet 5.5 ml protease solvent into the vial containing lyophilized QIAGEN Protease, as indicated on the label.

Dissolved QIAGEN Protease is stable for up to 2 months when stored at 2–8°C. Storage at –20°C is recommended to prolong the life of QIAGEN Protease, but repeated freezing and thawing should be avoided. For this reason, storage of aliquots of QIAGEN Protease is recommended.

Note: If you also use QIAamp MinElute Virus Kits, be aware that the QIAGEN Protease supplied with these kits is reconstituted in protease resuspension buffer or Buffer AVE and is not compatible with the QIAamp DNA Blood Mini Kit. After reconstituting a vial of QIAGEN Protease, label the resuspended QIAGEN Protease to indicate which buffer was used for resuspension.

Buffer AL† (store at room temperature, 15–25°C)

Mix Buffer AL thoroughly by shaking before use. Buffer AL is stable for 1 year when stored at room temperature.

Note: Do not add QIAGEN Protease or proteinase K directly to Buffer AL.

Buffer AW1† (store at room temperature, 15–25°C)

Buffer AW1 is supplied as a concentrate. Before using for the first time, add the appropriate amount of ethanol (96–100%) as indicated on the bottle.

Buffer AW1 is stable for 1 year when stored closed at room temperature.

Buffer AW2* (store at room temperature, 15–25°C)

Buffer AW2 is supplied as a concentrate. Before using for the first time, add the appropriate amount of ethanol (96–100%) to Buffer AW2 concentrate as indicated on the bottle.

Buffer AW2 is stable for 1 year when stored closed at room temperature.

* Contains sodium azide as a preservative.

† Contains chaotropic salt. See page 8 for safety information.

Carrier DNA

Use carrier DNA (e.g., poly dA, poly dT, poly dA:dT) when the sample is low-copy (i.e., when <10,000 copies are present). For preparation of DNA from free viral particles in fluids or suspensions (other than urine) using the Blood and Body Fluid protocols, we recommend the addition of 1 µl of an aqueous solution containing 5–10 µg of carrier DNA (e.g., poly dA, poly dT, poly dA:dT) to 200 µl Buffer AL. To ensure binding conditions are optimal, increase the volume of ethanol added at step 6 from 200 µl to 230 µl. Elution should be in 60 µl Buffer AE.

Amounts of starting material

Use the amounts of starting material indicated in Table 1.

Table 1. Amounts of starting material for QIAamp Mini procedures

Sample	Amount
Blood, plasma, serum	200 µl
Buffy coat	200 µl
Tissue	25 mg*
Cells (diploid)	5 x 10^6 cells

* When isolating DNA from spleen, 10 mg samples should be used.

Small samples should be adjusted to 200 µl with PBS before loading. For samples larger than 200 µl, the amount of lysis buffer and other reagents added to the sample before loading must be increased proportionally (see note below). Application of the lysed sample to the QIAamp Mini spin column will require more than one loading step if the initial sample volume is increased. The amounts of Buffer AW1 and Buffer AW2 used in the wash steps do not need to be increased.

Scaling up the tissue protocol is possible in principle. The volumes of Buffer ATL and proteinase K used should be increased proportionally, while the volumes of wash and elution buffers should remain constant. The user should determine the maximum amount of tissue used. It is important not to overload the column, as this can lead to significantly lower yields than expected.

Note: All QIAamp buffers can be purchased separately to supplement the QIAamp DNA Mini and QIAamp DNA Blood Mini Kits (see Ordering Information, page 70). QIAGEN Proteinase K is recommended for use with tissue samples. QIAGEN Protease is suitable for genomic DNA preparation from blood, cells, and body fluids.

Preparation of buffy coat

Buffy coat is a leukocyte-enriched fraction of whole blood. Preparing a buffy-coat fraction from whole blood is simple and yields approximately 5–10 times more DNA than an equivalent volume of whole blood.

Prepare buffy coat by centrifuging whole blood at 2500 x *g* for 10 minutes at room temperature. After centrifugation, 3 different fractions are distinguishable: the upper clear layer is plasma; the intermediate layer is buffy coat, containing concentrated leukocytes; and the bottom layer contains concentrated erythrocytes.

Handling of QIAamp Mini columns

Owing to the sensitivity of nucleic acid amplification technologies, the following precautions are necessary when handling QIAamp Mini columns to avoid cross-contamination between sample preparations:

- Carefully apply the sample or solution to the QIAamp Mini column. Pipet the sample into the QIAamp Mini column without wetting the rim of the column.
- Change pipet tips between all liquid transfers. The use of aerosol-barrier pipet tips is recommended.
- Avoid touching the QIAamp membrane with the pipet tip.
- After all pulse-vortexing steps, briefly centrifuge the 1.5 ml microcentrifuge tubes to remove drops from the inside of the lid.
- Wear gloves throughout the entire procedure. In case of contact between gloves and sample, change gloves immediately.

Centrifugation

QIAamp Mini columns will fit into most standard 1.5–2 ml microcentrifuge tubes. Additional 2 ml collection tubes are available separately.

Centrifugation of QIAamp Mini columns is performed at 6000 x *g* (8000 rpm) in order to reduce centrifuge noise. Centrifuging QIAamp Mini columns at full speed will not affect DNA yield. Centrifugation at lower speeds is also acceptable, provided that nearly all of each solution is transferred through the QIAamp membrane. When preparing DNA from buffy coat or lymphocytes, full-speed centrifugation is recommended to avoid clogging.

All centrifugation steps should be carried out at room temperature (15–25°C).

Processing QIAamp Mini columns using a microcentrifuge (spin protocols)

Close the QIAamp Mini column before placing it in the microcentrifuge. Centrifuge as described.

- Remove the QIAamp Mini column and collection tube from the microcentrifuge. Place the QIAamp Mini column in a new collection tube. Discard the filtrate and the collection tube. Note that the filtrate may contain hazardous waste and should be disposed of appropriately.
- Open only one QIAamp Mini column at a time, and take care to avoid generating aerosols.
- For efficient parallel processing of multiple samples, fill a rack with collection tubes to which the QIAamp Mini columns can be transferred after centrifugation. Used collection tubes containing the filtrate can be discarded, and the new collection tubes containing the QIAamp Mini columns can be placed directly in the microcentrifuge.

The QIAvac 24 Plus

The QIAvac 24 Plus is designed for fast and efficient vacuum processing of up to 24 QIAGEN spin columns in parallel. Samples and wash solutions are drawn through the column membranes by vacuum instead of centrifugation, providing greater speed and reduced hands-on time in purification procedures.

In combination with the QIAvac Connecting System (optional), the QIAvac 24 Plus can be used as a flow-through system. The sample flow-through is collected in a separate waste bottle.

For maintenance of the QIAvac 24 Plus, please refer to the handling guidelines in the *QIAvac 24 Plus Handbook.*

Processing QIAamp Mini columns on the QIAvac 24 Plus (vacuum protocols)

QIAamp Mini spin columns are processed on the QIAvac 24 Plus using disposable VacConnectors and reusable VacValves. VacValves (optional) are inserted directly into the luer slots of the QIAvac 24 Plus manifold and ensure a steady flow rate, facilitating parallel processing of samples of different natures (e.g., blood and body fluids), volumes, or viscosities. They should be used if sample flow rates differ significantly in order to ensure consistent vacuum. VacConnectors are disposable connectors that fit between QIAamp Mini columns and VacValves or between the QIAamp Mini columns and the luer slots of the QIAvac 24 Plus. They prevent direct contact between the spin column and VacValve during purification, avoiding any cross-contamination between samples. VacConnectors are discarded after a single use.

Handling guidelines for the QIAvac 24 Plus

- Always place the QIAvac 24 Plus on a secure bench top or work area. If dropped, the QIAvac 24 Plus manifold may crack.
- Always store the QIAvac 24 Plus clean and dry. For cleaning procedures see the *QIAvac 24 Plus Handbook.*
- The components of the QIAvac 24 Plus are not resistant to certain solvents (Table 2). If these solvents are spilled on the unit, rinse it thoroughly with water.
- To ensure consistent performance, do not apply silicone or vacuum grease to any part of the QIAvac 24 Plus manifold.
- Always use caution and wear safety glasses when working near a vacuum manifold under pressure.
- Contact QIAGEN Technical Services or your local distributor for information concerning spare or replacement parts.
- The vacuum pressure is the pressure differential between the inside of the vacuum manifold and the atmosphere (standard atmospheric pressure 1013 millibar or 760 mm Hg) and can be measured using the QIAvac Connecting System or a vacuum regulator (see Figure 2). The vacuum protocol requires a vacuum pump capable of producing a vacuum of –800 to –900 mbar (e.g., QIAGEN, Vacuum Pump). Higher vacuum pressures must be avoided. Use of vacuum pressures lower than recommended may reduce DNA yield and purity and increase the frequency of clogged membranes.

Table 2. Chemical resistance properties of QIAvac 24 Plus

**Resistant to:**		
Acetic acid	Chaotropic salts	Chlorine bleach
Chromic acid	Concentrated alcohols	Hydrochloric acid
SDS	Sodium chloride	Sodium hydroxide
Tween® 20	Urea	
**Not resistant to:**		
Benzene	Chloroform	Ethers
Phenol	Toluene	

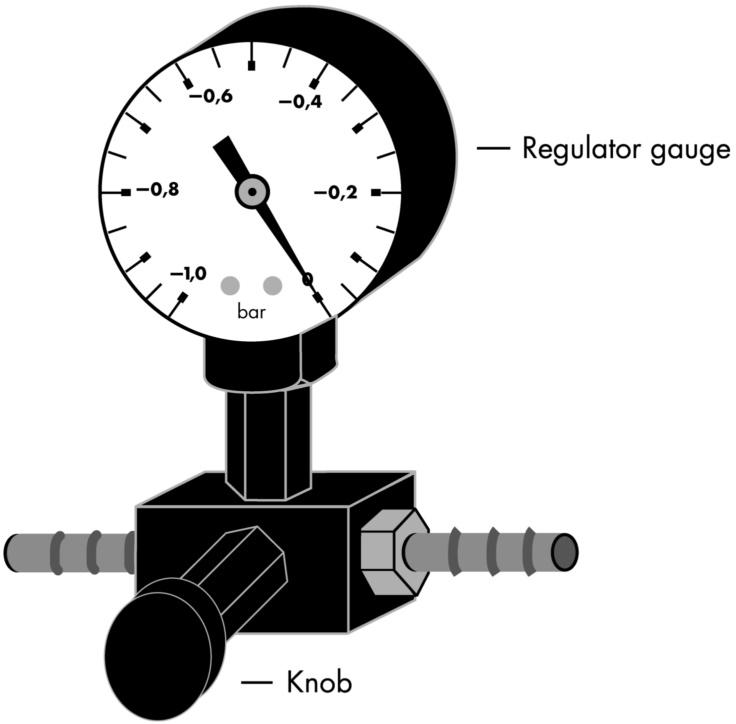

Figure 2. Schematic diagram of the Vacuum Regulator.

Setup of the QIAvac 24 Plus vacuum manifold

1. **Connect the QIAvac 24 Plus to a vacuum source. If using the QIAvac Connecting System, connect the system to the manifold and vacuum source as described in Appendix A of the *QIAvac 24 Plus Handbook.***
2. **Insert a VacValve into each luer slot of the QIAvac 24 Plus that is to be used (see Figure 3). Close unused luer slots with luer plugs or close the inserted VacValve.**

   VacValves should be used if flow rates of samples differ significantly to ensure consistent vacuum.
3. **Insert a VacConnector into each VacValve (see Figure 3).**

   Perform this step directly before starting the purification to avoid exposure of VacConnectors to potential contaminants in the air.
4. **Place the QIAamp Mini columns into the VacConnectors on the manifold (see Figure 3).**
5. **If necessary, insert an Extension Tube into each QIAamp Mini column (see Figure 4).**

   Extension Tubes are required for processing buccal swabs or large volumes.

6. **For nucleic acid purification, follow the instructions in the vacuum protocols. Discard the VacConnectors appropriately after use.**

   Leave the lid of the QIAamp Mini column open while applying vacuum.

   Switch off the vacuum between steps to ensure that a consistent, even vacuum is applied during processing. For faster vacuum release, a vacuum regulator should be used (see Figure 2).

   Note: Each VacValve can be closed individually when the sample is completely drawn through the spin column, allowing parallel processing of samples of different volumes or viscosities.

7. **After processing samples, clean the QIAvac 24 Plus (see "Cleaning and Decontaminating the QIAvac 24 Plus" in the *QIAvac 24 Plus Handbook*).**

   Note: Buffers AL and AW1 used in QIAamp DNA Mini and QIAamp DNA Blood Mini procedures are not compatible with disinfecting agents containing bleach. See page 8 for safety information.

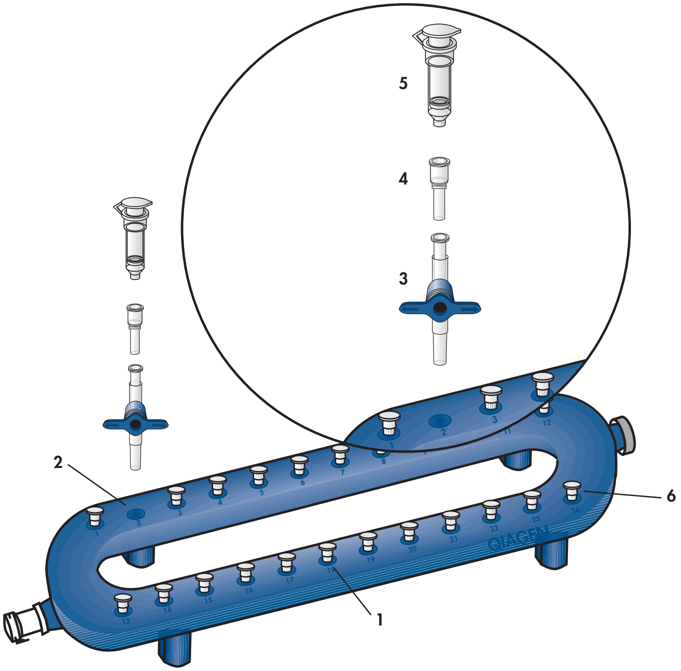

Figure 3. Setting up the QIAvac 24 Plus with QIAamp Mini columns using VacValves and VacConnectors.

1. QIAvac 24 Plus vacuum manifold
2. Luer slot of the QIAvac 24 Plus
3. VacValve*
4. VacConnector*
5. QIAamp Mini column
6. Luer slot closed with luer plug

* Must be purchased separately.

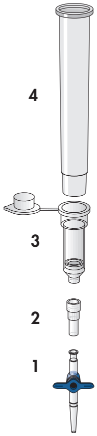

Figure 4. Assembly of QIAamp Mini columns with extension tubes (for buccal swabs or large volumes).

1. VacValve*
2. VacConnector*
3. QIAamp Mini column
4. Extension tube*

* Must be purchased separately.

Processing QIAamp Mini columns on the QIAcube

Sample preparation using the QIAcube follows the same steps as the manual procedure (i.e., lyse, bind, wash, and elute). For more information about the automated procedure, see the relevant protocol sheet available at www.qiagen.com/MyQIAcube .

Copurification of RNA

QIAamp Mini spin columns copurify DNA and RNA when both are present in the sample (see Table 3). RNA may inhibit some downstream enzymatic reactions but will not inhibit PCR. If RNA-free genomic DNA is required, 4 µl of an RNase A stock solution (100 mg/ml) should be added to the sample prior to the addition of Buffer AL. RNase A is not supplied with the kits and should be purchased separately. Ensure that the RNase A used is completely free of DNase activity.

Table 3. Yields of nucleic acids purified from various sources with QIAamp Kits

Sample	Nucleic acid yield (µg) without RNase A treatment	DNA yield (µg) with RNase A treatment
Blood (200 µl)	4–12	4–12
Buffy coat (200 µl)	25–50	25–50
Cultured cells (5 x 10^6)	20–30	15–20
Liver (25 mg)	60–115	10–30
Brain (25 mg)	35–60	15–30
Lung (25 mg)	8–20	5–10
Heart (25 mg)	25–45	5–10
Kidney (25 mg)	40–85	15–30
Spleen (10 mg)	25–45	5–30

DNA was purified with QIAamp Kits following standard protocols.

Elution mode for maximum yield or concentration

The yield of genomic DNA depends on the sample type and the number of cells in the sample. Typically, a 200 µl sample of whole blood from a healthy individual will yield 3–12 µg of DNA. (If higher yields are required, use QIAamp DNA Blood Midi or QIAamp DNA Blood Maxi Kits with up to 2 ml or up to 10 ml blood, respectively.) For most whole blood samples, a single elution with 200 µl elution buffer is sufficient. Samples with elevated white blood cell (WBC) counts, ranging from 1 x 10^7 to 1.5 x 10^7 cells/ml, will yield between 13 and 20 µg of DNA. If such a sample is loaded onto the column, approximately 80% of the DNA will elute in the first 200 µl, and up to 20% more in the next 200 µl. In samples with WBC counts exceeding 1.5 x 10^7 cells/ml, up to 60% of the DNA will elute in the first 200 µl and up to 70% of the remaining material in each subsequent 200 µl (see Table 4). Elution into a fresh tube is recommended to prevent dilution of the first eluate. More than 200 µl should not be eluted into a 1.5 ml microcentrifuge tube because the spin column will contact the eluate, leading to possible aerosol formation of samples during centrifugation. Eluting in 4 x 100 µl instead of 2 x 200 µl does not increase elution efficiency. In all cases, a single elution with 200 µl of elution buffer will provide sufficient DNA to perform multiple amplification reactions.

For some downstream applications, concentrated DNA may be required. Elution with volumes of less than 200 µl increases the final DNA concentration in the eluate significantly but slightly reduces overall DNA yield (see Table 5). For samples containing <3 µg of DNA, eluting the DNA in 100 µl is recommended. For samples containing less than 1 µg of DNA, only one elution in 50 µl Buffer AE or water is recommended.

Table 4. Total nucleic acid yields with QIAamp Kits using successive elutions

		Yield (µg)			
**Sample**	**Amount**	**Elution 1**	**Elution 2**	**Elution 3**	**Total**
Whole blood	200 µl	3–8	1–2	0–2	4–12
Lymphocytes	5 x 10^6 cells/200 µl	13–18	5–8	3–5	20–30
Buffy coat	200 µl	15–25	8–15	5–10	28–50
Liver*	25 mg	25–45	25–45	10–25	60–115
Brain*	25 mg	20–30	10–20	5–10	35–60
Lung*	25 mg	5–10	2–6	1–4	8–20
Heart*	25 mg	15–25	8–15	2–5	25–45
Kidney*	25 mg	25–40	20–30	5–15	50–85
Spleen*	10 mg	15–25	8–15	2–5	25–45

DNA was purified with QIAamp Kits following standard protocols. Successive elutions were each performed with 200 µl Buffer AE.

* Results refer to the QIAamp DNA Mini Kit only.

Table 5. Effect of elution volume on yield and concentration

Elution volume	Yield (µg)	Yield (%)	DNA concentration (ng/µl)
200	6.80	100	34.0
150	6.51	95	43.4
100	6.25	92	62.5
50	5.84	86	116.8

DNA was purified with QIAamp Kits following standard protocols. Average values obtained from 20 preparations are shown.

Protocol: DNA Purification from Blood or Body Fluids (Spin Protocol)

This protocol is for purification of total (genomic, mitochondrial, and viral) DNA from whole blood, plasma, serum, buffy coat, lymphocytes, and body fluids using a microcentrifuge. For total DNA purification using a vacuum manifold, see "Protocol: DNA Purification from Blood or Body Fluids (Vacuum Protocol)" on page 30.

Important points before starting

- All centrifugation steps are carried out at room temperature (15–25°C).
- Use carrier DNA if the sample contains <10,000 genome equivalents (see page 18).
- 200 µl of whole blood yields 3–12 µg of DNA. Preparation of buffy coat (see page 19) is recommended if a higher yield is required.

Things to do before starting

- Equilibrate samples to room temperature.
- Heat a water bath or heating block to 56°C for use in step 4.
- Equilibrate Buffer AE or distilled water to room temperature for elution in step 11.
- Ensure that Buffer AW1, Buffer AW2, and QIAGEN Protease have been prepared according to the instructions on page 17.
- If a precipitate has formed in Buffer AL, dissolve by incubating at 56°C.

Procedure

1. **Pipet 20 µl QIAGEN Protease (or proteinase K) into the bottom of a 1.5 ml microcentrifuge tube.**
2. **Add 200 µl sample to the microcentrifuge tube. Use up to 200 µl whole blood, plasma, serum, buffy coat, or body fluids, or up to 5 x 10^6 lymphocytes in 200 µl PBS.**

   If the sample volume is less than 200 µl, add the appropriate volume of PBS.

   QIAamp Mini spin columns copurify RNA and DNA when both are present in the sample. RNA may inhibit some downstream enzymatic reactions, but not PCR. If RNA-free genomic DNA is required, 4 µl of an RNase A stock solution (100 mg/ml) should be added to the sample before addition of Buffer AL.

   Note: It is possible to add QIAGEN Protease (or proteinase K) to samples that have already been dispensed into microcentrifuge tubes. In this case, it is important to ensure proper mixing after adding the enzyme.

3. **Add 200 µl Buffer AL to the sample. Mix by pulse-vortexing for 15 s.**

   In order to ensure efficient lysis, it is essential that the sample and Buffer AL are mixed thoroughly to yield a homogeneous solution.

   If the sample volume is larger than 200 µl, increase the amount of QIAGEN Protease (or proteinase K) and Buffer AL proportionally; for example, a 400 µl sample will require 40 µl QIAGEN Protease (or proteinase K) and 400 µl Buffer AL. If sample volumes larger than 400 µl are required, use of QIAamp DNA Blood Midi or Maxi Kits is recommended; these can process up to 2 ml or up to 10 ml of sample, respectively.

   Note: Do not add QIAGEN Protease or proteinase K directly to Buffer AL.

4. **Incubate at 56°C for 10 min.**

   DNA yield reaches a maximum after lysis for 10 min at 56°C. Longer incubation times have no effect on yield or quality of the purified DNA.

5. **Briefly centrifuge the 1.5 ml microcentrifuge tube to remove drops from the inside of the lid.**

6. **Add 200 µl ethanol (96–100%) to the sample, and mix again by pulse-vortexing for 15 s. After mixing, briefly centrifuge the 1.5 ml microcentrifuge tube to remove drops from the inside of the lid.**

   If the sample volume is greater than 200 µl, increase the amount of ethanol proportionally; for example, a 400 µl sample will require 400 µl of ethanol.

7. **Carefully apply the mixture from step 6 to the QIAamp Mini spin column (in a 2 ml collection tube) without wetting the rim. Close the cap, and centrifuge at 6000 x *g* (8000 rpm) for 1 min. Place the QIAamp Mini spin column in a clean 2 ml collection tube (provided), and discard the tube containing the filtrate.***

   Close each spin column in order to avoid aerosol formation during centrifugation.

   Centrifugation is performed at 6000 x *g* (8000 rpm) in order to reduce noise. Centrifugation at full speed will not affect the yield or purity of the DNA. If the lysate has not completely passed through the column after centrifugation, centrifuge again at higher speed until the QIAamp Mini spin column is empty.

   Note: When preparing DNA from buffy coat or lymphocytes, centrifugation at full speed is recommended to avoid clogging.

8. **Carefully open the QIAamp Mini spin column and add 500 µl Buffer AW1 without wetting the rim. Close the cap and centrifuge at 6000 x *g* (8000 rpm) for 1 min. Place the QIAamp Mini spin column in a clean 2 ml collection tube (provided), and discard the collection tube containing the filtrate.***

   It is not necessary to increase the volume of Buffer AW1 if the original sample volume is larger than 200 µl.

* Flow-through contains Buffer AL or Buffer AW1 and is therefore not compatible with bleach. See page 8 for safety information.

9. **Carefully open the QIAamp Mini spin column and add 500 µl Buffer AW2 without wetting the rim. Close the cap and centrifuge at full speed (20,000 x *g*; 14,000 rpm) for 3 min.**

10. **Recommended: Place the QIAamp Mini spin column in a new 2 ml collection tube (not provided) and discard the old collection tube with the filtrate. Centrifuge at full speed for 1 min.**

    This step helps to eliminate the chance of possible Buffer AW2 carryover.

11. **Place the QIAamp Mini spin column in a clean 1.5 ml microcentrifuge tube (not provided), and discard the collection tube containing the filtrate. Carefully open the QIAamp Mini spin column and add 200 µl Buffer AE or distilled water. Incubate at room temperature (15–25°C) for 1 min, and then centrifuge at 6000 x *g* (8000 rpm) for 1 min.**

    Incubating the QIAamp Mini spin column loaded with Buffer AE or water for 5 min at room temperature before centrifugation generally increases DNA yield.

    A second elution step with a further 200 µl Buffer AE will increase yields by up to 15%.

    Volumes of more than 200 µl should not be eluted into a 1.5 ml microcentrifuge tube because the spin column will come into contact with the eluate, leading to possible aerosol formation during centrifugation.

    Elution with volumes of less than 200 µl increases the final DNA concentration in the eluate significantly, but slightly reduces the overall DNA yield (see Table 5, page 26). For samples containing less than 1 µg of DNA, elution in 50 µl Buffer AE or water is recommended. Eluting with 2 x 100 µl instead of 1 x 200 µl does not increase elution efficiency.

    For long-term storage of DNA, eluting in Buffer AE and storing at –20°C is recommended, since DNA stored in water is subject to acid hydrolysis.

    A 200 µl sample of whole human blood (approximately 5 x 10^6 leukocytes/ml) typically yields 6 µg of DNA in 200 µl water (30 ng/µl) with an A_{260}/A_{280} ratio of 1.7–1.9.

    For more information about elution and how to determine DNA yield, purity, and length, refer to pages 25–26 and Appendix A, page 51.

Protocol: DNA Purification from Blood or Body Fluids (Vacuum Protocol)

This protocol is for purification of total (genomic, mitochondrial, and viral) DNA from whole blood, plasma, serum, lymphocytes, and body fluids using the QIAvac 24 Plus or equivalent vacuum manifold. For total DNA purification using a microcentrifuge, see "Protocol: DNA Purification from Blood or Body Fluids (Spin Protocol)" on page 27.

Important points before starting

- All centrifugation steps are carried out at room temperature (15–25°C).
- For setup of the QIAvac 24 Plus, see pages 20–24.
- Switch off vacuum between steps to ensure that a consistent, even vacuum is applied during protocol steps.
- Use carrier DNA if the sample contains <10,000 genome equivalents (see page 18).
- 200 µl of whole blood yields 3–12 µg of DNA.

Things to do before starting

- Equilibrate samples to room temperature.
- Heat a water bath or heating block to 56°C for use in step 4.
- Equilibrate Buffer AE or distilled water to room temperature for elution in step 11.
- Ensure that Buffer AW1, Buffer AW2, and QIAGEN Protease have been prepared according to the instructions on page 17.
- If a precipitate has formed in Buffer AL, dissolve by incubating at 56°C.

Procedure

1. **Pipet 20 µl QIAGEN Protease (or proteinase K) into the bottom of a 1.5 ml microcentrifuge tube.**
2. **Add 200 µl sample to the microcentrifuge tube. Use up to 200 µl whole blood, plasma, serum, or body fluids, or up to 5 x 10^6 lymphocytes in 200 µl PBS.**

   If the sample volume is less than 200 µl, add the appropriate volume of PBS.

   QIAamp Mini columns copurify RNA and DNA when both are present in the sample. RNA may inhibit some downstream enzymatic reactions, but not PCR. If RNA-free genomic DNA is required, 4 µl of an RNase A stock solution (100 mg/ml) should be added to the sample before addition of Buffer AL.

   Note: It is possible to add QIAGEN Protease (or proteinase K) to samples that have already been dispensed into microcentrifuge tubes. In this case, it is important to ensure proper mixing after adding the enzyme.

3. **Add 200 µl Buffer AL to the sample. Mix by pulse-vortexing for 15 s.**

   In order to ensure efficient lysis, it is essential that the sample and Buffer AL are mixed thoroughly to yield a homogeneous solution.

   If the sample volume is larger than 200 µl, increase the amount of QIAGEN Protease (or proteinase K) and Buffer AL proportionally; for example, a 400 µl sample will require 40 µl QIAGEN Protease (or proteinase K) and 400 µl Buffer AL. If sample volumes larger than 400 µl are required, use of QIAamp DNA Blood Midi or Maxi Kits is recommended; these can process up to 2 ml or up to 10 ml of sample, respectively.

   Note: Do not add QIAGEN Protease or proteinase K directly to Buffer AL.

4. **Incubate at 56°C for 10 min.**

   DNA yield reaches a maximum after lysis for 10 min at 56°C. Longer incubation times have no effect on yield or quality of the purified DNA.

5. **Briefly centrifuge the 1.5 ml microcentrifuge tube to remove drops from the inside of the lid.**

6. **Add 200 µl ethanol (96–100%) to the sample, and mix again by pulse-vortexing for 15 s. After mixing, briefly centrifuge the 1.5 ml microcentrifuge tube to remove drops from the inside of the lid.**

   If the sample volume is greater than 200 µl, increase the amount of ethanol proportionally; for example, a 400 µl sample will require 400 µl of ethanol.

7. **Insert the QIAamp Mini column into the VacConnector on the QIAvac vacuum manifold. Carefully apply the mixture from step 6 to the QIAamp Mini column without wetting the rim. Switch on the vacuum pump. Be sure to leave the lid of the QIAamp Mini column open while applying vacuum. After all lysates have been drawn through the spin column, switch off the vacuum pump.**

   The collection tube from the blister pack can be saved for the centrifugation in step 10.

   If at this stage all of the solution has not passed through the membrane, place the QIAamp Mini column into a clean 2 ml collection tube (provided), close the cap, and centrifuge at 6000 x *g* (8000 rpm) for 3 min or until all the liquid has completely passed through. Place the QIAamp Mini column into another clean 2 ml collection tube, and discard the tube containing the filtrate.* Continue with step 8 of the spin protocol on page 28.

8. **Apply 750 µl Buffer AW1 to the QIAamp Mini column without wetting the rim. Leave the lid of the QIAamp Mini column open and switch on the vacuum pump. After all of Buffer AW1 has been drawn through the QIAamp Mini column, switch off the vacuum pump.**

* Flow-through contains Buffer AL or Buffer AW1 and is therefore not compatible with bleach. See page 8 for safety information.

9. **Add 750 µl Buffer AW2 without wetting the rim of the QIAamp Mini column. Leave the lid of the QIAamp Mini column open and switch on the vacuum pump. After all of Buffer AW2 has been drawn through the QIAamp Mini column, switch off the vacuum pump.**
10. **Close the lid of the QIAamp Mini column, remove it from the vacuum manifold, and discard the VacConnector. Place the QIAamp Mini column into a clean 2 ml collection tube and centrifuge at 20,000 x *g* (14,000 rpm) for 1 min to dry the membrane completely.**
11. **Place the QIAamp Mini column in a clean 1.5 ml microcentrifuge tube (not provided). Discard the collection tube containing the filtrate. Carefully open the QIAamp Mini column. Add 200 µl Buffer AE or distilled water equilibrated to room temperature. Incubate at room temperature for 1 min, and then centrifuge at 6000 x *g* (8000 rpm) for 1 min.**

    Incubating the QIAamp Mini column loaded with Buffer AE or water for 5 min at room temperature before centrifugation generally increases DNA yield.

    A second elution step with a further 200 µl Buffer AE will increase yields by up to 15%.

    Volumes of more than 200 µl should not be eluted into a 1.5 ml microcentrifuge tube because the QIAamp Mini column will come into contact with the eluate, leading to possible aerosol formation during centrifugation.

    Elution with volumes of less than 200 µl increases the final DNA concentration in the eluate significantly, but slightly reduces overall DNA yield (see Table 5, page 26). For samples containing less than 1 µg of DNA, elution in 50 µl Buffer AE or water is recommended. Eluting with 2 x 100 µl instead of 1 x 200 µl does not increase elution efficiency.

    For long-term storage of DNA, eluting in Buffer AE and storing at –20°C is recommended, since DNA stored in water is subject to acid hydrolysis.

    A 200 µl sample of whole human blood (approximately 5 x 10^6 leukocytes/ml) typically yields 6 µg of DNA in 200 µl water (30 ng/µl) with an A_{260}/A_{280} ratio of 1.7–1.9.

    For more information about elution and how to determine DNA yield, purity, and length, refer to pages 25–26 and Appendix A, page 51.

Protocol: DNA Purification from Tissues (QIAamp DNA Mini Kit)

This protocol is for purification of total (genomic, mitochondrial, and viral) DNA from tissues using the QIAamp DNA Mini Kit.

Important points before starting

- All centrifugation steps are carried out at room temperature (15–25°C).
- Use carrier DNA if the sample contains <10,000 genome equivalents (see page 18).
- Avoid repeated freezing and thawing of stored samples, since this leads to reduced DNA size.
- Transcriptionally active tissues, such as liver and kidney, contain high levels of RNA which will copurify with genomic DNA. RNA may inhibit some downstream enzymatic reactions, but will not inhibit PCR. If RNA-free genomic DNA is required, include the RNase A digest, as described in step 5a of the protocol.

Things to do before starting

- Equilibrate the sample to room temperature (15–25°C).
- Heat 2 water baths or heating blocks: one to 56°C for use in step 3, and one to 70°C for use in step 5.
- Equilibrate Buffer AE or distilled water to room temperature for elution in step 11.
- Ensure that Buffers AW1 and AW2 have been prepared according to the instructions on page 17.
- If a precipitate has formed in Buffer ATL or Buffer AL, dissolve by incubating at 56°C.

Procedure

1. **Excise the tissue sample or remove it from storage. Determine the amount of tissue. Do not use more than 25 mg (10 mg spleen).**

   Weighing tissue is the most accurate way to determine the amount.

   If DNA is prepared from spleen tissue, no more than 10 mg should be used.

   The yield of DNA will depend on both the amount and the type of tissue processed.

   1 mg of tissue will yield approximately 0.2–1.2 µg of DNA.

2. Cut up (step 2a), grind (step 2b), or mechanically disrupt (step 2c) the tissue sample.

The QIAamp procedure requires no mechanical disruption of the tissue sample, but lysis time will be reduced if the sample is ground in liquid nitrogen (step 2b) or mechanically homogenized (step 2c) in advance.

2a. Cut up to 25 mg of tissue (up to 10 mg spleen) into small pieces. Place in a 1.5 ml microcentrifuge tube, and add 180 µl of Buffer ATL. Proceed with step 3.

It is important to cut the tissue into small pieces to decrease lysis time.

2 ml microcentrifuge tubes may be better suited for lysis.

2b. Place up to 25 mg of tissue (10 mg spleen) in liquid nitrogen, and grind thoroughly with a mortar and pestle. Decant tissue powder and liquid nitrogen into 1.5 ml microcentrifuge tube. Allow the liquid nitrogen to evaporate, but do not allow the tissue to thaw, and add 180 µl of Buffer ATL. Proceed with step 3.

2c. Add up to 25 mg of tissue (10 mg spleen) to a 1.5 ml microcentrifuge tube containing no more than 80 µl PBS. Homogenize the sample using the TissueRuptor or equivalent rotor–stator homogenizer. Add 100 µl Buffer ATL, and proceed with step 3.

Some tissues require undiluted Buffer ATL for complete lysis. In this case, grinding in liquid nitrogen is recommended. Samples cannot be homogenized directly in Buffer ATL, which contains detergent.

3. Add 20 µl proteinase K, mix by vortexing, and incubate at 56°C until the tissue is completely lysed. Vortex occasionally during incubation to disperse the sample, or place in a shaking water bath or on a rocking platform.

Note: Proteinase K must be used. QIAGEN Protease has reduced activity in the presence of Buffer ATL.

Lysis time varies depending on the type of tissue processed. Lysis is usually complete in 1–3 h. Lysis overnight is possible and does not influence the preparation. In order to ensure efficient lysis, a shaking water bath or a rocking platform should be used. If not available, vortexing 2–3 times per hour during incubation is recommended.

4. Briefly centrifuge the 1.5 ml microcentrifuge tube to remove drops from the inside of the lid.

5. If RNA-free genomic DNA is required, follow step 5a. Otherwise, follow step 5b.

Transcriptionally active tissues, such as liver and kidney, contain high levels of RNA which will copurify with genomic DNA. RNA may inhibit some downstream enzymatic reactions, but will not inhibit PCR.

5a. First add 4 µl RNase A (100 mg/ml), mix by pulse-vortexing for 15 s, and incubate for 2 min at room temperature. Briefly centrifuge the 1.5 ml microcentrifuge tube to remove drops from inside the lid before adding 200 µl Buffer AL to the sample. Mix again by pulse-vortexing for 15 s, and incubate at 70°C for 10 min. Briefly centrifuge the 1.5 ml microcentrifuge tube to remove drops from inside the lid.

It is essential that the sample and Buffer AL are mixed thoroughly to yield a homogeneous solution.

A white precipitate may form on addition of Buffer AL. In most cases it will dissolve during incubation at 70°C. The precipitate does not interfere with the QIAamp procedure or with any subsequent application.

5b. Add 200 µl Buffer AL to the sample, mix by pulse-vortexing for 15 s, and incubate at 70°C for 10 min. Briefly centrifuge the 1.5 ml microcentrifuge tube to remove drops from inside the lid.

It is essential that the sample and Buffer AL are mixed thoroughly to yield a homogeneous solution.

A white precipitate may form on addition of Buffer AL, which in most cases will dissolve during incubation at 70°C. The precipitate does not interfere with the QIAamp procedure or with any subsequent application.

6. Add 200 µl ethanol (96–100%) to the sample, and mix by pulse-vortexing for 15 s. After mixing, briefly centrifuge the 1.5 ml microcentrifuge tube to remove drops from inside the lid.

It is essential that the sample, Buffer AL, and the ethanol are mixed thoroughly to yield a homogeneous solution.

A white precipitate may form on addition of ethanol. It is essential to apply all of the precipitate to the QIAamp Mini spin column. This precipitate does not interfere with the QIAamp procedure or with any subsequent application.

Do not use alcohols other than ethanol since this may result in reduced yields.

7. Carefully apply the mixture from step 6 (including the precipitate) to the QIAamp Mini spin column (in a 2 ml collection tube) without wetting the rim. Close the cap, and centrifuge at 6000 x *g* (8000 rpm) for 1 min. Place the QIAamp Mini spin column in a clean 2 ml collection tube (provided), and discard the tube containing the filtrate.*

Close each spin column to avoid aerosol formation during centrifugation.

It is essential to apply all of the precipitate to the QIAamp Mini spin column.

Centrifugation is performed at 6000 x *g* (8000 rpm) in order to reduce noise. Centrifugation at full speed will not affect the yield or purity of the DNA. If the solution has not completely passed through the membrane, centrifuge again at a higher speed until all the solution has passed through.

* Flow-through contains Buffer AL or Buffer AW1 and is therefore not compatible with bleach. See page 8 for safety information.

Tissues

8. **Carefully open the QIAamp Mini spin column and add 500 µl Buffer AW1 without wetting the rim. Close the cap, and centrifuge at 6000 x *g* (8000 rpm) for 1 min. Place the QIAamp Mini spin column in a clean 2 ml collection tube (provided), and discard the collection tube containing the filtrate.***

9. **Carefully open the QIAamp Mini spin column and add 500 µl Buffer AW2 without wetting the rim. Close the cap and centrifuge at full speed (20,000 x *g*; 14,000 rpm) for 3 min.**

10. **Recommended: Place the QIAamp Mini spin column in a new 2 ml collection tube (not provided) and discard the old collection tube with the filtrate. Centrifuge at full speed for 1 min.**

    This step helps to eliminate the chance of possible Buffer AW2 carryover.

11. **Place the QIAamp Mini spin column in a clean 1.5 ml microcentrifuge tube (not provided), and discard the collection tube containing the filtrate. Carefully open the QIAamp Mini spin column and add 200 µl Buffer AE or distilled water. Incubate at room temperature for 1 min, and then centrifuge at 6000 x *g* (8000 rpm) for 1 min.**

12. **Repeat step 11.**

    A 5 min incubation of the QIAamp Mini spin column loaded with Buffer AE or water, before centrifugation, generally increases DNA yield.

    A third elution step with a further 200 µl Buffer AE will increase yields by up to 15%.

    Volumes of more than 200 µl should not be eluted into a 1.5 ml microcentrifuge tube because the spin column will come into contact with the eluate, leading to possible aerosol formation during centrifugation.

    Elution with volumes of less than 200 µl increases the final DNA concentration in the eluate significantly, but slightly reduces the overall DNA yield (see Table 5, page 26). Eluting with 4 x 100 µl instead of 2 x 200 µl does not increase elution efficiency.

    For long-term storage of DNA, eluting in Buffer AE and placing at –20°C is recommended, since DNA stored in water is subject to acid hydrolysis.

    Yields of DNA will depend both on the amount and the type of tissue processed. 25 mg of tissue will yield approximately 10–30 µg of DNA in 400 µl of water (25–75 ng/µl), with an A_{260}/A_{280} ratio of 1.7–1.9.

    For more information about elution and how to determine DNA yield, length, and purity, refer to pages 25–26 and Appendix A, page 51.

* Flow-through contains Buffer AL or Buffer AW1 and is therefore not compatible with bleach. See page 8 for safety information.

Protocol: DNA Purification from Buccal Swabs (Spin Protocol)

This protocol is for purification of total (genomic, mitochondrial, and viral) DNA from buccal swabs using a microcentrifuge. For total DNA purification using a vacuum manifold, see "Protocol: DNA Purification from Buccal Swabs (Vacuum Protocol)" on page 40.

Important points before starting

- Due to the increased volume of Buffer AL that is required for the buccal swab protocol, fewer preparations can be performed. Additional Buffer AL can be purchased separately (see Ordering Information on page 71).
- This protocol is recommended for the following swab types: C.E.P. (Omni Swabs from Whatman Bioscience, www.whatman.com), cotton, and DACRON (Daigger, Puritan® applicators with plastic stick and cotton or DACRON tip from Hardwood Products Company, www.hwppuritan.com , or from Hain Diagnostika, www.hain-diagnostika.de).
- To collect a sample, scrape the swab firmly against the inside of each cheek 6 times. Air-dry the swab for at least 2 h after collection. Ensure that the person providing the sample has not consumed any food or drink in the 30 min prior to sample collection.
- All centrifugation steps are carried out at room temperature (15–25°C).

Things to do before starting

- Prepare a 56°C water bath for use in step 3.
- Equilibrate Buffer AE or distilled water to room temperature for elution in step 10.
- Ensure that Buffer AW1, Buffer AW2, and QIAGEN Protease have been prepared according to the instructions on page 17.
- If a precipitate has formed in Buffer AL, dissolve by incubating at 56°C.

Procedure

1. **Place buccal swab in a 2 ml microcentrifuge tube. Add 400 µl (cotton and DACRON swab) or 600 µl (Omni Swab) PBS to the sample.**

   The Omni Swab is ejected into the microcentrifuge tube by pressing the stem end towards the swab. Cotton or DACRON swabs are separated from the stick by hand or with scissors.

QIAamp Mini spin columns copurify RNA and DNA in parallel when both are present in the sample. RNA may inhibit some downstream enzymatic reactions, but not PCR. If RNA-free genomic DNA is required, 4 µl of an RNase A stock solution (100 mg/ml) should be added to the sample prior to the addition of Buffer AL.

2. **Add 20 µl QIAGEN Protease stock solution (or proteinase K) and 400 µl (cotton or DACRON swab) or 600 µl (Omni Swab) Buffer AL to the sample. Mix immediately by vortexing for 15 s.**

   In order to ensure efficient lysis, it is essential that the sample and Buffer AL are mixed immediately and thoroughly.

   Note: Do not add QIAGEN Protease or proteinase K directly to Buffer AL.

3. **Incubate at 56°C for 10 min. Briefly centrifuge to remove drops from inside the lid.**
4. **Add 400 µl (cotton or DACRON swab) or 600 µl (Omni Swab) ethanol (96–100%) to the sample, and mix again by vortexing. Briefly centrifuge to remove drops from inside the lid.**
5. **Carefully apply 700 µl of the mixture from step 4 to the QIAamp Mini spin column (in a 2 ml collection tube) without wetting the rim. Close the cap, and centrifuge at 6000 x *g* (8000 rpm) for 1 min. Place the QIAamp Mini spin column in a clean 2 ml collection tube (provided), and discard the tube containing the filtrate.***

   Close each spin column in order to avoid aerosol formation during centrifugation.

6. **Repeat step 5 by applying up to 700 µl of the remaining mixture from step 4 to the QIAamp Mini spin column.**
7. **Carefully open the QIAamp Mini spin column and add 500 µl Buffer AW1 without wetting the rim. Close the cap and centrifuge at 6000 x *g* (8000 rpm) for 1 min. Place the QIAamp Mini spin column in a clean 2 ml collection tube (provided), and discard the collection tube containing the filtrate.***
8. **Carefully open the QIAamp Mini spin column and add 500 µl Buffer AW2 without wetting the rim. Close the cap and centrifuge at full speed (20,000 x *g*; 14,000 rpm) for 3 min.**
9. **Recommended: Place the QIAamp Mini spin column in a new 2 ml collection tube (not provided) and discard the old collection tube with the filtrate. Centrifuge at full speed for 1 min.**

   This step helps to eliminate the chance of possible Buffer AW2 carryover.

* Flow-through contains Buffer AL or Buffer AW1 and is therefore not compatible with bleach. See page 8 for safety information.

10. Place the QIAamp Mini spin column in a clean 1.5 ml microcentrifuge tube (not provided), and discard the collection tube containing the filtrate. Carefully open the QIAamp Mini spin column and add 150 µl Buffer AE or distilled water. Incubate at room temperature for 1 min, and then centrifuge at 6000 x *g* (8000 rpm) for 1 min.

Elution with 100 µl buffer increases the final DNA concentration in the eluate significantly, but may slightly reduce the overall DNA yield. Elution with volumes of less than 100 µl is not recommended as the overall DNA yield decreases dramatically.

A second elution step with the same 150 µl eluate containing the DNA will increase yield significantly. However this is not recommended when using the eluate for PCR.

For long-term storage of DNA, eluting in Buffer AE and placing at –20°C is recommended.

One buccal swab typically yields 0.5–3.5 µg of DNA in 150 µl of buffer (3–23 ng/µl), with A_{260}/A_{280} ratios of 1.7–1.9 (measured in water).

Protocol: DNA Purification from Buccal Swabs (Vacuum Protocol)

This protocol is for purification of total (genomic, mitochondrial, and viral) DNA from buccal swabs using the QIAvac 24 Plus or equivalent vacuum manifold. For total DNA purification using a microcentrifuge, see "Protocol: DNA Purification from Buccal Swabs (Spin Protocol)" on page 37.

Important points before starting

- Due to the increased volume of Buffer AL that is required for the buccal swab protocol, fewer preparations can be performed. Additional Buffer AL can be purchased separately (see Ordering Information on page 71).
- This protocol is recommended for the following swab types: C.E.P. (Omni Swabs from Whatman Bioscience, www.whatman.com), cotton, and DACRON (Daigger, Puritan applicators with plastic stick and cotton or DACRON tip from Hardwood Products Company, www.hwppuritan.com , or from Hain Diagnostika, www.hain-diagnostika.de).
- To collect a sample, scrape the swab firmly against the inside of each cheek 6 times. Air-dry the swab for at least 2 h after collection. Ensure that the person providing the sample has not consumed any food or drink in the 30 min prior to sample collection.
- All centrifugation steps are carried out at room temperature (15–25°C).
- For setup of the QIAvac 24 Plus, see pages 20–24.
- Switch off the vacuum between steps to ensure that a consistent, even vacuum is applied during protocol steps.

Things to do before starting

- Prepare a 56°C water bath for use in step 3.
- Equilibrate Buffer AE or distilled water to room temperature for elution in step 10.
- Ensure that Buffer AW1, Buffer AW2, and QIAGEN Protease have been prepared according to the instructions on page 17.
- If a precipitate has formed in Buffer AL, dissolve by incubating at 56°C.

Procedure

1. **Place buccal swab in a 2 ml microcentrifuge tube. Add 400 µl (cotton and DACRON swab) or 600 µl (Omni Swab) PBS to the sample.**

   The Omni Swab is ejected into the microcentrifuge tube by pressing the stem end towards the swab. Cotton or DACRON swabs are cut from the stick by hand or with scissors.

   QIAamp Mini columns copurify RNA and DNA in parallel when both are present in the sample. RNA may inhibit some downstream enzymatic reactions, but not the PCR itself. If RNA-free genomic DNA is required, 4 µl RNase A stock solution (100 mg/ml) should be added to the sample prior to the addition of Buffer AL.

2. **Add 20 µl QIAGEN Protease stock solution (or proteinase K) and 400 µl (cotton or DACRON swab) or 600 µl (Omni Swab) of Buffer AL to the sample. Mix immediately by vortexing for 15 s.**

   In order to ensure efficient lysis, it is essential that the sample and Buffer AL are mixed immediately.

   Note: Do not add QIAGEN Protease or proteinase K directly to Buffer AL.

3. **Incubate at 56°C for 10 min. Briefly centrifuge to remove drops from inside the lid.**

4. **Add 400 µl (cotton or DACRON swab) or 600 µl (Omni Swab) ethanol (96–100%) to the sample, and mix again by vortexing.**

5. **Insert the QIAamp Mini column into a VacConnector on the QIAvac vacuum manifold. Place an extension tube (see Ordering Information, page 69) on the column. Seal unused Luer Adapters with Luer plugs.**

6. **Apply the mixture from step 4 to the QIAamp Mini column. Switch on the vacuum pump to draw the lysate through the QIAamp Mini column. After the lysate has passed through the QIAamp Mini column, switch off the vacuum pump.**

7. **Add 750 µl Buffer AW1 into the extension tube. Switch on the vacuum pump to draw Buffer AW1 through the QIAamp Mini column. Switch off the vacuum pump. Carefully remove the extension tube from the QIAamp Mini column and discard.**

8. **Add 750 µl Buffer AW2 without wetting the rim of the QIAamp Mini column. Leave the lid of the QIAamp Mini column open and switch on the vacuum pump. After all of Buffer AW2 has been drawn through the spin column, switch off the vacuum pump.**

9. **Close the lid of the QIAamp Mini column, remove it from the vacuum manifold, and discard the VacConnector. Place the QIAamp Mini column into a clean 2 ml collection tube and centrifuge at 20,000 x *g* (14,000 rpm) for 1 min to dry the membrane completely.**

10. Place the QIAamp Mini column in a clean 1.5 ml microcentrifuge tube (not provided). Discard the collection tube and the filtrate. Carefully open the QIAamp Mini column. Elute the DNA with 150 µl Buffer AE or distilled water. Incubate at room temperature for 1 min, and then centrifuge at 6000 x *g* (8000 rpm) for 1 min.

Elution with 100 µl buffer increases the final DNA concentration in the eluate significantly, but may slightly reduce the overall DNA yield. Elution with volumes of less than 100 µl is not recommended as overall DNA yield decreases dramatically.

A second elution step with the same 150 µl eluate containing the DNA will increase yield significantly. However this is not recommended when using the eluate for PCR.

For long-term storage of DNA, eluting in Buffer AE and placing at –20°C is recommended.

One buccal swab typically yields 0.5–3.5 µg DNA in 150 µl buffer (3–23 ng/µl), with A_{260}/A_{280} ratios of 1.7–1.9 (measured in water).

Protocol: DNA Purification from Dried Blood Spots (QIAamp DNA Mini Kit)

This protocol is for purification of total (genomic, mitochondrial, and viral) DNA from blood, both untreated and treated with anticoagulants, which has been spotted and dried on filter paper (Schleicher and Schuell 903).

Important point before starting

- All centrifugation steps are carried out at room temperature (15–25°C).

Things to do before starting

- Prepare an 85°C water bath for use in step 2, a 56°C water bath for use in step 3, and a 70°C water bath for use in step 4.
- Equilibrate Buffer AE or distilled water to room temperature for elution in step 10.
- Ensure that Buffer AW1 and Buffer AW2 have been prepared according to the instructions on page 17.
- If a precipitate has formed in Buffer AL or Buffer ATL, dissolve by incubating at 56°C.

Procedure

1. **Place 3 punched-out circles from a dried blood spot into a 1.5 ml microcentrifuge tube and add 180 µl of Buffer ATL.**

   Cut 3 mm (1/8 inch) diameter punches from a dried blood spot with a single-hole paper puncher.

2. **Incubate at 85°C for 10 min. Briefly centrifuge to remove drops from inside the lid.**

3. **Add 20 µl proteinase K stock solution. Mix by vortexing, and incubate at 56°C for 1 h. Briefly centrifuge to remove drops from inside the lid.**

   Note: The addition of proteinase K is essential.

4. **Add 200 µl Buffer AL to the sample. Mix thoroughly by vortexing, and incubate at 70°C for 10 min. Briefly centrifuge to remove drops from inside the lid.**

   In order to ensure efficient lysis, it is essential that the sample and Buffer AL are mixed immediately and thoroughly.

   Note: Do not add proteinase K directly to Buffer AL.

   A white precipitate may form when Buffer AL is added to the sample. In most cases, the precipitate will dissolve during incubation. The precipitate does not interfere with the QIAamp procedure or with any subsequent application.

5. **Add 200 µl ethanol (96–100%) to the sample, and mix thoroughly by vortexing. Briefly centrifuge to remove drops from inside the lid.**

   It is essential that the sample and ethanol are mixed thoroughly.

6. **Carefully apply the mixture from step 5 to the QIAamp Mini spin column (in a 2 ml collection tube) without wetting the rim. Close the cap, and centrifuge at 6000 x *g* (8000 rpm) for 1 min. Place the QIAamp Mini spin column in a clean 2 ml collection tube (provided), and discard the tube containing the filtrate.***

   Close each QIAamp Mini spin column in order to avoid aerosol formation during centrifugation.

7. **Carefully open the QIAamp Mini spin column and add 500 µl Buffer AW1 without wetting the rim. Close the cap and centrifuge at 6000 x *g* (8000 rpm) for 1 min. Place the QIAamp Mini spin column in a clean 2 ml collection tube (provided), and discard the collection tube containing the filtrate.***

8. **Carefully open the QIAamp Mini spin column and add 500 µl Buffer AW2 without wetting the rim. Close the cap and centrifuge at full speed (20,000 x *g*; 14,000 rpm) for 3 min.**

9. **Recommended: Place the QIAamp Mini spin column in a new 2 ml collection tube (not provided) and discard the old collection tube with the filtrate. Centrifuge at full speed for 1 min.**

   This step helps to eliminate the chance of possible Buffer AW2 carryover.

10. **Place the QIAamp Mini spin column in a clean 1.5 ml microcentrifuge tube (not provided), and discard the collection tube containing the filtrate. Carefully open the QIAamp Mini spin column and add 150 µl Buffer AE or distilled water. Incubate at room temperature for 1 min, and then centrifuge at 6000 x *g* (8000 rpm) for 1 min.**

    Three punched-out circles (3 mm diameter) typically yield 150 ng and 75 ng of DNA from anticoagulated and untreated blood, respectively. If the yield from untreated blood is not sufficient, use 6 circles per prep instead of 3.

    The volume of the DNA eluate used in a PCR assay should not exceed 10%; for example, for a 50 µl PCR, add no more than 5 µl of eluate.

* Flow-through contains Buffer AL or Buffer AW1 and is therefore not compatible with bleach. See page 8 for safety information.

Troubleshooting Guide

This troubleshooting guide may be helpful in solving any problems that may arise. The scientists in QIAGEN Technical Services are always happy to answer any questions you may have about either the information and protocols in this handbook or sample and assay technologies (for contact information, see back cover or visit www.qiagen.com).

		Comments and suggestions
**Colored residues remain on the QIAamp Mini spin column after washing**		
a)	Inefficient cell lysis due to insufficient mixing of the sample with Buffer AL	Repeat the DNA purification procedure with a new sample. Be sure to mix the sample and Buffer AL immediately and thoroughly by pulse-vortexing.
b)	Inefficient cell lysis due to decreased protease activity	Repeat the DNA purification procedure with a new sample and with freshly prepared QIAGEN Protease stock solution. Be sure to store the stock solution at 2–8°C immediately after use. Ensure that QIAGEN Protease is not added directly to Buffer AL.
c)	No ethanol added to the lysate before loading onto the QIAamp Mini column	Repeat the purification procedure with a new sample.
d)	Buffer AW1 or AW2 prepared incorrectly	Ensure that Buffer AW1 and AW2 concentrates were diluted with the correct volumes of pure ethanol (see page 17). Do not use denatured alcohol, which contains other substances such as methanol or methylethylketone. Repeat the purification procedure with a new sample.
**Little or no DNA in the eluate**		
a)	Low concentration of cells or viruses in the sample	Concentrate a larger volume of a new cell-free sample to 200 µl using a Centricon®-100 (Amicon, USA). Repeat the DNA purification procedure, adding 5–10 µg of carrier DNA to each lysate (see page 18) if the sample has a low DNA content. If whole blood was used, prepare buffy coat (see procedure on page 19).

		Comments and suggestions
b)	Inefficient cell lysis due to insufficient mixing with Buffer AL	Repeat the DNA purification procedure with a new sample. Be sure to mix the sample and Buffer AL immediately and thoroughly by pulse-vortexing.
c)	Inefficient cell lysis due to decreased protease activity	Repeat the DNA purification procedure with a new sample and with freshly prepared QIAGEN Protease stock solution. Be sure to store the stock solution at 2–8°C immediately after use. Ensure that QIAGEN Protease is not added directly to Buffer AL.
d)	Inefficient cell lysis or protein degradation in Buffer AL or Buffer ATL due to insufficient incubation time	Repeat the procedure with a new sample. Ensure that the tissue sample is cut into small pieces and extend the incubation time. Ensure that no residual particulates are visible (bones or hair will not be lysed at all).
e)	No ethanol added to the lysate before loading onto the QIAamp Mini column	Repeat the purification procedure with a new sample.
f)	Low-percentage ethanol used instead of 100%	Repeat the purification procedure with a new sample. Do not use denatured alcohol, which contains other substances such as methanol or methylethylketone.
g)	Isopropanol used instead of ethanol with samples other than blood or plasma	We strictly recommend the use of ethanol with all samples other than blood or plasma (serum). The use of isopropanol results in reduced yields with all other samples.
h)	QIAamp Mini column not incubated at room temperature (15–25°C) for 1 min	After addition of Buffer AE or water, the QIAamp Mini column should be incubated at room temperature for at least 1 min.
i)	DNA not eluted efficiently	To increase elution efficiency, pipet Buffer AE or water onto the center of the QIAamp Mini column and incubate the column for 5 min at room temperature before centrifugation.
j)	pH of water incorrect (acidic)	Low pH may reduce DNA yield. Ensure that the pH of the water is at least 7.0 or use Buffer AE for elution.

		Comments and suggestions
k)	Buffer AW1 or AW2 prepared incorrectly	Check that Buffer AW1 and AW2 concentrates were diluted with the correct volumes of pure ethanol (see page 17). Do not use denatured alcohol, which contains other substances such as methanol or methylethylketone. Repeat the purification procedure with a new sample.
l)	Buffer AW1 or AW2 prepared with 70% ethanol	Check that Buffer AW1 and AW2 concentrates were diluted with 96–100% ethanol. Repeat the purification procedure with a new sample.
m)	Buffers AW1 and AW2 used in the wrong order	Ensure that Buffers AW1 and AW2 are used in the correct order in the protocol. Repeat the purification procedure with a new sample.
n)	Elution with too much Buffer AE	Elution with volumes of less than 200 µl increases the final DNA concentration in the eluate, but slightly reduces the overall DNA yield (see Table 5 on page 26). For samples containing less than 1 µg of DNA, elution in 50 µl of Buffer AE or water is always recommended.

A_{260}/A_{280} ratio for purified nucleic acids is low

a)	Inefficient cell lysis due to insufficient mixing with Buffer AL	Repeat the procedure with a new sample. Be sure to mix the sample and Buffer AL immediately and thoroughly by pulse-vortexing.
b)	Inefficient cell lysis due to decreased protease activity	Repeat the DNA purification procedure with a new sample and with freshly prepared QIAGEN Protease stock solution. Be sure to store the stock solution at 2–8°C immediately after use. Ensure that QIAGEN Protease is not added directly to Buffer AL.
c)	Inefficient cell lysis or protein degradation in Buffer AL or Buffer ATL due to insufficient incubation time	Repeat the procedure with a new sample. Extend the incubation time. Take care that no residual particulates are visible (bones or hair will not be lysed at all).
d)	No ethanol added to the lysate before loading onto the QIAamp Mini column	Repeat the purification procedure with a new sample.

		Comments and suggestions
e)	Low percentage ethanol used instead of 100%	Repeat the purification procedure with a new sample.
f)	Buffer AW1 or AW2 prepared incorrectly	Check that Buffer AW1 and AW2 concentrates were diluted with the correct volumes of pure ethanol (see page 17). Do not use denatured alcohol, which contains other substances such as methanol or methylethylketone. Repeat the purification procedure with a new sample.
g)	Buffer AW1 or AW2 prepared with 70% ethanol	Check that Buffer AW1 and AW2 concentrates were diluted with 96–100% ethanol. Repeat the purification procedure with a new sample.
h)	Buffers AW1 and AW2 used in the wrong order	Ensure that Buffers AW1 and AW2 are used in the correct order in the protocol. Repeat the purification procedure with a new sample.
**A_{260}/A_{280} ratio for purified nucleic acids is high**		
a)	High level of residual RNA	In future DNA preparations, use the optional RNase step included in the protocols.
b)	Buffer AL added to the sample before addition of RNase A	Always add RNase A first and vortex when using the optional RNase A step.
**DNA does not perform well in subsequent enzymatic reactions**		
a)	Not enough DNA in sample	Check "Little or no DNA in the eluate" on pages 45–47 of this troubleshooting guide for possible reasons. Increase the amount of eluate added to the reaction, if possible. If necessary, vacuum-concentrate the DNA or increase the amount of sample used, and repeat the purification procedure. If the amount of purified DNA is still expected to be low, reduce the elution volume to 50 µl. Lowering the elution volume will slightly reduce the overall yield, but will result in a higher concentration of nucleic acids in the eluate (see Table 5 on page 26). DNA remaining on the QIAamp Mini column can be recovered in a subsequent elution step by applying the same eluate to the column.

		Comments and suggestions
b)	Inhibitory substances in preparation	Check "A_{260}/A_{280} ratio for purified nucleic acids is low" on pages 47–48 for possible reasons.
c)	Residual Buffer AW2 in the eluate	Use recommended drying step in the relevant protocol.<br>Ensure that the QIAamp Mini column does not come into contact with the filtrate prior to elution.
d)	Buffers AW1 and AW2 used in the wrong order	Ensure that Buffers AW1 and AW2 are used in the correct order in the protocol. Repeat the purification procedure with a new sample.
e)	High level of residual RNA	In future DNA preparations, use the optional RNase step included in the protocols.
f)	Reduced sensitivity of amplification reaction	Adjust the volume of eluate added as template in the amplification reaction.
g)	Amplification reaction setup has been modified	Reoptimize the amplification system by adjusting the volume of eluate added.
**White precipitate in Buffer ATL or Buffer AL**		
	White precipitate may form after storage at low temperature or prolonged storage	Any precipitate in Buffer ATL or Buffer AL must be dissolved by incubation of the buffer at 56°C. The precipitate has no effect on function. Dissolving the precipitate at high temperature will not compromise yield or quality of the purified nucleic acids.
**White precipitate in steps 5 or 6 of the tissue protocol**		
	White precipitate may form on addition of Buffer AL in step 5 or ethanol in step 6	In most cases the precipitate formed in step 5 will dissolve during incubation at 70°C. The precipitates do not interfere with the QIAamp procedure or with any subsequent application.

		Comments and suggestions
**General handling**		
a)	Lysate not completely passed through the membrane	**Using spin protocol**: Centrifuge for 1 min at full speed or until all the lysate has passed through the membrane.
		**Using vacuum protocol**: Insufficient vacuum was applied or the lid of the QIAamp Mini column was closed during the vacuum step. Increase the vacuum, and open the lid while applying the vacuum. If the vacuum pressure cannot be increased, place the QIAamp Mini column in a clean 2 ml collection tube, close the cap, and centrifuge at 6000 x *g* (8000 rpm) for 3 min or until the lysate has completely passed through the membrane. Place the QIAamp Mini column into another clean 2 ml collection tube, and discard the tube containing the filtrate. Continue with step 8 of the spin protocol on page 28.
b)	Clogged membrane	**Blood samples**: Concentration of leukocytes in samples was greater than 5 x 10^6/200 µl. Dilute the sample with PBS and repeat the purification.
		**Plasma samples**: Cryoprecipitates have formed in plasma due to repeated freezing and thawing. Do not use plasma that has been frozen and thawed more than once.
c)	Cross-contamination between samples	To avoid cross-contamination when handling QIAamp Mini columns, read "Handling of QIAamp Mini columns" on page 19. Repeat the purification procedure with new samples.
d)	Vacuum pressure too high/too low	Using a vacuum pressure that is too high may damage the QIAamp membrane. Using a vacuum pressure that is too low may cause reduced DNA yield and purity. Use a vacuum regulator (see Ordering Information on page 69) to adjust the pressure to –800 to –900 mbar for all vacuum steps.

Appendix A: Determination of Concentration, Yield, Purity, and Length of DNA

Determination of concentration, yield, and purity

DNA yields are determined from the concentration of DNA in the eluate, measured by absorbance at 260 nm. Purity is determined by calculating the ratio of absorbance at 260 nm to absorbance at 280 nm. Pure DNA has an A_{260}/A_{280} ratio of 1.7–1.9.

Absorbance readings at 260 nm should lie between 0.1 and 1.0 to be accurate. Sample dilution should be adjusted accordingly. Use elution buffer or water (as appropriate) to dilute samples and to calibrate the spectrophotometer. Measure the absorbance at 260 and 280 nm, or scan absorbance from 220–320 nm (a scan will show if there are other factors affecting absorbance at 260 nm). Both DNA and RNA are measured with a spectrophotometer. To measure only DNA, a fluorometer must be used.

Determination of DNA length

The length of genomic DNA can be determined by pulsed-field gel electrophoresis (PFGE) through an agarose gel. The DNA should be concentrated by alcohol* precipitation and reconstituted by gentle agitation in approximately 30 µl TE buffer, pH 8.0,* for at least 30 minutes at 60°C. Avoid drying the DNA pellet for more than 10 minutes at room temperature since over-dried genomic DNA is very difficult to redissolve. Load 3–5 µg DNA per well. Standard PFGE conditions are as follows:

- 1% agarose gel in 0.5x TBE electrophoresis buffer*
- Switch intervals: 5–40 s
- Run time: 17 h
- Voltage: 170 V

Appendix B: Protocol for Cultured Cells

This protocol is for purification of total (genomic, mitochondrial, and viral) DNA from cultured cells using a microcentrifuge.

Additional equipment and reagents required

- Phosphate buffered saline (PBS)*

* When working with chemicals, always wear a suitable lab coat, disposable gloves, and protective goggles. For more information, consult the appropriate material safety data sheets (MSDSs), available from the product supplier.

- Equipment for harvesting cells. Depending on the method chosen, one or more of the following are required:
  - Microcentrifuge
  - Trypsin and culture media*
  - Cell scraper

Important points before starting

- Do not use more than 5 x 10^6 cells (with a normal set of chromosomes).
- All centrifugation steps are carried out at room temperature (15–25°C).
- Use carrier DNA if the sample contains <10,000 genome equivalents (see page 18).

Things to do before starting

- Heat a water bath or heating block to 56°C.
- Equilibrate Buffer AE or distilled water to room temperature for elution.
- Ensure that Buffer AW1, Buffer AW2, and QIAGEN Protease have been prepared according to the instructions on page 17.
- If a precipitate has formed in Buffer AL, dissolve by incubating at 56°C.

Procedure

B1. Harvest cells according to step B1a (for cells grown in suspension) or B1b (for cells grown in a monolayer).

B1a. Cells grown in suspension (do not use more than 5 x 10^6 cells with a normal set of chromosomes): Determine the number of cells. Centrifuge the appropriate number of cells for 5 min at 300 x *g* in a 1.5 ml microcentrifuge tube. Remove the supernatant completely and discard, taking care not to disturb the cell pellet. Continue with step B2.

B1b. Cells grown in a monolayer (do not use more than 5 x 10^6 cells with a normal set of chromosomes): Cells grown in a monolayer can be detached from the culture flask by either trypsinization or using a cell scraper.

To trypsinize cells:

Determine the number of cells. Aspirate the medium and wash cells with PBS. Aspirate the PBS, and add 0.10–0.25% trypsin. After cells have detached from the dish or flask, collect them in medium, and transfer the appropriate number of

* When working with chemicals, always wear a suitable lab coat, disposable gloves, and protective goggles. For more information, consult the appropriate material safety data sheets (MSDSs), available from the product supplier.

cells (do not use more than 5 x 10^6 cells with a normal set of chromosomes) to a 1.5 ml microcentrifuge tube. Centrifuge for 5 min at 300 x *g*. Remove the supernatant completely and discard, taking care not to disturb the cell pellet. Continue with step B2.

Using a cell scraper:

Detach cells from the dish or flask. Transfer the appropriate number of cells (do not use more than 5 x 10^6 cells with a normal set of chromosomes) to a 1.5 ml microcentrifuge tube and centrifuge for 5 min at 300 x *g*. Remove the supernatant completely and discard, taking care not to disturb the cell pellet. Continue with step B2.

B2. Resuspend cell pellet in PBS to a final volume of 200 µl.

B3. Add 20 µl QIAGEN Protease or proteinase K.

B4. Continue with step 3 of "Protocol: DNA Purification from Blood or Body Fluids (Spin Protocol)", page 28.

Appendix C: Protocols for Fixed Tissues

The QIAamp DNA FFPE Tissue Kit is recommended for purification of DNA from formalin-fixed, paraffin-embedded (FFPE) tissues. The kit combines the selective binding properties of a silica-based membrane with flexible elution volumes of between 20 and 100 µl. Specially optimized lysis conditions allow genomic DNA to be efficiently purified from FFPE tissue sections without the need for overnight incubation.

The QIAamp DNA Mini Kit can also be used to isolate DNA from fixed tissues. However, the length of DNA isolated from fixed tissues is usually <650 bp, depending on the type and age of the sample and the quality of the fixative used (Wright, D. K. and Manos, M. M. [1990] Sample preparation from paraffin-embedded tissues. In: Innis, M.A., Gelfont, D. H., Sninsky, J. J., White, T. J., eds. *PCR Protocols: A guide to methods and applications.* San Diego: Academic Press, p. 153–158).

Use of fixatives such as alcohol and formalin are recommended. Fixatives that cause cross-linking, such as osmium tetroxide, are not recommended as it can be difficult to obtain amplifiable DNA from tissue fixed with these agents.

Additional reagents required

- For paraffin-embedded tissues: Xylene*
- For formalin-fixed tissues: Phosphate-buffered saline (PBS)*

* When working with chemicals, always wear a suitable lab coat, disposable gloves, and protective goggles. For more information, consult the appropriate material safety data sheets (MSDSs), available from the product supplier.

Important points before starting

- Lysis time will vary from sample to sample depending on the type of tissue processed.
- Yields will depend both on the size and the age of the sample processed. Reduced yields compared to fresh or frozen tissues are expected. Therefore, eluting the DNA in 50–100 µl Buffer AE is recommended.
- All centrifugation steps are carried out at room temperature (15–25°C).
- Use carrier DNA if the sample contains <10,000 genome equivalents (see page 18).
- Avoid repeated freezing and thawing of stored samples, since this leads to reduced DNA size.
- Transcriptionally active tissues, such as liver and kidney, contain high levels of RNA which will copurify with genomic DNA. RNA may inhibit some downstream enzymatic reactions, but will not inhibit PCR. If RNA-free genomic DNA is required, include the RNase A digest, as described in step 5a of the protocol.

Things to do before starting

- Equilibrate the sample to room temperature (15–25°C).
- Heat 2 water baths or heating blocks: one to 56°C and one to 70°C.
- Equilibrate Buffer AE or distilled water to room temperature for elution.
- Ensure that Buffers AW1 and AW2 have been prepared according to the instructions on page 17.
- If a precipitate has formed in Buffer ATL or Buffer AL, dissolve by incubating at 56°C.

Isolation of genomic DNA from paraffin-embedded tissue

This protocol describes the removal of paraffin by extraction with xylene. The tissue sample is then processed according to the "Protocol: DNA Purification from Tissues" (page 33).

Note: Several customers have reported that it is not necessary to remove the paraffin by xylene extraction before processing. The paraffin melts during the 56°C incubation and does not affect the QIAamp procedure. While this may not work for all types of paraffin-embedded samples, you may wish to try omitting the xylene-extraction protocol, since it makes the isolation procedure much simpler.

Procedure

C1. Place a small section (not more than 25 mg) of paraffin-embedded tissue in a 2 ml microcentrifuge tube (not provided).

C2. Add 1200 µl xylene. Vortex vigorously.

C3. Centrifuge at full speed for 5 min at room temperature.

C4. Remove supernatant by pipetting. Do not remove any of the pellet.

C5. Add 1200 µl ethanol (96–100%) to the pellet to remove residual xylene and mix gently by vortexing.

C6. Centrifuge at full speed for 5 min at room temperature (15–25°C).

C7. Carefully remove the ethanol by pipetting. Do not remove any of the pellet.

C8. Repeat steps C5–C7 once.

C9. Incubate the open microcentrifuge tube at 37°C for 10–15 min until the ethanol has evaporated.

C10. Resuspend the tissue pellet in 180 µl Buffer ATL and follow the "Protocol: DNA Purification from Tissues" from step 3 (page 34).

Isolation of genomic DNA from formalin-fixed tissues

C1. Wash tissue sample twice with PBS to remove fixative.

C2. Process sample according to the "Protocol: DNA Purification from Tissues" on page 33.

Appendix D: Protocols for Bacteria

These protocols have been used successfully for bacteria such as *Escherichia coli, Bacillus subtilis, Bordetella pertussis* from nasopharyngeal swabs, *Borrelia burgdorferi* from cerebrospinal fluid, and *Legionella pneumophila* from broncho-alveolar lavage. For other bacteria, follow the protocol for Gram-positive bacteria, especially other Gram-positive bacteria, which may be difficult to lyse.

For isolation of bacterial DNA from urine, either follow the protocol for biological fluids, or use the QIAamp Viral RNA Mini Kit. Urine contains numerous unidentified PCR inhibitors. Buffer AVL (included in the QIAamp Viral RNA Mini Kit) is the buffer of choice to destroy these inhibitors.

Some bacteria (particularly Gram-positive bacteria) require pre-incubation with specific enzymes such as lysozyme* or lysostaphin* (e.g., staphylococci) to lyse the rigid multilayered cell wall. In these cases the protocol for Gram-positive bacteria should be used.

* When working with chemicals, always wear a suitable lab coat, disposable gloves, and protective goggles. For more information, consult the appropriate material safety data sheets (MSDSs), available from the product supplier.

Additional reagents required

- For swabs: Phosphate-buffered saline (PBS)* containing a common fungicide*
- For Gram-positive and difficult-to-lyse bacteria: 20 mg/ml lysozyme or 200 µg/ml lysostaphin solution in 20 mM Tris·Cl,* pH 8.0, 2 mM EDTA,* 1.2% Triton®*

Important points before starting

- All centrifugation steps are carried out at room temperature (15–25°C).
- Use carrier DNA if the sample contains <10,000 genome equivalents (see page 18).
- Avoid repeated freezing and thawing of stored samples, since this leads to reduced DNA size.

Things to do before starting

- Equilibrate the sample to room temperature (15–25°C).
- Heat 2 water baths or heating blocks: one to 56°C and one to 70°C.
- Equilibrate Buffer AE or distilled water to room temperature for elution.
- Ensure that Buffers AW1 and AW2 have been prepared according to the instructions on page 17.
- If a precipitate has formed in Buffer ATL or Buffer AL, dissolve by incubating at 56°C.

Isolation of bacterial DNA from biological fluids

D1. Pellet bacteria by centrifugation for 10 min at 5000 x *g* (7500 rpm).

D2. Resuspend bacterial pellet in 180 µl Buffer ATL (supplied in the QIAamp DNA Mini Kit).

D3. Follow the "Protocol: DNA Purification from Tissues" from step 3 (page 34).

Isolation of bacterial DNA from eye, nasal, pharyngeal, or other swabs†

D1. Collect samples and place in 2 ml PBS containing a common fungicide. Incubate for several hours at room temperature.

D2. Follow the biological fluids protocol above from step D1.

* When working with chemicals, always wear a suitable lab coat, disposable gloves, and protective goggles. For more information, consult the appropriate material safety data sheets (MSDSs), available from the product supplier.

† See also "Protocol: DNA Purification from Buccal Swabs (Spin Protocol)" on page 37.

Isolation of genomic DNA from bacterial plate cultures

D1. Remove bacteria from culture plate with an inoculation loop and suspend in 180 µl of Buffer ATL (supplied in the QIAamp DNA Mini Kit) by vigorous stirring.

D2. Follow the "Protocol: DNA Purification from Tissues" from step 3 (page 34).

Isolation of genomic DNA from bacterial suspension cultures

D1. Pipet 1 ml of bacterial culture into a 1.5 ml microcentrifuge tube, and centrifuge for 5 min at 5000 x *g* (7500 rpm).

D2. Calculate the volume of the pellet or concentrate and add Buffer ATL (supplied in the QIAamp DNA Mini Kit) to a total volume of 180 µl.

D3. Follow the "Protocol: DNA Purification from Tissues" from step 3 (page 34).

Isolation of genomic DNA from Gram-positive bacteria

D1. Pellet bacteria by centrifugation for 10 min at 5000 x *g* (7500 rpm).

D2. Suspend bacterial pellet in 180 µl of the appropriate enzyme solution (20 mg/ml lysozyme or 200 µg/ml lysostaphin; 20 mM Tris·HCl, pH 8.0; 2 mM EDTA; 1.2% Triton).

D3. Incubate for at least 30 min at 37°C.

D4. Add 20 µl proteinase K and 200 µl Buffer AL. Mix by vortexing.

D5. Incubate at 56°C for 30 min and then for a further 15 min at 95°C.

Note: Extended incubation at 95°C can lead to some DNA degradation.

D6. Centrifuge for a few seconds.

D7. Follow the "Protocol: DNA Purification from Tissues" from step 6 (page 34).

Appendix E: Protocol for Yeast (e.g., Cultured *Candida* spp.)

Additional reagents required

- Sorbitol buffer (1 M sorbitol, 100 mM EDTA, 14 mM β-mercaptoethanol)*
- Zymolase or lyticase*

* When working with chemicals, always wear a suitable lab coat, disposable gloves, and protective goggles. For more information, consult the appropriate material safety data sheets (MSDSs), available from the product supplier.

Important points before starting

- Lysis time and yield will vary from sample to sample depending on the cell number and species processed. 3 ml of log-phase culture will yield approximately 15–25 µg of DNA in 400 µl of water (37–62 ng/µl), with an A_{260}/A_{280} ratio of 1.6–1.8.
- A third elution with 200 µl of Buffer AE or water will increase yield.
- All centrifugation steps are carried out at room temperature (15–25°C).
- Use carrier DNA if the sample contains <10,000 genome equivalents (see page 18).
- Avoid repeated freezing and thawing of stored samples, since this leads to reduced DNA size.

Things to do before starting

- Equilibrate the sample to room temperature (15–25°C).
- Heat 3 water baths or heating blocks: one to 30°C, one to 56°C, and one to 70°C.
- Equilibrate Buffer AE or distilled water to room temperature for elution.
- Ensure that Buffers AW1 and AW2 have been prepared according to the instructions on page 17.
- If a precipitate has formed in Buffer ATL or Buffer AL, dissolve by incubating at 56°C.

Procedure

E1. Grow yeast culture in YPD medium* to an OD_{600} of 10.

E2. Harvest 3 ml of culture by centrifuging for 10 min at 5000 x *g* (7500 rpm).

E3. Resuspend the pellet in 600 µl sorbitol buffer. Add 200 U zymolase or lyticase and incubate at 30°C for 30 min.

E4. Pellet the spheroplasts by centrifuging for 5 min at 5000 x *g* (7500 rpm).

E5. Resuspend the spheroplasts in 180 µl Buffer ATL (supplied in the QIAamp DNA Mini Kit).

E6. Follow the "Protocol: DNA Purification from Tissues" from step 3 (page 34).

* When working with chemicals, always wear a suitable lab coat, disposable gloves, and protective goggles. For more information, consult the appropriate material safety data sheets (MSDSs), available from the product supplier.

Appendix F: Protocols for Viral DNA

For simultaneous purification of viral DNA and RNA from plasma or serum, we recommend using the QIAamp MinElute Virus Vacuum Kit or the QIAamp MinElute Virus Spin Kit. These kits provide viral nucleic acid purification with minimal elution volumes for higher sensitivity in downstream applications. All buffers and components of these kits are guaranteed to be RNase-free. Viral nucleic acid purification using the QIAamp MinElute Virus Spin Kit can be fully automated on the QIAcube for increased standardization and ease of use.

Important points before starting

- Stool, plasma, serum, urine, cerebrospinal fluid, and other body fluids often contain very low numbers of cells or viruses. In these cases, concentrating samples from up to 3.5 ml to a final volume of 200 µl, as described in the "Protocol for sample concentration" on page 66, is recommended.
- All centrifugation steps are carried out at room temperature (15–25°C).
- Use carrier DNA if the sample contains <10,000 genome equivalents (see page 18).

Things to do before starting

- Equilibrate samples to room temperature.
- Heat a water bath or heating block to 56°C.
- Equilibrate Buffer AE or distilled water to room temperature for elution.
- Ensure that Buffer AW1, Buffer AW2, and QIAGEN Protease have been prepared according to the instructions on page 17.
- If a precipitate has formed in Buffer AL, dissolve by incubating at 56°C.

Integrated viral DNA

Integrated viral DNA is prepared by the same procedures as genomic DNA (see standard protocols).

Free viral DNA from fluids or suspensions

For preparation of DNA from free viral particles in fluids or suspensions (other than urine) using the "Protocol: DNA Purification from Blood or Body Fluid" protocols we recommend the addition of 1 µl of an aqueous solution containing 5–10 µg of carrier DNA (e.g., poly dA, poly dT, poly dA:dT)* to 200 µl Buffer AL.

* When working with chemicals, always wear a suitable lab coat, disposable gloves, and protective goggles. For more information, consult the appropriate material safety data sheets (MSDSs), available from the product supplier.

To ensure binding conditions are optimal, increase the volume of ethanol added at step 6 from 200 µl to 230 µl.

Elution should be in 60 µl Buffer AE.

Free viral DNA from stool

Additional equipment and reagents required

- 0.89% saline solution*
- 0.22 µm filter

Procedure

F1. Suspend 0.5–1.0 ml of a stool specimen in not more than 5 ml of 0.89% NaCl (maximum dilution 1:10).

F2. Clarify the solution by centrifugation for 20 min at 4000 x *g*.

F3. Filter supernatant through a 0.22 µm filter.

Filtration will remove cells from the sample, eliminating cellular DNA from the preparation.

F4. Pipet 200 µl of the filtrate into a 1.5 ml microcentrifuge tube. Add 20 µl QIAGEN Protease and continue with the "Protocol: DNA Purification from Blood or Body Fluids (Spin Protocol)" from step 3 (page 28).

Free viral DNA from eye, nasal, pharyngeal, or other swabs

Additional reagent required

- Phosphate-buffered saline (PBS) containing a common fungicide*

Procedure

F1. Collect samples and transfer to 2 ml PBS containing a common fungicide and bactericide. Incubate for 2–3 h at room temperature.

F2. Concentrate the samples from 2 ml to 200 µl as described in the "Protocol for sample concentration" on page 66.

F3. Pipet 200 µl concentrate into a 1.5 ml microcentrifuge tube. Add 20 µl QIAGEN Protease and continue with the "Protocol: DNA Purification from Blood or Body Fluid (Spin Protocol)" from step 3 (page 28).

* When working with chemicals, always wear a suitable lab coat, disposable gloves, and protective goggles. For more information, consult the appropriate material safety data sheets (MSDSs), available from the product supplier.

Viral DNA from urine

Use the QIAamp Viral RNA Mini Kit. Urine contains numerous unidentified PCR inhibitors. Buffer AVL (included in the QIAamp Viral RNA Mini Kit) is the buffer of choice to inactivate these inhibitors.

Eluting the DNA in 50–100 µl elution buffer or water is recommended.

Appendix G: Protocols for Eye, Nasal, or Pharyngeal Swabs*

Stool, plasma, serum, urine, cerebrospinal fluid, and other body fluids often contain very low numbers of cells or viruses. In these cases, concentrating samples from up to 3.5 ml to a final volume of 200 µl, as described in the "Protocol for Sample Concentration" on page 66, is recommended.

DNA viruses

See "Protocols for Viral DNA" on page 59.

Bacteria

See "Protocols for Bacteria" on page 55.

Cells

Additional reagent required

- Phosphate-buffered saline (PBS) containing a common fungicide†

Important points before starting

- All centrifugation steps are carried out at room temperature (15–25°C).
- Use carrier DNA if the sample contains <10,000 genome equivalents (see page 18).

* See also "Protocol: DNA Purification from Buccal Swabs (Spin Protocol)" on page 37.

† When working with chemicals, always wear a suitable lab coat, disposable gloves, and protective goggles. For more information, consult the appropriate material safety data sheets (MSDSs), available from the product supplier.

Things to do before starting

- Equilibrate samples to room temperature.
- Heat a water bath or heating block to 56°C.
- Equilibrate Buffer AE or distilled water to room temperature for elution.
- Ensure that Buffer AW1, Buffer AW2, and QIAGEN Protease have been prepared according to the instructions on page 17.
- If a precipitate has formed in Buffer AL, dissolve by incubating at 56°C.

Procedure

G1. Collect samples and transfer into 2 ml PBS containing a common fungicide and bactericide. Incubate for 2–3 h at room temperature (15–25°C).

G2. Concentrate the samples from 2 ml to 200 µl as described in the "Protocol for sample concentration" on page 66. Alternatively pellet the cells by centrifuging for 10 min at 5000 x *g* (7500 rpm).

G3. Pipet 200 µl concentrate into a 1.5 ml microcentrifuge tube. Alternatively resuspend the cell pellet in 200 µl PBS. Add 20 µl QIAGEN Protease and continue with the "Protocol: DNA Purification from Blood or Body Fluids (Spin Protocol)" from step 3 (page 28).

Eluting the DNA in 50–100 µl of Buffer AE or water is recommended.

Appendix H: Protocol for Mitochondrial DNA from Platelets

Additional reagent required

- Due to the increased volumes of Buffer AL and QIAGEN Protease that are required for the following protocol, fewer preparations can be performed. Additional Buffer AL and QIAGEN Protease can be purchased separately (see Ordering Information on page 71).

Important point before starting

- All centrifugation steps are carried out at room temperature (15–25°C).

Things to do before starting

- Equilibrate samples to room temperature.
- Heat a water bath or heating block to 56°C.
- Equilibrate Buffer AE or distilled water to room temperature for elution.

- Ensure that Buffer AW1, Buffer AW2, and QIAGEN Protease have been prepared according to the instructions on page 17.
- If a precipitate has formed in Buffer AL, dissolve by incubating at 56°C.

Procedure

H1. Draw blood in the presence of a common anticoagulant.

H2. Take 8 ml of the blood and prepare platelet-rich plasma by centrifugation at 100 x *g* for 15 min at room temperature.

H3. Transfer upper layer into a new tube and remove residual blood cells by centrifugation at 200 x *g* for 10 min at room temperature (15–25°C).

H4. Transfer supernatant to a new tube.

H5. Add 400 µl platelet suspension to a 1.5 ml microcentrifuge tube containing 40 µl QIAGEN Protease or proteinase K. Add 400 µl Buffer AL and mix thoroughly by vortexing.

H6. Incubate at 56°C for 10 min. Briefly centrifuge to remove drops from inside the lid.

H7. Add 400 µl ethanol (96–100%), and mix again by vortexing. Briefly centrifuge to remove drops from inside the lid.

H8. Apply 620 µl of the lysate to the QIAamp Mini spin column (in a 2 ml collection tube) without wetting the rim. Close the cap, and centrifuge at 6000 x *g* (8000 rpm) for 1 min. Place the QIAamp Mini spin column in a clean 2 ml collection tube (provided), and discard the tube containing the filtrate.*

H9. Apply the remainder of the lysate to the QIAamp Mini spin column without wetting the rim. Close the cap, and centrifuge at 6000 x *g* (8000 rpm) for 1 min. Place the QIAamp Mini spin column in a clean 2 ml collection tube, and discard the tube containing the filtrate.*

H10. Continue with step 8 of the "Protocol: DNA Purification from Blood or Body Fluids (Spin Protocol)" (page 28).

Eluting the DNA in 50–100 µl of Buffer AE or water is recommended.

Appendix I: Protocol for CSF and Bone Marrow on Hematological Slides

Additional equipment and reagents required

- Phosphate-buffered saline (PBS)
- Clean microscope slide

* Flow-through contains Buffer AL or Buffer AW1 and is therefore not compatible with bleach. See page 8 for safety information.

Important points before starting

- All centrifugation steps are carried out at room temperature (15–25°C).
- Use carrier DNA if the sample contains <10,000 genome equivalents (see page 18).

Things to do before starting

- Equilibrate samples to room temperature.
- Heat a water bath or heating block to 56°C.
- Equilibrate Buffer AE or distilled water to room temperature for elution.
- Ensure that Buffer AW1, Buffer AW2, and QIAGEN Protease have been prepared according to the instructions on page 17.
- If a precipitate has formed in Buffer AL, dissolve by incubating at 56°C.

Procedure

I1. Moisten the dried material with a drop of PBS.

I2. Add 180 µl PBS to a 1.5 ml microcentrifuge tube.

I3. Scrape cytological material into the microcentrifuge tube using the edge of a clean slide.

I4. Dissolve the resulting sludge by pipetting up and down.

I5. Add 20 µl QIAGEN Protease and continue with the "Protocol: DNA Purification from Blood or Body Fluids (Spin Protocol)" from step 3 (page 28).

Appendix J: Protocol for Crude Cell Lysates and Other Samples

For preparation of genomic DNA from samples other than those listed in this handbook or for which specialized protocols are not available, the following procedure is recommended.

QIAGEN is continuously developing and optimizing QIAamp protocols for new sample sources not included in this handbook. Additional preliminary protocols developed by customers are available for bone, hair, nails, sperm, fungi, and many other sample types. Please contact one of our Technical Service Departments or your local distributor (see back cover or visit www.qiagen.com) for more information.

Additional reagent required

- Cell lysis buffer*

Important points before starting

- Optimal lysis conditions must first be found for the specific sample being processed. QIAamp lysis buffers are not suitable for all sample sources.
- All centrifugation steps are carried out at room temperature (15–25°C).
- Use carrier DNA if the sample contains <10,000 genome equivalents (see page 18).

Things to do before starting

- Equilibrate samples to room temperature.
- Heat a water bath or heating block to 56°C.
- Equilibrate Buffer AE or distilled water to room temperature for elution.
- Ensure that Buffer AW1, Buffer AW2, and QIAGEN Protease have been prepared according to the instructions on page 17.
- If a precipitate has formed in Buffer AL, dissolve by incubating at 56°C.

Procedure

J1. Lyse sample in the sample-specific lysis buffer in as small a volume as possible (200 µl of lysis buffer is optimal).

J2. Estimate the volume of the lysate.

J3. Add 20 µl proteinase K per 200 µl lysate.

J4. Add 200 µl Buffer AL per 200 µl lysate.

J5. Mix immediately by pulse-vortexing for 15 s.

J6. Incubate at 56°C for 10 min. Briefly centrifuge to remove drops from inside the lid.

J7. Check the pH of the lysate. The pH must be acidic (<7.0) to obtain maximum binding of DNA to the QIAamp membrane.

J8. Add 200 µl ethanol (96–100%) per 200 µl lysate, and mix again by pulse-vortexing for 15 s. Briefly centrifuge to remove drops from inside the lid.

* When working with chemicals, always wear a suitable lab coat, disposable gloves, and protective goggles. For more information, consult the appropriate material safety data sheets (MSDSs), available from the product supplier.

J9. Apply 620 µl of the lysate to the QIAamp Mini spin column (in a 2 ml collection tube) without wetting the rim. Close the cap, and centrifuge at 6000 x *g* (8000 rpm) for 1 min. Place the QIAamp Mini spin column in a clean 2 ml collection tube (provided), and discard the tube containing the filtrate.*

J10. Repeat step J9 until the whole lysate is loaded. A maximum of 5 x 620 µl can be loaded onto the QIAamp Mini spin column.

J11. Continue with step 8 of the "Protocol: DNA Purification from Blood or Body Fluids (Spin Protocol)" (page 28).

Note: Yields will vary from sample to sample depending on the cell number and species processed.

Appendix K: Protocol for Sample Concentration

Plasma, serum, urine, cerebrospinal fluid, and other body fluids often contain very low numbers of cells, bacteria, or viruses. In these cases, concentrating samples from up to 3.5 ml to a final volume of 200 µl is recommended.

Additional equipment required

- Centrifugal microconcentrators such as Amicon® Centricon-100 (Millipore, 2 ml), Microsep 100 (Filtron, 3.5 ml), and UltraFree®- CL (Millipore, 2 ml), or equivalents from other suppliers

Procedure

K1. Apply up to 3.5 ml sample to the microconcentrator, according to manufacturer's instructions.

K2. Centrifuge according to manufacturer's instructions to a final volume of 200 µl.

It may not always be possible to concentrate samples to 200 µl due to the high viscosity of the sample (e.g., plasma). In these cases, centrifugation for 6 h is recommended.

K3. Pipet 200 µl concentrate into a 1.5 ml microcentrifuge tube and follow the appropriate QIAamp protocol for the specific sample.

* Flow-through contains Buffer AL or Buffer AW1 and is therefore not compatible with bleach. See page 8 for safety information.

Ordering Information

Product	Contents	Cat. no.
**QIAamp DNA Mini Kits — for genomic DNA purification from tissue, blood, and body fluids**		
QIAamp DNA Mini Kit (50)	For 50 DNA preps: 50 QIAamp Mini Spin Columns, Proteinase K, Reagents, Buffers, Collection Tubes (2 ml)	51304
QIAamp DNA Mini Kit (250)	For 250 DNA preps: 250 QIAamp Mini Spin Columns, Proteinase K, Reagents, Buffers, Collection Tubes (2 ml)	51306
**QIAamp DNA Blood Mini Kits — for genomic DNA purification from blood and body fluids**		
QIAamp DNA Blood Mini Kit (50)	For 50 DNA minipreps: 50 QIAamp Mini Spin Columns, QIAGEN Protease, Reagents, Buffers, Collection Tubes (2 ml)	51104
QIAamp DNA Blood Mini Kit (250)	For 250 DNA minipreps: 250 QIAamp Mini Spin Columns, QIAGEN Protease, Reagents, Buffers, Collection Tubes (2 ml)	51106
**QIAcube and QIAcube accessories— for fully automated sample preparation using QIAGEN spin-column kits**		
QIAcube<br>(110 V)*†<br>(230 V)‡	Robotic workstation for automated purification of nucleic acids or proteins using QIAGEN spin-column kits, 1-year warranty on parts and labor	9001292*†<br>9001293‡
Warranty PLUS 2 Full, QIAcube	3-year warranty, 48-hour (2 working days) priority response, all labor, travel, and repair parts	9240834

* US and Canada.

† Japan.

‡ Rest of world.

Ordering Information

Product	Contents	Cat. no.
Starter Pack, QIAcube	Pack includes: reagent bottle racks (3); rack labeling strips (8); 200 µl filter-tips (1024); 1000 µl filter-tips (1024); 1000 µl filter-tips, wide-bore (1024); 30 ml reagent bottles (18); rotor adapters (240); rotor adapter holder	990395
Filter-Tips, 200 µl (1024)	Sterile, Disposable Filter-Tips, racked	990332
Filter-Tips, 1000 µl (1024)	Sterile, Disposable Filter-Tips, racked	990352
Filter-Tips, 1000 µl, wide-bore (1024)	Sterile, Disposable Filter-Tips, wide-bore, racked; (8 x 128); not required for all protocols	990452
Rotor Adapters (10 x 24)	For 240 preps: 240 Disposable Rotor Adapters; for use with the QIAcube	990394
QIAamp DNA Blood Mini Accessory Set A	Additional Buffers and Reagents; typically for use with 10 x QIAamp DNA Blood Mini Kits (50), catalog number 51104, on the QIAcube	1043368
QIAamp DNA Blood Mini Accessory Set B	Additional Buffers and Reagents; typically for use with 2 x QIAamp DNA Blood Mini Kits (250), catalog number 51106, on the QIAcube	1043369
QIAamp DNA Accessory Set A	Additional Buffers and Reagents; for use with at least 12 x QIAamp DNA Mini Kits (50), catalog number 51304, or 8 x QIAamp DNA Stool Mini Kits (50), catalog number 51504, on the QIAcube	1048145
QIAamp DNA Accessory Set B	Additional Buffers and Reagents; for use with at least 4 x QIAamp DNA Mini Kits (250), catalog number 51306, on the QIAcube	1048146
**QIAvac 24 Plus System — for vacuum processing of 1–24 QIAGEN spin columns**		
QIAvac 24 Plus	Vacuum Manifold for processing 1–24 spin columns: includes QIAvac 24 Plus Vacuum Manifold, Luer Plugs, Quick Couplings	19413

Ordering Information

Product	Contents	Cat. no.
VacConnectors (500)	500 disposable connectors for use with QIAamp spin columns on luer slots or VacValves	19407
Vacuum Regulator	For use with QIAvac manifolds	19530
Vacuum Pump<br>(115 V, 60 Hz)*<br>(110 V, 60 Hz)†<br>(230 V, 50 Hz)‡	Universal vacuum pump (capacity 34 L/min, 8 mbar vacuum abs.)	84010*<br>84000†<br>84020‡
Extension Tubes (3 ml)	For use with QIAGEN spin columns on vacuum manifolds: 100 per pack	19587
VacValves (24)	24 valves for use with the QIAvac 24 Plus	19408
QIAvac Connecting System	System to connect vacuum manifold with vacuum pump: includes Tray, Waste Bottles, Tubing, Couplings, Valve, Gauge, 24 VacValves	19419
**Related products**		
**QIAamp DNA Blood Kits — for genomic DNA purification from blood and body fluids**		
QIAamp DNA Blood Midi Kit (20)§	For 20 DNA midipreps: 20 QIAamp Midi Spin Columns, QIAGEN Protease, Buffers, Collection Tubes (15 ml)	51183
QIAamp DNA Blood Maxi Kit (10)§	For 10 DNA maxipreps: 10 QIAamp Maxi Spin Columns, QIAGEN Protease, Buffers, Collection Tubes (50 ml)	51192
**QIAamp 96 DNA Blood Kits¶ — for high-throughput genomic DNA purification from blood and body fluids**		
QIAamp 96 DNA Blood Kit (4)§	For 4 x 96 DNA preps: 4 QIAamp 96 Plates, QIAGEN Protease, Reagents, Buffers, Lysis Blocks, Tape Pads, Collection Vessels	51161

* US and Canada.

† Japan.

‡ Rest of world.

§ Larger kit sizes available; please inquire.

¶ Requires use of the QIAGEN 96-Well-Plate Centrifugation System; please inquire.

Ordering Information

Product	Contents	Cat. no.
**QIAamp MinElute Virus Kits — for purification of viral RNA from plasma, serum, and cell-free body fluids**		
QIAamp MinElute Virus Spin Kit (50)*	For 50 preps: 50 QIAamp MinElute Columns, QIAGEN Protease, Carrier RNA, Buffers, Collection Tubes (2 ml)	57704
QIAamp MinElute Virus Vacuum Kit (50)	For 50 preps: 50 QIAamp MinElute Columns, QIAGEN Protease, Carrier RNA, Buffers, Extension Tubes (3 ml), Collection Tubes (2 ml)	57714
**QIAamp Viral RNA Mini Kit — for purification of viral RNA from cell-free body fluids**		
QIAamp Viral RNA Mini Kit (50)*†	For 50 RNA preps: 50 QIAamp Mini Spin Columns, Carrier RNA, Collection Tubes (2 ml), RNase-free Buffers	52904
**QIAamp DNA Stool Mini Kit — for purification of up to 30 µg genomic, bacterial, viral, and parasite DNA from stool**		
QIAamp DNA Stool Mini Kit (50)*	For 50 DNA preps: 50 QIAamp Mini Spin Columns, QIAGEN Proteinase K, InhibitEX® tablets, Buffers, Collection Tubes (2 ml)	51504
**QIAamp DNA FFPE Tissue Kit — for purification of genomic DNA from formalin-fixed, paraffin-embedded tissues**		
QIAamp DNA FFPE Tissue Kit (50)	For 50 DNA preps: 50 QIAamp MinElute Columns, Proteinase K, Buffers, Collection Tubes (2 ml)	56404
**QIAamp DNA Investigator Kit — for purification of total (genomic and mitochondrial) DNA from forensic and human identity samples**		
QIAamp DNA Investigator Kit (50)*	For 50 DNA preps: 50 QIAamp MinElute Columns, Proteinase K, Carrier RNA, Buffers, Collection Tubes (2 ml)	56504

* Fully automatable on the QIAcube. See www.qiagen.com/MyQIAcube for protocols.

† Larger kit sizes available; please inquire.

Ordering Information

Product	Contents	Cat. no.
**Accessories**		
TissueRuptor<br>(120 V, 50/60 Hz)<br>(230 V, 50/60 Hz)	Handheld rotor–stator homogenizer, 5 TissueRuptor Disposable Probes	Varies
TissueRuptor Disposable Probes (25)	25 nonsterile plastic disposable probes for use with the TissueRuptor	990890
Buffer AW1 (concentrate, 242 ml)	242 ml Wash Buffer (1) Concentrate for 1000 spin, 250 midi, or 100 maxi preps	19081
Buffer AW2 (concentrate, 324 ml)	324 ml Wash Buffer (2) Concentrate	19072
Buffer AVL (155 ml)	155 ml Viral Lysis Buffer and 4.2 mg Carrier RNA for 250 preps	19073
Buffer AL (216 ml)	216 ml Lysis Buffer AL	19075
Buffer ATL (200 ml)	200 ml Tissue Lysis Buffer for 1000 preps	19076
Buffer AE (240 ml)	240 ml Elution Buffer AE	19077
Collection Tubes (2 ml)	1000 Collection Tubes (2 ml)	19201
QIAGEN Protease (7.5 AU)	7.5 Anson units per vial (lyophilized)	19155
QIAGEN Protease (30 AU)	4 x 7.5 Anson units per vial (lyophilized)	19157
QIAGEN Proteinase K (2 ml)	2 ml (>600 mAU/ml, solution)	19131
QIAGEN Proteinase K (10 ml)	10 ml (>600 mAU/ml, solution)	19133
RNase A (17,500 U)	2.5 ml (100 mg/ml; 7000 units/ml, solution)	19101

For up-to-date licensing information and product-specific disclaimers, see the respective QIAGEN kit handbook or user manual. QIAGEN kit handbooks and user manuals are available at www.qiagen.com or can be requested from QIAGEN Technical Services or your local distributor.

www.qiagen.com

Australia ▪ Orders 1-800-243-800 ▪ Fax 03-9840-9888 ▪ Technical 1-800-243-066

Austria ▪ Orders 0800-28-10-10 ▪ Fax 0800/28-10-19 ▪ Technical 0800-28-10-11

Belgium ▪ Orders 0800-79612 ▪ Fax 0800-79611 ▪ Technical 0800-79556

Brazil ▪ Orders 0800-557779 ▪ Fax 55-11-5079-4001 ▪ Technical 0800-557779

Canada ▪ Orders 800-572-9613 ▪ Fax 800-713-5951 ▪ Technical 800-DNA-PREP (800-362-7737)

China ▪ Orders 86-21-3865-3865 ▪ Fax 86-21-3865-3965 ▪ Technical 800-988-0325

Denmark ▪ Orders 80-885945 ▪ Fax 80-885944 ▪ Technical 80-885942

Finland ▪ Orders 0800-914416 ▪ Fax 0800-914415 ▪ Technical 0800-914413

France ▪ Orders 01-60-920-926 ▪ Fax 01-60-920-925 ▪ Technical 01-60-920-930 ▪ Offers 01-60-920-928

Germany ▪ Orders 02103-29-12000 ▪ Fax 02103-29-22000 ▪ Technical 02103-29-12400

Hong Kong ▪ Orders 800 933 965 ▪ Fax 800 930 439 ▪ Technical 800 930 425

Ireland ▪ Orders 1800 555 049 ▪ Fax 1800 555 048 ▪ Technical 1800 555 061

Italy ▪ Orders 800-789-544 ▪ Fax 02-334304-826 ▪ Technical 800-787980

Japan ▪ Telephone 03-6890-7300 ▪ Fax 03-5547-0818 ▪ Technical 03-6890-7300

Korea (South) ▪ Orders 080-000-7146 ▪ Fax 02-2626-5703 ▪ Technical 080-000-7145

Luxembourg ▪ Orders 8002-2076 ▪ Fax 8002-2073 ▪ Technical 8002-2067

Mexico ▪ Orders 01-800-7742-639 ▪ Fax 01-800-1122-330 ▪ Technical 01-800-7742-639

The Netherlands ▪ Orders 0800-0229592 ▪ Fax 0800-0229593 ▪ Technical 0800-0229602

Norway ▪ Orders 800-18859 ▪ Fax 800-18817 ▪ Technical 800-18712

Singapore ▪ Orders 1800-742-4362 ▪ Fax 65-6854-8184 ▪ Technical 1800-742-4368

Spain ▪ Orders 91-630-7050 ▪ Fax 91-630-5145 ▪ Technical 91-630-7050

Sweden ▪ Orders 020-790282 ▪ Fax 020-790582 ▪ Technical 020-798328

Switzerland ▪ Orders 055-254-22-11 ▪ Fax 055-254-22-13 ▪ Technical 055-254-22-12

UK ▪ Orders 01293-422-911 ▪ Fax 01293-422-922 ▪ Technical 01293-422-999

USA ▪ Orders 800-426-8157 ▪ Fax 800-718-2056 ▪ Technical 800-DNA-PREP (800-362-7737)

QIAGEN

10663018 04/2010

Sample & Assay Technologies
