# Supplementary material for: Screening and identifying of biomarkers in early colorectal cancer and adenoma based on genome-wide methylation profiles
Source: World J Surg Oncol. 2023 Oct 2;21:312. doi: 10.1186/s12957-023-03189-1 (PMC10544418; doi:10.1186/s12957-023-03189-1)
Supplement: Supplementary file 2 — Additional file 2: Supplement 2. EZ DNA Methylation™ Kit. [file 12957_2023_3189_MOESM2_ESM.pdf]

ZYMO RESEARCH
The Beauty of Science is to Make Things Simple

INSTRUCTION MANUAL

EZ DNA Methylation™ Kit

Catalog Nos. **D5001 & D5002**

Highlights

- Streamlined, proven procedure for bisulfite conversion of DNA.
- Desulphonation and recovery of bisulfite-treated DNA with a spin column.
- Recovered DNA is ideal for downstream analyses including PCR, endonuclease digestion, sequencing, microarrays, etc.

Contents

Product Contents 1
Introduction to DNA Methylation 2
Product Description 3
Product Specifications 4
Reagent Preparation 4
Protocol 5
Appendix 6
Frequently Asked Questions 7
Ordering Information 8
List of Related Products 9

For Research Use Only

Ver. 1.2.2

Product Contents:

EZ DNA Methylation™ Kit	**D5001** 50 rxns.	**D5002** 200 rxns.	**Storage Temperature**
**CT Conversion Reagent***	5 tubes	20 tubes	Room Temp.
**M-Dilution Buffer**	1.3 ml	5.2 ml	Room Temp.
**M-Binding Buffer**	20 ml	80 ml	Room Temp.
**M-Wash Buffer****	6 ml	24 ml	Room Temp.
**M-Desulphonation Buffer**	10 ml	40 ml	Room Temp.
**M-Elution Buffer**	1 ml	4 ml	Room Temp.
**Zymo-Spin™ IC Columns**	50 columns	200 columns	Room Temp.
**Collection Tubes**	50 tubes	200 tubes	Room Temp.
**Instruction Manual**	1	1	–

Note - Integrity of kit components is guaranteed for one year from date of purchase. Reagents are routinely tested on a lot-to-lot basis to ensure they provide maximal performance and reliability.

* 750 µl water and 210 µl **M-Dilution Buffer** are added per tube of **CT Conversion Reagent** and mixed prior to use.

** Add 24 ml of 100% ethanol to the 6 ml **M-Wash Buffer** concentrate (D5001) or 96 ml of 100% ethanol to the 24 ml **M-Wash Buffer** concentrate (D5002) before use.

The Polymerase Chain Reaction (PCR) process is covered by U.S. Pat. Nos. 4,683,195 and 4,683,202 assigned to Hoffmann-La Roche. Patents pending in other countries. No license under these patents to use the PCR process is conveyed expressly or by implication to the purchaser by the purchase of Zymo Research's EZ DNA Methylation kits. Further information on purchasing licenses to practice the PCR process can be obtained from the director of Licensing at Applied Biosystems, 850 Lincoln Centre Drive, Foster City, California 94404 or at Roche Molecular Systems, Inc., 1145 Atlantic Avenue, Alameda, California 94501.

Use of Methylation Specific PCR (MSP) is protected by US Patents 5,786,146 & 6,017,704 & 6,200,756 & 6,265,171 and International Patent WO 97/46705. No license under these patents to use the MSP process is conveyed expressly or by implication to the purchaser by the purchase of this product.

Note - ™ Trademarks of Zymo Research Corporation. This product is for research use only and should only be used by trained professionals. Some reagents included with this kit are irritants. Wear protective gloves and eye protection. Follow the safety guidelines and rules enacted by your research institution or facility.

Introduction to DNA Methylation:

DNA methylation is a naturally occurring event in both prokaryotic and eukaryotic organisms. In prokaryotes DNA methylation provides a way to protect host DNA from digestion by restriction endonucleases that are designed to eliminate foreign DNA, and in higher eukaryotes DNA methylation functions in the regulation/control of gene expression (1). It has been demonstrated that aberrant DNA methylation is a widespread phenomenon in cancer and may be among the earliest changes to occur during oncogenesis (2). DNA methylation has also been shown to play a central role in gene imprinting, embryonic development, X-chromosome gene silencing, and cell cycle regulation. In many plants and animals, DNA methylation consists of the addition of a methyl group to the fifth carbon position of the cytosine pyrimidine ring via a methyltransferase enzyme (3). The majority of DNA methylation in mammals occurs in 5'-CpG-3' dinucleotides, but other methylation patterns do exist. In fact, about 80 percent of all 5'-CpG-3' dinucleotides in mammalian genomes are found to be methylated, whereas the majority of the twenty percent that remain unmethylated are within promoters or in the first exons of genes.

The ability to detect and quantify DNA methylation efficiently and accurately has become essential for the study of cancer, gene expression, genetic diseases, as well as many other important aspects of biology. To date, a number of methods have been developed to detect/quantify DNA methylation including: high-performance capillary electrophoresis (4) and methylation-sensitive arbitrarily primed PCR (5). However, the most common technique used today remains the bisulfite conversion method (6). This technique involves treating methylated DNA with bisulfite, which converts unmethylated cytosines into uracil. Methylated cytosines remain unchanged during the treatment. Once converted, the methylation profile of the DNA can be determined by PCR amplification followed by DNA sequencing (see below).

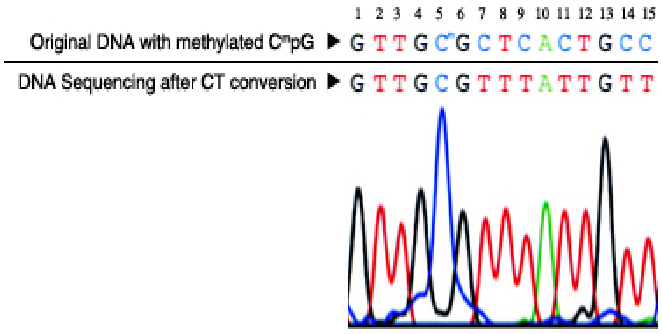

DNA sequencing results following bisulfite treatment. DNA with methylated C^{m}pG at nucleotide position #5 was processed using the **EZ DNA Methylation™ Kit**. The recovered DNA was amplified by PCR and then sequenced directly. The methylated cytosine at position #5 remained intact while the unmethylated cytosines at positions #7, 9, 11, 14 and 15 were completely converted into uracil following bisulfite treatment and detected as thymine following PCR.

References:

1. Costello JF, Plass CJ. Med. Genet. 2001; 38(5): 285-303.

2. Stirzaker C. Cancer Res. 1997; 57(11): 2229-2237.

3. Adams RL. Bioessays. 1995; 17(2): 139-145.

4. Fraga MF, *et al.* Electrophoresis. 2000; 21(14): 2990-2994.

5. Gonzalgo ML. Cancer Res. 1997; 57(4): 594-599.

6. Frommer M. Proc. Natl. Acad. Sci. USA. 1992; 89(5): 1827-1831.

Product Description:

Selected EZ DNA Methylation™ Kit Citations:

1. Ehrich M, *et al.* Nuc. Acids Res. 2007; 35 (5): e29

2. Kaneda M, *et al.* Nature. 2004; 429: 900-903

3. Zhang F, *et al.* Proc. Natl. Acad. Sci. USA. 2007; 104 (11): 4395-4400.

4. Oda M, *et al.* Genes & Dev. 2006; 20: 3382-3394.

5. England RPM, *et al.* Nature Meth. 2005; 2: 1-2.

The **EZ DNA Methylation™ Kit** features a simplified procedure that streamlines bisulfite conversion of DNA. The kit is based on the three-step reaction that takes place between cytosine and sodium bisulfite where cytosine is converted into uracil. The product's innovative in-column desulphonation technology eliminates otherwise cumbersome precipitations. The kit is designed to reduce template degradation, minimize DNA loss during treatment and clean-up, while ensuring complete conversion of the DNA. Purified, converted DNA is ideal for PCR amplification for downstream analyses including endonuclease digestion, sequencing, microarrays, etc.

An outline comparing the **EZ DNA Methylation™ Kit** procedure to Zymo Research's other methylation kits is shown below.

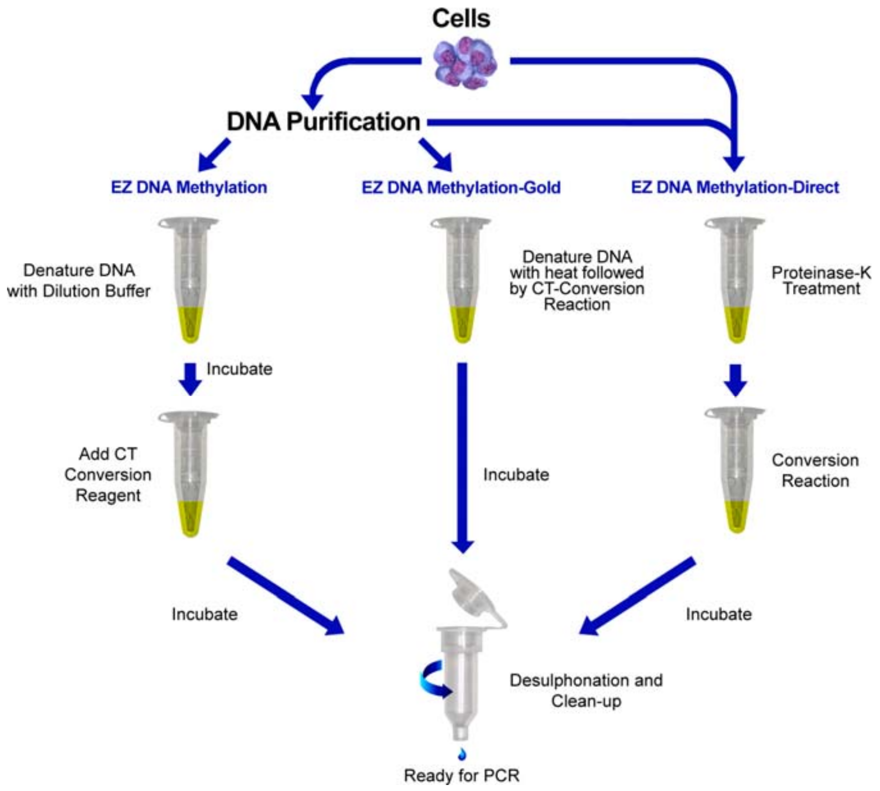

Outline of the **EZ DNA Methylation™**, **EZ DNA Methylation-Gold™** and **EZ DNA Methylation-Direct™ Kit** procedures.

Specifications:

- **DNA Input:** Samples containing 500 pg - 2 µg of DNA. For optimal results, the amount of input DNA should be from 200 to 500 ng.

- **Conversion Efficiency:** > 99% of non-methylated C residues are converted to U; > 99% protection of methylated cytosines.

- **DNA Recovery:** > 80%

Reagent Preparation:

- **Preparation of CT Conversion Reagent**

  The **CT Conversion Reagent** supplied within this kit is a solid mixture and must be prepared prior to first use. Prepare as follows:

  1. Add 750 µl water and 210 µl of **M-Dilution Buffer** to a tube of **CT Conversion Reagent**.

  2. Mix at room temperature with frequent vortexing or shaking for 10 minutes.

     Note: It is normal to see trace amounts of undissolved reagent in the **CT Conversion Reagent**. Each tube of **CT Conversion Reagent** is designed for 10 separate DNA treatments.

     Storage: The **CT Conversion Reagent** is light sensitive, so minimize its exposure to light. For best results, the **CT Conversion Reagent** should be used immediately following preparation. If not used immediately, the **CT Conversion Reagent** solution can be stored overnight at room temperature, one week at 4°C, or up to one month at -20°C. Stored **CT Conversion Reagent** solution must be warmed to 37°C, then vortexed prior to use.

- **Preparation of M-Wash Buffer**

  Add 24 ml of 100% ethanol to the 6 ml **M-Wash Buffer** concentrate (D5001) or 96 ml of 100% ethanol to the 24 ml **M-Wash Buffer** concentrate (D5002) before use.

Protocol:

1. Add 5 µl of **M-Dilution Buffer** to the DNA sample and adjust the total volume to 50 µl with water. Mix the sample by flicking or pipetting up and down.

   Example: For 14 µl of a DNA sample add 5 µl M-Dilution Buffer and 31 µl water.

2. Incubate the sample at 37°C for 15 minutes.

3. After the above incubation, add 100 µl of the prepared **CT Conversion Reagent** to each sample and mix.

The **CT Conversion reagent** is light sensitive, so try to minimize the reaction's exposure to light whenever possible.

4. Incubate the sample in the dark at 50°C for 12 – 16 hours.

   Please see **Appendix** (page 6) for alternative incubation conditions (e.g., when using the Illumina Infinium® Methylation Assay)

5. Incubate the sample at 0 - 4°C (e.g., on ice) for 10 minutes.

The capacity of the collection tube with the column inserted is 800 µl. Empty the collection tube whenever necessary to prevent contamination of the column contents by the flow-through.

6. Add 400 µl of **M-Binding Buffer** to a **Zymo-Spin™ IC Column** and place the column into a provided **Collection Tube**.

7. Load the sample (from Step 5) into the **Zymo-Spin™ IC Column** containing the **M-Binding Buffer**. Close the cap and mix by inverting the column several times.

8. Centrifuge at full speed (≥10,000 x *g*) for 30 seconds. Discard the flow-through.

9. Add 100 µl of **M-Wash Buffer** to the column. Centrifuge at full speed for 30 seconds.

10. Add 200 µl of **M-Desulphonation Buffer** to the column and let stand at room temperature (20°C – 30°C) for 15 - 20 minutes. After the incubation, centrifuge at full speed for 30 seconds.

11. Add 200 µl of **M-Wash Buffer** to the column. Centrifuge at full speed for 30 seconds. Add another 200 µl of **M-Wash Buffer** and centrifuge for an additional 30 seconds.

Alternatively, water or TE (pH ≥ 6.0) can be used for elution if required for your experiments.

12. Place the column into a 1.5 ml microcentrifuge tube. Add 10 µl of **M-Elution Buffer** directly to the column matrix. Centrifuge for 30 seconds at full speed to elute the DNA.

    The DNA is ready for immediate analysis or can be stored at or below -20°C for later use. For long term storage, store at or below -70°C. We recommend using 1 - 4 µl of eluted DNA for each PCR, however, up to 10 µl can be used if necessary. The elution volume can be > 10 µl depending on the requirements of your experiments, but small elution volumes will yield more concentrated DNA.

Appendix: Bisulfite Conversion and PCR Optimization

1. **Incomplete C to T Conversion.**

   A. Increase temperature in Step 2 of the **Protocol** to 42°C and extend the incubation time to 30 minutes. If the problem persists, use modified conversion conditions (see **B,** below).

   B. In Step 1 of the **Protocol**, add 7.5 µl **M-Dilution Buffer** instead of 5 µl (the total volume should remain 50 µl). If this change is made, the preparation of the **CT-Conversion Reagent** must also be modified by reducing the volume of **M-Dilution Buffer** from 210 µl to 185 µl. In Step 3 of the **Protocol**, add 97.5 µl prepared **CT-Conversion Reagent** per reaction instead of 100 µl.

2. **PCR Primer Design.** Generally, primers of 24 to 32 bases are required for amplification of bisulfite converted DNA. For most eukaryotes, all non-methylated cytosine residues will be converted into uracil during the bisulfite treatment. These Cs should be treated as Ts for primer design purposes. For example, for the sequence 5'-AACCTTACAGGCAC-3', the corresponding primer should be 5'-AA**TT**TTA**T**AGG**T**A**T**-3'.

   If the primer contains CpG dinucleotides with uncertain methylation status, then mixed bases with C and T can be used. Usually, there should be no more than three mixed positions per primer and they should be located toward the 5' end of the primer. It is not recommended to have mixed bases located at the 3' end of the primer.

3. **Amount of DNA Required for Bisulfite Conversion.** The minimal amount of human or mouse genomic DNA required for bisulfite treatment and subsequent PCR amplification is 500 pg. The optimal amount of DNA per bisulfite treatment is 200 to 500 ng. Although, up to 2 µg of DNA can also be processed, it should be noted that high input levels of DNA may result in incomplete bisulfite conversion for some GC-rich regions.

4. **PCR Conditions.** Usually, 35 to 40 cycles are required for successful PCR amplification of bisulfite converted DNA. Optimal amplicon size should be between 150 - 300 bp; however larger amplicons (up to 1 kb) can be generated with optimization of the bisulfite reaction and PCR conditions. We have found that annealing temperatures between 55 - 60°C typically work well. As most non-methylated cytosine residues are converted into uracil, the bisulfite-treated DNA usually is AT-rich and has low GC composition. Thus, it may be necessary to reduce the annealing temperature accordingly.

   Non-specific PCR amplification is relatively common with bisulfite treated DNA due to its AT-rich nature. PCR using "hot start" polymerases is strongly recommended for the amplification of bisulfite-treated DNA.

   Zymo*Taq*™ is a "hot start" DNA polymerase specifically designed for the amplification of bisulfite treated DNA. (see page 9 for details)

5. **Quantifying Bisulfite Treated DNA.** Following bisulfite treatment of genomic DNA, non-methylated cytosine residues are converted into uracil. The recovered DNA is typically A, U, and T-rich. The original base-pairing no longer exists. Instead, it is single stranded with limited non-specific base-pairing at room temperature. The absorption coefficient at 260 nm resembles that of RNA. Use a value of 40 µg/ml for Ab_{260} = 1.0 when determining the concentration of the recovered bisulfite-treated DNA.

6. **Alternative Incubation Conditions When Using the Illumina Infinium® Methylation Assay.** For Steps 4 & 5 of the protocol, incubate the sample(s) in a thermocycler at…

   (95 °C for 30 sec., 50 °C for 60 min.) x 16 cycles, then "hold" at 4 °C

Infinium® is a registered trademark of Illumina, Inc.

Frequently Asked Questions:

Q: Should the input DNA be dissolved in TE, water, or some other buffer prior to its conversion?

A: *Water, TE or modified TE buffers can be used to dissolve the DNA and do not interfere with the conversion process.*

Q: At what temperature and for how long can converted DNA be stored?

A: *The sample should be stored at ≤ -20ºC whenever possible. The quality of the DNA should remain relatively unchanged for up to 3 months.*

Q: Which *Taq* polymerase(s) do you recommend for PCR amplification of converted DNA?

A: *We recommend a "hot start" DNA polymerase (e.g., ZymoTaq™, page 9).*

Q: Why are there two different catalog numbers for the EZ-96 DNA Methylation™ Kit?

A: *The two different catalog numbers are used to differentiate between the binding plates that are included in the kit. Deep and shallow-well binding plates are available to accommodate most rotors and microplate carriers. Below is a comparison of the two binding plates.*

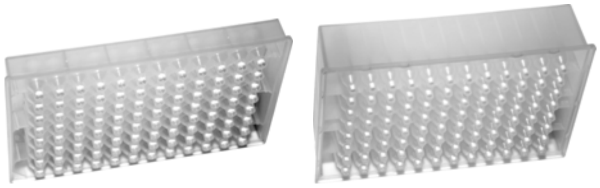

**Binding Plate**	**Silicon-A™ Plate**	**Zymo-Spin™ I-96 Plate**
**Style**	Shallow-Well	Deep-Well
**Height of Binding Plate**	19 mm (0.75 inches)	35 mm (1.38 inches)
**Binding Plate/Collection Plate Assembly**	43 mm (1.69 inches)	60 mm (2.36 inches)
**Binding Cap./Minimum Elution Volume**	5 µg/30 µl	5 µg/15 µl
**Catalog Numbers**	**D5003**	**D5004**

Ordering Information:

Product Description	Catalog No.	Kit Size
**EZ DNA Methylation™ Kit**	D5001	50 rxns.
**EZ DNA Methylation™ Kit**	D5002	200 rxns.
**EZ-96 DNA Methylation™ Kit (Shallow-Well)**	D5003	2 x 96 rxns.
**EZ-96 DNA Methylation™ Kit (Deep-Well)**	D5004	2 x 96 rxns.

For Individual Sale	Catalog No.	Amount(s)
**CT Conversion Reagent**	D5001-1<br>D5003-1	1 tube<br>1 bottle
**M-Dilution Buffer**	D5001-2<br>D5002-2	1.3 ml<br>5.2 ml
**M-Binding Buffer**	D5001-3<br>D5002-3	20 ml<br>80 ml
**M-Wash Buffer**	D5001-4<br>D5002-4<br>D5007-4	6 ml<br>24 ml<br>36 ml
**M-Desulphonation Buffer**	D5001-5<br>D5002-5	10 ml<br>40 ml
**M-Elution Buffer**	D5001-6<br>D5002-6	1 ml<br>4 ml
**Zymo-Spin™ IC Columns** (capped)	C1004-50<br>C1004-250	50 columns<br>250 columns
**Collection Tubes**	C1001-50<br>C1001-500<br>C1001-1000	50 tubes<br>500 tubes<br>1,000 tubes
**Zymo-Spin™ I-96 Binding Plates**	C2004	2 plates
**Silicon-A™ Binding Plates**	C2001	2 plates
**Conversion Plates w/ Pierceable Cover Film**	C2005	2 plates/films
**Collection Plates**	C2002	2 plates
**Elution Plates**	C2003	2 plates

THE Epigenetics COMPANY™

Product	Description	Kit Size	Cat No. (Format)
	Bisulfite Kits for DNA Methylation Detection		
**EZ DNA Methylation™ Kit**	For the conversion of unmethylated cytosines in DNA to uracil via the chemical-denaturation of DNA and a specially designed CT Conversion Reagent. *Fast-Spin* technology ensures ultra-pure, converted DNA for subsequent DNA methylation analysis.	50 Rxns.<br>200 Rxns.<br>2x96 Rxns.<br>2x96 Rxns.	**D5001** (spin column)<br>**D5002** (spin column)<br>**D5003** (shallow-well plate)<br>**D5004** (deep-well plate)
**EZ DNA Methylation-Gold™ Kit**	For the fast (3 hr.) conversion of unmethylated cytosines in DNA to uracil via heat/chemical-denaturation of DNA and a specially designed CT Conversion Reagent. *Fast-Spin* technology ensures ultra-pure, converted DNA for subsequent DNA methylation analysis.	50 Rxns.<br>200 Rxns.<br>2x96 Rxns.<br>2x96 Rxns.	**D5005** (spin column)<br>**D5006** (spin column)<br>**D5007** (shallow-well plate)<br>**D5008** (deep-well plate)
**EZ DNA Methylation-Direct™ Kit**	Features simple and reliable DNA bisulfite conversion directly from blood, tissue (FFPE/LCM), and cells without the prerequisite for DNA purification in as little as 4-6 hrs. The increased sensitivity of this kit makes it possible to amplify bisulfite converted DNA from as few as 10 cells or 50 pg DNA.	50 Rxns.<br>200 Rxns.<br>2x96 Rxns.<br>2x96 Rxns.	**D5020** (spin column)<br>**D5021** (spin column)<br>**D5022** (shallow-well plate)<br>**D5023** (deep-well plate)
**EZ DNA Methylation-Startup™ Kit**	Consolidated product for sample processing, bisulfite treatment of DNA, then PCR amplification of "converted" DNA for methylation analysis. Includes the EZ DNA Methylation-Direct™ Kit, the Universal Methylated DNA Standard, and the Zymo*Taq*™ PreMix.	1 Kit	**D5024**
**EZ Bisulfite DNA Clean-up Kit™**	Desulfonation and purification of DNA from any "homebrew" or commercially derived reaction mixture containing bisulfite.	50 Preps.<br>200 Preps.<br>2x96 Preps.<br>2x96 Preps.	**D5025** (spin column)<br>**D5026** (spin column)<br>**D5027** (shallow-well plate)<br>**D5028** (deep-well plate)
	Methylated DNA Standards		
**Universal Methylated DNA Standard**	pUC19 plasmid DNA having all CpG sites methylated. To be used for the evaluation of bisulfite-mediated conversion of DNA. Supplied with a control primer set.	1 set	**D5010**
**Universal Methylated Human DNA Standard**	Human (male) genomic DNA having all CpG sites methylated. To be used for the evaluation of bisulfite-mediated conversion of DNA. Supplied with a control primer set.	1 set	**D5011**
**Universal Methylated Mouse DNA Standard**	Mouse (male) DNA having all CpG sites methylated. To be used for the evaluation of bisulfite-mediated conversion of DNA. Supplied with a control primer set.	1 set	**D5012**
	Other…		
**ChIP DNA Clean & Concentrator™**	Clean and concentrate DNA from any reaction or "crude" preparation in 2 min. A 6 µl minimum elution volume allows for highly concentrated DNA. Designed for samples containing up to 5 µg of DNA.	50 Preps.<br>50 Preps.	**D5201** (uncapped column)<br>**D5205** (capped column)
**Zymo*Taq*™ DNA Polymerase**	Zymo*Taq*™ "hot start" DNA Polymerase is specifically designed for the amplification of "difficult" DNA templates including: bisulfite-treated DNA for methylation detection. The product generates specific amplicons with little or no by-product formation. Available either as a single buffer premix or as a polymerase system with components provided separately.	50 Rxns.<br>200 Rxns.<br><br>50 Rxns.<br>200 Rxns.	**E2001** (system)<br>**E2002** (system)<br><br>**E2003** (premix)<br>**E2004** (premix)
**Anti-5′-Methylcytosine Monoclonal Antibody (clone 10G4)**	Mouse monoclonal antibody developed to facilitate the differentiation between methylated and non-methylated cytosines in DNA. Can be used in immunoprecipitation-based procedures including Methylated DNA Immunoprecipitation (MeIP).	50 µg/50 µl<br>200 µg/200 µl	**A3001-50**<br>**A3001-200**
