# Supplementary material for: Screening and identifying of biomarkers in early colorectal cancer and adenoma based on genome-wide methylation profiles
Source: World J Surg Oncol. 2023 Oct 2;21:312. doi: 10.1186/s12957-023-03189-1 (PMC10544418; doi:10.1186/s12957-023-03189-1)
Supplement: Supplementary file 3 — Additional file 3: Supplement 3. EpiTect® Bisulfite Handbook. [file 12957_2023_3189_MOESM3_ESM.pdf]

February 2020

EpiTect® Bisulfite Handbook

For complete bisulfite conversion and cleanup of DNA for methylation analysis

Sample to Insight

QIAGEN

Contents

Kit Contents 3
Storage 3
Intended Use 4
Quality Control 4
Safety Information 5
Introduction 6
Principle and procedure 7
Description of protocols 11
Automated purification of DNA on QIAcube Instruments 12
Equipment and Reagents to Be Supplied by User 14
Important Notes 15
Protocol: Sodium Bisulfite Conversion of Unmethylated Cytosines in DNA 17
Protocol: Sodium Bisulfite Conversion of Unmethylated Cytosines in DNA from Low-Concentration Solutions 23
Protocol: Sodium Bisulfite Conversion of Unmethylated Cytosines in DNA Isolated from FFPE Tissue Samples 29
Protocol: Sodium Bisulfite Conversion of Unmethylated Cytosines in Small Amounts of Fragmented DNA 35
Troubleshooting Guide 41
Ordering Information 44
Document Revision History 47

Kit Contents

EpiTect Bisulfite Kit<br>Catalog no.<br>Number of preps	(48)<br>59104<br>48
**Chemical Module**	
Bisulfite Mix (aliquots for 8 reactions)	6
DNA Protect Buffer	1.9 ml
RNase-Free Water	3 x 1.9 ml
**Purification Module**	
EpiTect Spin Columns	48
Collection Tubes (2 ml)	96
Buffer BL*	31 ml
Buffer BW (concentrate)	2 x 13 ml
Buffer BD (concentrate)	3 ml
Buffer EB	15 ml
Carrier RNA	310 µg

* Contains a guanidine salt. Not compatible with disinfectants containing bleach. See "Safety Information" (page 5).

Storage

The EpiTect Bisulfite Kit is shipped at room temperature (15–25°C). Upon arrival, the EpiTect Bisulfite spin columns, DNA Protect Buffer and Buffer BD should be stored at 2–8°C. However, short-term storage (up to 4 weeks) at room temperature does not affect their performance.

All other buffers and the Bisulfite Mix should be stored at room temperature and are stable for at least 6 months under these conditions, if not otherwise stated on the label.

Dissolved Bisulfite Mix can be stored at –30°C to –15°C for up to 4 weeks.

Lyophilized carrier RNA can be stored at room temperature for 1 year. Carrier RNA can only be dissolved in RNase-free water. Dissolved carrier RNA should be immediately added to Buffer BL, as described in "Things to do before starting" in each protocol. This solution should be prepared fresh and is stable at 2–8°C for up to 48 hours. Unused portions of carrier RNA dissolved in RNase-free water should be frozen in aliquots at –30°C to –15°C and can be stored for up to 1 year.

Intended Use

The EpiTect Bisulfite Kit is intended for molecular biology applications. This product is neither intended for the diagnosis, prevention or treatment of a disease, nor has it been validated for such use either alone or in combination with other products. Therefore, the performance characteristics of the product for clinical use (i.e., diagnostic, prognostic, therapeutic or blood banking) are unknown.

QIAcube® Connect is designed to perform fully automated purification of nucleic acids and proteins in molecular biology applications. The system is intended for use by professional users trained in molecular biological techniques and the operation of QIAcube Connect.

All due care and attention should be exercised in the handling of the products. We recommend all users of QIAGEN® products to adhere to the NIH guidelines that have been developed for recombinant DNA experiments or to other applicable guidelines.

Quality Control

In accordance with QIAGEN's ISO-certified Quality Management System, each lot of EpiTect Bisulfite Kits is tested against predetermined specifications to ensure consistent product quality.

Safety Information

When working with chemicals, always wear a suitable lab coat, disposable gloves and protective goggles. For more information, please consult the appropriate safety data sheets (SDSs). These are available online in convenient and compact PDF format at **www.qiagen.com/safety** where you can find, view and print the SDS for each QIAGEN kit and kit component.

CAUTION: DO NOT add bleach or acidic solutions directly to waste containing Buffer BL.

Buffer BL contains a guanidine salt, which can form highly reactive compounds when combined with bleach. If liquid containing this buffer is spilt, clean with suitable laboratory detergent and water.

Introduction

Epigenetics describes the study of heritable changes in gene function that occur without a change in the nuclear DNA sequence. In addition to RNA-associated silencing and histone modification, a major epigenetic mechanism in higher-order eukaryotes is DNA methylation.

Methylation of DNA occurs on cytosine residues, especially on CpG dinucleotides enriched in small regions of DNA (<500 bp). These regions, with a GC content greater than 55%, are known as CpG islands. They are usually clustered around the regulatory region of genes and can affect the transcriptional regulation of these genes. Methylation of CpG islands by DNA methylases has been shown to be associated with gene inactivation and plays an important role in the development of cancer and cell aging. Reversal of DNA methylation at these sites is a potential therapeutic strategy as this reversal may restore expression of transcriptionally silenced genes. In addition to CpG, methylated cytosine residues are also found at CpNpG or CpNpN sites (N = A, T or C) in plants.

The methylation status of a DNA sequence can best be determined using sodium bisulfite. Incubation of the target DNA with sodium bisulfite results in conversion of unmethylated cytosine residues into uracil, leaving the methylated cytosines unchanged. Therefore, bisulfite treatment gives rise to different DNA sequences for methylated and unmethylated DNA (see below).

	**Original sequence**	**After bisulfite treatment**
Unmethylated DNA	N-C-G-N-C-G-N-C-G-N	N-U-G-N-U-G-N-U-G-N
Methylated DNA	N-C-G-N-C-G-N-C-G-N	N-C-G-N-C-G-N-C-G-N

The most critical step for correct determination of a methylation pattern is the complete conversion of unmethylated cytosines. This is achieved by incubating the DNA in high bisulfite salt concentrations at high temperature and low pH. These harsh conditions usually lead to a

high degree of DNA fragmentation and subsequent loss of DNA during purification. Purification is necessary to remove bisulfite salts and chemicals used in the conversion process that inhibit sequencing procedures. Common bisulfite procedures usually require high amounts of input DNA. However, due to DNA degradation during conversion and DNA loss during purification, such procedures often lead to low DNA yield, highly fragmented DNA and irreproducible conversion rates.

The EpiTect Bisulfite Kit now provides a fast and streamlined 6-hour procedure for efficient conversion and purification of as little as 1 ng of DNA. DNA fragmentation is prevented during the bisulfite conversion reaction by the unique DNA Protect Buffer, which contains a pH-indicator dye as a mixing control in reaction setup, allowing confirmation of the correct pH for cytosine conversion.

Furthermore, the bisulfite thermal cycling program provides an optimized series of incubation steps necessary for thermal DNA denaturation and subsequent sulfonation and cytosine deamination, enabling high cytosine conversion rates of over 99%. Desulfonation, the final step in chemical conversion of cytosines, is achieved by a convenient on-column step included in the purification procedure.

Principle and procedure

The EpiTect Bisulfite procedure comprises a few simple steps: bisulfite-mediated conversion of unmethylated cytosines; binding of the converted single-stranded DNA to the membrane of an EpiTect spin column; washing; desulfonation of membrane-bound DNA; washing of the membrane-bound DNA to remove desulfonation agent; and elution of the pure, converted DNA from the spin column. The eluted DNA is suited for all techniques currently used for the analysis of DNA methylation, including PCR, real-time PCR, MSP-PCR, bisulfite sequencing (direct und cloning), COBRA and Pyrosequencing®.

Note: The protocols "Protocol: Sodium Bisulfite Conversion of Unmethylated Cytosines in DNA Isolated from FFPE Tissue Samples", page 29, and "Protocol: Sodium Bisulfite Conversion of

Unmethylated Cytosines in Small Amounts of Fragmented DNA", page 35, include an additional step designed to enhance binding of DNA.

Automated purification of bisulfite-converted DNA is possible on QIAcube Connect (see "Automated purification of DNA on QIAcube Instruments", page 12). Twelve samples are processed simultaneously, and protocols can easily be downloaded from **www.qiagen.com/qiacubeprotocols**.

Bisulfite Mix

The Bisulfite Mix is conveniently provided in separate aliquots (8 conversion reactions per aliquot). The sodium bisulfite in each aliquot is supplied in a unique formulation that provides the optimal pH for complete conversion of cytosine to uracil, without the need for tedious pH adjustment. The Bisulfite Mix must be dissolved in 800 µl RNase-free water before use (see "Things to do before starting", pages 17, 23, 29 or 35). Dissolved Bisulfite Mix can be stored at –30°C to –15°C for up to 4 weeks.

EpiTect Bisulfite Conversion Procedure

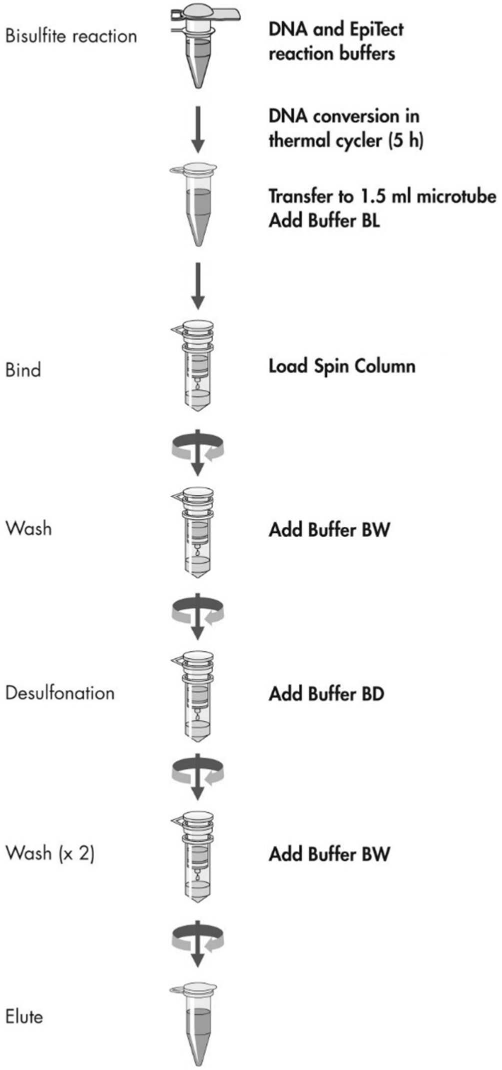

DNA Protect Buffer

DNA Protect Buffer is uniquely formulated to prevent the fragmentation usually associated with bisulfite treatment of DNA at high temperatures and low pH values. It also provides effective DNA denaturation, resulting in the single-stranded DNA necessary for complete cytosine conversion. In addition, DNA Protect Buffer contains a pH indicator dye as a mixing control and to allow confirmation of the correct pH for cytosine conversion.

Bisulfite thermal cycling

The thermal cycling program provides an optimized series of incubation steps necessary for thermal DNA denaturation and subsequent sulfonation and cytosine deamination, enabling highest cytosine conversion rates.

Carrier RNA

Carrier RNA is provided to enhance the binding of small quantities of DNA to the EpiTect spin-column membrane. If using more than 100 ng genomic DNA template, it is not necessary to use carrier RNA, though we strongly recommend its use when processing fragmented DNA or DNA isolated from formalin-fixed, paraffin-embedded (FFPE) tissues. Carrier RNA should be dissolved in RNase-free water before use (see "Things to do before starting", pages 17, 23, 29 or 35).

Optimized buffers

The EpiTect Bisulfite Kit contains carefully optimized buffers enabling maximum cytosine conversion and subsequent DNA purification. Buffer BL promotes binding of the converted single-stranded DNA to the EpiTect spin column membrane. Subsequently, the membrane-bound DNA is washed using Buffer BW, which efficiently removes residual sodium bisulfite. After desulfonation using Buffer BD, the DNA is further desalted using Buffer BW before elution from the spin-column membrane using Buffer EB.

Storage stability of converted and purified DNA

DNA converted and purified using the EpiTect Bisulfite Kit can be stored at –30°C to –15°C for at least 3 years without decrease in quality or conversion. Further investigations into long-term storage are ongoing. Contact QIAGEN for more information.

Description of protocols

The EpiTect Bisulfite Kit is suited for a wide range of DNA starting amounts. The standard protocol "Sodium Bisulfite Conversion of Unmethylated Cytosines in DNA", on page 17, can be used for conversion of 1 ng – 2 µg DNA.

The protocol "Sodium Bisulfite Conversion of Unmethylated Cytosines in DNA from Low-Concentration Solutions", on page 23, is intended for the efficient conversion of very small amounts of DNA from limited and/or precious samples, such as low concentrations of DNA from microdissected biopsies or freely circulating DNA. This protocol is optimized for the conversion of 1–500 ng DNA in a volume of up to 40 µl.

The protocol "Sodium Bisulfite Conversion of Unmethylated Cytosines in DNA Isolated from FFPE Tissue Samples", on page 29, is designed for conversion of DNA from formalin-fixed, paraffin-embedded (FFPE) tissues and includes an optimized step to facilitate binding of DNA. This protocol can be used with 1 ng – 2 µg DNA.

The protocol "Sodium Bisulfite Conversion of Unmethylated Cytosines in Small Amounts of Fragmented DNA", on page 35, is intended for the conversion of small amounts of DNA that might also be fragmented. With an optimized step to facilitate binding of DNA, this protocol can be used with less than 500 pg DNA in a volume of up to 40 µl.

All protocols achieve the same cytosine conversion rates and lead to equal DNA recoveries after purification of converted DNA, independent of DNA starting amounts.

QIAamp® and DNeasy® Kits enable purification of high-quality genomic DNA from a variety of sample types (including blood, tissue, body fluids and FFPE tissues) that is highly suited for cytosine conversion using the EpiTect Bisulfite Kit. See ordering information, which starts on page 44.

Automated purification of DNA on QIAcube Instruments

Purification of DNA can be fully automated on QIAcube Connect or the classic QIAcube. The innovative QIAcube instruments use advanced technology to process QIAGEN spin columns, enabling seamless integration of automated, low-throughput sample prep into your laboratory workflow. Sample preparation using QIAcube instruments follows the same steps as the manual procedure (i.e., lyse, bind, wash and elute), enabling you to continue using the EpiTect Bisulfite Kit for purification of high-quality DNA.

QIAcube instruments are preinstalled with protocols for purification of plasmid DNA, genomic DNA, RNA, viral nucleic acids and proteins, plus DNA and RNA cleanup. The range of protocols available is continually expanding, and additional QIAGEN protocols can be downloaded free of charge at **www.qiagen.com/qiacubeprotocols**.

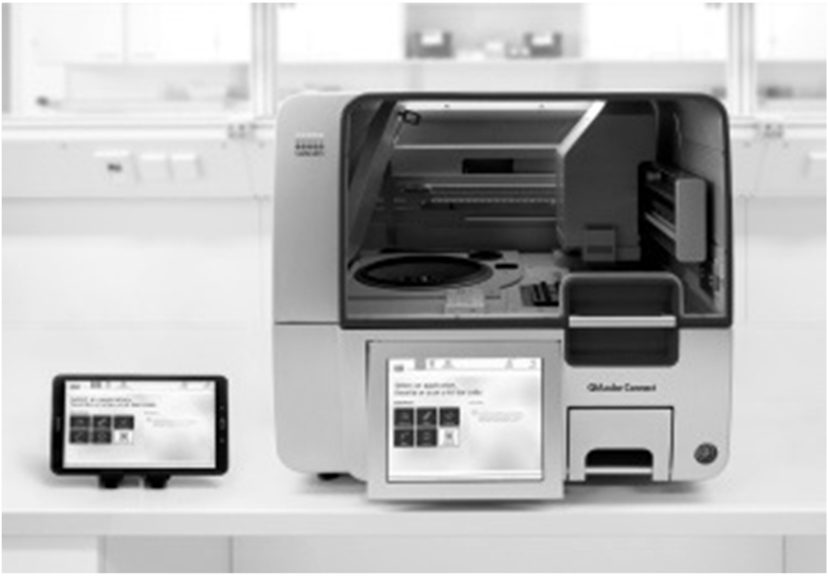

QIAcube Connect.

Equipment and Reagents to Be Supplied by User

When working with chemicals, always wear a suitable lab coat, disposable gloves and protective goggles. For more information, consult the appropriate safety data sheets (SDSs), available from the product supplier.

- Ethanol (molecular biology grade, 96–100%)*
- Pipets and pipet tips (we recommend pipet tips with aerosol barriers for preventing cross-contamination)
- PCR tubes
- Thermal cycler with heated lid (since the bisulfite reaction is not overlaid with mineral oil, only thermal cyclers with heated lids are suitable for this procedure)
- 1.5 ml microcentrifuge tubes for elution steps (available from Brinkmann [Safe-Lock, cat. no. 022363204], Eppendorf® [Safe-Lock, cat. no. 0030 120.086 or Sarstedt [Safety Cap, cat. no. 72.690])†
- Microcentrifuge
- **Optional**: Heating block, thermomixer or heated orbital incubator (see step 1 of each protocol)

* Do not use denatured alcohol, which contains other substances, such as methanol or methylethylketone.

† This is not a complete list of suppliers and does not include many important vendors of biological supplies.

Important Notes

Yield and size of DNA

The yield of DNA purified after bisulfite conversion depends on the amount of DNA and source of the starting material.

Using the standard protocol, the EpiTect Bisulfite Kit is suited for DNA input amounts ranging from 1 ng to 2 µg, with high levels of DNA recovery throughout this range.

The size of the template DNA can vary between 500 bp (in laser microdissections) and 30 kb (fresh samples or blood). DNA purified from serum, urine or FFPE tissue may be <500 bp in length.

If purifying bisulfite-treated DNA originating from very small sample amounts or that is very fragmented (e.g., from biopsies or FFPE tissues), we strongly recommend adding carrier RNA to Buffer BL (see "Things to do before starting", pages 17, 23, 29 or 35).

Note: The purified sample will contain considerably more carrier RNA than DNA. Carrier RNA does not influence downstream applications.

Starting material

The bisulfite conversion also depends on the nature of DNA used as starting material. Genomic DNA should be used for bisulfite treatment without any previous restriction digest step.

If working with plasmid DNA, please linearize the DNA first due to the very quick reannealing of the single-stranded DNA after the denaturation step.

When working with fragmented DNA or already restriction digested DNA we recommend using the protocol "Sodium Bisulfite Conversion of Unmethylated Cytosines in Small Amounts of Fragmented DNA", on page 35.

Handling of EpiTect spin columns

Due to the sensitivity of nucleic acid amplification technologies, the following precautions are necessary when handling EpiTect spin columns to avoid cross-contamination between sample preps:

- Carefully pipet the sample or solution into the EpiTect spin column without wetting the rim of the column. Avoid touching the EpiTect spin column membrane with the pipet tip.
- Always change pipet tips between liquid transfers. We recommend the use of aerosol-barrier pipet tips.
- Open one EpiTect spin column at a time, and take care to avoid generating aerosols.
- Wear gloves throughout the entire procedure. In case of contact between gloves and sample, change gloves immediately.

Centrifugation

EpiTect spin columns will fit into most standard 1.5–2 ml microcentrifuge tubes. A set of 2 ml collection tubes is supplied for the dry centrifugation step. If required, additional 2 ml collection tubes can be purchased separately (cat. no. 19201).

All centrifugation steps should be carried out at room temperature (15–25°C).

Processing EpiTect spin columns in a microcentrifuge

- Always close EpiTect spin columns before placing them in the microcentrifuge.
- For efficient parallel processing of multiple samples, we recommend filling a rack with the collection tubes into which EpiTect spin columns can be transferred after centrifugation. Collection tubes can be used several times.

Protocol: Sodium Bisulfite Conversion of Unmethylated Cytosines in DNA

DNA amounts of 1 ng – 2 µg in a volume of up to 20 µl can be processed using this standard protocol.

Important points before starting

- Each aliquot of Bisulfite Mix is sufficient for 8 conversion reactions. If converting fewer than 8 DNA samples, dissolved Bisulfite Mix can be stored at –30°C to –15°C for up to 4 weeks without any loss of performance.
- DNA Protect Buffer should turn from green to blue after addition to DNA–Bisulfite Mix (step 2), indicating sufficient mixing and correct pH for the bisulfite conversion reaction.
- Perform all centrifugation steps at room temperature (15–25°C).

Things to do before starting

- Add 30 ml ethanol (96–100%) to Buffer BW and store at room temperature (15–25°C). Invert the bottle several times before starting the procedure.
- Add 27 ml ethanol (96–100%) to Buffer BD and store at 2–8°C. Invert the bottle several times before starting the procedure and make sure to close the bottle immediately after use. White precipitates may form in the
  Buffer BD–ethanol mix after some storage time. These precipitates will not affect the performance of Buffer BD. However, avoid transferring precipitates to the EpiTect spin column.

- Add 310 µl RNase-free water to the lyophilized carrier RNA (310 µg) to obtain a 1 µg/µl solution. Dissolve the carrier RNA thoroughly by vortexing. When processing 48 samples at once, add the complete volume of dissolved carrier RNA to the bottle of Buffer BL, and check the box on the bottle lid label. If processing fewer samples, split the dissolved carrier RNA into conveniently sized aliquots (e.g., 50 µl) and store at –30°C to –15°C. Aliquots can be stored for up to 1 year. If fewer than 48 conversions will be performed in a 2-week period, then only make up enough Buffer BL–carrier RNA solution as required (see Table 1, page 18 for example volumes). Carrier RNA enhances binding of DNA to the EpiTect spin-column membrane, especially if there are very few target molecules in the sample. Carrier RNA is not necessary if >100 ng DNA is used.
- Add dissolved carrier RNA to Buffer BL. Calculate the volume of Buffer BL and dissolved carrier RNA required for the number of samples to be processed (see Table 1 for example volumes). If Buffer BL contains precipitates, dissolve by heating (maximum 70°C) with gentle agitation.
- **Optional**: Set a thermomixer, heating block or heated orbital incubator to 60°C for use in step 1.

Table 1. Carrier RNA and Buffer BL volumes

Number of samples	1	4	8	16	24	48
Volume of Buffer BL*	620 µl	2.5 ml	5 ml	10 ml	15 ml	31 ml
Volume of carrier RNA solution†	6.2 µl	25 µl	50 µl	100 µl	150 µl	310 µl

* The volumes given contain a 10% surplus for pipetting inaccuracies.

† Resulting in a final concentration of 10 µg/ml carrier RNA in Buffer BL.

Procedure

Bisulfite DNA conversion

1. Thaw DNA to be used in the bisulfite reactions. Dissolve the required number of aliquots of Bisulfite Mix by adding 800 µl RNase-free water to each aliquot. Vortex until the Bisulfite Mix is completely dissolved. This can take up to 5 min.

   Note: If necessary, heat the Bisulfite Mix–RNase-free water solution to 60°C and vortex again.

   Note: Do not place dissolved Bisulfite Mix on ice.

2. Prepare the bisulfite reactions in 200 µl PCR tubes according to Table 2, page 19. Add each component in the order listed.

   Note: The combined volume of DNA solution and RNase-free water must total 20 µl.

Table 2. Bisulfite reaction components

Component	Volume per reaction (µl)
DNA solution (1 ng – 2 µg)	Variable* (maximum 20 µl)
RNase-free water	Variable*
Bisulfite Mix (dissolved), see step 1	85
DNA Protect Buffer	35
Total volume	140

* The combined volume of DNA solution and RNase-free water must total 20 µl.

3. Close the PCR tubes and mix the bisulfite reactions thoroughly. Store the tubes at room temperature (15–25°C).

   Note: DNA Protect Buffer should turn from green to blue after addition to DNA–Bisulfite Mix, indicating sufficient mixing and correct pH for the bisulfite conversion reaction.

4. Perform the bisulfite DNA conversion using a thermal cycler. Program the thermal cycler according to Table 3.

   The complete cycle should take approximately 5 h.

   Note: If using a thermal cycler that does not allow you to enter the reaction volume 40 µl), set the instrument to the largest volume setting available.

Table 3. Bisulfite conversion thermal cycler conditions

Step	Time	Temperature
Denaturation	5 min	95°C
Incubation	25 min	60°C
Denaturation	5 min	95°C
Incubation	85 min (1 h 25 min)	60°C
Denaturation	5 min	95°C
Incubation	175 min (2 h 55 min)	60°C
Hold	Indefinite†	20°C

† Converted DNA can be left in the thermal cycler overnight without any loss of performance.

5. Place the PCR tubes containing the bisulfite reactions into the thermal cycler. Start the thermal cycling incubation.

   IMPORTANT: Since the bisulfite reaction is not overlaid with mineral oil, only thermal cyclers with heated lids are suitable for this procedure. It is important to use PCR tubes that close tightly.

   Converted DNA can be left in the thermal cycler overnight without any loss of performance.

Cleanup of bisulfite converted DNA

6. Once the bisulfite conversion is complete, briefly centrifuge the PCR tubes containing the bisulfite reactions, and then transfer the complete bisulfite reactions to clean 1.5 ml microcentrifuge tubes.

   Transfer of precipitates in the solution will not affect the performance or yield of the reaction.
7. Add 560 µl freshly prepared Buffer BL containing 10 µg/ml carrier RNA (see "Things to do before starting", page 17) to each sample. Mix the solutions by vortexing and then centrifuge briefly.

   Note: Carrier RNA is not necessary when using >100 ng DNA.
8. Place the necessary number of EpiTect spin columns and collection tubes in a suitable rack. Transfer the entire mixture from each tube in step 7 into the corresponding EpiTect spin column.
9. Centrifuge the spin columns at maximum speed for 1 min. Discard the flow-through, and place the spin columns back into the collection tubes.
10. Add 500 µl Buffer BW to each spin column, and centrifuge at maximum speed for 1 min. Discard the flow-through, and place the spin columns back into the collection tubes.
11. Add 500 µl Buffer BD to each spin column, and incubate for 15 min at room temperature (15–25°C).

    If there are precipitates in Buffer BD, avoid transferring them to the spin columns.

    IMPORTANT: The bottle containing Buffer BD should be closed immediately after use to avoid acidification from carbon dioxide in the air.

    Note: It is important to close the lids of the spin columns before incubation.
12. Centrifuge the spin columns at maximum speed for 1 min. Discard the flow-through, and place the spin columns back into the collection tubes.

13. Add 500 µl Buffer BW to each spin column and centrifuge at maximum speed for 1 min. Discard the flow-through and place the spin columns back into the collection tubes.
14. Repeat step 13 once.
15. Place the spin columns into new 2 ml collection tubes, and centrifuge the spin columns at maximum speed for 1 min to remove any residual liquid.
16. **Recommended**: Place the spin columns with open lids into clean 1.5 ml microcentrifuge tubes (not provided) and incubate the spin columns for 5 min at 56°C in a heating block.

    This step enables evaporation of any remaining liquid.
17. Place the spin columns into clean 1.5 ml microcentrifuge tubes (not provided). Dispense 20 µl Buffer EB onto the center of each membrane. Elute the purified DNA by centrifugation for 1 min at approximately 15,000 x *g* (12,000 rpm).

    Note: To increase the yield of DNA in the eluate, dispense an additional 20 µl Buffer EB to the center of each membrane, and centrifuge for 1 min at maximum speed.

    Note: If the purified DNA is to be stored for up to 24 h, we recommend storage at 2–8°C. For storage longer than 24 h, we recommend storage at –30°C to –15°C. At –30°C to –15°C, DNA converted and purified using the EpiTect Bisulfite Kit can be stored for at least 3 years* without decrease of quality or conversion.

* Investigations into longer storage of converted DNA are ongoing. Contact QIAGEN for more information.

Protocol: Sodium Bisulfite Conversion of Unmethylated Cytosines in DNA from Low-Concentration Solutions

This protocol is optimized for low concentrations of DNA, which enables use of larger input volumes. With this protocol, 1–500 ng DNA in a maximum volume of 40 µl can be processed.

Important points before starting

- Each aliquot of Bisulfite Mix is sufficient for 8 conversion reactions. If converting fewer than 8 DNA samples, reconstituted Bisulfite Mix can be stored at –30°C to –15°C for up to 4 weeks without any loss of performance.
- DNA Protect Buffer should turn from green to blue after addition to DNA–Bisulfite Mix (step 2), indicating sufficient mixing and correct pH for the bisulfite conversion reaction.
- Perform all centrifugation steps at room temperature (15–25°C).

Things to do before starting

- Add 30 ml ethanol (96–100%) to Buffer BW and store at room temperature (15–25°C). Invert the bottle several times before starting the procedure.
- Add 27 ml ethanol (96–100%) to Buffer BD and store at 2–8°C. Invert the bottle several times before starting the procedure and make sure to close the bottle immediately after use. White precipitates may form in the Buffer BD–ethanol mix after some storage time. These precipitates will not affect the performance of Buffer BD. However, avoid transferring precipitates to the EpiTect spin column.

- Add 310 µl RNase-free water to the lyophilized carrier RNA (310 µg) to obtain a 1 µg/µl solution. Dissolve the carrier RNA thoroughly by vortexing. When processing 48 samples at once, add the complete volume of dissolved carrier RNA to the bottle of Buffer BL, and check the box on the bottle lid label. If processing fewer samples, split the dissolved carrier RNA into conveniently sized aliquots (e.g., 50 µl) and store at –30°C to –15°C. Aliquots can be stored for up to 1 year. If fewer than 48 conversions will be performed in a 2-week period, then only make up enough Buffer BL–carrier RNA solution as required (see Table 1, page 18 for example volumes). Carrier RNA enhances binding of DNA to the EpiTect spin-column membrane, especially if there are very few target molecules in the sample. Carrier RNA is not necessary if >100 ng DNA is used.
- Add dissolved carrier RNA to Buffer BL. Calculate the volume of Buffer BL and dissolved carrier RNA required for the number of samples to be processed (see Table 1, page 18, for example volumes). If Buffer BL contains precipitates, dissolve by heating (maximum 70°C) with gentle agitation.
- Equilibrate samples and buffers to room temperature.
- **Optional**: Set a thermomixer, heating block or heated orbital incubator to 60°C for use in step 1.

Procedure

Bisulfite DNA conversion

1. Thaw DNA to be used in the bisulfite reactions. Dissolve the required number of Bisulfite Mix aliquots by adding 800 µl RNase-free water to each aliquot. Vortex until the Bisulfite Mix is completely dissolved. This can take up to 5 min.

   Note: If necessary, heat the Bisulfite Mix–RNase-free water solution to 60°C and vortex again.

   Note: Do not place dissolved Bisulfite Mix on ice.

2. Prepare the bisulfite reactions in 200 µl PCR tubes according to Table 4. Add each component in the order listed.

Note: The combined volume of DNA solution and RNase-free water must total 40 µl.

Table 4. Bisulfite reaction components

Component	Volume per reaction (µl)
DNA solution (1–500 ng)	Variable* (maximum 40 µl)
RNase-free water	Variable*
Bisulfite Mix (dissolved), see step 1	85
DNA Protect Buffer	35
Total volume	140

* The combined volume of DNA solution and RNase-free water must total 40 µl.

3. Close the PCR tubes and mix the bisulfite reactions thoroughly. Store tubes at room temperature (15–25°C).

   Note: DNA Protect Buffer should turn from green to blue after addition to DNA–Bisulfite Mix, indicating sufficient mixing and correct pH for the bisulfite conversion reaction.

4. Perform the bisulfite DNA conversion using a thermal cycler. Program the thermal cycler according to Table 5.

   The complete cycle should take approximately 5 h.

   Note: If using a thermal cycler that does not allow you to enter the reaction volume (140 µl), set the instrument to the largest volume setting available.

Table 5. Bisulfite conversion thermal cycler conditions

Step	Time	Temperature
Denaturation	5 min	95°C
Incubation	25 min	60°C
Denaturation	5 min	95°C
Incubation	85 min (1 h 25 min)	60°C
Denaturation	5 min	95°C
Incubation	175 min (2 h 55 min)	60°C
Hold	Indefinite†	20°C

† Converted DNA can be left in the thermal cycler overnight without any loss of performance.

5. Place the PCR tubes containing the bisulfite reactions into the thermal cycler. Start the thermal cycling incubation.

   IMPORTANT: Since the bisulfite reaction is not overlaid with mineral oil, only thermal cyclers with heated lids are suitable for this procedure.

   Converted DNA can be left in the thermal cycler overnight without any loss of performance.

Cleanup of bisulfite converted DNA

6. Once the bisulfite conversion is complete, briefly centrifuge the PCR tubes containing the bisulfite reactions, and then transfer the complete bisulfite reactions to clean 1.5 ml microcentrifuge tubes.

   Transfer of precipitates in the solution will not affect the performance or yield of the reaction.

7. Add 560 µl freshly prepared Buffer BL containing 10 µg/ml carrier RNA (see "Things to do before starting", page 23) to each sample. Mix the solutions by vortexing and then centrifuge briefly.

   Note: Carrier RNA is not necessary when using >100 ng DNA.

8. Place the necessary number of EpiTect spin columns and collection tubes in a suitable rack. Transfer the entire mixture from the tubes in step 7 into the corresponding EpiTect spin columns.
9. Centrifuge the spin columns at maximum speed for 1 min. Discard the flow-through, and place the spin columns back into the collection tubes.
10. Add 500 µl Buffer BW (wash buffer) to each spin column, and centrifuge at maximum speed for 1 min. Discard the flow-through, and place the spin columns back into the collection tubes.
11. Add 500 µl Buffer BD (desulfonation buffer) to each spin column, and incubate for 15 min at room temperature (15–25°C).

    If there are precipitates in Buffer BD, avoid transferring them to the spin columns.

    IMPORTANT: The bottle containing Buffer BD should be closed immediately after use to avoid acidification from carbon dioxide in the air.

    Note: It is important to close the lids of the spin columns before incubation.

12. Centrifuge the spin columns at maximum speed for 1 min. Discard the flow-through, and place the spin columns back into the collection tubes.
13. Add 500 µl Buffer BW to each spin column and centrifuge at maximum speed for 1 min. Discard the flow-through, and place the spin columns back into the collection tubes.
14. Repeat step 13 once.
15. Place the spin columns into new 2 ml collection tubes, and centrifuge the spin columns at maximum speed for 1 min to remove any residual liquid.

16. **Recommended**: Place the spin columns with open lids into clean 1.5 ml microcentrifuge tubes (not provided), and incubate the columns for 5 min at 56°C in a heating block.

    This step enables evaporation of any remaining liquid.

17. Place the spin columns into clean 1.5 ml microcentrifuge tubes (not provided). Dispense 20 µl Buffer EB onto the center of each membrane. Elute the purified DNA by centrifugation for 1 min at approximately 15,000 x *g* (12,000 rpm.)

    Note: To increase the yield of DNA in the eluate, dispense an additional 20 µl Buffer EB onto the center of each membrane, and centrifuge for 1 min at maximum speed.

    Note: If the purified DNA is to be stored for up to 24 h, we recommend storage at 2–8°C. For storage longer than 24 h, we recommend storage at –30°C to –15°C. At –30°C to –15°C, DNA converted and purified using the EpiTect Bisulfite Kit can be stored for at least 3 years* without decrease of quality or conversion.

* Investigations into longer storage of converted DNA are ongoing. Contact QIAGEN for more information.

Protocol: Sodium Bisulfite Conversion of Unmethylated Cytosines in DNA Isolated from FFPE Tissue Samples

This protocol is designed for processing DNA isolated from formalin-fixed, paraffin-embedded (FFPE) tissue samples (e.g., using QIAamp, DNeasy or EZ1® Kits). An optimized binding step in the cleanup stage facilitates binding of DNA from fixed tissues. Using this protocol, 1 ng – 2 µg DNA in a volume of up to 20 µl can be processed.

Important points before starting

- Each aliquot of Bisulfite Mix is sufficient for 8 conversion reactions. If converting fewer than 8 DNA samples, reconstituted Bisulfite Mix can be stored at –30°C to –15°C for up to 4 weeks without any loss of performance.
- DNA Protect Buffer should turn from green to blue after addition to DNA–Bisulfite Mix (step 2), indicating sufficient mixing and correct pH for the bisulfite conversion reaction.
- Perform all centrifugation steps at room temperature (15–25°C).

Things to do before starting

- Add 30 ml ethanol (96–100%) to Buffer BW and store at room temperature (15–25°C). Invert the bottle several times before starting the procedure.
- Add 27 ml ethanol (96–100%) to Buffer BD and store at 2–8°C. Invert the bottle several times before starting the procedure and make sure to close the bottle immediately after use. White precipitates may form in the Buffer BD–ethanol mix after some storage time. These precipitates will not affect the performance of Buffer BD. However, avoid transferring precipitates to the EpiTect spin column.

- Add 310 µl RNase-free water to the lyophilized carrier RNA (310 µg) to obtain a 1 µg/µl solution. Dissolve the carrier RNA thoroughly by vortexing. When processing 48 samples at once, add the complete volume of dissolved carrier RNA to the bottle of Buffer BL, and check the box on the bottle lid label. If processing fewer samples, split the dissolved carrier RNA into conveniently sized aliquots (e.g., 50 µl) and store at –30°C to –15°C. Aliquots can be stored for up to 1 year. If fewer than 48 conversions will be performed in a 2-week period, then only make up enough Buffer BL–carrier RNA solution as required (see Table 1, page 18 for example volumes). Carrier RNA enhances binding of DNA to the EpiTect spin-column membrane, especially if there are very few target molecules in the sample.
- Add dissolved carrier RNA to Buffer BL. Calculate the volume of Buffer BL and dissolved carrier RNA required for the number of samples to be processed (see Table 1, page 18, for example volumes). If Buffer BL contains precipitates, dissolve by heating (maximum 70°C) with gentle agitation.
- Equilibrate samples and buffers to room temperature.
- **Optional**: Set a thermomixer, heating block or heated orbital incubator to 60°C for use in step 1.

Procedure

Bisulfite DNA conversion

1. Thaw DNA to be used in the bisulfite reactions. Dissolve the required number of Bisulfite Mix aliquots by adding 800 µl RNase-free water to each aliquot. Vortex until the Bisulfite Mix is completely dissolved. This can take up to 5 min.

   Note: If necessary, heat the Bisulfite Mix–RNase-free water solution to 60°C and vortex again.

   Note: Do not place dissolved Bisulfite Mix on ice.

2. Prepare the bisulfite reactions in 200 µl PCR tubes according to Table 6. Add each component in the order listed.

   Note: The combined volume of DNA solution and RNase-free water must total 20 µl.

Table 6. Bisulfite reaction components

Component	Volume per reaction (µl)
DNA solution (1 ng – 2 µg)	Variable* (maximum 20 µl)
RNase-free water	Variable*
Bisulfite Mix (dissolved), see step 1	85
DNA Protect Buffer	35
Total volume	140

* The combined volume of DNA solution and RNase-free water must total 20 µl.

3. Close the PCR tubes and mix the bisulfite reactions thoroughly. Store tubes at room temperature (15–25°C).

   Note: DNA Protect Buffer should turn from green to blue after addition to DNA–Bisulfite Mix, indicating sufficient mixing and correct pH for the bisulfite conversion reaction.

4. Perform the bisulfite DNA conversion using a thermal cycler. Program the thermal cycler according to Table 7.

   The complete cycle should take approximately 5 h.

   Note: If using a thermal cycler that does not allow you to enter the reaction volume (140 µl), set the instrument to the largest volume setting available.

Table 7. Bisulfite conversion thermal cycler conditions

Step	Time	Temperature
Denaturation	5 min	95°C
Incubation	25 min	60°C
Denaturation	5 min	95°C
Incubation	85 min (1 h 25 min)	60°C
Denaturation	5 min	95°C
Incubation	175 min (2 h 55 min)	60°C
Hold	Indefinite†	20°C

† Converted DNA can be left in the thermal cycler overnight without any loss of performance.

5. Place the PCR tubes containing the bisulfite reactions into the thermal cycler. Start the thermal cycling incubation.

   IMPORTANT: Since the bisulfite reaction is not overlaid with mineral oil, only thermal cyclers with heated lids are suitable for this procedure.

   Converted DNA can be left in the thermal cycler overnight without any loss of performance.

Cleanup of bisulfite converted DNA

6. Once the bisulfite conversion is complete, briefly centrifuge the PCR tubes containing the bisulfite reactions, and then transfer the complete bisulfite reactions to clean 1.5 ml microcentrifuge tubes.

   Transfer of precipitates in the solution will not affect the performance or yield of the reaction.
7. Add 310 µl freshly prepared Buffer BL containing 10 µg/ml carrier RNA (see "Things to do before starting", page 29) to each sample. Mix the solutions by vortexing and then centrifuge briefly.
8. Add 250 µl ethanol (96–100%) to each sample. Mix the solutions by pulse vortexing for 15 s, and centrifuge briefly to remove the drops from inside the lid.
9. Place the necessary number of EpiTect spin columns and collection tubes in a suitable rack. Transfer the entire mixture from the tubes in steps 7 and 8 into the corresponding EpiTect spin columns.
10. Centrifuge the spin columns at maximum speed for 1 min. Discard the flow-through, and place the spin columns back into the collection tubes.
11. Add 500 µl Buffer BW (wash buffer) to each spin column, and centrifuge at maximum speed for 1 min. Discard the flow-through, and place the spin columns back into the collection tubes.
12. Add 500 µl Buffer BD (desulfonation buffer) to the spin columns and incubate for 15 min at room temperature (15–25°C).

    If there are precipitates in Buffer BD, avoid transferring them to the spin columns.

    IMPORTANT: The bottle containing Buffer BD should be closed immediately after use to avoid acidification from carbon dioxide in the air.

    Note: It is important to close the lids of the spin columns before incubation.

13. Centrifuge the spin columns at maximum speed for 1 min. Discard the flow-through, and place the spin columns back into the collection tubes.
14. Add 500 µl Buffer BW to each spin column and centrifuge at maximum speed for 1 min. Discard the flow-through, and place the spin columns back into the collection tubes.
15. Repeat step 14 once.
16. Place the spin columns into new 2 ml collection tubes, and centrifuge the spin columns at maximum speed for 1 min to remove any residual liquid.
17. **Recommended**: Place the spin columns with open lids into a clean 1.5 ml microcentrifuge tube (not provided) and incubate the columns for 5 min at 56°C in a heating block.

    This step enables the evaporation of any remaining liquid.
18. Place the spin columns into clean 1.5 ml microcentrifuge tubes (not provided). Dispense 20 µl Buffer EB onto the center of each membrane. Elute the purified DNA by centrifugation for 1 min at approximately 15,000 x *g* (12,000 rpm.)

    Note: To increase the yield of DNA in the eluate, dispense an additional 20 µl Buffer EB onto the center of each membrane, and centrifuge for 1 min at maximum speed.

    Note: If the purified DNA is to be stored for up to 24 h, we recommend storage at 2–8°C. For storage longer than 24 h, we recommend storage at –30°C to –15°C. At –30°C to –15°C , DNA converted and purified using the EpiTect Bisulfite Kit can be stored for least 3 years* without decrease of quality or conversion.

* Investigations into longer storage of converted DNA are ongoing. Contact QIAGEN for more information.

Protocol: Sodium Bisulfite Conversion of Unmethylated Cytosines in Small Amounts of Fragmented DNA

This protocol is designed for processing small amounts of DNA, which may also be fragmented. An optimized binding step in the cleanup stage facilitates binding of DNA. Less than 500 pg DNA in a volume of up to 40 µl can be processed using this protocol.

Important points before starting

- Each aliquot of Bisulfite Mix is sufficient for 8 conversion reactions. If converting fewer than 8 DNA samples, reconstituted Bisulfite Mix can be stored at –30°C to –15°C for up to 4 weeks without any loss of performance.
- DNA Protect Buffer should turn from green to blue after addition to DNA–Bisulfite Mix (step 2), indicating sufficient mixing and correct pH for the bisulfite conversion reaction.
- Perform all centrifugation steps at room temperature (15–25°C).

Things to do before starting

- Add 30 ml ethanol (96–100%) to Buffer BW and store at room temperature (15–25°C). Invert the bottle several times before starting the procedure.
- Add 27 ml ethanol (96–100%) to Buffer BD and store at 2–8°C. Invert the bottle several times before starting the procedure and make sure to close the bottle immediately after use. White precipitates may form in the
  Buffer BD–ethanol mix after some storage time. These precipitates will not affect the performance of Buffer BD. However, avoid transferring precipitates to the EpiTect spin column.

- Add 310 µl RNase-free water to the lyophilized carrier RNA (310 µg) to obtain a 1 µg/µl solution. Dissolve the carrier RNA thoroughly by vortexing. When processing 48 samples at once, add the complete volume of dissolved carrier RNA to the bottle of Buffer BL, and check the box on the bottle lid label. If processing fewer samples, split the dissolved carrier RNA into conveniently sized aliquots (e.g., 50 µl) and store at –30°C to –15°C. Aliquots can be stored for up to 1 year. If fewer than 48 conversions will be performed in a 2-week period, then only make up enough Buffer BL–carrier RNA solution as required (see Table 1, page 18, for example volumes). Carrier RNA enhances binding of DNA to the EpiTect spin-column membrane, especially if there are very few target molecules in the sample.
- Add dissolved carrier RNA to Buffer BL. Calculate the volume of Buffer BL and dissolved carrier RNA required for the number of samples to be processed (see Table 1, page 18, for example volumes). If Buffer BL contains precipitates, dissolve by heating (maximum 70°C) with gentle agitation.
- Equilibrate samples and buffers to room temperature.
- **Optional**: Set a thermomixer, heating block or heated orbital incubator to 60°C for use in step 1.

Procedure

Bisulfite DNA conversion

1. Thaw DNA to be used in the bisulfite reactions. Dissolve the required number of Bisulfite Mix aliquots by adding 800 µl RNase-free water to each aliquot. Vortex until the Bisulfite Mix is completely dissolved. This can take up to 5 min.

   Note: If necessary, heat the Bisulfite Mix–RNase-free water solution to 60°C and vortex again.

   Note: Do not place dissolved Bisulfite Mix on ice.

2. Prepare the bisulfite reactions in 200 µl PCR tubes according to Table 8. Add each component in the order listed.

   Note: The combined volume of DNA solution and RNase-free water must total 40 µl.

Table 8. Bisulfite reaction components

Component	Volume per reaction (µl)
DNA solution (1 ng – 2 µg)	Variable* (maximum 20 µl)
RNase-free water	Variable*
Bisulfite Mix (dissolved), see step 1	85
DNA Protect Buffer	35
Total volume	140

* The combined volume of DNA solution and RNase-free water must total 20 µl.

3. Close the PCR tubes and mix the bisulfite reactions thoroughly. Store tubes at room temperature (15–25°C).

   Note: DNA Protect Buffer should turn from green to blue after addition to DNA–Bisulfite Mix, indicating sufficient mixing and correct pH for the bisulfite conversion reaction.

4. Perform the bisulfite DNA conversion using a thermal cycler. Program the thermal cycler according to Table 9.

   The complete cycle should take approximately 5 h.

   Note: If using a thermal cycler that does not allow you to enter the reaction volume (140 µl), set the instrument to the largest volume setting available.

Table 9. Bisulfite conversion thermal cycler conditions

Step	Time	Temperature
Denaturation	5 min	95°C
Incubation	25 min	60°C
Denaturation	5 min	95°C
Incubation	85 min (1 h 25 min)	60°C
Denaturation	5 min	95°C
Incubation	175 min (2 h 55 min)	60°C
Hold	Indefinite†	20°C

† Converted DNA can be left in the thermal cycler overnight without any loss of performance.

5. Place the PCR tubes containing the bisulfite reactions into the thermal cycler. Start the thermal cycling incubation.

   IMPORTANT: Since the bisulfite reaction is not overlaid with mineral oil, only thermal cyclers with heated lids are suitable for this procedure. It is important to use PCR tubes that close tightly.

   Converted DNA can be left in the thermal cycler overnight without any loss of performance.

Cleanup of bisulfite converted DNA

6. Once the bisulfite conversion is complete, briefly centrifuge the PCR tubes containing the bisulfite reactions, and then transfer the complete bisulfite reactions to clean 1.5 ml microcentrifuge tubes.

   Transfer of precipitates in the solution will not affect the performance or yield of the reaction.
7. Add 310 µl freshly prepared Buffer BL containing 10 µg/ml carrier RNA (see "Things to do before starting", page 35) to each sample. Mix the solutions by vortexing and then centrifuge briefly.

8. Add 250 µl ethanol (96–100%) to each sample. Mix the solutions by pulse vortexing for 15 s, and then centrifuge briefly to remove the drops from inside the lid.
9. Place the necessary number of EpiTect spin columns and collection tubes in a suitable rack. Transfer the entire mixture from the tubes in steps 7 and 8 into the corresponding EpiTect spin columns.
10. Centrifuge the spin columns at maximum speed for 1 min. Discard the flow-through, and place the spin columns back into the collection tubes.
11. Add 500 µl Buffer BW to each spin column, and centrifuge at maximum speed for 1 min. Discard the flow-through, and place the spin columns back into the collection tubes.
12. Add 500 µl Buffer BD to each spin column and incubate for 15 min at room temperature. (15–25°C)

    If there are precipitates in Buffer BD, avoid transferring them to the spin columns.

    IMPORTANT: The bottle containing Buffer BD should be closed immediately after use to avoid acidification from carbon dioxide in the air.

    Note: It is important to close the lids of the spin columns before incubation.
13. Centrifuge the spin columns at maximum speed for 1 min. Discard the flow-through, and place the spin columns back into the collection tubes.
14. Add 500 µl Buffer BW to each spin column and centrifuge at maximum speed for 1 min. Discard the flow-through, and place the spin columns back into the collection tubes.
15. Repeat step 14 once.
16. Place the spin columns into new 2 ml collection tubes, and centrifuge the spin columns at maximum speed for 1 min to remove any residual liquid.
17. **Recommended**: Place the spin columns with open lids into clean 1.5 ml microcentrifuge tubes (not provided) and incubate the spin columns for 5 min at 56°C in a heating block.

    This step enables evaporation of any remaining liquid.

18. Place the spin columns into clean 1.5 ml microcentrifuge tubes (not provided). Dispense 20 µl Buffer EB onto the center of each membrane. Elute the purified DNA by centrifugation for 1 min at approximately 15,000 x *g* (12,000 rpm).

Note: To increase the yield of DNA in the eluate, dispense an additional 20 µl Buffer EB onto the center of each membrane, and centrifuge for 1 min at maximum speed.

Note: If the purified DNA is to be stored for up to 24 h, we recommend storage at 2–8°C. For storage longer than 24 h, we recommend storage at –30°C to –15°C. At –30°C to –15°C, DNA converted and purified using the EpiTect Bisulfite Kit can be stored for at least 3 years* without decrease of quality or conversion.

* Investigations into longer storage of converted DNA are ongoing. Contact QIAGEN for more information.

Troubleshooting Guide

This troubleshooting guide may be helpful in solving any problems that may arise. For more information, see also the Frequently Asked Questions page at our Technical Support Center: **www.qiagen.com/FAQ/FAQlist.aspx**. The scientists in QIAGEN Technical Services are always happy to answer any questions you may have about either the information and protocols in this handbook or sample and assay technologies (for contact information, see back cover or visit **www.qiagen.com**).

	Comments and suggestions
**Little or no DNA recovery in purification step**	
a) Carrier RNA not added to Buffer BL	Prepare carrier RNA and add to Buffer BL, as described in "Things to do before starting", pages 17, 23, 29 or 35.
b) Buffer BW or Buffer BD prepared incorrectly	Check that Buffer BW and BD concentrates were diluted with the correct volumes of ethanol (96–100%). Do not use denatured alcohol, which contains other substances, such as methanol or methylethylketone.
c) Buffer BW or BD prepared with 70% ethanol	Check that Buffer BW and BD concentrates were diluted with 96–100% ethanol. Do not use denatured alcohol, which contains other substances, such as methanol or methylethylketone.
d) Buffer BW and Buffer BD used in the wrong order	Ensure that Buffer BW and Buffer BD are used in the correct order in the protocol.
e) Sample not completely passed through the membrane	Centrifuge for 1 min at full speed or until the entire sample has passed through the membrane.
f) Buffer BL contains precipitates	Check Buffer BL for precipitate. Dissolve by heating (maximum 70°C) with gentle agitation.
**Low conversion rate**	
a) Bisulfite reaction components not added in the correct order	Ensure that DNA, Bisulfite Mix and DNA Protect Buffer are added in the order indicated in Table 2 (page 19), Table 4 (page 25), Table 6 (page 31) or Table 8 (page 37).

	Comments and suggestions
b) Incorrect thermal cycling conditions used	Use the thermal cycling conditions given in Table 3 (page 20), Table 5 (page 25), Table 7 (page 32) or Table 9 (page 37).
c) Poor DNA quality (i.e., protein contamination)	Check that the A_{260}/A_{280} ratio of the sample DNA is between 1.7 and 1.9.<br>Ensure that sample DNA is purified using an appropriate kit (see ordering information, starting on page 44, for suitable QIAGEN kits for DNA purification).
d) Amount of DNA used outside recommended range	Increase or decrease the amount of starting DNA material to stay within the range of 1 ng to 2 µg DNA for standard applications, 1 ng to 500 ng for low concentrations of DNA in solutions (page 23), or 1 ng to 2 µg for DNA from FFPE tissues (page 29).
e) Bisulfite Mix stored incorrectly	Dissolved Bisulfite Mix can be stored at –30°C to –15°C for 4 weeks.
f) DNA Protect Buffer not added	Upon addition of DNA Protect Buffer, the DNA–Bisulfite Mix solution should turn from green to blue indicating sufficient mixing and the correct pH for DNA binding to the EpiTect spin column. If this color change does not occur, repeat the reaction ensuring that DNA Protect Buffer has been added.
g) Presence of a special CpG region with high sequence representation of CpGs	Extend the bisulfite conversion thermal-cycling conditions by adding the following step: Denaturation for 5 min at 95°C and 2 h at 60°C; then hold at 20°C.
**Poor results in downstream methylation-specific PCR**	
a) Little or no PCR product even in control reaction	If performing hot-start PCR, confirm that the initial enzyme activation step was performed.<br>Ensure that all PCR components were added and that suitable cycling conditions were used.

	Comments and suggestions
b) Failure of conversion reaction	The starting DNA was not sufficiently pure. Ensure that only high-quality DNA is used for the conversion reaction. See ordering information, starting on page 44, for suitable QIAGEN kits for DNA purification.<br>Ensure that all steps of the modification and cleanup protocol were followed.<br>Sample DNA was degraded before modification reaction. Ensure that sample DNA is handled and stored correctly.<br>PCR primers were not appropriate or incorrectly designed. Check primer design.<br>Amount of template DNA used in PCR was insufficient. Increase amount of template DNA.
**Unexpected findings in buffers**	
a) Color of DNA Protect Buffer changes from light green to olive during storage	DNA Protect Buffer is stable at 2–8°C for one year, and a change in color within this time has no influence on performance.
b) Precipitates in Buffer BD	There may be slight clouding and/or insoluble precipitates in Buffer BD during storage.<br>Buffer BD is stable at 2–8°C for one year, and a precipitate within this time has no influence on performance. Precipitates should not be transferred onto the membrane.

Ordering Information

Product	Contents	Cat. no.
EpiTect Bisulfite Kit (48)	48 EpiTect Bisulfite Spin Columns, Reaction Mix, DNA Protect Buffer, Carrier RNA, Buffers	59104
EpiTect 96 Bisulfite Kit (2)	2x EpiTect Bisulfite 96-well Plates, Reaction Mix, DNA Protect Buffer, Carrier RNA, Buffers	59110
EpiTect Whole Bisulfitome Kit (25)	REPLI-g® Midi DNA Polymerase, EpiTect WBA Reaction Buffer, Nuclease-free water	59203
EpiTect MSP PCR Kit (100)	EpiTect MSP Master Mix for 100x50 µl reactions	59305
**EpiTect MethyLight PCR Kit — for real time quantification of methylation status**		
EpiTect MethyLight PCR Kit (200)	Master Mix for Methylation-specific Real-Time PCR analysis, 200 x50 µl reactions	59436
EpiTect MethyLight PCR + ROX Vial Kit (200)	Master Mix without ROX for Methylation-specific Real-Time PCR analysis, 200 x50 µl reactions	59496
EpiTect Control DNA, methylated (100)	Methylated and bisulfite converted control human DNA for 100 control PCR reactions	59655
EpiTect Control DNA, unmethylated (100)	Unmethylated and bisulfite converted control human DNA for 100 control PCR reactions	59665
EpiTect Control DNA (1000)	Unmethylated control human DNA for 1000 control PCR reactions	59568

Product	Contents	Cat. no.
EpiTect PCR Control DNA Set (100)	Control human DNA Set for 100 control PCR reactions	59695
**QIAamp DNA Mini Kit — for purification of genomic, mitochondrial, bacterial, parasite or viral DNA from a wide variety of samples**		
QIAamp DNA Mini Kit (50)	For 50 DNA preps: 50 QIAamp Mini Spin Columns, QIAGEN Proteinase K, Reagents, Buffers, Collection Tubes (2 ml)	51304
QIAamp DNA Mini Kit (250)	For 250 DNA preps: 250 QIAamp Mini Spin Columns, QIAGEN Proteinase K, Reagents, Buffers, Collection Tubes (2 ml)	51306
**QIAamp DNA Blood Kits — for purification of genomic, mitochondrial or viral DNA from blood and related body fluids**		
QIAamp DNA Blood Mini Kit (50)*	For 50 DNA minipreps: 50 QIAamp Mini Spin Columns, QIAGEN Protease, Reagents, Buffers, Collection Tubes (2 ml)	51104
QIAamp DNA Blood Midi Kit (20)*	For 20 DNA midipreps: 20 QIAamp Midi Spin Columns, QIAGEN Protease, Buffers, Collection Tubes (15 ml)	51183
QIAamp DNA Blood Maxi Kit (10)*	For 10 DNA maxipreps: 10 QIAamp Maxi Spin Columns, QIAGEN Protease, Buffers, Collection Tubes (50 ml)	51192
**EZ1 DNA Tissue Kit — for automated purification of high-quality DNA from 1–14 tissue samples using the EZ1 instruments**		
EZ1 DNA Tissue Kit (48)	For 48 DNA preps: 48 Reagent Cartridges (Tissue), Disposable Tip Holders, Disposable Filter-Tips, Sample Tubes (2 ml), Elution Tubes (1.5 ml), Buffer G2, Proteinase K	953034

Product	Contents	Cat. no.
EZ1 DNA Tissue Card	Preprogrammed card for purification of DNA using the BioRobot® EZ1	9015588
EZ1 Advanced DNA Tissue Card	Preprogrammed card for purification of DNA using the EZ1 Advanced	9018295
EZ1 Advanced XL DNA Tissue Card	Preprogrammed card for purification of DNA using the EZ1 Advanced XL	9018701
EZ1 DNA Paraffin Section Card	Preprogrammed card for purification of DNA using the BioRobot EZ1	9015862
EZ1 Advanced DNA Paraffin Section Card	Preprogrammed card for purification of DNA using the EZ1 Advanced	9018298
EZ1 Advanced XL DNA Paraffin Section Card	Preprogrammed card for purification of DNA using the EZ1 Advanced XL	9018700
**DNeasy Tissue Kits — for purification of total cellular DNA from animal tissues and cells, yeast or bacteria**		
DNeasy Blood & Tissue Kit (50)*	50 DNeasy Mini Spin Columns, Proteinase K, Buffers, Collection Tubes (2 ml)	69504
**EZ1 DNA Blood Kits — for automated purification of DNA from 1–14 blood samples using the EZ1 instruments**		
EZ1 DNA Blood 200 µl Kit (48)	For 48 DNA preps: 48 Reagent Cartridges (Blood 200 µl), Disposable Tip Holders, Disposable Filter-Tips, Sample Tubes (2 ml), Elution Tubes (1.5 ml)	951034
EZ1 DNA Blood 350 µl Kit (48)	For 48 DNA preps: 48 Reagent Cartridges (Blood 350 µl), Disposable Tip Holders, Disposable Filter-Tips, Sample Tubes (2 ml), Elution Tubes (1.5 ml)	951054
EZ1 DNA Blood Card	Preprogrammed card for BioRobot EZ1 DNA Blood 200 µl and 350 µl Protocols	9015585

Product	Contents	Cat. no.
EZ1 Advanced DNA Blood Card	Preprogrammed card for purification of DNA using the EZ1 Advanced	9018293
EZ1 Advanced XL DNA Blood Card	Preprogrammed card for purification of DNA using the EZ1 Advanced XL	9018695
**QIAcube Connect — for fully automated nucleic acid extraction with QIAGEN spin-column kits**		
QIAcube Connect†	Instrument, connectivity package, 1 year warranty on parts and labor	Inquire
Starter Pack, QIAcube	Filter-tips, 200 µl (1024), 1000 µl filter-tips (1024), 30 ml reagent bottles (12), rotor adapters (240), elution tubes (240), rotor adapter holder	990395

* Larger kit sizes available; please inquire.

† All QIAcube Connect instruments are provided with a region-specific connectivity package, including tablet and equipment necessary to connect to the local network. Further, QIAGEN offers comprehensive instrument service products, including service agreements, installation, introductory training and preventive subscription. Contact your local sales representative to learn about your options.

For up-to-date licensing information and product-specific disclaimers, see the respective QIAGEN kit handbook or user manual. QIAGEN kit handbooks and user manuals are available at **www.qiagen.com** or can be requested from QIAGEN Technical Services or your local distributor.

Document Revision History

Date	Changes
February 2020	Updated text, ordering information and intended use for QIAcube Connect.

Notes

Notes

Notes

Limited License Agreement

Use of this product signifies the agreement of any purchaser or user of the EpiTect Bisulfite Kit to the following terms:

1. The EpiTect Bisulfite Kit may be used solely in accordance with the *EpiTect Bisulfite Kit Handbook* and for use with components contained in the Kit only. QIAGEN grants no license under any of its intellectual property to use or incorporate the enclosed components of this Kit with any components not included within this Kit except as described in the *EpiTect Bisulfite Kit Handbook* and additional protocols available at www.qiagen.com.
2. Other than expressly stated licenses, QIAGEN makes no warranty that this Kit and/or its use(s) do not infringe the rights of third-parties.
3. This Kit and its components are licensed for one-time use and may not be reused, refurbished, or resold.
4. QIAGEN specifically disclaims any other licenses, expressed or implied other than those expressly stated.
5. The purchaser and user of the Kit agree not to take or permit anyone else to take any steps that could lead to or facilitate any acts prohibited above. QIAGEN may enforce the prohibitions of this Limited License Agreement in any Court, and shall recover all its investigative and Court costs, including attorney fees, in any action to enforce this Limited License Agreement or any of its intellectual property rights relating to the Kit and/or its components.

For updated license terms, see **www.qiagen.com**.

Trademarks: QIAGEN®, Sample to Insight®, QIAamp®, QIAcube®, BioRobot®, DNeasy®, EpiTect®, EZ1®, FlexiGene®, Pyrosequencing®, REPLI-g® (QIAGEN Group); Eppendorf® (Eppendorf-Netheler-Hinz GmbH). Registered names, trademarks, etc. used in this document, even when not specifically marked as such, are not to be considered unprotected by law.

HB-0243-003 © 2020 QIAGEN, all rights reserved.

HB-0243-003
