# Supplementary material for: Screening and identifying of biomarkers in early colorectal cancer and adenoma based on genome-wide methylation profiles
Source: World J Surg Oncol. 2023 Oct 2;21:312. doi: 10.1186/s12957-023-03189-1 (PMC10544418; doi:10.1186/s12957-023-03189-1)
Supplement: Supplementary file 4 — Additional file 4: Supplement 4. EpiTect Bisulfite Kit. [file 12957_2023_3189_MOESM4_ESM.docx]

**EpiTect Bisulfite Kit**

1. Configuration of reaction system:

|  |  | Recommended for small amounts of DNA |
| --- | --- | --- |
| DNA water（50ng-2ug) | Total 20 uL | Total 40 uL |
| Bisulfite Mix | 85 uL | 85 uL |
| DNA protect Buffer | 35 uL | 15 uL |
| Total volume | 140 uL | 140 uL |

2. Vortex, mix, observe the color change of DNA protect Buffer (from green to blue), indicating sufficient mixing and correct PH value during the transformation process.

3. The transformation was performed on the Type 9700 PCR instrument (with a hot cover), and the procedure was as follows:

| Step | Time | Temperature |
| --- | --- | --- |
| Denaturation | 5 min | 95℃ |
| Incubation | 25 min | 60℃ |
| Denaturation | 5 min | 95℃ |
| Incubation | 85 min | 60℃ |
| Denaturation | 5 min | 95℃ |
| Incubation | 175 min | 60℃ |
| Hold | Indefinite | 20℃ |

4. the time is about 5 hours, choose the maximum reaction system.

5. After the transformation, centrifuge and transfer the above products to 1.5mL centrifuge tube;

6. Add 560 uL Buffer BL, mix and centrifuge briefly;

7. All the mixtures in step 6 were transferred to the EpiTect spin column;

8. Centrifuge at 14,000 rpm for 1 min; Discard the waste liquid, the column is still put back on the collection pipe;

9. Add 500 uL Buffer BW at 14,000 rpm and centrifuge for 1 min; Discard the waste liquid, the column is still put back on the collection pipe;

10. Add 500 uL Buffer BD, cover tightly, and place at room temperature for 15 min;

11. Centrifuge at 14,000 rpm for 1 min; Discard the waste liquid, the column is still put back on the collection pipe;

12. Add 500 uL Buffer BW at 14,000 rpm and centrifuge for 1 min; Discard the waste liquid, the column is still put back on the collection pipe;

13. Repeat Step 12;

14. Transfer the column to a new collection tube at 14,000 rpm and centrifuge for 1 min to remove the residue;

15. Place the column on the 56℃ heating module, open the cover and place it for 15min, then dry it;

16. The column was placed on a new 1.5mL centrifuge tube, 20 uL Buffer EB was added, centrifuged at 12,000 rpm for 1 min, and the DNA was refrigerated.
