# Supplementary material for: Screening and identifying of biomarkers in early colorectal cancer and adenoma based on genome-wide methylation profiles
Source: World J Surg Oncol. 2023 Oct 2;21:312. doi: 10.1186/s12957-023-03189-1 (PMC10544418; doi:10.1186/s12957-023-03189-1)
Supplement: Supplementary file 5 — Additional file 5: Table S1. Patient characteristics. [file 12957_2023_3189_MOESM5_ESM.docx]

Table S1. Patient characteristics

|  | Colorectal Cancer tissue | Adenomas | Normal mucosa |
| --- | --- | --- | --- |
| Number | 46 | 9 | 20 |
| Middle age | 59 | 53 | 60 |
| Sex |  |  |  |
| male | 20 | 4 | 8 |
| female | 26 | 5 | 12 |
| Smoking | - | - | - |
| Location |  |  |  |
| colon | 35 | 7 | 13 |
| rectum | 11 | 2 | 7 |
| Distant metastasis |  |  |  |
| Presence | 10 |  |  |
| Absence | 36 |  |  |
| Lymph node metastasis |  |  |  |
| Presence | 19 |  |  |
| Absence | 27 |  |  |
| Stage |  |  |  |
| Stage I + Stage II | 16 |  |  |
| Stage III +Stage IV | 30 |  |  |
