# Supplementary material for: Screening and identifying of biomarkers in early colorectal cancer and adenoma based on genome-wide methylation profiles
Source: World J Surg Oncol. 2023 Oct 2;21:312. doi: 10.1186/s12957-023-03189-1 (PMC10544418; doi:10.1186/s12957-023-03189-1)
Supplement: Supplementary file 6 — Additional file 6: Table S2. Patient characteristics. [file 12957_2023_3189_MOESM6_ESM.docx]

Table S2. Patient characteristics

|  | Colorectal Cancer tissue | Adenomas | Normal mucosa |
| --- | --- | --- | --- |
| Number | 68 | 31 | 49 |
| Middle age | 58 | 54 | 58 |
| Sex |  |  |  |
| male | 37 | 15 | 11 |
| female | 31 | 16 | 10 |
| Smoking | - | - | - |
| Location |  |  |  |
| colon | 36 | 18 | 5 |
| rectum | 32 | 13 | 16 |
| Distant metastasis |  |  |  |
| Presence | 14 |  |  |
| Absence | 54 |  |  |
| Lymph node metastasis |  |  |  |
| Presence | 29 |  |  |
| Absence | 39 |  |  |
| Stage |  |  |  |
| Stage I + Stage II | 36 |  |  |
| Stage III +Stage IV | 32 |  |  |
