# Supplementary material for: Screening and identifying of biomarkers in early colorectal cancer and adenoma based on genome-wide methylation profiles
Source: World J Surg Oncol. 2023 Oct 2;21:312. doi: 10.1186/s12957-023-03189-1 (PMC10544418; doi:10.1186/s12957-023-03189-1)
Supplement: Supplementary file 7 — Additional file 7: Table S3. Correlations of clinical characteristics with methylation status of ZNF471 in colorectal cancers. [file 12957_2023_3189_MOESM7_ESM.docx]

**Table S3** Correlations of clinical characteristics with methylation status of ZNF471 in colorectal cancers

| Groups | N | Methylation Index（x±s） | Range | | Median | Mann-Whitney U value | Sig. |
| --- | --- | --- | --- | --- | --- | --- | --- |
| Gender | | | | | | | |
| Male | 37 | 22.09±16.77 | 0.00 | 63.47 | 19.57 | 527.500 | 0.571 |
| Female | 31 | 26.98±24.40 | 0.00 | 77.69 | 23.12 |  |  |
| Age | | | | | | | |
| >58years | 33 | 22.62±21.49 | 0.00 | 76.69 | 16.51 | 466.500 | 0.178 |
| ≤58years | 35 | 25.92 ±18.47 | 3.30 | 77.69 | 25.70 |  |  |
| Tumor location | | | | | | | |
| Colon | 36 | 22.86±16.94 | 0.00 | 63.47 | 23.99 | 549.000 | 0.740 |
| Rectum | 32 | 25.96 ±22.97 | 3.30 | 77.69 | 15.64 |  |  |
| Distant metastasis | | | | | | | |
| Presence | 14 | 30.37 ± 21.86 | 2.77 | 72.69 | 30.91 | 298.500 | 0.228 |
| Absence | 54 | 22.75±19.28 | 0.00 | 77.69 | 17.63 |  |  |
| Lymph node metastasis | | | | | | | |
| Presence | 29 | 27.18± 22.33 | 2.29 | 77.69 | 20.82 | 497.000 | 0.396 |
| Absence | 39 | 22.19±17.91 | 0.00 | 63.47 | 17.55 |  |  |
| Tumor Staging | | | | | | | |
| I+II stage | 36 | 20.79 ±16.52 | 0.00 | 63.47 | 16.35 | 479.000 | 0.233 |
| III+IV stage | 32 | 28.29 ±22.75 | 2.29 | 77.69 | 22.41 |  |  |
