# Supplementary material for: Screening and identifying of biomarkers in early colorectal cancer and adenoma based on genome-wide methylation profiles
Source: World J Surg Oncol. 2023 Oct 2;21:312. doi: 10.1186/s12957-023-03189-1 (PMC10544418; doi:10.1186/s12957-023-03189-1)
Supplement: Supplementary file 8 — Additional file 8: Table S4. Correlations of clinical characteristics with methylation status of SND1 in colorectal cancers. [file 12957_2023_3189_MOESM8_ESM.docx]

**Table S4** Correlations of clinical characteristics with methylation status of SND1 in colorectal cancers

| Groups | N | Methylation Index（x±s） | Range | | Median | Mann-Whitney U value | Sig. |
| --- | --- | --- | --- | --- | --- | --- | --- |
| Gender | | | | | | | |
| Male | 37 | 61.28±17.58 | 13.30 | 86.58 | 64.40 | 529.000 | 0.584 |
| Female | 31 | 54.43±26.84 | 4.11 | 88.11 | 66.43 |  |  |
| Age | | | | | | | |
| >58years | 33 | 53.10±24.55 | 4.11 | 84.50 | 56.77 | 488.500 | 0.113 |
| ≤58years | 35 | 62.92 ±19.26 | 4.97 | 88.11 | 67.49 |  |  |
| Tumor location | | | | | | | |
| Colon | 36 | 56.34±19.59 | 9.89 | 86.58 | 65.18 | 564.500 | 0.888 |
| Rectum | 32 | 59.78±25.35 | 4.11 | 88.11 | 65.44 |  |  |
| Distant metastasis | | | | | | | |
| Presence | 14 | 68.71±15.78 | 32.67 | 84.50 | 72.84 | 239.000 | 0.035 |
| Absence | 54 | 55.42±23.13 | 4.11 | 88.11 | 61.50 |  |  |
| Lymph node metastasis | | | | | | | |
| Presence | 29 | 59.51 ±23.32 | 4.11 | 88.11 | 63.940 | 518.500 | 0.560 |
| Absence | 39 | 57.15±21.90 | 4.97 | 84.50 | 66.430 |  |  |
| Tumor Staging | | | | | | | |
| I+II stage | 36 | 55.59 ±22.03 | 4.97 | 80.43 | 64.3400 | 472.500 | 0.203 |
| III+IV stage | 32 | 61.04 ±22.77 | 4.11 | 88.11 | 66.4550 |  |  |
