# Supplementary material for: Screening and identifying of biomarkers in early colorectal cancer and adenoma based on genome-wide methylation profiles
Source: World J Surg Oncol. 2023 Oct 2;21:312. doi: 10.1186/s12957-023-03189-1 (PMC10544418; doi:10.1186/s12957-023-03189-1)
Supplement: Supplementary file 9 — Additional file 9: Table S5. Correlations of clinical characteristics with methylation status of SPOCK1 in colorectal cancers. [file 12957_2023_3189_MOESM9_ESM.docx]

**Table S5** Correlations of clinical characteristics with methylation status of SPOCK1 in colorectal cancers

| Groups | N | Methylation Index（x±s） | Range | | Median | Mann-Whitney U value | Sig. |
| --- | --- | --- | --- | --- | --- | --- | --- |
| Gender | | | | | | | |
| Male | 37 | 38.54±18.92 | 3.41 | 75.21 | 36.53 | 460.000 | 0.162 |
| Female | 31 | 45.46±19.91 | 4.95 | 77.11 | 40.26 |  |  |
| Age | | | | | | | |
| >58years | 33 | 41.84±18.54 | 4.95 | 74.25 | 39.28 | 576.000 | 0.985 |
| ≤58years | 35 | 41.56 ±20.71 | 3.41 | 77.11 | 38.98 |  |  |
| Tumor location | | | | | | | |
| Colon | 36 | 37.67±16.95 | 3.48 | 75.21 | 36.46 | 436.000 | 0.085 |
| Rectum | 32 | 46.23 ±21.48 | 3.41 | 77.11 | 51.09 |  |  |
| Distant metastasis | | | | | | | |
| Presence | 14 | 41.88 ± 18.51 | 3.48 | 68.57 | 40.22 | 373.000 | 0.940 |
| Absence | 54 | 41.65±19.97 | 3.41 | 77.11 | 39.13 |  |  |
| Lymph node metastasis | | | | | | | |
| Presence | 29 | 42.14± 22.80 | 3.41 | 77.11 | 37.59 | 558.000 | 0.926 |
| Absence | 39 | 41.36±17.03 | 6.98 | 74.25 | 40.26 |  |  |
| Tumor Staging | | | | | | | |
| I+II stage | 36 | 40.92 ±16.92 | 6.98 | 74.25 | 39.22 | 548.000 | 0.731 |
| III+IV stage | 32 | 42.56 ±22.37 | 3.41 | 77.11 | 38.44 |  |  |
