# Supplementary material for: Screening and identifying of biomarkers in early colorectal cancer and adenoma based on genome-wide methylation profiles
Source: World J Surg Oncol. 2023 Oct 2;21:312. doi: 10.1186/s12957-023-03189-1 (PMC10544418; doi:10.1186/s12957-023-03189-1)
Supplement: Supplementary file 10 — Additional file 10: Table S6. Correlations of clinical characteristics with methylation status of FBLIM1 in colorectal cancers. [file 12957_2023_3189_MOESM10_ESM.docx]

**Table S6** Correlations of clinical characteristics with methylation status of FBLIM1 in colorectal cancers

| Groups | N | Methylation Index（x±s） | Range | | Median | Mann-Whitney U value | Sig. |
| --- | --- | --- | --- | --- | --- | --- | --- |
| Gender | | | | | | | |
| Male | 37 | 61.41±47.19 | 4.84 | 80.51 | 62.09 | 544.000 | 0.716 |
| Female | 31 | 58.69±21.69 | 2.71 | 87.99 | 64.61 |  |  |
| Age | | | | | | | |
| >58years | 33 | 60.44±22.48 | 2.71 | 89.92 | 64.6100 | 477.000 | 0.217 |
| ≤58years | 35 | 53.63 ±23.91 | 4.84 | 85.71 | 60.3200 |  |  |
| Tumor location | | | | | | | |
| Colon | 36 | 5587±22.04 | 6.11 | 89.92 | 58.3900 | 515.000 | 0.454 |
| Rectum | 32 | 58.14±24.95 | 2.71 | 88.97 | 64.6300 |  |  |
| Distant metastasis | | | | | | | |
| Presence | 14 | 63.96±2118 | 15.64 | 89.92 | 70.7000 | 292.000 | 0.192 |
| Absence | 54 | 55.11±23.66 | 2.71 | 87.99 | 61.4850 |  |  |
| Lymph node metastasis | | | | | | | |
| Presence | 29 | 57.15±27.30 | 2.71 | 89.92 | 62.9200 | 514.000 | 0.523 |
| Absence | 39 | 56.77±20.20 | 7.92 | 88.97 | 61.4300 |  |  |
| Tumor Staging | | | | | | | |
| I+II stage | 36 | 55.05±19.94 | 7.92 | 87.99 | 57.9200 | 465.000 | 0.173 |
| III+IV stage | 32 | 59.06 ±26.76 | 2.71 | 89.92 | 70.1400 |  |  |
