# Supplementary material for: Screening and identifying of biomarkers in early colorectal cancer and adenoma based on genome-wide methylation profiles
Source: World J Surg Oncol. 2023 Oct 2;21:312. doi: 10.1186/s12957-023-03189-1 (PMC10544418; doi:10.1186/s12957-023-03189-1)
Supplement: Supplementary file 11 — Additional file 11: Table S7. Correlations of clinical characteristics with methylation status of OTX1 in colorectal cancers. [file 12957_2023_3189_MOESM11_ESM.docx]

**Table S7** Correlations of clinical characteristics with methylation status of OTX1 in colorectal cancers

| Groups | N | Methylation Index（x±s） | Range | | Median | Mann-Whitney U value | Sig. |
| --- | --- | --- | --- | --- | --- | --- | --- |
| Gender | | | | | | | |
| Male | 37 | 57.28±14.79 | 11.38 | 82.27 | 58.5000 | 519.500 | 0.506 |
| Female | 31 | 57.26±21.65 | 5.01 | 82.83 | 61.9800 |  |  |
| Age | | | | | | | |
| >58years | 33 | 55.26±18.02 | 7.70 | 82.24 | 57.3400 | 488.500 | 0.275 |
| ≤58years | 35 | 59.15 ±18.23 | 5.01 | 82.83 | 63.2400 |  |  |
| Tumor location | | | | | | | |
| Colon | 36 | 55.60±16.45 | 9.93 | 82.27 | 56.4350 | 457.500 | 0.145 |
| Rectum | 32 | 59.14±19.89 | 5.01 | 82.83 | 63.9500 |  |  |
| Distant metastasis | | | | | | | |
| Presence | 14 | 58.53 ±19.32 | 9.93 | 82.24 | 58.46 | 359.500 | 0.779 |
| Absence | 54 | 56.94±17.95 | 5.01 | 82.83 | 60.95 |  |  |
| Lymph node metastasis | | | | | | | |
| Presence | 29 | 56.07±18.86 | 7.70 | 82.83 | 55.53 | 513.500 | 0.519 |
| Absence | 39 | 58.16±17.11 | 5.01 | 82.24 | 63.01 |  |  |
| Tumor Staging | | | | | | | |
| I+II stage | 36 | 56.62 ±17.47 | 5.01 | 81.74 | 61.31 | 551.5 | 0.763 |
| III+IV stage | 32 | 58.00 ±19.04 | 7.70 | 82.83 | 60.95 |  |  |
