# Supplementary material for: Screening and identifying of biomarkers in early colorectal cancer and adenoma based on genome-wide methylation profiles
Source: World J Surg Oncol. 2023 Oct 2;21:312. doi: 10.1186/s12957-023-03189-1 (PMC10544418; doi:10.1186/s12957-023-03189-1)
Supplement: Supplementary file 12 — Additional file 12: Table S8. The primers of methylation markers. [file 12957_2023_3189_MOESM12_ESM.docx]

Table S8. The primers of methylation markers

| **Gene** | **Primer sequence（5' → 3'）** | **Annealing temperature（℃）** |
| --- | --- | --- |
| ZNF471 | Forward primer: GGATGAGAGAGTTTGGGTGTTAGA | 56.9 |
|  | Reverse primer: CCCATCTCTCCCACAACTCA | 59.9 |
|  | Sequencing primer: CCACCCAAAACAAAC | 44.1 |
|  | Sequencing primer:GGAGTAGGAGGGATTTGA | 39.8 |
| SND1 | Forward primer: GAGGGAGTAGTTGGGTGG | 60.5 |
|  | Reverse primer: CCCCTAAACCCCCCTCTTCAAAACCA | 62.6 |
|  | Sequencing primer:TGGTAGTTGATAGGTTGGA | 44.1 |
| SPOCK1 | Forward primer: GGGGAGGGGTATTAAGTTTTTGAT | 61.1 |
|  | Reverse primer: AACCCCTCTCACCAATAAAAAACTCAT | 59.3 |
|  | Sequencing primer:TGGGGTATTAGGAGAATAGA | 41.9 |
| FBLIM1 | Forward primer: GGGGAGAGGGTTTAGTGAG | 55.2 |
|  | Reverse primer: CCCCTTACCTAACCCTAAC | 58.9 |
|  | Sequencing primer:AGGGTTTAGTGAGGT | 46.8 |
| OTX1 | Forward primer: GTTTTGGTAGTAGGATATGGGATAAGAG | 58.5 |
|  | Reverse primer: ATACACCAACTACCTCATCTATCT | 59.5 |
|  | Sequencing primer:GGTGGAGGAGTTTGTAGTTA | 39.7 |
