# Supplementary material for: Screening and identifying of biomarkers in early colorectal cancer and adenoma based on genome-wide methylation profiles
Source: World J Surg Oncol. 2023 Oct 2;21:312. doi: 10.1186/s12957-023-03189-1 (PMC10544418; doi:10.1186/s12957-023-03189-1)
Supplement: Supplementary file 13 — Additional file 13. Infinium HD assay methylation protocol guide. [file 12957_2023_3189_MOESM13_ESM.pdf]

illumina

Infinium HD Assay Methylation Protocol Guide

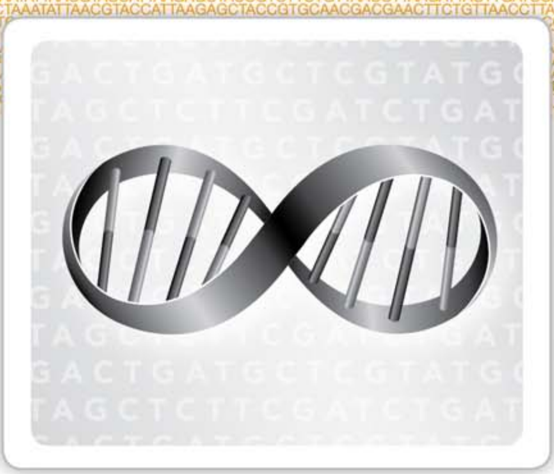

FOR RESEARCH USE ONLY

ILLUMINA PROPRIETARY
Catalog # WG-914-1001
Part # 15019519 Rev. A
December 2010

This document and its contents are proprietary to Illumina, Inc. and its affiliates ("Illumina"), and are intended solely for the contractual use of its customer in connection with the use of the product(s) described herein and for no other purpose. This document and its contents shall not be used or distributed for any other purpose and/or otherwise communicated, disclosed, or reproduced in any way whatsoever without the prior written consent of Illumina. Illumina does not convey any license under its patent, trademark, copyright, or common-law rights nor similar rights of any third parties by this document.

The instructions in this document must be strictly and explicitly followed by qualified and properly trained personnel in order to ensure the proper and safe use of the product(s) described herein. All of the contents of this document must be fully read and understood prior to using such product(s).

FAILURE TO COMPLETELY READ AND EXPLICITLY FOLLOW ALL OF THE INSTRUCTIONS CONTAINED HEREIN MAY RESULT IN DAMAGE TO THE PRODUCT(S), INJURY TO PERSONS, INCLUDING TO USERS OR OTHERS, AND DAMAGE TO OTHER PROPERTY.

ILLUMINA DOES NOT ASSUME ANY LIABILITY ARISING OUT OF THE IMPROPER USE OF THE PRODUCT(S) DESCRIBED HEREIN (INCLUDING PARTS THEREOF OR SOFTWARE) OR ANY USE OF SUCH PRODUCT(S) OUTSIDE THE SCOPE OF THE EXPRESS WRITTEN LICENSES OR PERMISSIONS GRANTED BY ILLUMINA IN CONNECTION WITH CUSTOMER'S ACQUISITION OF SUCH PRODUCT(S).

FOR RESEARCH USE ONLY

© 2008-2010 Illumina, Inc. All rights reserved.

Illumina, **illuminaDx**, **Solexa**, **Making Sense Out of Life**, **Oligator**, **Sentrix**, **GoldenGate**, **GoldenGate Indexing**, **DASL**, **BeadArray**, **Array of Arrays**, **Infinium**, **BeadXpress**, **VeraCode**, **IntelliHyb**, **iSelect**, **CSPro**, **GenomeStudio**, **Genetic Energy**, **HiSeq**, **HiScan**, **Eco**, and **TruSeq** are registered trademarks or trademarks of Illumina, Inc. All other brands and names contained herein are the property of their respective owners.

Revision History

Catalog #<br>Part #, Revision Letter	Date
Catalog # WG-914-1001<br>Part # 15019519 Rev A	December 2010

Table of Contents

Revision History iii
Table of Contents iv
List of Tables vi

Chapter 1 Overview 1

Introduction to Infinium HD Assay for Methylation 2
Audience and Purpose 3
Illumina Infinium HD Methylation Assay 4
Infinium HD Methylation BeadChip 11
Illumina Lab Protocols 12
Tracking Tools 13
Tecan GenePaint 16
iScan, HiScan, and AutoLoader2 17

Chapter 2 Manual Protocol 19

Introduction to Infinium HD Methylation Manual Protocol 20
Infinium HD Methylation Manual Workflow 21
Equipment, Materials, and Reagents 23
Quantitate DNA (Optional) 27
Make BCD Plate 35
Make MSA4 Plate 41
Incubate MSA4 Plate 46
Fragment MSA4 Plate 47
Precipitate MSA4 Plate 50
Resuspend MSA4 Plate 55
Hybridize Multi BeadChip 58
Wash BeadChip 78
Single-Base Extension and Stain BeadChip 90
Image BeadChip 110
Illumina GenomeStudio 112

Chapter 3 Automated Protocol 113

Introduction to Infinium HD Methylation Automated Protocol 114
Infinium HD Methylation Automated Workflow 115
Equipment, Materials, and Reagents 117
Quantitate DNA (Optional) 121
Make BCD Plate 131
Make MSA4 Plate 137
Incubate MSA4 Plate 144
Fragment MSA4 Plate 145
Precipitate MSA4 Plate 150
Resuspend MSA4 Plate 157
Hybridize Multi BeadChip 162
Wash BeadChip 180
Single-Base Extension and Stain BeadChip 192
Image BeadChip 227
Illumina GenomeStudio 229

Chapter 4 System Controls 231

Introduction 232
Control Dashboard 233
Controls Table 234
Control Diagrams 236

Index 245

Technical Assistance 247

List of Tables

Table 1 Sample Sheet Guidelines 13
Table 2 AutoLoader2 Features 17
Table 3 User-Supplied Equipment 23
Table 4 Illumina-Supplied Equipment 23
Table 5 User Supplied Materials 24
Table 6 Illumina-Supplied Reagents 26
Table 7 Concentrations of Lambda DNA 29
Table 8 Volumes for PicoGreen Reagents 31
Table 9 Genomic DNA Input for Bisulfite Conversion 38
Table 10 User-Supplied Equipment Methylation Assay (Automated) 117
Table 11 Illumina-Supplied Materials 118
Table 12 User-Supplied Materials Methylation Assay (Automated) 119
Table 13 Illumina-Supplied Reagents 120
Table 14 Concentrations of Lambda DNA 123
Table 15 Volumes for PicoGreen Reagents 125
Table 16 Genomic DNA Input for Bisulfite Conversion 134
Table 17 List of Reactions 201
Table 18 Controls 234
Table 19 Illumina General Contact Information 247
Table 20 Illumina Customer Support Telephone Numbers 247

Chapter 1

Overview

Introduction to Infinium HD Assay for Methylation 2
Audience and Purpose 3
Illumina Infinium HD Methylation Assay 4
Infinium HD Methylation BeadChip 11
Illumina Lab Protocols 12
Tracking Tools 13
Tecan GenePaint 16
iScan, HiScan, and AutoLoader2 17

Introduction to Infinium HD Assay for Methylation

The Illumina® Infinium® HD Assay for Methylation revolutionizes DNA Methylation analysis by streamlining sample preparation and enabling high multiplexing. Using one or two bead types per CpG locus and dual color channel approach, the Illumina Infinium HD Methylation Assay scales Methylation profiling to thousands of CpG loci per sample, dependent only on the number of features (bead types) on the array.

The Illumina Infinium HD Methylation Assay accomplishes this high multiplexing by combining Bisulfite conversion of genomic DNA and whole-genome amplification (WGA) sample preparation with direct, array-based capture and enzymatic scoring of the CpG loci. Locus discrimination is provided by a combination of sequence-specific hybridization capture and allele-specific, single-base primer extension. One or two probes are used to interrogate CpG locus, depending on the probe design for a particular CpG site. (The Infinium I design has two probes per site and Infinium II has one probe per site.) The 3' end of the primers are positioned directly across from the CpG site (for Infinium I) or immediately adjacent to the site (for Infinium II).

Allele-specific single base Extension of the primer incorporates a biotin nucleotide or a dinitrophenyl labeled nucleotide. (C and G nucleotides are biotin labeled; A and T nucleotides are dinitrophenyl labeled.) Signal amplification of the incorporated label further improves the overall signal-to-noise ratio of the assay.

The Illumina Infinium HD Methylation Assay offers:

- High multiplexing
- Single tube amplification—single chip—no PCR
- Minimal risk of carryover contamination
- Low DNA input into bisulfite-conversion—500 ng for manual process or 1000 ng for automated process
- Walk-away automation using Tecan Genesis or Freedom EVO Robots and Tecan GenePaint system
- Compatibility with both Illumina iScan™ and HiScan™ Systems
- Multiple-Sample (12-) BeadChip format

Audience and Purpose

This guide is for laboratory technicians running the Illumina Infinium HD Methylation Assay. The guide documents the laboratory protocols associated with the assay. Follow all of the protocols in the order shown.

Chapter 2 Manual Protocol explains how to run the assay manually in the lab.

Chapter 3 Automated Protocol explains how to automate the protocol with the aid of the Tecan eight-tip robot.

Important Note

Before following any of the procedures in this guide, read the SOP, which explains how to equip and run an Illumina Infinium HD Methylation Assay laboratory. The guide includes important information on the following topics:

- Prevention of amplification product contamination
- Safety precautions
- Equipment, materials, and reagents
- Standard lab procedures
- Robot use
- BeadChip imaging
- System maintenance
- GenomeStudio™ controls
- Troubleshooting

The instructions apply equally to all whole-genome genotyping and methylation chips provided by Illumina. All of the Illumina Infinium HD Methylation Assay Protocol guides assume that you have already set up the laboratory space and are familiar with the standard procedures and safety precautions.

Illumina Infinium HD Methylation Assay

This section describes and illustrates the assay protocol. The assay requires only 500 ng of gDNA for the manual assay or 1000 ng of gDNA for the automated assay input into bisulfite conversion.

Bisulfite Conversion (Pre-AMP)

DNA is treated with sodium bisulfite using an Illumina-specified bisulfite conversion kit. After the treatment, unmethylated cytosines convert to uracil, while methylated cytosines remain unchanged.

Figure 1 Bisulfite Conversion

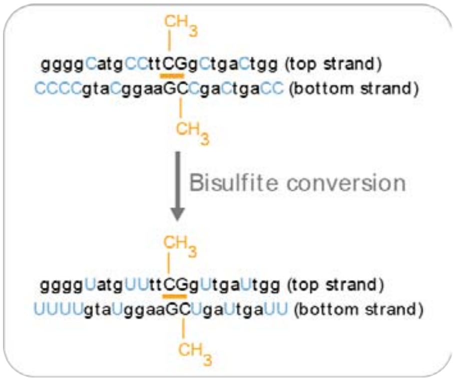

See *Make BCD Plate* on page 35 for manual processing. See *Make BCD Plate* on page 131 for automated processing.

Amplify BCD (Pre-AMP)

The BCD samples are denatured and neutralized to prepare them for amplification.

Figure 2 Denaturing and Neutralizing BCD

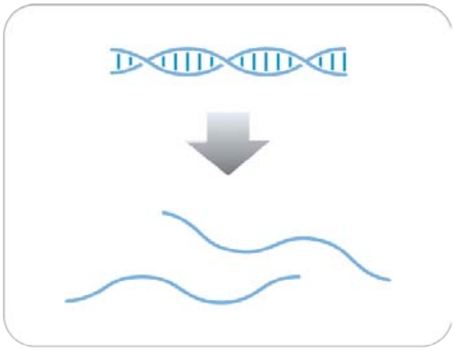

See *Make MSA4 Plate* on page 41 for manual processing. See *Make MSA4 Plate* on page 137 for automated processing.

Incubate DNA

The denatured DNA is isothermally amplified in an overnight step. The whole-genome amplification uniformly increases the amount of the DNA sample by several thousand fold without introducing large amounts of amplification bias.

Figure 3 Incubating DNA to Amplify

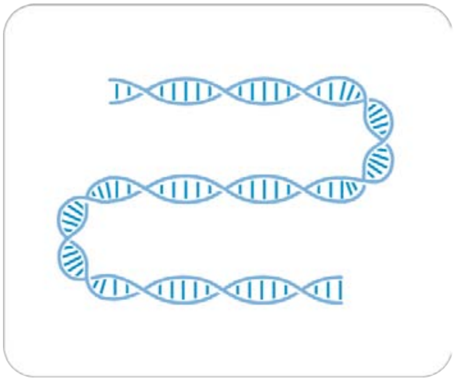

See *Incubate MSA4 Plate* on page 46 for manual processing. See *Incubate MSA4 Plate* on page 144 for automated processing.

Fragment DNA (Post-AMP)

The amplified product is fragmented by a controlled enzymatic process that does not require gel electrophoresis. The process uses end-point fragmentation to avoid overfragmenting the sample.

Figure 4 Fragmenting DNA

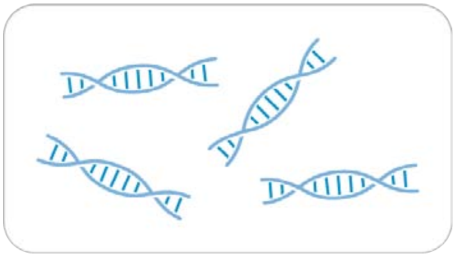

See *Fragment MSA4 Plate* on page 47 for manual processing. See *Fragment MSA4 Plate* on page 145 for automated processing.

Precipitate DNA (Post-AMP)

After an isopropanol precipitation, the fragmented DNA is collected by centrifugation at 4° C.

Figure 5 Precipitating DNA

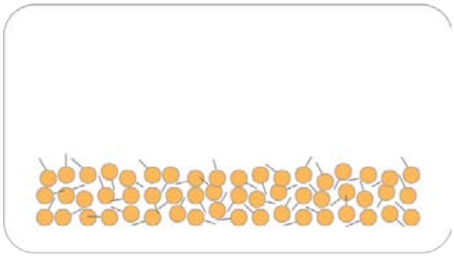

See *Precipitate MSA4 Plate* on page 50 for manual processing. See *Precipitate MSA4 Plate* on page 150 for automated processing.

Resuspend DNA (Post-AMP)

The precipitated DNA is resuspended in hybridization buffer.

Figure 6 Resuspending DNA

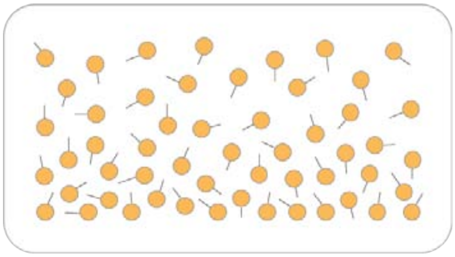

See *Resuspend MSA4 Plate* on page 55 for manual processing. See *Resuspend MSA4 Plate* on page 157 for automated processing.

Hyb Multi BeadChip (Post-AMP)

Dispense the fragmented, resuspended DNA samples onto BeadChips. Incubate the BeadChips in the Illumina Hybridization Oven to hybridize the samples onto the BeadChips.

Twelve samples are applied to each BeadChip, which keeps them separate with an IntelliHyb seal. The prepared BeadChip is incubated overnight in the Illumina Hybridization Oven. The amplified and fragmented DNA samples anneal to locus-specific 50mers (covalently linked to one of over 500,000 bead types) during hybridization. Two bead types correspond to each CpG locus for Infinium I assays: one bead type corresponds to methylated (C), another bead type to unmethylated (T) state of the CpG site. One beadtype corresponds to each CpG locus for Infinium II assays.

Figure 7 Hybridize to BeadChip (Post-AMP)

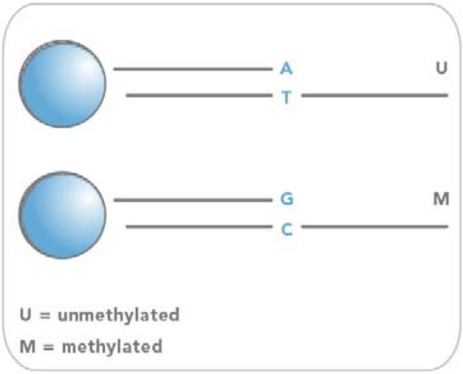

See *Hybridize Multi BeadChip* on page 58 for manual processing. See *Hybridize Multi BeadChip* on page 162 for automated processing.

Wash BeadChip (Post-AMP)

Unhybridized and non-specifically hybridized DNA is washed away, and the chip is prepared for staining and extension.

Figure 8 Washing BeadChip

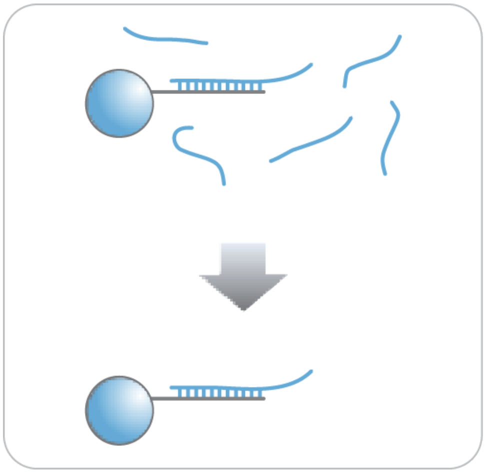

See *Wash BeadChip* on page 78 for manual processing. See *Wash BeadChip* on page 180 for automated processing.

Extend and Stain (XStain) BeadChip (Post-AMP)

Unhybridized and non-specifically hybridized DNA is washed away. The chip undergoes extension and staining in capillary flow-through chambers. Single-base extension of the oligos on the BeadChip, using the captured DNA as a template, incorporates detectable labels on the BeadChip and determines the methylation level of the query CpG sites.

Figure 9 Extending and Staining BeadChip

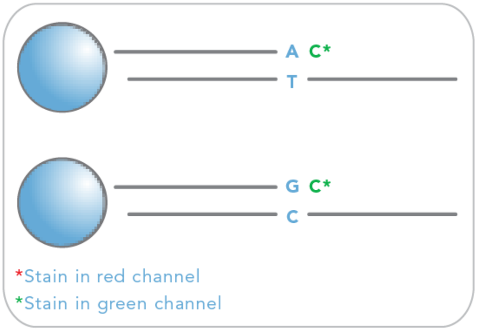

See *Single-Base Extension and Stain BeadChip* on page 90 for manual processing. See *Single-Base Extension and Stain BeadChip* on page 192 for automated processing.

Image BeadChip

The Illumina iScan or HiScan Systems scan the BeadChip, using a laser to excite the fluorophore of the single-base extension product on the beads. The scanner records high-resolution images of the light emitted from the fluorophores. See the chapter on imaging BeadChips in the SOP.

Figure 10 Imaging BeadChip

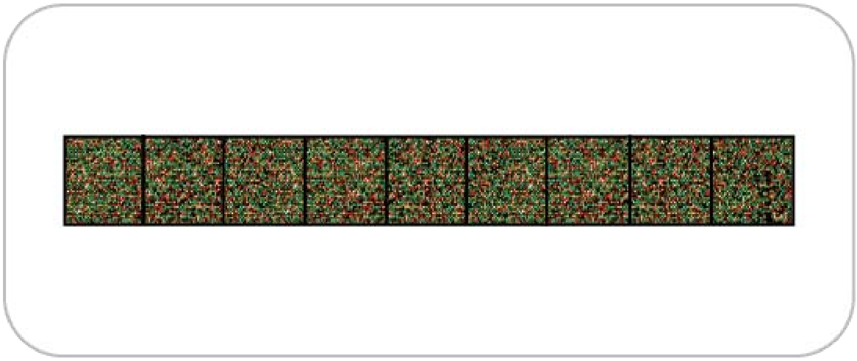

Image BeadChip on the iScan System

The iScan™ Reader is an easy-to-use, laser-based, high-resolution benchtop optical imaging system that can rapidly scan and collect large volumes of data from Illumina DNA analysis and RNA analysis high-density BeadChips.

Image BeadChip on the HiScan System

The HiScan™ System is an easy-to-use, laser-based, high-resolution benchtop optical imaging system that integrates the high-throughput capability of genotyping and gene expression arrays with the power and resolution of next-generation sequencing, delivering unprecedented flexibility for experimental design.

Scanning Settings for Imaging BeadChips on the HiScan and iScan Systems

Use the **Methylation NXT** setting when scanning BeadChips using the HiScan or iScan systems. For general information about scan settings, see the *iScan System User Guide* or *HiScanSQ System User Guide*.

Infinium HD Methylation BeadChip

Illumina Infinium HD BeadChips are sophisticated silicon-based array devices. The sample sections of the slide are separated by an IntelliHyb® seal so that you can run multiple samples simultaneously.

Each individual sample section may hold thousands of different oligonucleotide probe sequences. These are in turn attached to beads assembled into the microwells of the BeadChip substrate. Because the microwells outnumber the distinct bead types, multiple copies of each bead type are present in the array. This built-in redundancy improves robustness and measurement precision. The BeadChip manufacturing process includes hybridization-based quality controls of each array feature, allowing consistent production of high-quality, reproducible arrays.

Illumina Lab Protocols

Illumina lab protocols are designed to promote efficiency and minimize the risk of contamination. The SOP documents standard operating procedures and tools for an Infinium assay lab and explains how to set up and maintain separate pre- and post-amplification areas. Familiarize yourself with this guide before performing any Infinium assays.

Chapter 2 Manual Protocol and Chapter 3 Automated Protocol show how to perform the assay protocol with clearly divided pre- and post-amplification processes using a manual and automated process respectively.

Tracking Tools

Illumina provides the following tools for sample tracking and guidance in the lab:

- **Experienced User Cards** to guide you through the protocols. There are separate sets of cards for the manual and automated processes.
- **Lab Tracking Form** to map DNA samples to BeadChips and record the barcode of each reagent and plate used in the protocol.
- **Sample Sheet template** to record information about your samples for later use in data analysis.

All of these documents are available for printing and reference at http://www.illumina.com/documentation.

Sample Sheet

To effectively track your samples and assay, Illumina recommends you create a Sample Sheet. The Sample Sheet will later be used by the GenomeStudio application for data analysis. For instructions on data analysis, see the *GenomeStudio Methylation Module User Guide*.

Create your Sample Sheet according to the guidelines provided in the table below.

Table 1 Sample Sheet Guidelines

	Description	Optional (O) or Required (R)
**Sample_Name**	Example: S12345<br>If not user-specified, the GenomeStudio application will assign a default sample name, concatenating the sample plate and sample well names.	R
**Sample_Plate**	Example: GS0005623-BCD<br>User-specified name for the plate containing bisulfite-converted DNA samples.	O

	Description	Optional (O) or Required (R)
**Sample_Well**	Example: A01<br>The well containing the specific sample in the MSA4 plate.	O
**Sample_Group**	Example: Group 1<br>User-specified name of the sample group. If the Sample_ Group is missing, GenomeStudio creates one group and assigns it a default name.	R
**Pool_ID**	N/A	N/A
**Sentrix_ID**	Example: 1529221001<br>BeadChip ID.	R
**Sentrix_Position**	R01C01, R01C02, etc. - position of the sample on the Bead Chip	R
**Notes**	Your sample sheet header may contain whatever information you choose.<br>Your sample sheet may contain any number of columns you choose.<br>Your sample sheet must be in a comma-delimited (.csv) file format. Commas in the Sample sheet are not allowed.<br>Save the sample sheet under any name you wish; for example, the user-defined experiment name.	

The following figure provides an example of the Sample Sheet format. Product documentation includes an electronic, read-only Sample Sheet template file (Sample Sheet Template.csv) that you can copy and use.

Figure 11 Sample Sheet Example

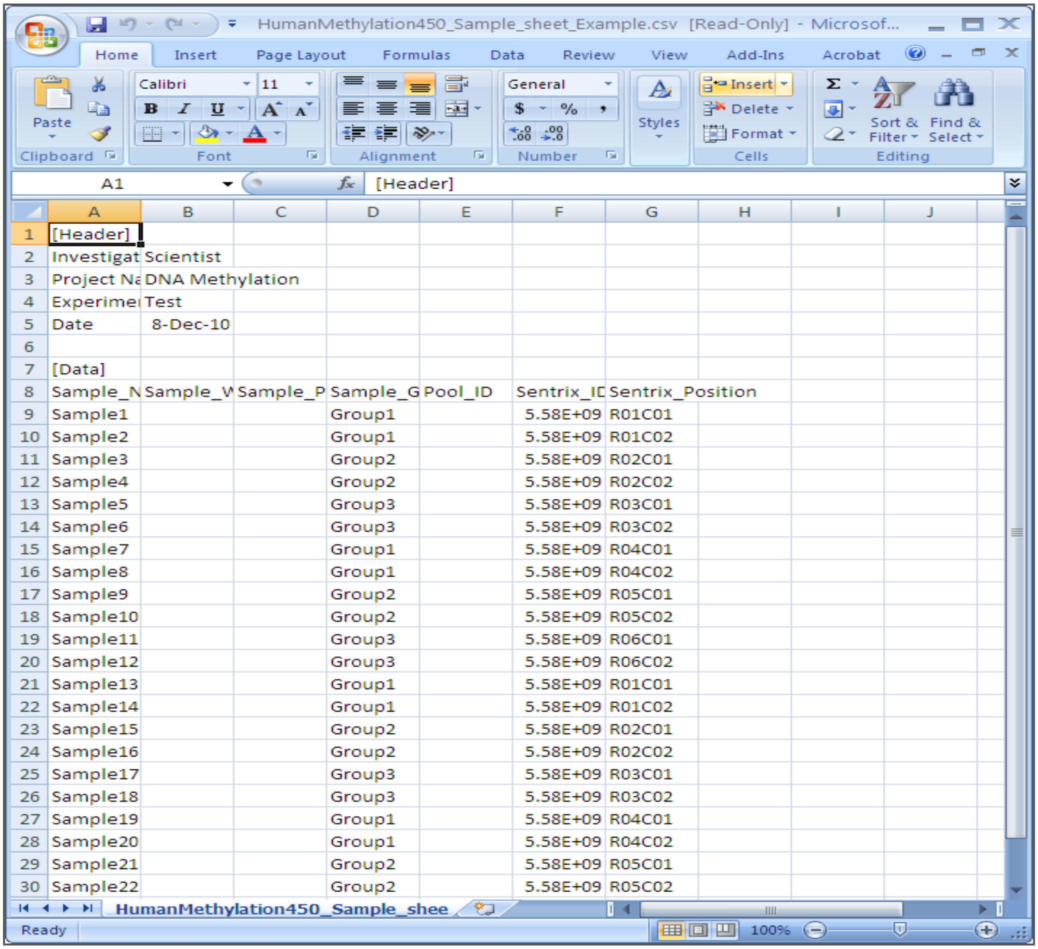

	A	B	C	D	E	F	G
1	[Header]						
2	Investigat	Scientist					
3	Project Na	DNA Methylation					
4	Experimer	Test					
5	Date	8-Dec-10					
6							
7	[Data]						
8	Sample_N	Sample_W	Sample_P	Sample_G	Pool_ID	Sentrix_ID	Sentrix_Position
9	Sample1			Group1		5.58E+09	R01C01
10	Sample2			Group1		5.58E+09	R01C02
11	Sample3			Group2		5.58E+09	R02C01
12	Sample4			Group2		5.58E+09	R02C02
13	Sample5			Group3		5.58E+09	R03C01
14	Sample6			Group3		5.58E+09	R03C02
15	Sample7			Group1		5.58E+09	R04C01
16	Sample8			Group1		5.58E+09	R04C02
17	Sample9			Group2		5.58E+09	R05C01
18	Sample10			Group2		5.58E+09	R05C02
19	Sample11			Group3		5.58E+09	R06C01
20	Sample12			Group3		5.58E+09	R06C02
21	Sample13			Group1		5.58E+09	R01C01
22	Sample14			Group1		5.58E+09	R01C02
23	Sample15			Group2		5.58E+09	R02C01
24	Sample16			Group2		5.58E+09	R02C02
25	Sample17			Group3		5.58E+09	R03C01
26	Sample18			Group3		5.58E+09	R03C02
27	Sample19			Group1		5.58E+09	R04C01
28	Sample20			Group1		5.58E+09	R04C02
29	Sample21			Group2		5.58E+09	R05C01
30	Sample22			Group2		5.58E+09	R05C02

Tecan GenePaint

The Illumina Infinium HD Methylation Assay uses Tecan's GenePaint automated slide processor to process BeadChips. The GenePaint system employs a capillary gap flow-through chamber to enable reagent entrapment and exchange over the BeadChip's active surface. Washing, blocking, extension, and signal amplification are all performed by simple reagent additions to the flow cell. Addition of a new reagent displaces the entrapped reagent from the flow cell. For maximum flexibility, these additions can be performed either manually or via the Tecan Genesis or Tecan Freedom Evo robots. The optional automated robotic processing and single-use reagent tube barcoding assure maximum consistency from slide to slide.

CAUTION
Do not run any other programs or applications while using the Tecan robot. Your computer and the robot may lock up and stop a run.

iScan, HiScan, and AutoLoader2

BeadChips are imaged using either the Illumina iScan System or HiScan System. Both of these are two-channel high-resolution laser imagers that scan BeadChips at two wavelengths simultaneously and create an image file for each channel (i.e., two per array).

The iScan Control Software determines intensity values for each bead type and creates data files for each channel. GenomeStudio uses this data file in conjunction with the individual bead pool map (*.bpm) to analyze the data from the assay.

Loading and unloading the iScan System or HiScan System can be automated with the optional AutoLoader2. AutoLoaders support unattended processing by placing BeadChips carriers in the imaging system's tray, so that it can scan the BeadChips. Features include those listed below:

Table 2 AutoLoader2 Features

Feature	AutoLoader2
Integrated with iScan and HiScan Control Software	•
Email alert system	•
Single-reader or dual-reader configuration	•
Number of BeadChips supported per carrier	4
Number of carriers processed at a time	48

Illumina GenomeStudio, Illumina's integrated data analysis software platform, provides a common environment for analyzing data obtained from microarray and sequencing technologies. Within this common environment, or framework, the Illumina GenomeStudio software modules allow you to perform application-specific analyses. The Illumina GenomeStudio Methylation Module, included with your Illumina Infinium Methylation Assay system, is an application for extracting genome-wide DNA methylation data from data files collected from systems such as the Illumina HiScan Reader. Experiment performance is based on built-in controls that accompany each experiment.

Data analysis features of the Illumina GenomeStudio Methylation Module include:

- Choice of assay analysis within a single application
- Data tables for information management and manipulation
- Plotting and graphing tools
- Whole-genome display of sample data in the IGV (Illumina Genome Viewer)
- Data visualization of one or more samples in the ICB (Illumina Chromosome Browser)
- Data normalization
- Custom report file formats
- Differential methylation analysis
- Assay-specific controls dashboards

For feature descriptions and instructions on using the Illumina GenomeStudio platform to visualize and analyze genome-wide DNA methylation data, see the *GenomeStudio Framework User Guide* and the *GenomeStudio Methylation Module User Guide*.

For technical assistance, contact Illumina Technical Support.

Manual Protocol

Chapter 2

Introduction to Infinium HD Methylation Manual Protocol 20
Infinium HD Methylation Manual Workflow 21
Equipment, Materials, and Reagents 23
Quantitate DNA (Optional) 27
Make BCD Plate 35
Make MSA4 Plate 41
Incubate MSA4 Plate 46
Fragment MSA4 Plate 47
Precipitate MSA4 Plate 50
Resuspend MSA4 Plate 55
Hybridize Multi BeadChip 58
Wash BeadChip 78
Single-Base Extension and Stain BeadChip 90
Image BeadChip 110
Illumina GenomeStudio 112

Introduction to Infinium HD Methylation Manual Protocol

This chapter describes pre- and post-amplification manual laboratory protocols for the Illumina Infinium HD Methylation Assay. Follow the protocols in the order shown.

Infinium HD Methylation Manual Workflow

The following figure graphically represents the Illumina Infinium HD Methylation Assay manual workflow for the Infinium Methylation BeadChips. These protocols describe the procedure for preparing 96 DNA samples.

Figure 12 Illumina Infinium HD Methylation AssayManual Workflow

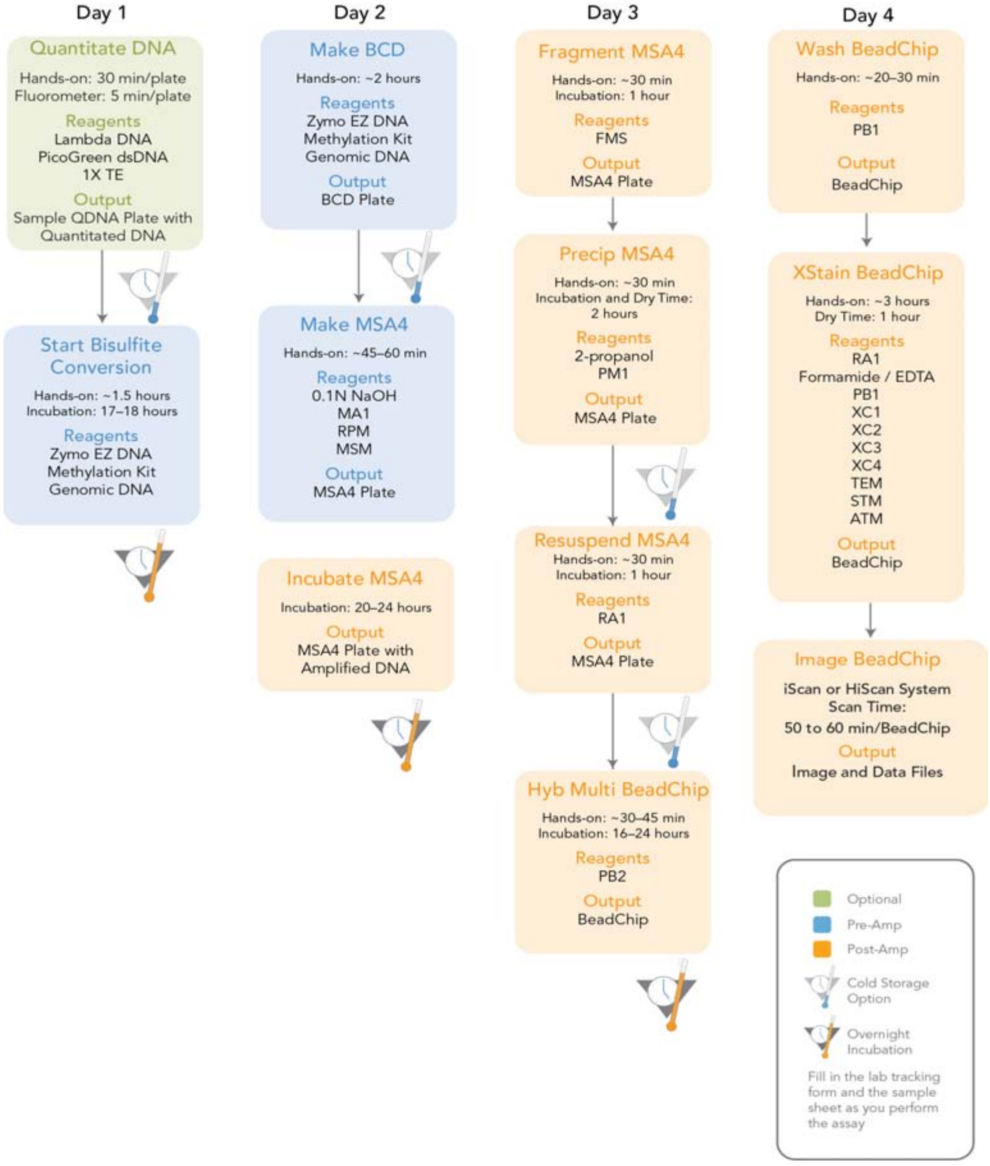

Equipment, Materials, and Reagents

These materials are specific to the manual Illumina Infinium HD Methylation Assay. For a list of other equipment, materials, and reagents needed in an Illumina Infinium HD Methylation Assay lab, see the SOP.

User-Supplied Equipment

Table 3 User-Supplied Equipment

Item	Catalog #
Forceps	VWR Catalog # 25601-008
Auto-desiccator cabinet (Optional—allows scanning of BeadChips up to three days after processing)	VWR Catalog # 74950-342

Illumina-Supplied Equipment

Table 4 Illumina-Supplied Equipment

Item	Catalog #
Multi-Sample BeadChip Alignment Fixture	Illumina part # 218528

User-Supplied Materials

Table 5 User Supplied Materials

Item	Catalog #
Robots	Tecan
Tube vortexer	N/A
Tube rack	VWR, International
Combination optical tachometer/stroboscope	Cole-Parmer; cat# A-87700-06; www.coleparmer.com
Microtiter plate centrifuge with g-force range 8-3000xg, for dedicated pre- and post-AMP use	N/A
Adaptor for centrifuge plates and tubes	N/A
Pipets (two separate sets)	2 each of P-20, P-200, and P-1000
8-channel precision pipet (two separate sets)	50 µL to 300uL
Stop watch/timer	N/A
Forceps	VWR International; cat# 25601-008; www.vwr.com
Powder-free gloves (two separate stocks)	N/A
Lab coats (separate pre-PCR and post-PCR)	N/A
Safety glasses (two separate stocks)	N/A
15 mL conical tubes	N/A
96-well 0.2 mL skirted microplates	MJ Research; cat# MSP-9601; www.mjr.com
0.8 mL storage plate (MIDI plate), conical well bottom	Abgene; cat#AB-0765; www.abgene.com
Heat Sealing foil sheets, Thermo-Seal	Abgene; cat# AB-0559; www.abgene.com

Item	Catalog #
96-well cap mats (piercable, nonautoclavable)	Abgene; cat# AB-0566; www.abgene.com
Absorbent pads	N/A
Kimwipes®	N/A
Mild detergent, such as Alconox® Powder	VWR, International; cat# 21835-032; www.vwr.com
Detergent	N/A
Aerosol filter pipet tips (two separate stocks)	20uL, 200uL, 1000uL
Disposable pipetting troughs	VWR; cat# 21007-970
Reservoir, full, 150 mL	Beckman Coulter, Inc.; cat# 372784
Reservoir, half, 75 mL	Beckman Coulter, Inc.; cat# 372786; www.beckman.com
Reservoir, quarter, 40 mL	Beckman Coulter, Inc.; cat#372790; www.beckman.com
Reservoir frame	Beckman Coulter, Inc.; cat# 372795; www.beckman.com

Illumina-Supplied Materials

- WG#-MSA4 barcode labels
- WG#-BCD barcode labels
- WG#-DNA barcode labels

Illumina-Supplied Reagents

Table 6 Illumina-Supplied Reagents

Item	Part #
**ATM**—Anti-Stain Two-Color Master Mix	11208317
**FMS**—Fragmentation solution	11203428
**MA1**—Multi-Sample Amplification 1 Mix	11202880
**RPM**—Random Primer Mix	15010230
**MSM**—Multi-Sample Amplification Master Mix	11203410
**PB1**—Reagent used to prepare BeadChips for hybridization	11291245
**PB2**—Humidifying buffer used during hybridization	11191130
**PM1**—Precipitation solution	11292436
**RA1**—Resuspension, hybridization, and wash solution	11292441
**STM**—Superior Two-Color Master Mix	11288046
**TEM**—Two-Color Extension Master Mix	11208309
**XC1**—XStain BeadChip solution 1	11208288
**XC2**—XStain BeadChip solution 2	11208296
**XC3**—XStain BeadChip solution 3	11208392
**XC4**—XStain BeadChip solution 4	11208430

Quantitate DNA (Optional)

Illumina recommends the Molecular Probes PicoGreen assay to quantitate dsDNA samples. The PicoGreen assay can quantitate small DNA volumes and measures DNA directly. Other techniques may pick up contamination such as RNA and proteins. Illumina recommends using a spectrofluorometer because fluorometry provides DNA-specific quantification. Spectrophotometry might also measure RNA and yield values that are too high.

Estimated Time

Hands-on time: ~20 minutes per plate plus 10 minutes to prepare the PicoGreen

Spectrofluorometer read time: ~5 minutes per plate

Consumables

Item	Quantity	Storage	Supplied By
PicoGreen dsDNA quantitation reagent	See Instructions	2° to 8° C	User
1X TE	See Instructions	Room temperature	User
Lambda DNA	See Instructions	2° to 8° C	User
96-well 0.65 ml microtiter plate	1 per 96 samples		User
Fluotrac 200 96-well flat-bottom plate	1 per Std DNA plate<br>1 per Sample DNA plate		User

Preparation

- Thaw PicoGreen to room temperature for 60 minutes in a light-impermeable container.
- Hand-label the microtiter plate "Standard DNA."

- Hand-label one of the Fluotrac plates “Standard QDNA.”
- Hand-label the other Fluotrac plate “Sample QDNA.” This plate will contain the quantitated DNA.
- In the Sample Sheet, enter the Sample_Name (optional) and Sample_Plate for each Sample_Well.

Make Standard DNA Plate

In this process, you create a Standard DNA plate with serial dilutions of stock Lambda DNA in the wells of column 1.

1 Add stock Lambda DNA to well A1 in the plate labeled “Standard DNA” and dilute it to 75 ng/µl in a final volume of 233.3 µl. Pipette up and down several times.

   a Use the following formula to calculate the amount of stock Lambda DNA to add to A1:

(233.3 µl) X (75 ng/µl) (stock Lambda DNA concentration)	=	µl of stock Lambda DNA to add to A1

   b Dilute the stock DNA in well A1 using the following formula:
   µl of 1X TE to add to A1 = 233.3 µl - µl of stock Lambda DNA in well A1

2 Add 66.7 µl 1X TE to well B1.

3 Add 100 µl 1X TE to wells C, D, E, F, G, and H of column 1.

Figure 13 Dilution of Stock Lambda DNA Standard

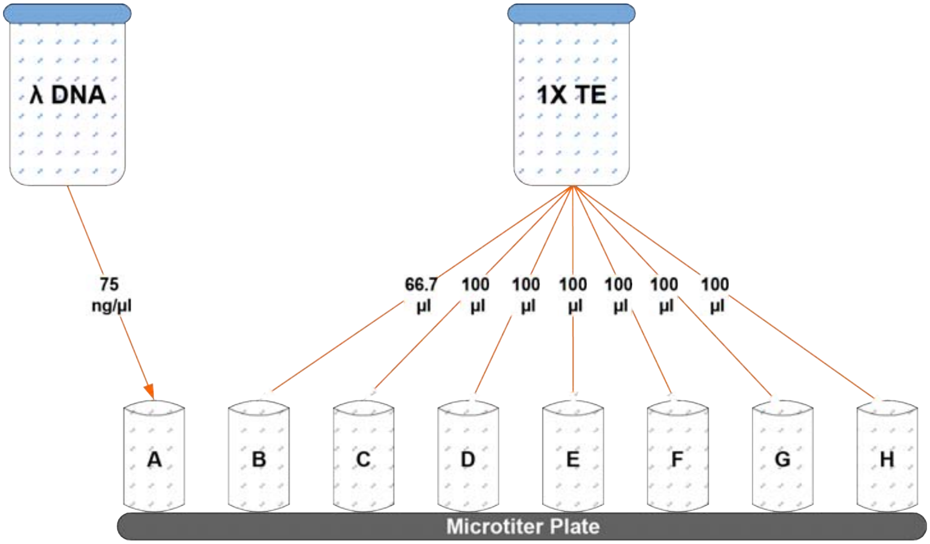

4 Transfer 133.3 µl of Lambda DNA from well A1 into well B1. Pipette up and down several times.

5 Change tips. Transfer 100 µl from well B1 into well C1. Pipette up and down several times.

6 Repeat for wells D1, E1, F1, and G1, changing tips each time. **Do not transfer from well G1 to H1.** Well H1 serves as the blank 0 ng/µl Lambda DNA.

Table 7 Concentrations of Lambda DNA

Row-Column	Concentration (ng/µl)	Final Volume in Well (µl)
A1	75	100
B1	50	100
C1	25	100
D1	12.5	100

Row-Column	Concentration (ng/µl)	Final Volume in Well (µl)
E1	6.25	100
F1	3.125	100
G1	1.5262	200
H1	0	100

Figure 14 Serial Dilutions of Lambda DNA

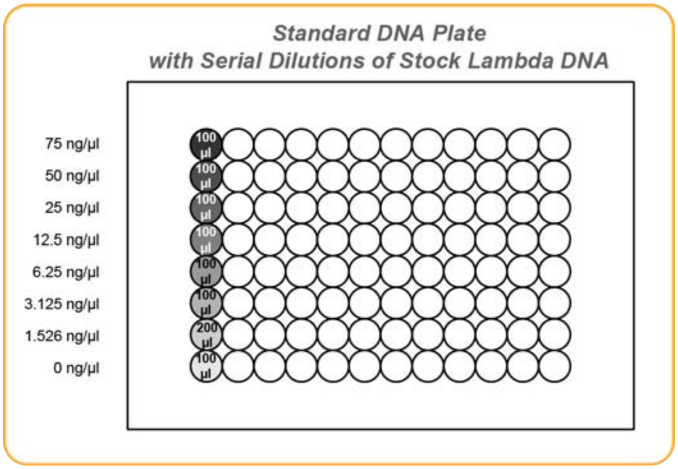

7 Cover the Standard DNA plate with cap mat.

Dilute PicoGreen

The diluted PicoGreen will be added to both the Standard QDNA and Sample QDNA plates, to make the DNA fluoresce when read with the spectrofluorometer.

CAUTION
PicoGreen reagent degrades quickly in the presence of light. Also, do not use glass containers for PicoGreen reagent, because it adheres to glass, thereby lowering its effective concentration in solution and effecting the upper response range accuracy.

1 Prepare a 1:200 dilution of PicoGreen into 1X TE, using a sealed 100 ml or 250 ml Nalgene bottle wrapped in aluminum foil.
Refer to the following table to identify the volumes needed to produce diluted reagent for multiple 96-well QDNA plates. For fewer than 96 DNA samples, scale down the volumes.

Table 8 Volumes for PicoGreen Reagents

# QDNA Plates	PicoGreen Volume (µl)	1X TE Volume (ml)
1	115	23
2	215	43
3	315	63

2 Cap the foil-wrapped bottle and vortex to mix.

Create QDNA Standard Plate with Diluted PicoGreen

In this process you transfer the serial dilutions from the Standard DNA plate into the Standard QDNA Fluotrac plate and add diluted PicoGreen.

1 Pour the PicoGreen/1X TE dilution into a clean reagent reservoir.

2 Using a multichannel pipette, transfer 195 µl PicoGreen/1X TE dilution into each well of columns 1 and 2 of the Fluotrac plate labeled “Standard QDNA”.

3 Add 2 µl of each stock Lambda DNA dilution from the Standard DNA plate to columns 1 and 2 of the Standard QDNA Fluotrac plate.

Figure 15 Standard QDNA Plate with PicoGreen

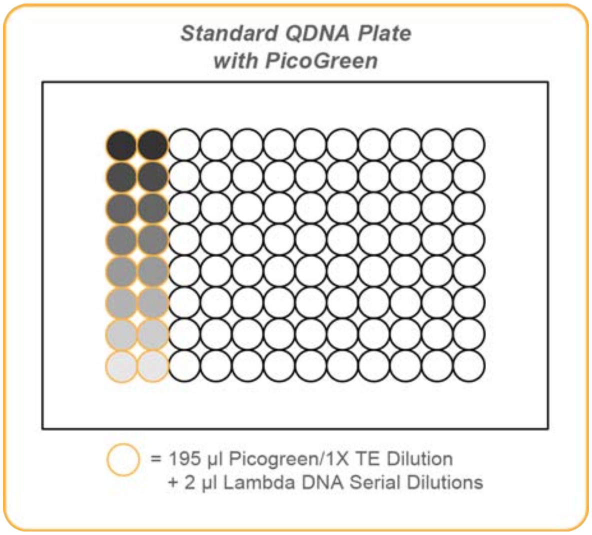

4 Immediately cover the plate with an adhesive aluminum seal.

Prepare QDNA Sample Plate with PicoGreen and DNA

In this process, you create a new Sample QDNA plate that contains DNA sample and PicoGreen.

1 Using a multichannel pipette, transfer 195 µl PicoGreen/1X TE dilution into each well of columns 1 and 2 of the Fluotrac plate labeled “Sample QDNA”.

2 Add 2 µl of DNA sample to all 96 wells of the Sample QDNA plate. Only the first two columns will also contain PicoGreen/1X TE dilution.

Figure 16 Sample QDNA Plate with PicoGreen

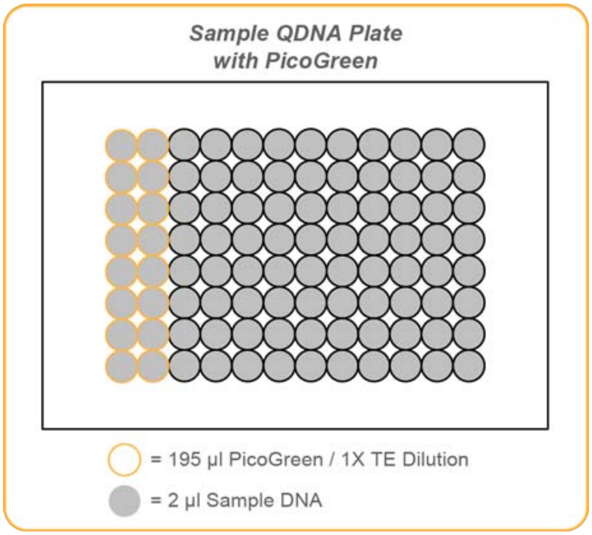

3 Immediately cover the plate with an adhesive aluminum seal.

Read QDNA Plate

In this process, you use the Gemini XS or XPS Spectrofluorometer to read the Standard QDNA and Sample QDNA plates. The spectrofluorometer creates a standard curve from the known concentrations in the Standard QDNA plate, which you use to determine the concentration of DNA in the Sample QDNA plates. For the best performance, Illumina recommends a minimum concentration of 50 ng/µl.

NOTE
The SoftMax Pro screens and menu options may vary from those depicted, depending up on the software version that you are running.

1 Turn on the spectrofluorometer. At the PC, open the SoftMax Pro program.

2 Load the Illumina QDNA.ppr file from the installation CD that came with your system.

3 Select **Protocols | GTS_QDNA**.

4 Place the Standard QDNA Fluotrac Plate into the spectrofluorometer loading rack with well A1 in the upper left corner.

5 Click the blue arrow next to **Illumina QDNA | SQDNA_STD**.

6 Click **Read** in the SoftMax Pro interface to begin reading the Standard QDNA Plate.

7 When the software finishes reading the data, remove the plate from the drawer.

8 Click the blue arrow next to **Standard Curve** to view the standard curve graph.

9 If the standard curve is acceptable, continue with the sample plate. Otherwise, click **Standard Curve** again.

10 Place the first Sample QDNA plate in the spectrofluorometer with well A1 in the upper left corner.

11 Click the blue arrow next to **SQDNA** and click **Read**.

12 When the software finishes reading the plate, remove the plate from the drawer.

13 Repeat steps 10 through 12 to quantitate all Sample QDNA plates.

14 Once all plates have been read, click **File | Save** to save the output data file (*.pda).

15 When you have saved the *.pda file, click **File | Import/Export | Export** and export the file as a *.txt file. You can open the *.txt file in Microsoft Excel for data analysis.

16 Do one of the following:
- Proceed to *Make BCD Plate* on page 35.
- Store the quantitated DNA at 2° to 8° C for up to one month.

Make BCD Plate

This process uses the Zymo EZ DNA Methylation Kit to convert unmethylated cytosines (C) in genomic DNA to uracil (U), while leaving methylated cytosines (C) unchanged for methylation analysis.

Figure 17 Bisulfite Conversion

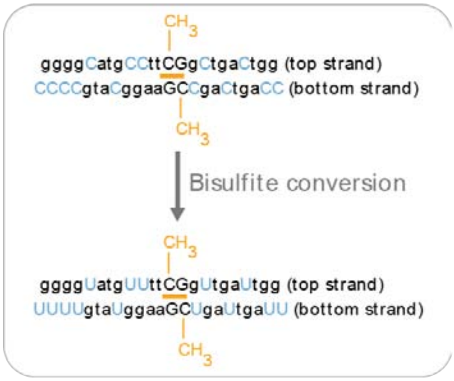

Methylation detection in bisulfite-converted DNA (BCD) is based on the different sensitivity of cytosine and 5-methylcytosine to deamination by bisulfite. Under acidic conditions, cytosine undergoes conversion to uracil, while methylated cytosine remains unreactive. An effective bisulfite-conversion protocol is a necessary prerequisite for a successful Illumina Infinium HD Methylation Assay. Incomplete conversion of cytosine to uracil can result in false-positive methylation signals, and can reduce the overall quality of the assay data.

NOTE
Always perform bisulfite conversion of DNA in the Pre-AMP area.

Figure 18 Bisulfite Conversion Workflow

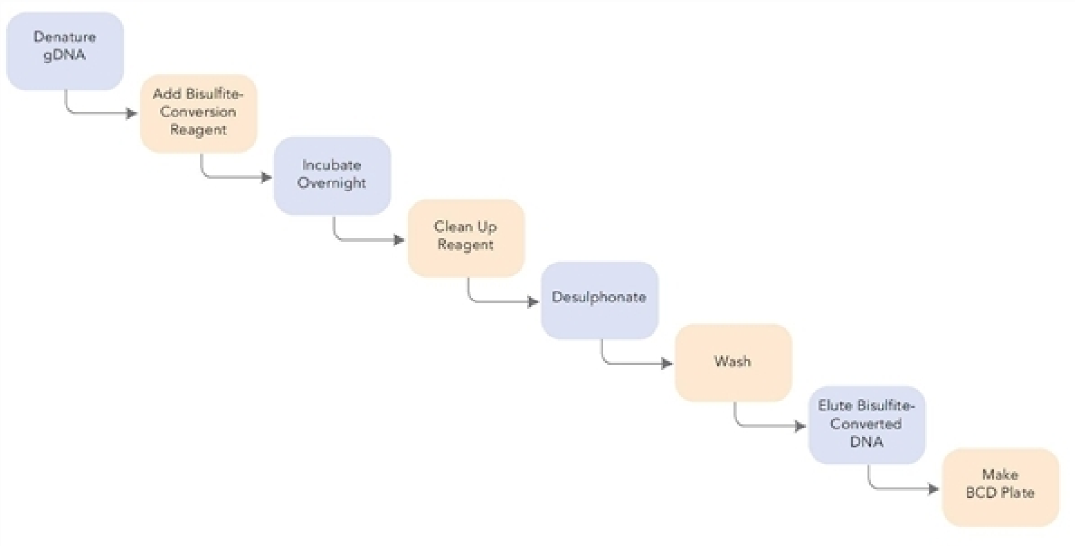

Bisulfite-convert the genomic DNA samples using the Zymo EZ DNA Methylation Kit. Transfer the bisulfite-converted samples to the BCD plate.

Estimated Time

Hands-on time:

- ~1.5 hours on Day 1
- ~2 hours on Day 2

Incubation time: 16–17 hours on Day 1 (Overnight)

Consumables

Item	Quantity	Storage	Supplied By
Zymo EZ DNA Methylation kit (includes bisulfite-conversion reagent, dilution buffer, desulphonation buffer, elution buffer)	1 kit per 50 samples<br>1 kit per 200 samples<br>or<br>1 kit per 2 plates	Room temperature	User
96-well 0.2 ml skirted microplate	1–3 plates		User
Genomic DNA	≥ 500 ng for each bisulfite conversion reaction for manual process.<br><br>≥1000 ng for each bisulfite conversion reaction for the Automated process		User

Illumina recommends using the Zymo Research EZ DNA methylation kit for bisulfite conversion of genomic DNA. Follow Illumina recommended incubation conditions during bisulfite conversion to maximize DNA conversion rate.

For all other steps, follow the manufacturer's instructions for use, because the protocols vary significantly for different kits.

CAUTION
Bisulfite-conversion kits that are not specified in this guide are not supported for use with the Illumina Infinium HD Methylation Assay.

Preparation

Use this table to determine the correct amount of genomic DNA for each bisulfite-conversion process.

Table 9 Genomic DNA Input for Bisulfite Conversion

	Amount of gDNA	Elution Buffer	Number of Activation Reactions Supported
Manual process	> 500 ng	12 µl	Enough for two replicates
Automated process	1000 ng	22 µl	Enough for two replicates

- Prepare the conversion reagent according to the manufacturer's instructions. For best results, use it immediately.

CAUTION
The conversion reagent is photosensitive, so minimize its exposure to light.

- Prepare the wash buffer according to the manufacturer's instructions.
- Apply a BCD barcode label to each new 96-well 0.2 ml skirted microplate.
- On the Lab Tracking Forms, record:
  - Date and time
  - Operator

NOTE
To record information about your assay such as operator information, start and stop times, and barcodes, use the lab tracking form provided at http://www.illumina.com/documentation. This form can be filled out and saved online, or printed and filled in by hand.

Steps to Make BCD Plate

Day 1

The following steps are intended only to provide an overview of the process. For specific instructions, use the manufacturer's documentation.

1 Follow the instructions in the Zymo EZ DNA Methylation Kit to denature the genomic DNA and add conversion reagent.

2 Incubate in a thermocycler using the following conditions for 16 cycles:
  - 95° C for 30 seconds
  - 50° C for 1 hour

NOTE
Denaturation is necessary for bisulfite conversion since the conversion reagent only works on single-stranded DNA.

3 Hold DNA at 4° C for 10 minutes in the thermocycler until you are ready to do the clean-up.

Day 2

1 Follow the instructions in the Zymo EZ DNA Methylation Kit to do the following:
   a Clean the samples using the provided spin columns or filter plate. Wash off the remaining conversion reagent.

NOTE
When using the Zymo EZ DNA Methylation Kit, centrifuge the plate to between 3000 and 5000 xg for optimal performance.

   b Desulphonate the column or plate with desulphonation buffer. Incubate at room temperature for 15 minutes.
   c Clean the samples and wash twice to remove the desulphonation buffer.
   d Add elution buffer:
      — 12 µl for manual process from 500 ng gDNA
      — 22 µl for automated process from 1 µg of gDNA
   e Centrifuge to elute.

NOTE
When using the Zymo EZ DNA Methylation Kit, centrifuge the plate to between 3000 and 5000 xg for 5 minutes for optimal performance.

2 Transfer the bisulfite-converted DNA (BCD) samples to the BCD plate.

3 On the lab tracking form, record the WG#-BCD and BCD plate barcodes.

4 Heat-seal the plate and store it at -15° to -25° C. Thaw the plate completely and vortex to mix contents before using it in an assay.

CAUTION
Do not store BCD for more than one month at -15° to -25° C.

SAFE STOPPING POINT
This is a good stopping point in the process.

Prepare BCD Plate

NOTE
This preparation step should be performed only for the Illumina Infinium HD Methylation Assay.

1 Retrieve your BCD samples and thaw to room temperature.

2 Apply a BCD barcode label to a new 0.8 ml microtiter storage plate (MIDI) or a new 0.2 ml skirted microplate (TCY).

3 Dispense BCD according to the figure shown below:
   - For MIDI plate: 20 µl BCD sample to each well (requires ≥ 1000 ng input in bisulfite conversion).
   - For TCY plate: 10 µl BCD sample to each well (requires ≥ 500 ng input in bisulfite conversion).

Figure 19 BCD Plate Sample Well Distribution

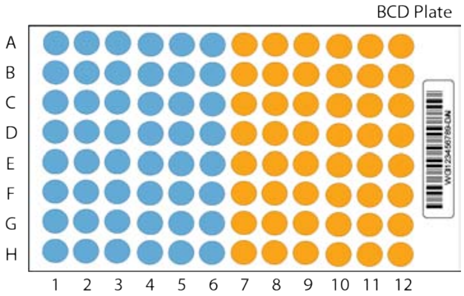

The example shown in this figure applies to 96 samples. For 48 samples, fill the first half of the plate only (blue shaded section).

Make MSA4 Plate

This process creates a MSA4 plate for BCD amplification. MA1 is first added to the MSA4 plate, followed by the BCD samples. Next, the 0.1N NaOH is added to denature the BCD samples. The RPM reagent neutralizes the sample. Lastly, MSM (Multi-Sample Amplification Master Mix) is added to the plate.

Figure 20 Denaturing and Neutralizing BCD

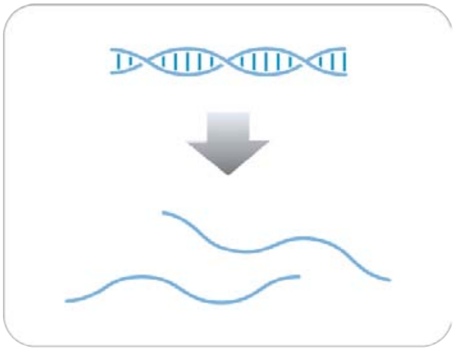

Estimated Time

Hands-on time:

- 45 minutes for 48 samples
- 60 minutes for 96 samples

Incubation time: 20–24 hours

Consumables

Item	Quantity	Storage	Supplied By
MA1	2 tubes (per 96 samples)	Room temperature	Illumina
RPM	2 tubes (per 96 samples)	-15° to -25° C	Illumina
MSM	2 tubes (per 96 samples)	-15° to -25° C	Illumina
0.1N NaOH	5 ml (per 96 samples)	2° to 8° C	User
96-well 0.8 ml microtiter plate (MIDI)	1 plate		User
BCD plate with Bisulfite-converted DNA samples	1 plate	-15° to -25° C	User

NOTE
There are excess reagents provided in a 24-sample kit. Please discard leftover reagents.

NOTE
Thaw all reagents completely at room temperature and allow to equilibrate. Once thawed, gently invert each tube several times to thoroughly mix the reagent. Pulse centrifuge each tube to 280 xg to eliminate bubbles and collect reagent at the bottom of the tube.

Preparation

- In preparation for the Incubate MSA4 process (*Incubate MSA4 Plate* on page 46), pre-heat the Illumina Hybridization Oven in the post-amp area to 37° C and allow the temperature to equilibrate.
- Apply an MSA4 barcode label to a new MIDI plate.
- Thaw MA1, RPM, and MSM tubes to room temperature.

- Thaw BCD samples to room temperature.
- In the Sample Sheet, enter the Sample_Name and Sample_Plate for each Sample_Well.
- On the lab tracking form, record:
  - Date/Time
  - Operator
  - BCD plate barcode
  - MSA4 plate barcode(s)
  - MA1 tube barcode(s)
  - RPM tube barcode(s)
  - MSM tube barcode(s)

NOTE
To record information about your assay such as operator information, start and stop times, and barcodes, use the lab tracking form provided at http://www.illumina.com/documentation. This form can be filled out and saved online, or printed and filled in by hand.

Steps to Make MSA4 Plate

1. If you do not already have a BCD plate, add DNA into either a:
   - MIDI plate: 20 µl to each BCD plate well
   - TCY plate: 10 µl to each BCD plate well
2. Apply a barcode label to the new BCD plate.
3. Dispense 20 µl MA1 into the MSA4 plate wells.
4. Transfer 4 µl of the DNA sample, from the BCD plate to the corresponding wells in the MSA4 plate.
5. On the lab tracking form, record the original DNA sample ID for each well in the MSA4 plate.
6. Dispense 4 µl 0.1N NaOH into each well of the MSA4 plate that contains MA1 and sample.

Refer to the following figure throughout the Make MSA4 process.

Figure 21 Distributing Sample to Wells

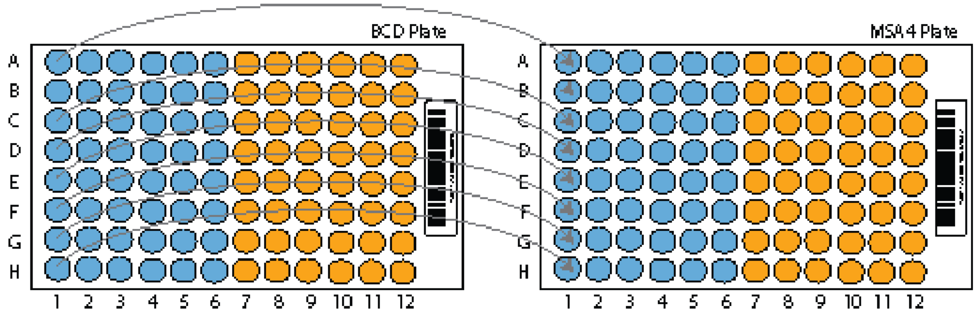

NOTE
To ensure optimal performance, exchange tips between DNA samples and use aerosol filter tips when pipetting DNA.

7 Seal the MSA4 plate with the 96-well cap mat.

CAUTION
Orient the cap mat so that A1 on the cap matches A1 on the plate. To prevent evaporation and spills, which could lead to assay variability and cross-contamination, ensure that all 96 caps are securely seated.

8 Vortex the plate at 1600 rpm for 1 minute.

9 Centrifuge to 280 xg for 1 minute.

10 Incubate for 10 minutes at room temperature.

11 Dispense 68 µl RPM into each well of the MSA4 plate containing sample.

NOTE
To ensure optimal performance, place the tips against the top edge of the wells. Use this technique for all subsequent dispensing steps.

12 Dispense 75 µl MSM into each well of the MSA4 plate containing sample.

13 Seal MSA4 plate with cap mat.

14 Invert the sealed plate at least 10 times to mix contents.

15 Pulse centrifuge to 280 xg for 1 minute.

16 Discard unused reagents in accordance with facility standards.

17 Proceed immediately to the next step.

Incubate MSA4 Plate

This process incubates the MSA4 plate for 20–24 hours at 37° C in the Illumina Hybridization Oven. It generates a sufficient quantity of each individual DNA sample to be used twice in the Illumina Infinium HD Methylation Assay.

Figure 22 Incubating DNA to Amplify

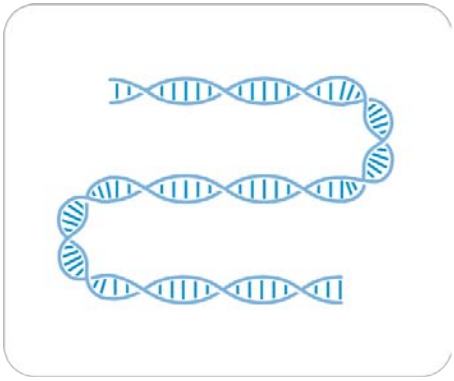

Estimated Incubation Time

20–24 hours

Steps to Incubate MSA4 Plate

1. Incubate MSA4 plate in the Illumina Hybridization Oven for at least 20 but no more than 24 hours at 37° C.
2. On the lab tracking form, record the start and stop times.

NOTE
To record information about your assay such as operator information, start and stop times, and barcodes, use the lab tracking form provided at http://www.illumina.com/documentation. This form can be filled out and saved online, or printed and filled in by hand.

Fragment MSA4 Plate

This process enzymatically fragments the amplified DNA samples. An end-point fragmentation is used to prevent over-fragmentation.

Figure 23 Fragmenting DNA

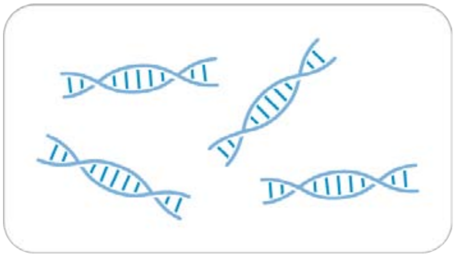

Estimated Time

Hands-on time: ~30 minutes

Incubation time: 1 hour

Consumables

Item	Quantity	Storage	Supplied By
FMS	2 tubes (per 96 samples)	-15° to -25° C	Illumina

NOTE
Thaw all reagents completely at room temperature and allow to equilibrate. Once thawed, gently invert each tube several times to thoroughly mix the reagent. Pulse centrifuge each tube to 280 xg to eliminate bubbles and collect reagent at the bottom of the tube.

Preparation

- Remove the MSA4 plate from the Illumina Hybridization Oven.
- On the lab tracking form, record:
    - Date/Time

- Operator
- FMS tube barcode(s)

NOTE
To record information about your assay such as operator information, start and stop times, and barcodes, use the lab tracking form provided at http://www.illumina.com/documentation. This form can be filled out and saved online, or printed and filled in by hand.

Steps to Fragment MSA4 Plate

1. Centrifuge the plate to 50 xg for 1 minute.
2. Carefully remove the cap mat.
3. When you remove a cap mat, set it aside, upside down, in a safe location for use later in the protocol. When you place the cap mat back on the plate, be sure to match it to its original plate and orient it correctly.
4. Add 50 µl FMS to each well containing sample.
5. Seal the MSA4 plate with the 96-well cap mat.

CAUTION
Orient the cap mat so that A1 on the cap matches A1 on the plate. To prevent evaporation and spills, which could lead to assay variability and cross-contamination, ensure that all 96 caps are securely seated.

6. Vortex the plate at 1600 rpm for 1 minute.
7. Centrifuge the plate to 50 xg for 1 minute.
8. Place the sealed plate on the 37° C heat block for 1 hour.
9. On the lab tracking form, record the start and stop times.
10. Discard unused reagents in accordance with facility standards.
11. Do one of the following:
    - Proceed to *Precipitate MSA4 Plate* on page 50. Leave plate in 37° C heat block until setup is complete.
    - Store the sealed MSA4 plate at -15° to -25° C if you do not plan to proceed to the next step immediately.

SAFE STOPPING POINT
This is a good stopping point in the process.

Precipitate MSA4 Plate

Add PM1 and 2-propanol to the MSA4 plate to precipitate the DNA samples.

Figure 24 Precipitating DNA

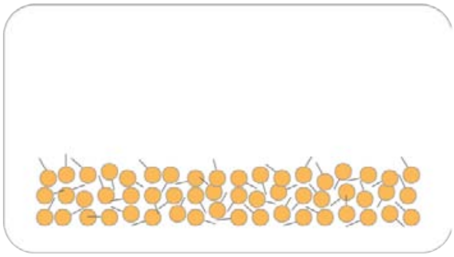

Estimated Time

Hands-on time: ~30 minutes

Incubation and dry time: 2 hours

Consumables

Item	Quantity	Storage	Supplied By
PM1	2 tubes (per 96 samples)	-15° to -25° C	Illumina
100% 2-propanol	40 ml (per 96 samples)	Room temperature	User

NOTE
Pour out only the recommended reagent volume needed for the suggested number of samples listed in the Consumables table of each section. Some reagents are used later in the protocol.

NOTE
Thaw all reagents completely at room temperature and allow to

equilibrate. Once thawed, gently invert each tube several times to thoroughly mix the reagent. Pulse centrifuge each tube to 280 xg to eliminate bubbles and collect reagent at the bottom of the tube.

Preparation

- Do one of the following:
  - If you froze the MSA4 plate after fragmentation, thaw it to room temperature. Centrifuge to 280 xg for 1 minute.
  - If you are proceeding to precipitate MSA4 immediately after Fragment MSA4, leave the plate in the 37° C heat block until setup is complete.
- On the lab tracking form, record:
  - Date/Time
  - Operator
  - PM1 tube barcode(s)
  - 2-propanol lot number and date opened

NOTE
To record information about your assay such as operator information, start and stop times, and barcodes, use the lab tracking form provided at http://www.illumina.com/documentation. This form can be filled out and saved online, or printed and filled in by hand.

- Preheat heat block to 37° C.
- If frozen, thaw MSA4 plate to room temperature. Pulse centrifuge to 280 xg.
- Set centrifuge to 22° C (room temperature).
- Turn on the heat sealer.
- Remove the 96-well cap mat.

Steps to Precipitate MSA4 Plate

1. Add 100 µl PM1 to each MSA4 plate well containing sample.
2. Seal the plate with the cap mat.
3. Vortex the plate at 1600 rpm for 1 minute.
4. Incubate at 37° C for 5 minutes.
5. Centrifuge to 50 xg at 22° C for 1 minute.

NOTE
Set centrifuge to 4° C in preparation for the next centrifuge step.

6 Add 300 µl 100% 2-propanol to each well containing sample.

7 Carefully seal the MSA4 plate with a new, ***dry*** cap mat, taking care not to shake the plate in any way until the cap mat is fully seated.

8 Invert at least 10 times to mix contents thoroughly.

9 Incubate at 4° C for 30 minutes.

10 Place the sealed MSA4 plate in the centrifuge opposite another plate of equal weight.

Figure 25 Sealed MSA4 Plate and Plate of Equal Balance in Centrifuge

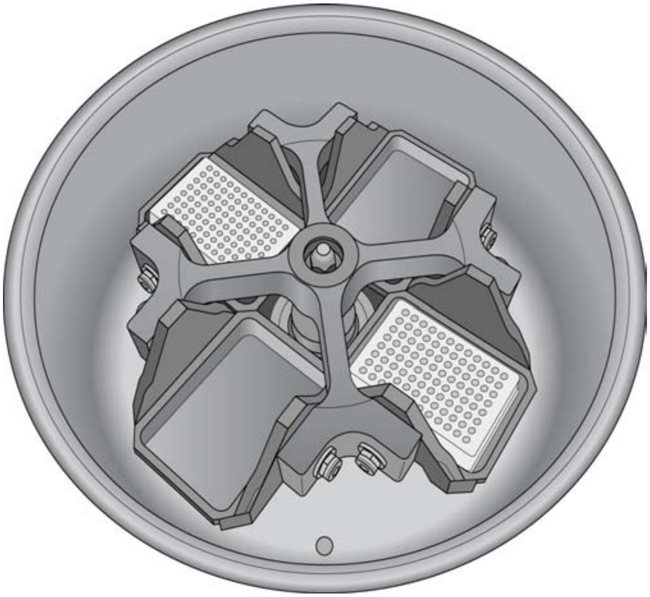

11 Centrifuge to 3,000 xg at 4° C for 20 minutes. Immediately remove the MSA4 plate from centrifuge.

CAUTION
Perform the next step immediately to avoid dislodging the blue pellet. If any delay occurs, repeat the 20-minute centrifugation before proceeding.

12 Remove the cap mat and discard it.

13 Decant supernatant by quickly inverting the MSA4 plate and smacking it down onto an absorbent pad.

14 Tap firmly several times for 1 minute or until all wells are devoid of liquid. Do not allow supernatant to pour into other wells.

CAUTION
Keep plate inverted. To ensure optimal performance, do not allow supernatant in wells to pour into other wells.

15 Leave the uncovered, inverted plate on the tube rack for 1 hour at room temperature to air dry the pellet.
At this point, blue pellets should be present at the bottoms of the wells.

Figure 26 Uncovered MSA4 Plate Inverted for Air Drying

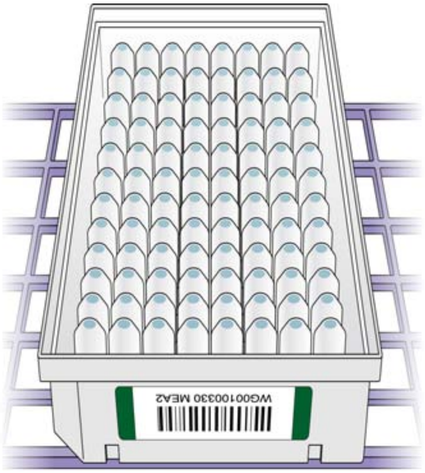

CAUTION
Do not over-dry the pellet. Pellets that are over-dried will be difficult to resuspend. Poorly resuspended samples will lead to poor data quality.

16 On the lab tracking form, enter the start and stop times.

17 Discard unused reagents in accordance with facility standards.

18 Do one of the following:

- Proceed to *Resuspend MSA4 Plate* on page 55.
- Seal the MSA4 plate with a cap mat and store it at -15° to -25° C if you do not plan to proceed to the next step immediately.

SAFE STOPPING POINT
This is a good stopping point in the process.

Resuspend MSA4 Plate

Add RA1 to the MSA4 plate to resuspend the precipitated DNA samples.

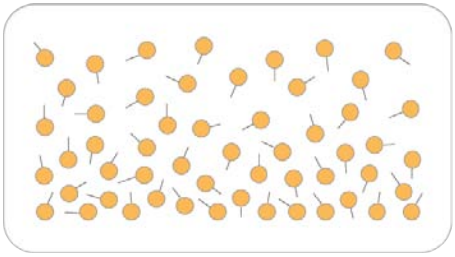

Figure 27 Resuspending DNA

Estimated Time

Hands-on time: ~30 minutes

Incubation time: 1 hour

Consumables

Item	Quantity	Storage	Supplied By
RA1	Bottle (46 µl per sample well)	-15° to -25° C	Illumina

NOTE
Pour out only the recommended volume of RA1 needed for the suggested number of samples listed in the consumables table. Additional RA1 is used later in the **XStain HD BeadChip** step.

WARNING
This protocol involves the use of an aliphatic amide that is a probable reproductive toxin. Personal injury can occur through inhalation, ingestion, skin contact, and eye contact. For more information, consult the material data safety sheet for this assay at

http://www.illumina.com/msds. Dispose of containers and any unused contents in accordance with the governmental safety standards for your region.

Preparation

- RA1 is shipped frozen. Gradually warm the reagent to room temperature, preferably in a 20–25° C water bath. Gently mix to dissolve any crystals that may be present.
- If you stored the MSA4 plate at -15° to -25° C, thaw it to room temperature. Remove the cap mat and discard it.
- Preheat the Illumina Hybridization Oven to 48° C.
- Turn on the heat sealer to preheat. Allow 20 minutes.
- On the lab tracking form, record:
  - Date/Time
  - Operator
  - RA1 bottle barcode(s)

NOTE
To record information about your assay such as operator information, start and stop times, and barcodes, use the lab tracking form provided at http://www.illumina.com/documentation. This form can be filled out and saved online, or printed and filled in by hand.

Steps to Resuspend MSA4 Plate

1. Add 46 µl RA1 to each well of the MSA4 plate containing a DNA pellet. Reserve any leftover reagent for Hyb Multi BeadChip and XStain BeadChip.
2. Apply foil seal to MSA4 plate by firmly holding the heat-sealer sealing block down for 5 seconds.
3. Place the sealed plate in the Illumina Hybridization Oven and incubate for 1 hour at 48° C.
4. Vortex the plate at 1800 rpm for 1 minute.

NOTE
If you store the pellets at -15° to -25° C for extended periods of time after the Precip MSA4 process, you may need to repeat steps 3 through 5 until the pellets are completely resuspended.

5 Pulse centrifuge to 280 xg.

6 Discard unused reagents in accordance with facility standards.

7 Do one of the following:
- Proceed to *Hybridize Multi BeadChip* on page 58.
- If you do not plan to proceed to the next step immediately, store the sealed MSA4 plate at -15° to -25° C. Store RA1 at -15° to -25° C
- If you plan to store the plate for more than 24 hours, store it at -80° C. Store RA1 at -15° to -25° C.

SAFE STOPPING POINT
This is a good stopping point in the process.

Hybridize Multi BeadChip

In this process, you dispense the fragmented and resuspended DNA samples onto BeadChips. Place the DNA-loaded BeadChips into the Hyb Chamber inserts, and then place the inserts into the Hyb Chambers. Incubate the Hyb Chambers in the Illumina Hybridization Oven for 16–24 hours at 48° C.

Figure 28 Hybridize Multi BeadChip

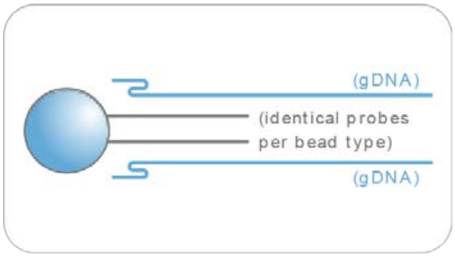

Estimated Time

Hands-on time:

- ~30 minutes for 48 samples
- ~40 minutes for 96 samples

Incubation time: 16–24 hours

Consumables

Item	Quantity (per 96 Samples)	Storage	Supplied By
PB2	2 tubes	Room temperature	Illumina
BeadChips	8		Illumina
EtOH	500 ml		User
Hyb Chambers	2		Illumina
Hyb Chamber gaskets	2		Illumina
Hyb Chamber inserts	8		Illumina

NOTE
Thaw all reagents completely at room temperature and allow to equilibrate. Once thawed, gently invert each tube several times to thoroughly mix the reagent. Pulse centrifuge each tube to 280 xg to eliminate bubbles and collect reagent at the bottom of the tube.

Preparation

- Preheat the heat block to 95° C.
- Preheat the Illumina Hybridization Oven to 48° C and set the rocker speed to 5.
- Prepare the Illumina Hybridization Oven as follows:
  a Preheat the oven to 48° C:
    - Press the "F" button once to change the display to **TSET**.
    - Press the "S" button to enter the set-temperature mode, and then use the Increment/Decrement dial to set the oven to 48° C.
    - Press the "S" button again to set 48° C as the temperature.
  b Set the rocker speed to 5:
    - Press the "F" button twice until **SPd** is indicated on the display.
    - Press the "S" button to enter the rocker speed mode.
    - Use the Increment/Decrement dial to set the rocker speed to "5".

— Press the "S" button again.

- Calibrate the Illumina Hybridization Oven with the Full-Scale Plus digital thermometer supplied with your system.
- On the lab tracking form, record:
  - Date/Time
  - Operator
  - PB2 tube barcode

NOTE
To record information about your assay such as operator information, start and stop times, and barcodes, use the lab tracking form provided at http://www.illumina.com/documentation. This form can be filled out and saved online, or printed and filled in by hand.

Assemble Hybridization Chambers

1 Place the following items on the bench top:
  - BeadChip Hyb Chambers(2)
  - Hyb Chamber Gaskets (2)
  - BeadChip Hyb Chamber inserts (8)

Figure 29 BeadChip Hyb Chamber Components

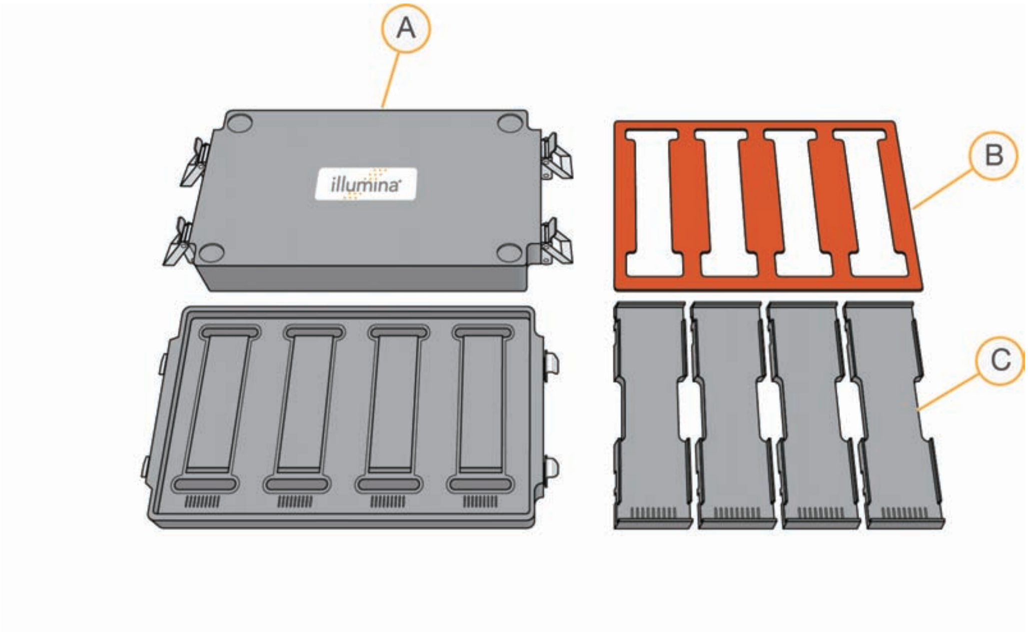

A Hyb Chamber
B Hyb Chamber Gasket
C Hyb Chamber Inserts

NOTE
To ensure optimal results from Hyb Chambers keep the Hyb Chamber lids and bases together. Adopt a labeling convention that keeps each Hyb Chamber base paired with its original lid. Check Hyb Chamber lid-base pairs regularly to ensure that the fit remains secure. Check hinges regularly for any signs of abnormal wear or loose fittings. It is important that the hinges provide adequate clamping strength to ensure an airtight seal between the lid and the base. Record the Hyb Chamber that was used for each BeadChip, so that Hyb Chambers can be investigated and evaluated in the event of sample evaporation or other lab processing anomalies.

2 Place the BeadChip Hyb Chamber gaskets into the BeadChip Hyb Chambers as shown.

a Match the wider edge of the Hyb Chamber gasket to the barcode-ridge side of the Hyb Chamber.

Figure 30 Hyb Chamber and Gasket

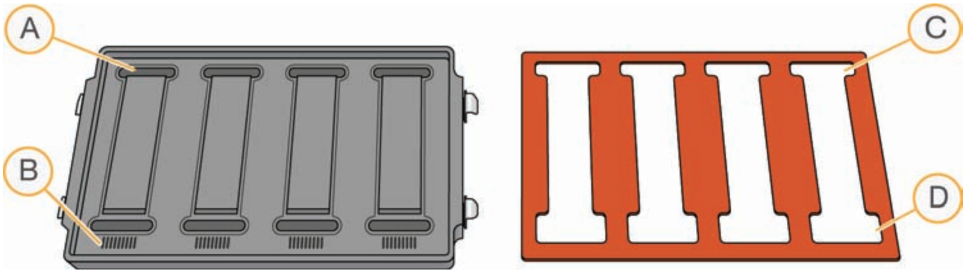

A Reservoirs
B Barcode Ridges
C Narrower Edges
D Wider Edges

b Lay the gasket into the Hyb Chamber, and then press it down all around.

Figure 31 Placing Gasket into Hyb Chamber

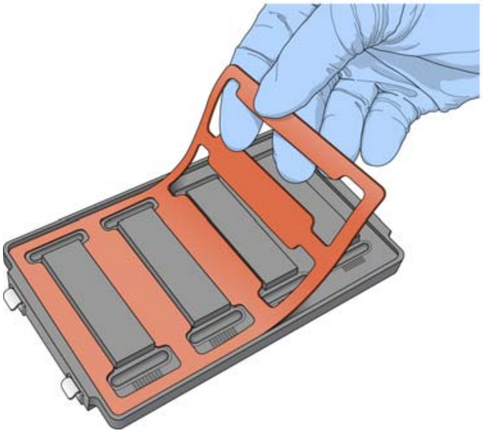

c Make sure the Hyb Chamber gaskets are properly seated.

Figure 32 Hyb Chamber with Gasket in Place

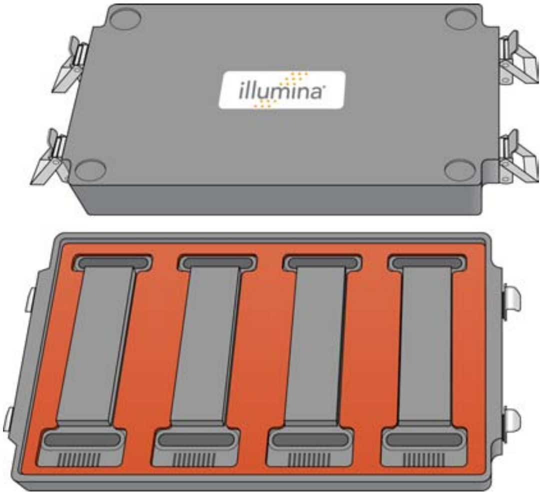

3 Dispense 400 µl PB2 into the humidifying buffer reservoirs in the Hyb Chambers.

Figure 33 Dispensing PB2 into Hyb Chamber Reservoir

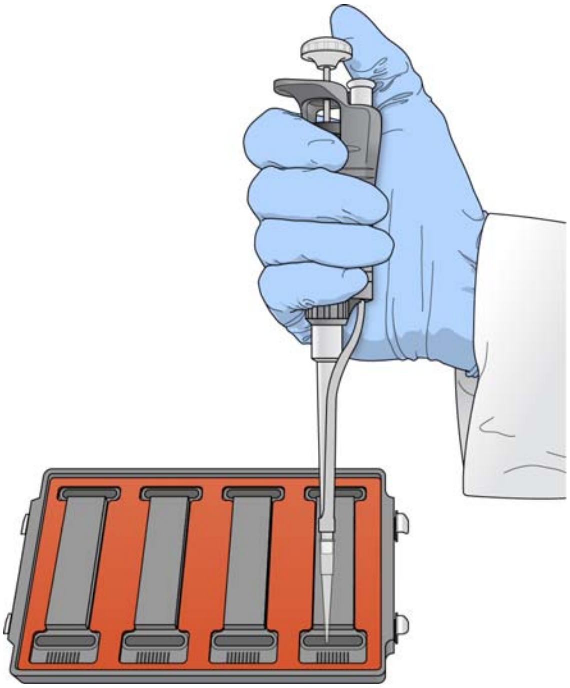

WARNING

Do not replace PB2 in the Hyb Chamber with RA1. This will decrease the stringency and may negatively affect sample call rates and logRdev. PB2 is formulated to produce the appropriate amount of humidity within the Hyb Chamber environment to prevent sample from evaporating during hybridization.

4 Close and lock the BeadChip Hyb Chamber lid.
   a Seat the lid securely on the bottom plate.
   b Snap two clamps shut, kitty-corner across from each other.
   c Snap the other two clamps.

Figure 34 Sealing the Hyb Chamber

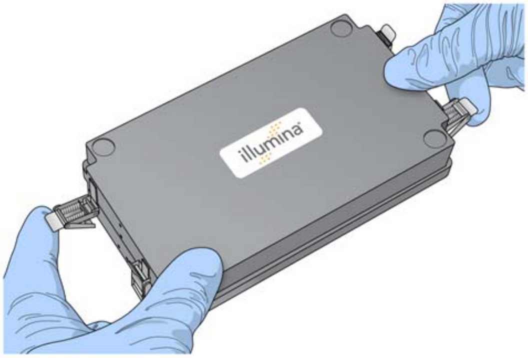

5 Leave the closed Hyb Chambers on the bench at room temperature until the BeadChips are loaded with DNA sample. Load BeadChips into the Hyb Chamber within one hour.

Hybridize and Load BeadChip

1 Place the resuspended MSA4 plate on the heat block to denature the samples at 95° C for 20 minutes.

CAUTION
Do not unpackage BeadChips until you are ready to begin hybridization.

2 After the 20-minute incubation, remove the MSA4 plate from the heat block, and place it on the benchtop at room temperature for 30 minutes.

3 After the 30-minute cool down, pulse centrifuge the MSA4 plate to 280 xg for one minute.

4 Remove the BeadChips from 2–8° C storage but do not unpackage.

5 Just before loading DNA samples, remove all BeadChips from their packages.

6 Place each BeadChip in a Hyb Chamber insert, orienting the barcode end so that it matches the barcode symbol on the Hyb Chamber insert.

Figure 35 Placing BeadChips into Hyb Chamber Inserts

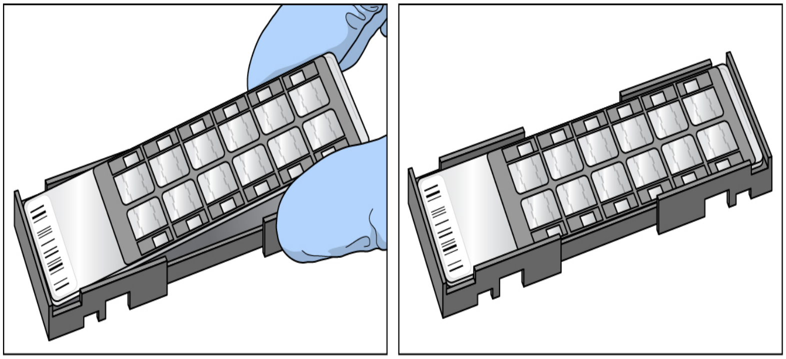

CAUTION

Hold the BeadChip by the ends with your thumb and forefinger (thumb at the barcode end). Do not hold the BeadChip by the sides near the sample inlets. Avoid contacting the beadstripe area and sample inlets.

NOTE

For an alternative DNA loading protocol, see *Load BeadChip (Alternate Method)* on page 74.

7 Using a multi-channel precision pipette, dispense 15 µl of each DNA sample onto the appropriate BeadChip section, according to the chart on the lab tracking form and the following illustrations:

a Load samples A1–F1 from the MSA4 plate into the left side of the BeadChip.

Figure 36 Loading Samples A1–F1

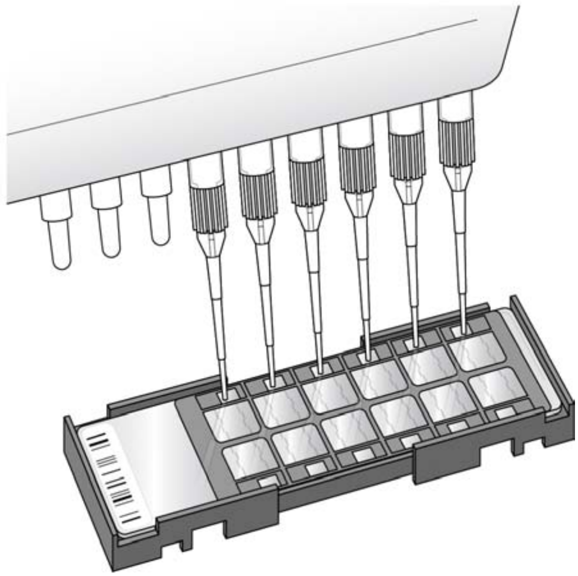

b Load samples in G1 and H1 from the MSA4 plate into the top two inlets of the right side of the BeadChip.

c Load samples A2–D2 into the remaining four inlets on the right side of the BeadChip.

d Continue in this manner, following the color-coded sections shown below, until all samples are loaded.

Figure 37 Distributing Sample in MSA4 Plate

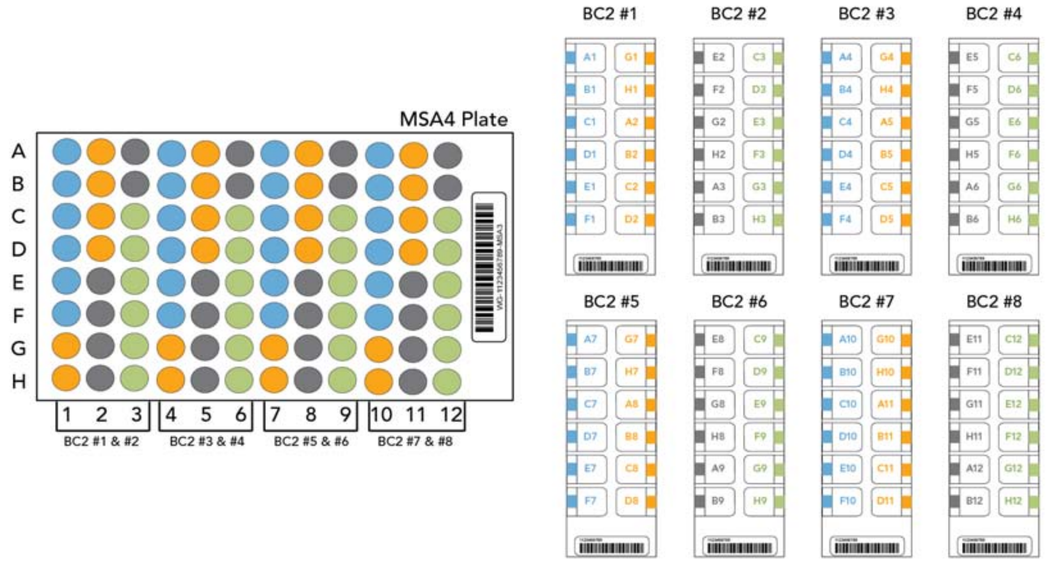

NOTE
Load samples by directly placing pipette tips to the array surface and hold the pipette straight up above the array surface. Do not hold the pipette at an angle while you are loading the sample. Proceed immediately to the next step as soon as all arrays have received sample.

8 On the lab tracking form, record the BeadChip barcode for each group of samples.

9 After loading all DNA onto the BeadChip, wait for the sample to disperse over the entire surface.

10 Inspect the loading port to see if a large bolus of liquid remains. Excess sample volume in the BeadChip loading port helps prevent low-intensity areas resulting from evaporation.

Figure 38 Bolus of Liquid at Loading Port

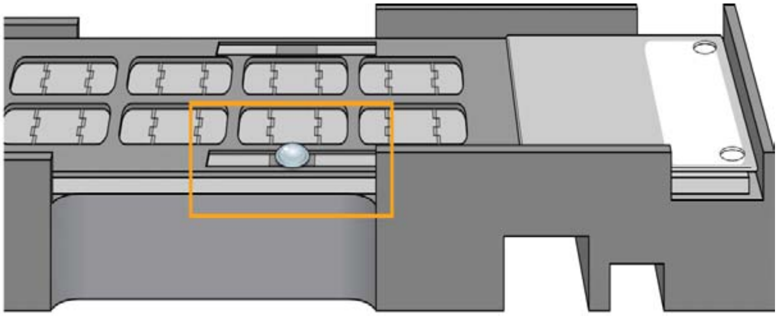

11 If no excess liquid is visible, it is acceptable to add additional sample from the leftover volume in the amplification plate until there is a large bolus around the loading port.

NOTE
Do not top off with RA1 (sample hyb buffer) as this will dilute the sample.

12 Record the top-off activity on the lab tracking form.

13 Heat-seal any residual sample in the MSA4 plate with foil, and store at -15° to -25° C. Store at -80° C if you do not plan to use the sample again within 24 hours.

Set up Multi BeadChip for Hybridization

CAUTION
For optimal performance, take care to keep the Hyb Chamber inserts containing BeadChips steady and level when lifting or moving. Avoid shaking and keep parallel to the lab bench at all times. Do not hold by the sides near the sample inlets.

1 Load the Hyb Chamber inserts containing BeadChips into the Illumina Hyb Chamber (below). Position the barcode end over the ridges indicated on the Hyb Chamber.

WARNING
Hyb Chambers should be at room temperature when you load the

BeadChips. They should not be preconditioned in the Illumina Hybridization Oven prior to loading the BeadChips. Heating the PB2 and then opening the Hyb Chamber to add BeadChips causes some of the PB2 to evaporate, leading to a change in the osmolality of PB2 and an imbalance in the vapor pressure between PB2 and RA1 (sample hyb buffer).

Figure 39 Placing Hyb Chamber Inserts into the Hyb Chamber

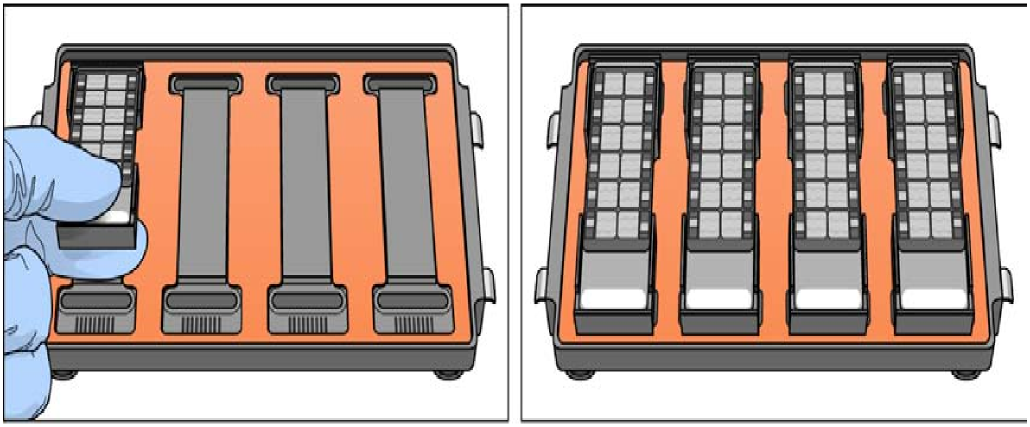

2 Place the back side of lid onto the Hyb Chamber and then slowly bring down the front end to avoid dislodging the Hyb Chamber inserts.

Figure 40 Seating Lid onto Hyb Chamber

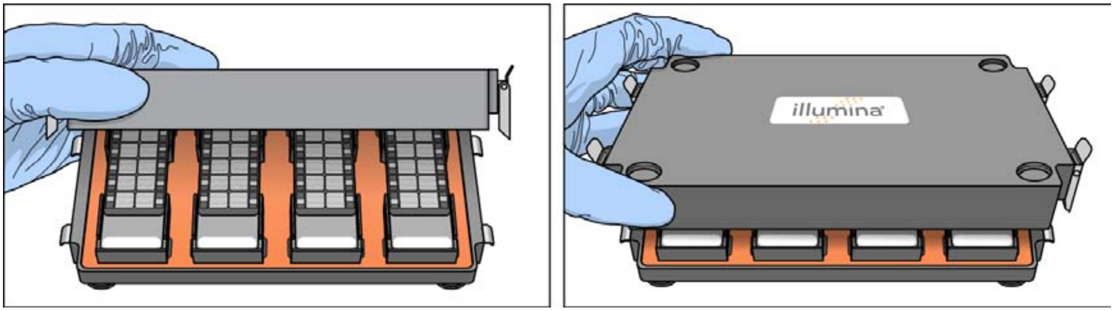

3 Close the clamps on both sides of the Hyb Chamber. It is best to close them in a kitty-corner fashion, closing first the top left clamp, then the bottom right, then the top right followed by the bottom left.

NOTE
For optimal performance, take care to keep the Hyb Chamber steady and level when lifting or moving. Avoid shaking the Hyb Chamber, keep the Hyb Chamber parallel to the lab bench while you transfer it to the Illumina Hybridization Oven.

4 Place the Hyb Chamber into the 48° C Illumina Hybridization Oven so that the clamps of the Hyb Chamber face the left and right side of the oven. The Illumina logo on top of the Hyb Chamber should be facing you.

CAUTION
After loading the BeadChips into the Hyb Chambers, place the Hyb Chambers into the Illumina Hybridization Oven immediately. Do not modify the hybridization environment by adding additional fixtures or humidifying elements. Leave the Hyb Chambers in the oven at the correct orientation and temperature until hybridization is complete. Changes to the hybridization environment can have unexpected effects on data quality.

Figure 41 Hyb Chamber Correctly Placed in Hyb Oven

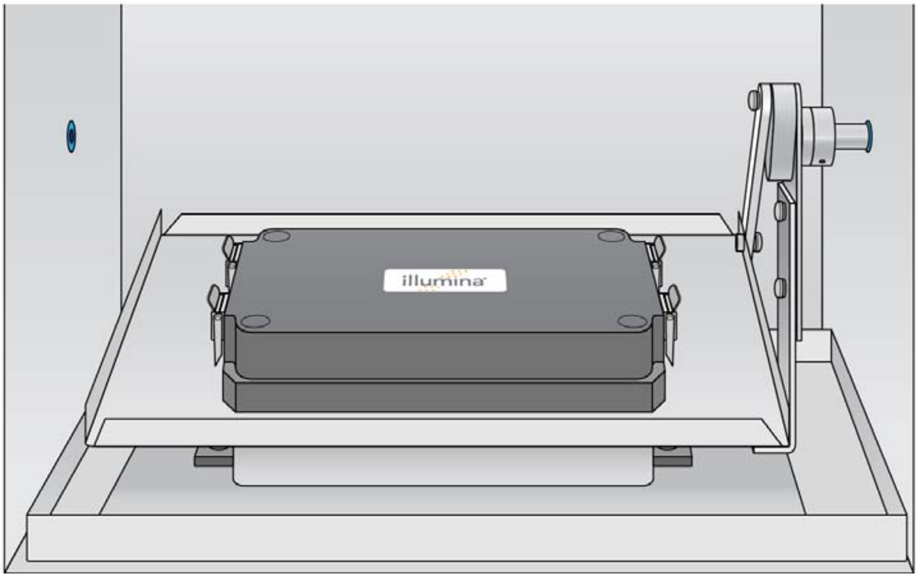

5 If you are loading multiple Hyb Chambers, you may stack them on top of each other. You can stack up to four Hyb Chambers.

NOTE
If you are stacking multiple Hyb Chambers in the Illumina Hybridization Oven, make sure the feet of the top Hyb Chamber fit into the matching indents on top of the bottom Hyb Chamber. This will hold the Hyb Chambers in place while they are rocking.

Figure 42 Two Hyb Chambers Correctly Placed in Hyb Oven

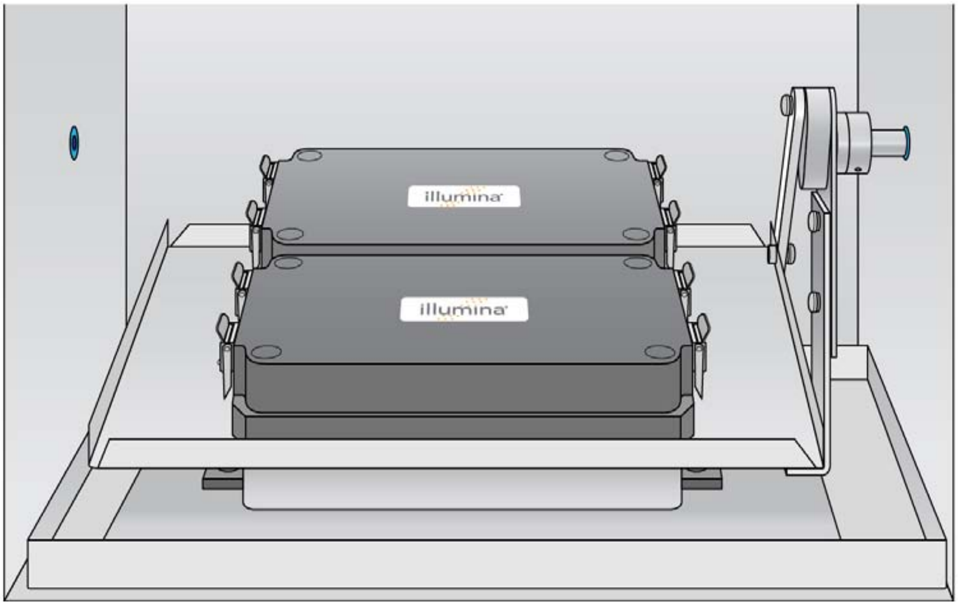

Figure 43 Incorrectly Placed Hyb Chamber

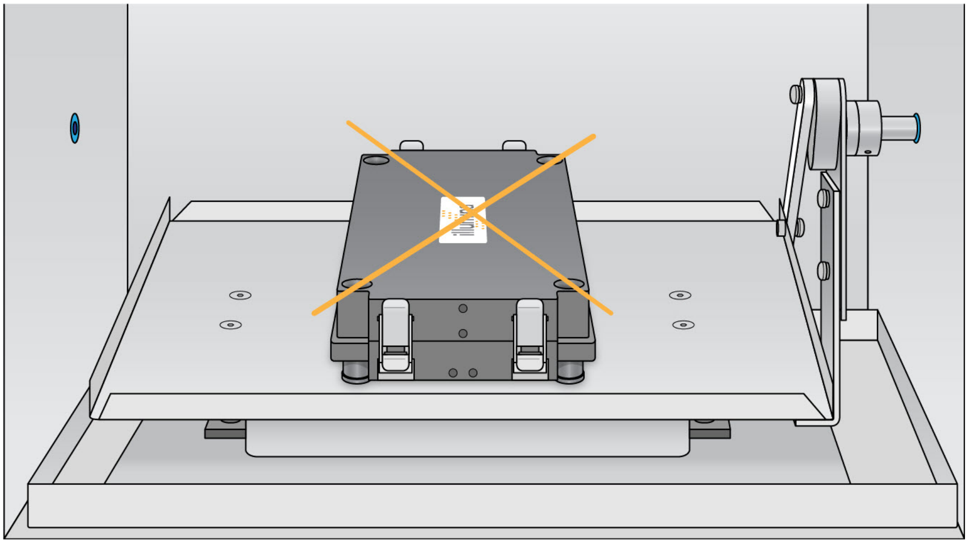

6 Start the rocker, setting the speed to 5 (optional).

7 Incubate at 48° C for at least 16 hours but no more than 24 hours.

8 On the lab tracking form, enter the start and stop times.

9 Place RA1 into the freezer at -15° to -25° C for use the next day.

Resuspend XC4 Reagent for XStain BeadChip

Keep the XC4 in the bottle in which it was shipped until you are ready to use it. In preparation for the XStain protocol, follow these steps to resuspend the XC4 reagent:

1 Add 330 ml 100% EtOH to the XC4 bottle. The final volume will be 350 ml.
Each XC4 bottle (350 ml) has enough solution to process up to 24 BeadChips.

2 Shake vigorously for 15 seconds.

3 Leave the bottle upright on the lab bench overnight.

4 Shake again to ensure that the pellet is completely resuspended. If any coating is visible, vortex at 1625 rpm until it is in complete suspension. Once resuspended with 330 ml 100% EtOH, bring XC4 to room temperature before use.

NOTE
If the XC4 was not left to resuspend overnight, you can still proceed with the assay. Add the EtOH and put the XC4 on its side on a rocker to resuspend. Leave it there until the BeadChips are ready for coating.

Load BeadChip (Alternate Method)

This section describes an alternative method of manually loading the DNA by rows instead of columns from the MSA4 plate. For the original method of loading DNA samples, see *Hybridize and Load BeadChip* on page 65.

1 Place the resuspended MSA4 plate on the heat block to denature the samples at 95° C for 20 minutes.

2 After the 20-minutes incubation, remove the MSA4 plate from the heat block and place it on the benchtop at room temperature for 30 minutes.

3 After the 30-minute cool down, pulse centrifuge the MSA4 plate to 280 xg for one minute.

4 Remove all the BeadChips from their packages.

5 Place each BeadChip in a Hyb Chamber insert, orienting the barcode end so that it matches the barcode symbol on the Hyb Chamber insert. Repeat for each BeadChip.

Figure 44 Placing BeadChips into Hyb Chamber Inserts

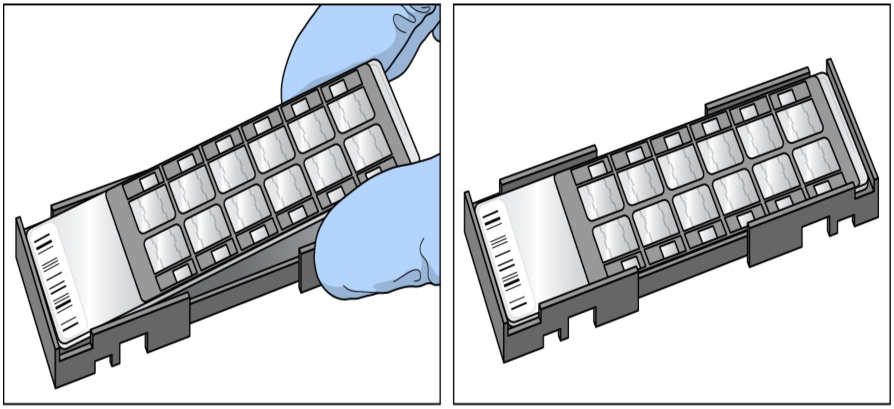

6 Remove the MSA4 plate from the centrifuge. Remove the foil seal.

7 Using a multi-channel precision pipette, dispense 15 µl of each DNA sample onto the appropriate BeadChip stripe. Follow the alternate loading pattern on the Lab Tracking Forms:

a Load samples A1–A6 from the MSA4 plate to the left side of the BeadChip.

Figure 45 Dispensing Sample onto Arrays

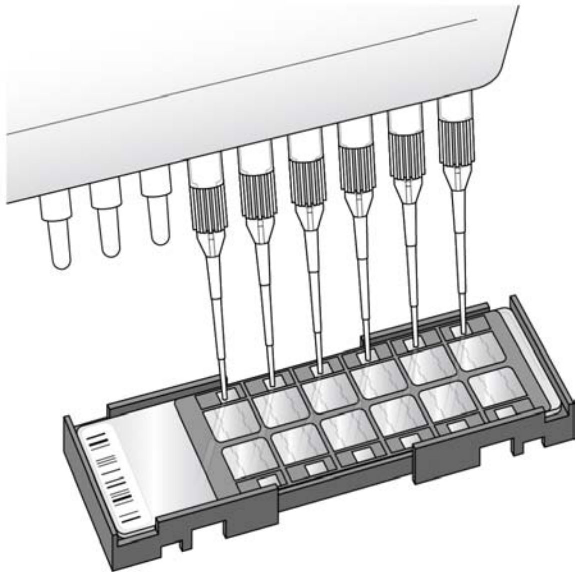

b Load samples B1–B6 from the MSA4 plate to the right side of the BeadChip.

c Continue in this manner for Rows C through H.

d When you finish the left half of the plate, move to the right half of the plate. Load samples A7–A12 to the left side of the BeadChip and samples B7–B12 to the right side of the BeadChip. Continue in this manner for rows C through H.

Figure 46 Alternate Distributing Sample in MSA4 Plate

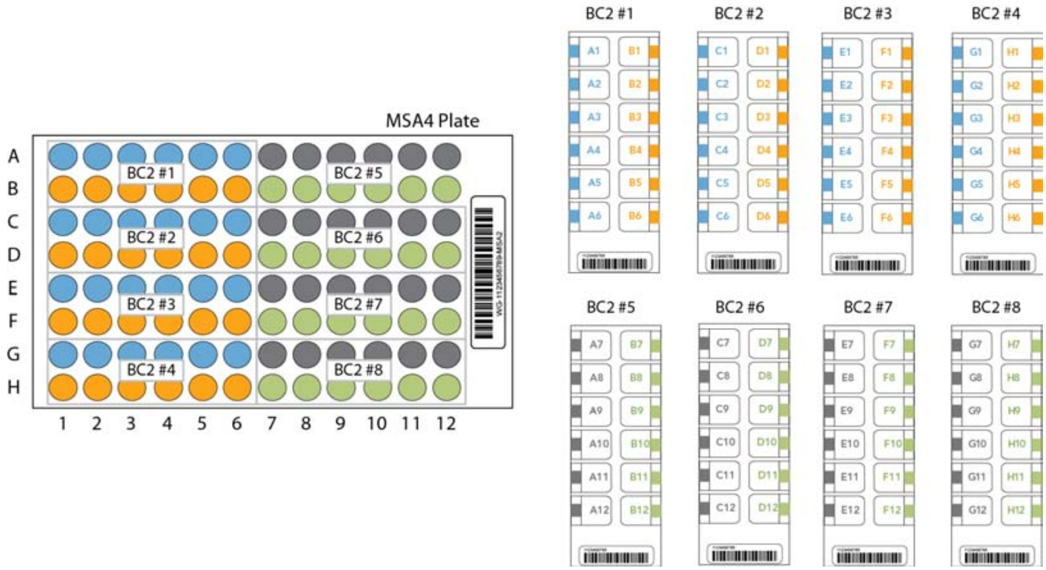

8 On the Lab Tracking Forms, record the BeadChip barcode for each group of samples.

9 Visually inspect all sections of the BeadChips to ensure the DNA sample covers all of the sections of each bead stripe. Record any sections that are not completely covered.

10 Heat-seal residual sample in the MSA4 plate with foil and store at -15 to -25° C. Store at -80° C if you do not plan to use it again within 24 hours.

Wash BeadChip

In this process, the BeadChips are prepared for the XStain BeadChip process. Coverseals are removed from BeadChips and the BeadChips are washed in PB1 reagent. BeadChips are then assembled into Flow-Through Chambers under the PB1 buffer.

Figure 47 Washing BeadChip

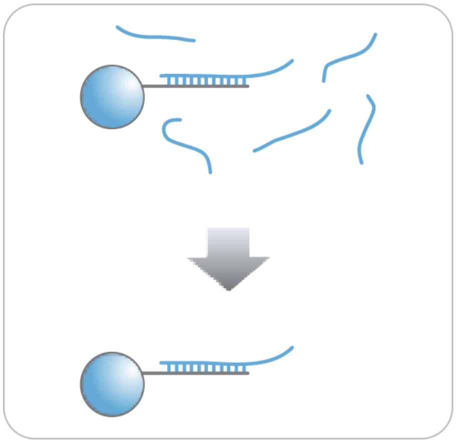

Estimated Time

- 20 minutes for 4 BeadChips
- 30 minutes for 8 BeadChips

Consumables

Item	Quantity (per 4 BeadChips)	Storage	Supplied By
PB1	550 ml (up to 8 BeadChips)	Room temperature	Illumina
Multi-Sample BeadChip Alignment Fixture	1 (per 8 BeadChips)		Illumina
Te-Flow -Through Chambers (with Black Frames, Spacers, Glass Back Plates, and Clamps)	4		Illumina
Wash Dish	2 (up to 8 BeadChips)		Illumina
Wash Rack	1 (up to 8 BeadChips)		Illumina

NOTE
Pour out only the recommended reagent volume needed for the suggested number of samples listed in the Consumables table of each section. Some reagents are used later in the protocol.

WARNING
This protocol involves the use of an aliphatic amide that is a probable reproductive toxin. Personal injury can occur through inhalation, ingestion, skin contact, and eye contact. For more information, consult the material data safety sheet for this assay at http://www.illumina.com/msds. Dispose of containers and any unused contents in accordance with the governmental safety standards for your region.

NOTE
Thaw all reagents completely at room temperature and allow to equilibrate. Once thawed, gently invert each tube several times to thoroughly mix the reagent. Pulse centrifuge each tube to 280 xg to eliminate bubbles and collect reagent at the bottom of the tube.

Preparation

- Remove each Hyb Chamber from the Illumina Hybridization Oven. Let cool on the benchtop for 25 minutes prior to opening.
- Have ready on the lab bench:
  - Two wash dishes:
    - Containing 200 ml PB1, and labeled as such
  - Multi-Sample BeadChip Alignment Fixture
    - Using a graduated cylinder, fill with 150 ml PB1
  - Te-Flow -Through Chamber components:
    - Black frames
    - Spacers (separated for ease of handling)
    - Clean glass back plates (Clean as directed in the SOP)
    - Clamps
- On the Lab Tracking Forms, record:
  - Date/Time
  - Operator
  - Robot
  - PB1 bottle barcode

NOTE
You can download and print copies of the lab tracking worksheet from http://www.illumina.com/documentation.

Steps to Wash BeadChip

1. Attach the wire handle to the rack and submerge the wash rack in the wash dish containing 200 ml PB1.
2. Remove the Hyb Chamber(s) from the Illumina Hybridization Oven.
3. Wait 30 minutes for the Hyb Chambers to cool down, and then remove BeadChips from the Hyb Chamber inserts one at a time.

Figure 48

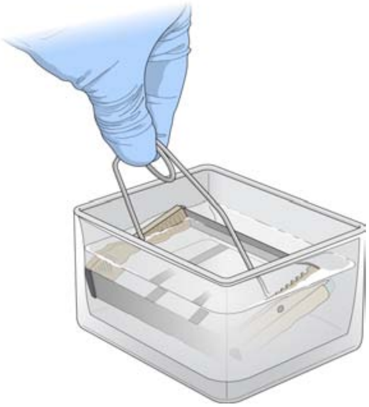

4 Remove the coverseal from the BeadChip as follows:

   a Using powder-free gloved hands, hold the BeadChip in one hand with your thumb and forefinger on the long edges of the BeadChip. The BeadChip may also be held with the thumb and forefinger on the short edges of the BeadChip. In either case avoid contact with the sample inlets. The barcode should be facing up and be closest to you, and the top side of the BeadChip should be angled slightly away from you.

   b Remove the entire seal in a single, slow, consistent motion by pulling it off in a diagonal direction away from yourself. Start with a corner on the barcode end and pull with a continuous upward motion away from you and towards the opposite corner on the top side of the BeadChip. Do not stop and start the pulling action. Do not touch the exposed active areas.

NOTE
To ensure no solution splatters on you, be sure to pull the coverseal away from yourself. Illumina recommends removing the coverseal over an absorbent cloth or paper towels, preferably in a hood.

   c Discard the coverseal.

Figure 49 Removing the Coverseal

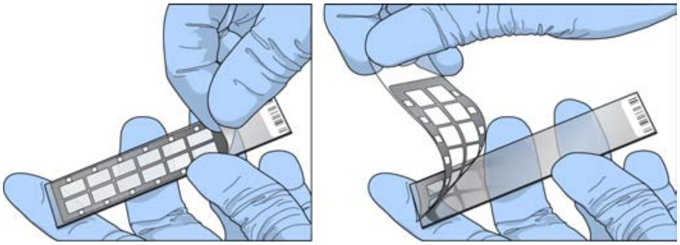

CAUTION
Do not touch the arrays!

5 Immediately and carefully slide the BeadChips into the wash rack, making sure that they are completely submerged in the PB1.

Figure 50 Submerging BeadChips in Wash Dish Containing PB1

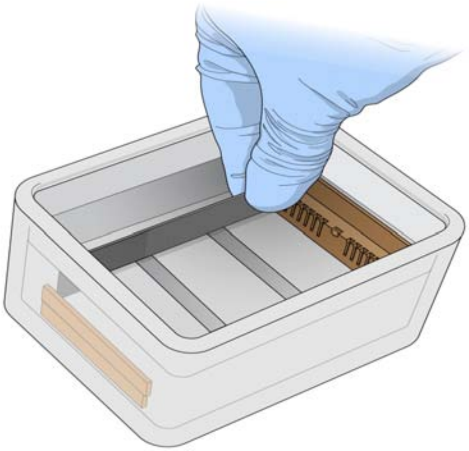

6 Repeat steps 4 through 5 for each BeadChip to be processed. The wash rack holds up to 8 BeadChips.

7 Once all BeadChips are in the wash rack, move the wash rack up and down for 1 minute, breaking the surface of the PB1 with gentle, slow agitation.

8 Move the wash rack to the other wash dish containing clean PB1. Make sure the BeadChips are completely submerged.

9 Move the wash rack up and down for 1 minute, breaking the surface of the PB1 with gentle, slow agitation.

10 When you remove the BeadChips from the wash rack, inspect them for remaining residue.

NOTE
Residue that can adversely affect results is sometimes left on BeadChips after seals are removed. If there is residue left on the BeadChips after the second PB1 wash, use a 200 ul pipette tip for each BeadChip and slowly and carefully scrape off the residues outward (away) from the bead-sections under PB1. Use a new pipette tip for each BeadChip. Then, continue with the protocol.

11 If you are processing more than 8 BeadChips.

a Complete the steps in the next section, Assemble Flow-Through Chambers, for the first eight BeadChips.

b Place the assembled Flow-Through Chambers of the first eight BeadChips on the lab bench in a horizontal position.

NOTE
Keep the Flow-Through Chambers in a horizontal position on the lab bench until all assembled Flow-Through Chambers are ready to be loaded into the Chamber Rack. Do not place the Flow-Through Chambers in the Chamber Rack until all BeadChips are prepared in Flow-Through Chambers.

c Repeat steps 2 through 10 from this section for any additional BeadChips. Use new PB1 for each set of eight BeadChips.

12 ***Immediately*** wash the Hyb Chamber reservoirs with dH_2O and scrub them with a small cleaning brush, ensuring that no PB2 remains in the Hyb Chamber reservoir.

CAUTION
It is important to wash the Hybridization Chamber reservoirs immediately and thoroughly to ensure that no traces of PB2 remain in the wells.

Assemble Flow-Through Chambers

NOTE
The 150 ml used in the BeadChip alignment fixture can be used for up to eight BeadChips. You must used 150 ml of fresh PB1 for every additional set of eight BeadChips.

1 For each BeadChip to be processed, place a black frame into the Multi-Sample BeadChip Alignment Fixture pre-filled with PB1.

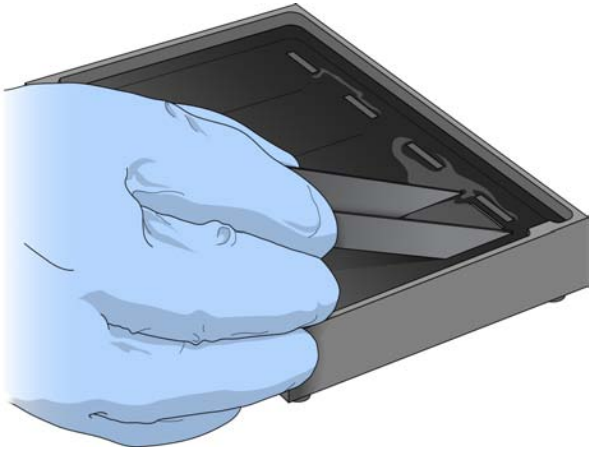

Figure 51 Placing Black Frames into Multi-Sample BeadChip Alignment Fixture

2 Place each BeadChip to be processed into a black frame, aligning its barcode with the ridges stamped onto the Alignment Fixture.

NOTE
Inspect the surface of each BeadChip for residue left by the seal. Use a pipette tip to remove any residue under buffer and be careful not to scratch the bead area.

Figure 52 Placing BeadChip into Black Frame on Alignment Fixture

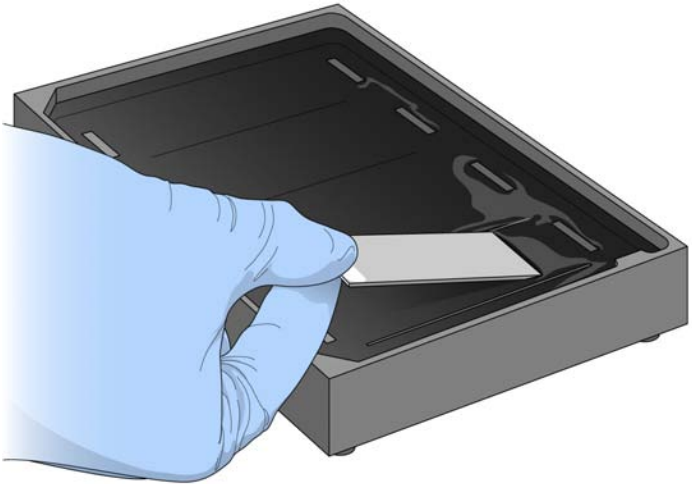

3 Place a clear spacer onto the top of each BeadChip. Use the Alignment Fixture grooves to guide the spacers into proper position.

NOTE
Be sure to use the clear plastic spacers, not the white ones.

Figure 53 Placing Clear Plastic Spacer onto BeadChip

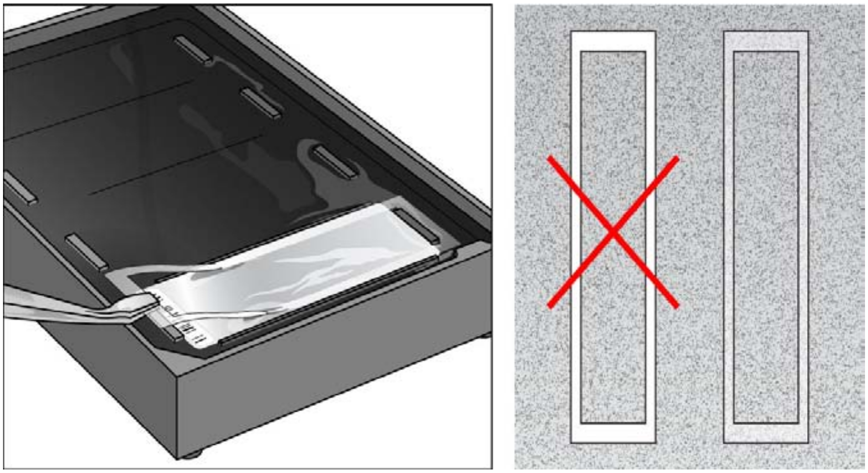

4 Place the Alignment Bar onto the Alignment Fixture.

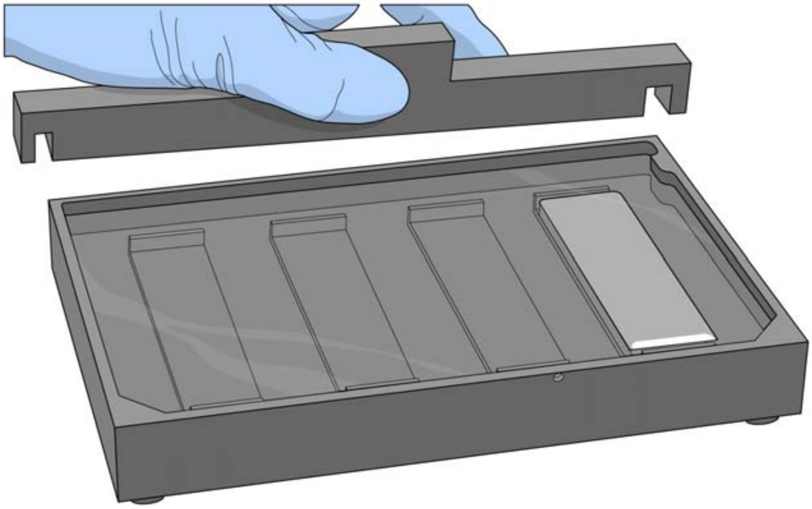

Figure 54 Placing Alignment Bar onto Alignment Fixture

5 Place a clean glass back plate on top of the clear spacer covering each BeadChip. The plate reservoir should be at the barcode end of the BeadChip, facing inward to create a reservoir against the BeadChip surface.

Figure 55 Placing Glass Back Plate onto BeadChip

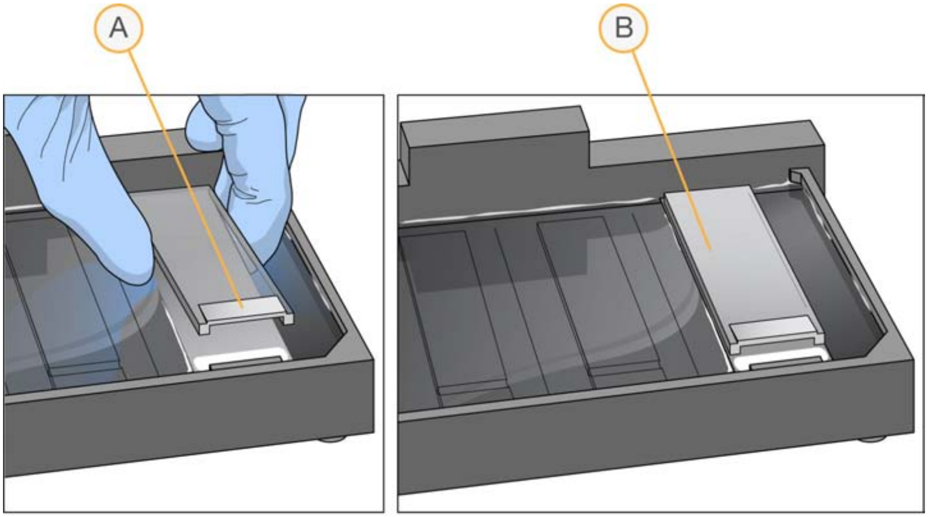

A Reservoir at Barcode End of Glass Back Plate
B Glass Plate Back in Position

6 Attach the metal clamps to the Flow-Through Chambers as follows:
   a Gently push the glass back plate up against the Alignment Bar with one finger.
   b Place the first metal clamp around the Flow-Through Chamber so that one stripe shows between it and the Alignment Bar.
   c Place the second metal clamp around the Flow-Through Chamber at the barcode end, just below the reagent reservoir, so that no stripes show between the clamp and the barcode.

Figure 56 Securing Flow-Through Chamber Assembly with Metal Clamps

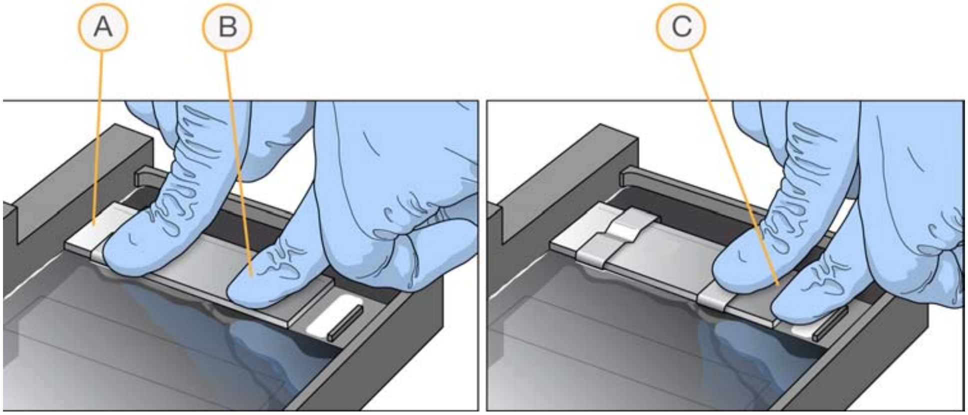

A One Stripe Shows Between First Clamp and Alignment Bar
B Glass Back Plate Pressed Against Alignment Bar
C No Stripes Show Between Second Clamp and Barcode

7 Using scissors, trim the ends of the clear plastic spacers from the Flow-Through Chamber assembly. Slip scissors up over the barcode to trim the other end:
   a Trim spacer ends at the non-barcode end of the assembly.
   b On the barcode end of the assembly, slip scissors up over the barcode to trim spacer ends.

Figure 57 Trimming Spacer Ends from Flow-Through Chamber Assembly

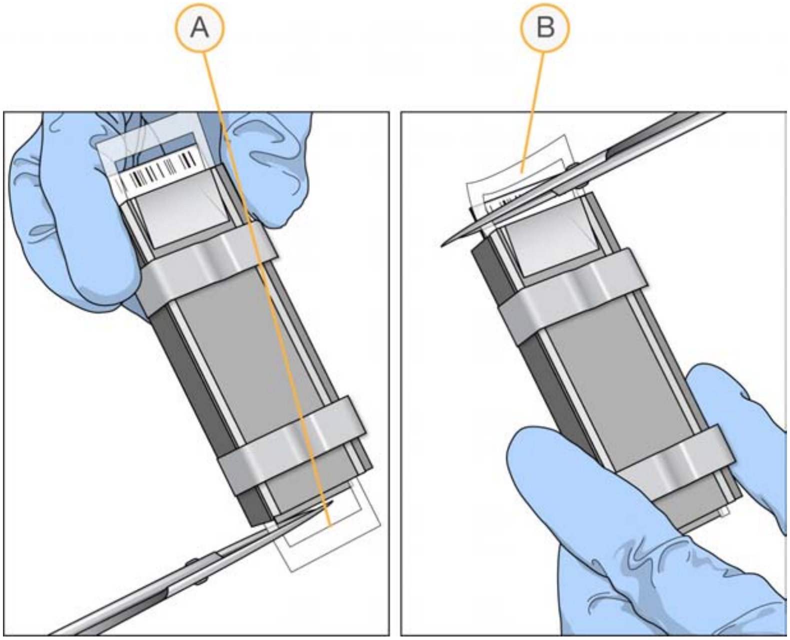

A Trim Spacer at Non-Barcode End of Flow-Through Chamber
B Trim Spacer at Barcode End of Flow-Through Chamber

8 Discard unused reagents in accordance with facility standards.

CAUTION
Place all assembled Flow-Through Chambers on the lab bench in a horizontal position while you perform the preparation steps for XStain BeadChip. Do not place the Flow-Through Chambers in the Chamber Rack until all necessary steps are completed.

Single-Base Extension and Stain BeadChip

In this process, you use RA1 reagent to wash away unhybridized and non-specifically hybridized DNA sample. Dispense TEM reagent into the Flow-Through Chambers to extend the primers hybridized to DNA on the BeadChip. This reaction incorporates labeled nucleotides into the extended primers. 95% formamide/1 mM EDTA is added to remove the hybridized DNA. After neutralization using the XC3 reagent, the labeled extended primers undergo a multi-layer staining process on the Chamber Rack. Next, you disassemble the Flow-Through Chambers and wash the BeadChips in the PB1 reagent, coat them with XC4, and then dry them.

Figure 58 Extending and Staining BeadChip

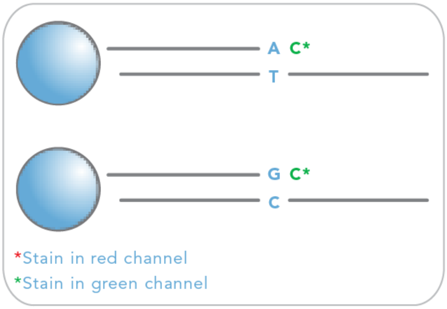

Estimated Time

Hands-on time: ~3 hours for 8 BeadChips

Dry time: 1 hour

Consumables

Item	Quantity (Per 8 BeadChips)	Storage	Supplied By
RA1	10 ml (see *Setup* for special instructions)	-15° to -25° C	Illumina
XC1	2 tubes	-15° to -25° C	Illumina
XC2	2 tubes	-15° to -25° C	Illumina
TEM	2 tubes	-15° to -25° C	Illumina
XC3	75 ml	Room temperature	Illumina
STM (Make sure that all STM tubes indicate the same stain temperature on the label)	2 tubes	-15° to -25° C	Illumina
ATM	2 tubes	-15° to -25° C	Illumina
PB1	310 ml	Room temperature	Illumina
XC4	310 ml	-15° to -25° C	Illumina
Alconox Powder Detergent	as needed		Illumina
EtOH	as needed	Room temperature	Illumina
95% formamide/1 mM EDTA	15 ml	-15° to -25° C	User

CAUTION
Pour out only the recommended reagent volume needed for the suggested number of BeadChips listed in the consumables table of each section. Some of the reagents are used later in the protocol.

NOTE

It is important to use fresh RA1 for each protocol step in the assay where it is required. RA1 that has been stored properly and has not been dispensed for use in either the XStain or Resuspension step is considered fresh RA1. After RA1 has been poured out into a reservoir and exposed to room temperature air for extended periods of time, it is no longer fresh.

WARNING

This protocol involves the use of an aliphatic amide that is a probable reproductive toxin. Personal injury can occur through inhalation, ingestion, skin contact, and eye contact. For more information, consult the material data safety sheet for this assay at http://www.illumina.com/msds. Dispose of containers and any unused contents in accordance with the governmental safety standards for your region.

Preparation

- RA1 is shipped and stored at -15° to -25° C. Gradually warm the reagent to room temperature, preferably in a 20–25° C water bath. Gently mix to dissolve any crystals that may be present.
- Place all reagent tubes in a rack in the order in which they will be used. If frozen, allow them to thaw to room temperature, and then gently invert and centrifuge to 3000 xg for 3 minutes.

Figure 59 XStain BeadChip Reagent Tubes and Bottles

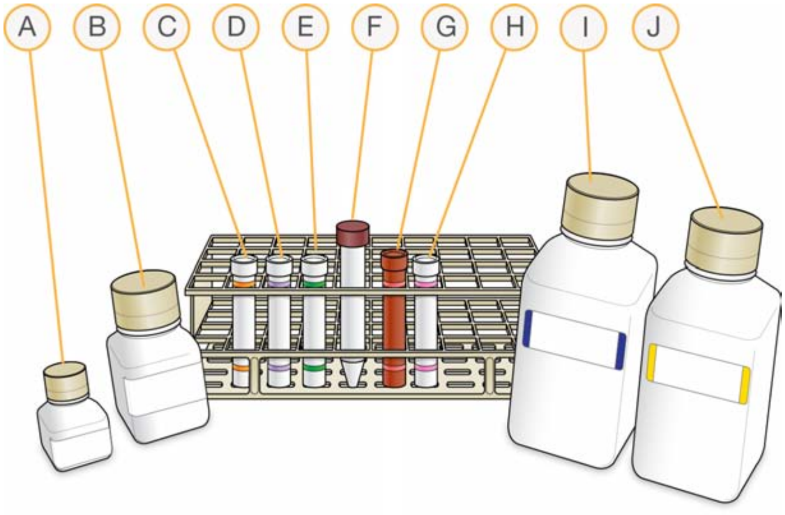

A RA1
B XC3
C XC1
D XC2
E TEM
F 95% Formamide / 1mM EDTA
G STM
H ATM
I PB1
J XC4

- Dispense all bottled reagents into disposable reservoirs, as they are needed.
- On the lab tracking form, record:
  - Date/Time
  - Operator
  - RA1 barcode
  - XC3 barcode
  - XC1 barcode(s)
  - XC2 barcode(s)
  - TEM barcode(s)

- STM barcode(s)
- ATM barcode(s)
- PB1 barcode
- XC4 barcode(s)

NOTE
To record information about your assay such as operator information, start and stop times, and barcodes, use the lab tracking form provided at http://www.illumina.com/documentation. This form can be filled out and saved online, or printed and filled in by hand.

Set Up Chamber Rack

1. Ensure the water circulator reservoir is filled with water to the appropriate level. See the *VWR Operator's Manual*, VWR part # 110-229.
2. Turn on the water circulator and set it to a temperature that brings the Chamber Rack to 44° C at equilibrium.
   This temperature may vary depending on facility ambient conditions.

Figure 60 Water Circulator Connected to Chamber Rack

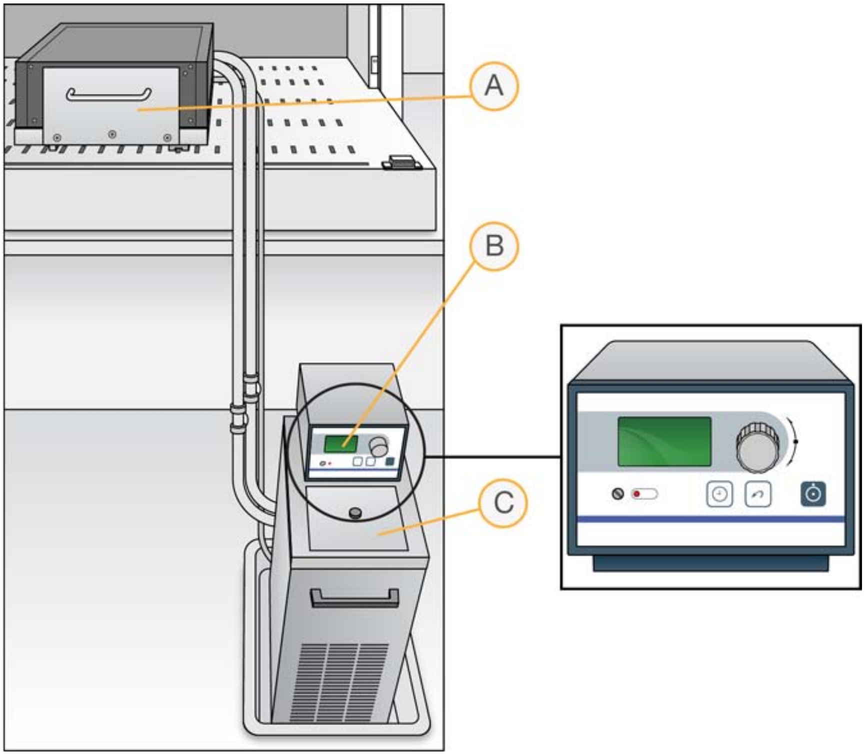

A Chamber Rack on robot Bed
B Water Circulator with Programmable Temperature Controls
C Reservoir Cover

3 The temperature displayed on the water circulator LCD screen may differ from the actual temperature on the Chamber Rack. Confirm the actual temperature using the temperature probe for the Chamber Rack.

4 You must remove bubbles trapped in the Chamber Rack ***each time*** you run this process. Follow instructions in the *Te-Flow (Tecan Flow-Through Module) Operating Manual*, Tecan Doc ID 391584.

5 Use the Illumina Temperature Probe in several locations to ensure that the Chamber Rack is at 44° C.

NOTE
Do not leave the temperature probe in the first three rows of the Chamber Rack. Reserve this space for BeadChips.

Figure 61 IlluminaTemperature Probe and Temperature Probe in Chamber Rack

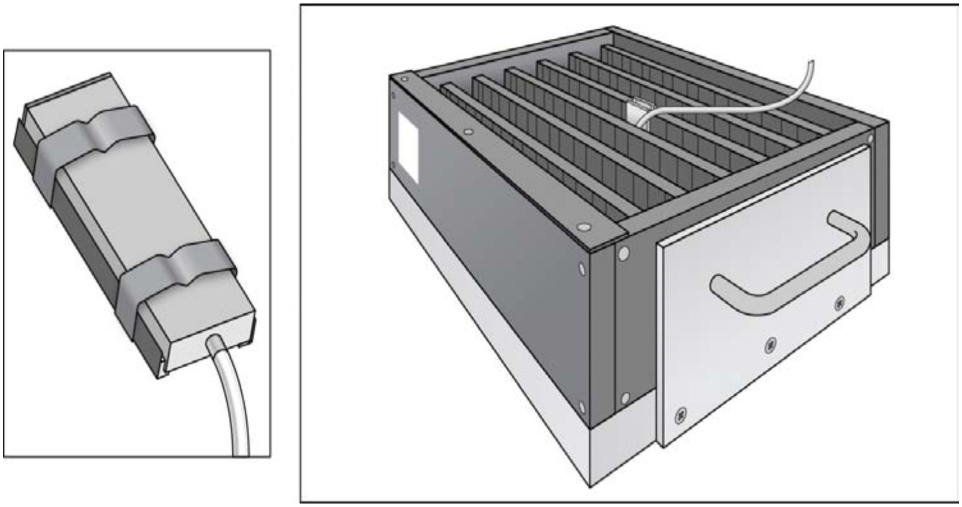

6 For accurate temperature measurement, ensure the Temperature Probe is touching the base of the Chamber Rack.

7 Confirm the Chamber Rack is seated in column 36 on the robot bed.

8 Slide the Chamber Rack back to ensure it is firmly seated.

NOTE
The remaining steps in this protocol must be performed without interruption.

Single-Base Extension

CAUTION
The remaining steps must be performed without interruption.

1 When the Chamber Rack reaches 44° C, quickly place each Flow-Through Chamber assembly into the Chamber Rack.
For 4 BeadChips, place the Flow-Through Chambers in every other position, starting at 1, in the first row of the Chamber Rack. For larger numbers of BeadChips, fill all positions in the first row, then the second and third.

2 Ensure each Flow-Through Chamber is properly seated on its rack to allow adequate heat exchange between the rack and the chamber.

3 On the lab tracking form, record the Chamber Rack position for each BeadChip.

4 Shake the XC4 bottle vigorously to ensure complete resuspension. If necessary, vortex until completely dissolved.

CAUTION
Do not allow pipette tips to contact BeadChip surface. Touch off in the reservoir of the glass back plate.

5 Into the reservoir of each Flow-Through Chamber, dispense:
   a 150 µl RA1. Incubate for 30 seconds.
   b Repeat 5 times.

Figure 62 Dispensing RA1 into Each Flow-Through Chamber

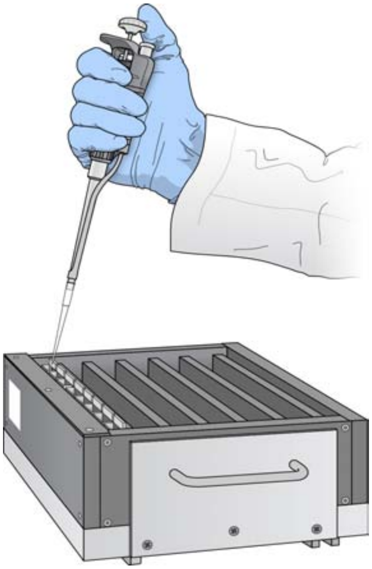

CAUTION
Pipette tip must not contact BeadChip surface.

c 450 µl XC1. Incubate for 10 minutes.
d 450 µl XC2. Incubate for 10 minutes.
e 200 µl TEM. Incubate for 15 minutes.
f 450 µl 95% formamide/1 mM EDTA. Incubate for 1 minute. Repeat once.
g Incubate 5 minutes.
h Begin ramping the Chamber Rack temperature to the temperature indicated on the STM tube, or to 32° C if none is shown.
i 450 µl XC3. Incubate for 1 minute. Repeat once.
j Wait until the Chamber Rack reaches the correct temperature.

Stain BeadChip

1 If you plan on imaging the BeadChip immediately after the staining process, turn on the iScan or HiScan now to allow the lasers to stabilize.

2 Into the reservoir of each Flow-Through Chamber, dispense:
a 250 µl STM and incubate for 10 minutes.
b 450 µl XC3 and incubate for 1 minute. Repeat once, and then wait 5 minutes.
c 250 µl ATM and incubate for 10 minutes.
d 450 µl XC3 and incubate for 1 minute. Repeat once, and then wait 5 minutes.
e 250 µl STM and incubate for 10 minutes.
f 450 µl XC3 and incubate for 1 minute. Repeat once, and then wait 5 minutes.
g 250 µl ATM and incubate for 10 minutes.
h 450 µl XC3 and incubate for 1 minute. Repeat once, and then wait 5 minutes.
i 250 µl STM and incubate for 10 minutes.
j 450 µl XC3 and incubate for 1 minute. Repeat once, and then wait 5 minutes.
k Incubate 5 minutes.

3 Immediately remove the Flow-Through Chambers from the Chamber Rack and place horizontally on a lab bench at room temperature.

Wash and Coat 8 BeadChips

Preparation

Before starting the Wash and Coat process, please read these important notes:

Take the utmost care to minimize the chance of lint or dust entering the wash dishes, which could transfer to the BeadChips. Place wash dish covers on wash dishes when stored or not in use. Clean wash dishes with low-pressure air to remove particulates before use.

In preparation for XC4 BeadChip coating, wash the tube racks and wash dishes thoroughly before and after use. Rinse with DI water. Immediately following wash, place racks and wash dishes upside down on a wash rack to dry.

Place Kimwipes in three layers on the lab bench. Place a tube rack on top of these Kimwipe layers. Do not place on absorbent lab pads. You will place the staining rack containing BeadChips on this tube rack after removing it from the XC4 wash dish.

Prepare an additional clean tube rack that fits the internal dimensions of vacuum desiccator for removal of the BeadChips. Allow one rack per 8 BeadChips. No Kimwipes are required under this tube rack.

Equipment Needed

Place the following items on the bench:

- 1 staining rack
- 1 vacuum desiccator
- 1 tube rack
- Self-locking tweezers
- Large Kimwipes
- Vacuum hose

Steps

1. Set up two top-loading wash dishes, labeled "PB1" and "XC4".
2. To indicate the fill volume before filling wash dishes with PB1 and XC4, pour 310 ml water into the wash dishes and mark the water level on the side. Empty the water from the wash dish. This enables you to pour reagent directly from the PB1

and XC4 bottles into the wash dishes, minimizing contaminant transfer from labware to wash dishes.

Figure 63 PB1 and XC4 Wash Dishes with BeadChip Rack

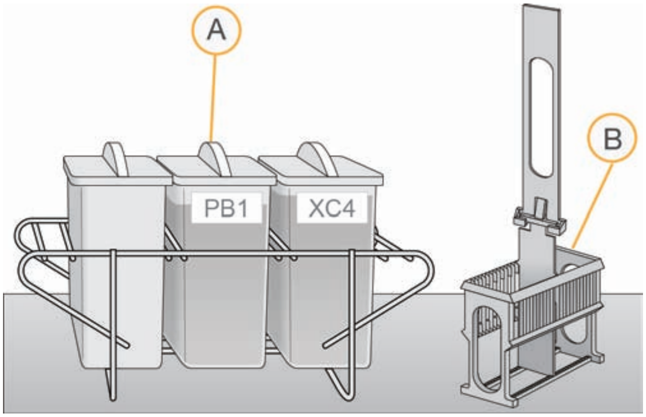

A Wash Dishes
B Staining Rack

3 Pour 310 ml PB1 into the wash dish labeled "PB1."

4 Submerge the unloaded staining rack into the wash dish with the locking arms and tab ***facing toward*** you. This orients the staining rack so that you can safely remove the BeadChips. Let the staining rack sit in the wash dish. You will use it to carry the BeadChips after disassembling the Flow-Through Chambers.

CAUTION
Handle the BeadChips only by the edges or the barcode end. Do not let the BeadChips dry out.

Figure 64 Staining Rack Locking Arms and Tabs

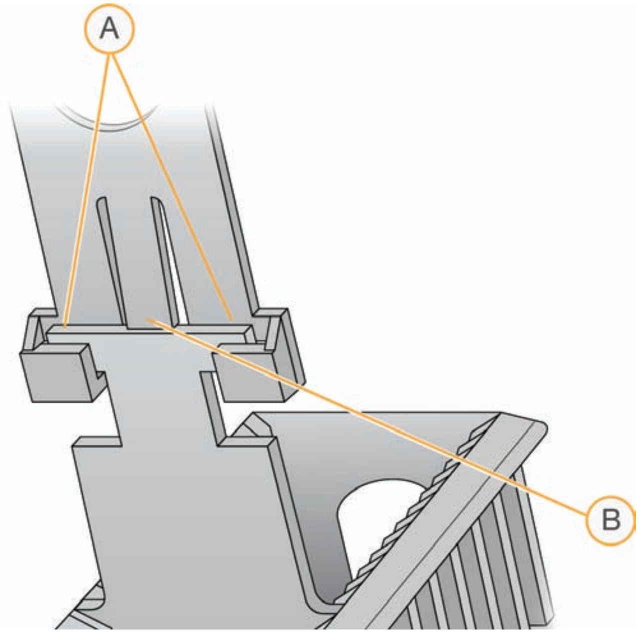

A Locking Arms
B Tabs

CAUTION
If the staining rack handle is not correctly oriented, the BeadChips may be damaged when you remove the staining rack handle before removing the BeadChips.

CAUTION
Do not leave the BeadChips in the PB1 for more than 30 minutes.

CAUTION
Do not let the XC4 sit for more than 10 minutes.

CAUTION
Do not touch the stripes with the wipe or allow EtOH to drip onto the stripes.

5 One at a time, disassemble each Flow-Through Chamber:

a Use the dismantling tool to remove the two metal clamps.

CAUTION
It is important to use the dismantling tool to avoid chipping the glass back plates.

Figure 65 Removing the Metal Clamps from Flow-Through Chamber

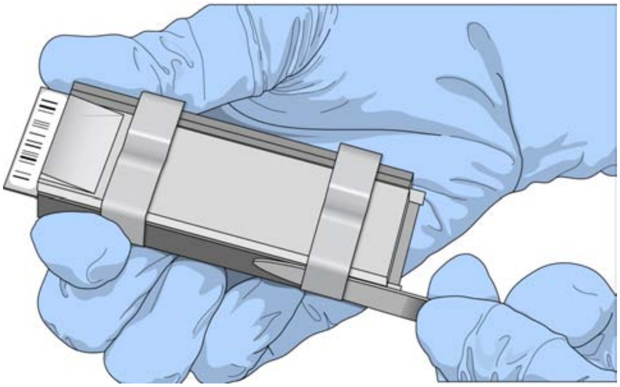

b Remove the glass back plate.

c Set the glass back plate aside. When you finish the XStain BeadChip protocol, clean the glass back plates as described in the SOP.

d Remove the spacer. To avoid damaging the stripes on the BeadChip, pull the spacer out so that the long sides slide along the sides of the BeadChip.

e Remove the BeadChip.

CAUTION
Do not touch the face of the BeadChips. Handle them by the barcode end or by the edges.

6 Place the BeadChips in the staining rack while it is submerged in PB1. Put four BeadChips above the staining rack handle and four below. The BeadChip barcodes should ***face away*** from you; the locking arms on the handle should ***face towards*** you.
If necessary, briefly lift the staining rack out of the wash dish to seat the BeadChip. Replace it immediately after inserting each BeadChip.

7 Ensure that the BeadChips are completely submerged.

CAUTION
Do not allow the BeadChips to dry. Submerge each BeadChip in the wash dish as soon as possible.

8 Move the staining rack up and down 10 times, breaking the surface of the PB1.

NOTE
If the top edges of the BeadChips begin to touch during either PB1 or XC4 washes, gently move the staining rack back and forth to separate the slides. It is important for the solution to circulate freely between all BeadChips.

Figure 66 Washing BeadChips in PB1

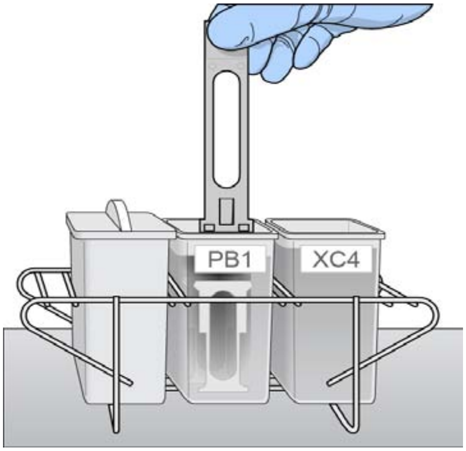

9 Allow the BeadChips to soak for an additional 5 minutes.

NOTE
Do not leave the BeadChips submerged in PB1 for longer than 30 minutes.

10 Shake the XC4 bottle vigorously to ensure complete resuspension.

11 Pour 310 ml XC4 into the dish labeled "XC4," and cover the dish to prevent any lint or dust from falling into the solution.

NOTE
Use the XC4 within 10 minutes after filling the wash dish.

12 Remove the staining rack from the PB1 dish and place it directly into the wash dish containing XC4. For proper handling and coating, The barcode labels on the BeadChips must ***face away*** from you; the locking arms on the handle must ***face towards*** you.

Figure 67 Moving BeadChips from PB1 to XC4

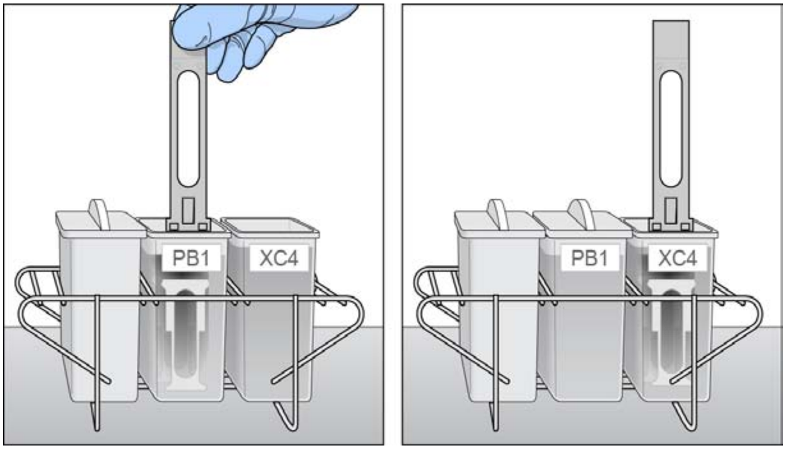

13 Move the staining rack up and down 10 times, breaking the surface of the XC4.

NOTE
If the top edges of the BeadChips begin to touch during either PB1 or XC4 washes, gently move the staining rack back and forth to separate the slides. It is important for the solution to circulate freely between all BeadChips.

14 Allow the BeadChips to soak for an additional 5 minutes.

CAUTION
Use XC4 only once. To process subsequent BeadChips, use a new, clean wash dish with fresh XC4.

15 Prepare a clean tube rack for the staining rack by placing two folded Kimwipes under the tube rack.

16 Prepare one additional tube rack per 8 BeadChips (Illumina-provided from VWR catalog # 60916-748) that fits the internal dimensions of vacuum desiccator

17 Remove the staining rack in one smooth, rapid motion and place it directly on the prepared tube rack, making sure the barcodes *face up* and the locking arms and tabs *face down*.

Figure 68 Staining Rack in Correct Orientation

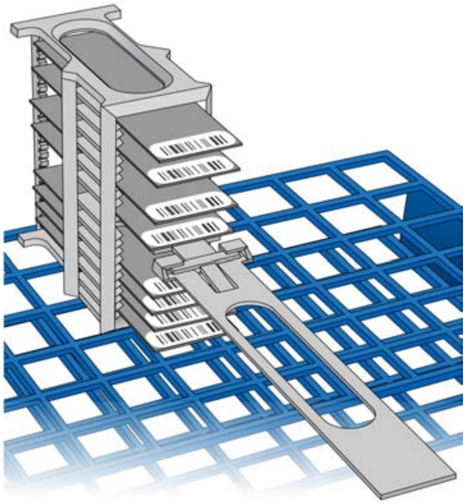

18 To ensure uniform coating, place the staining rack on the center of the tube rack, avoiding the raised edges.

Figure 69 Moving the BeadChip Carrier from XC4 to Tube Rack

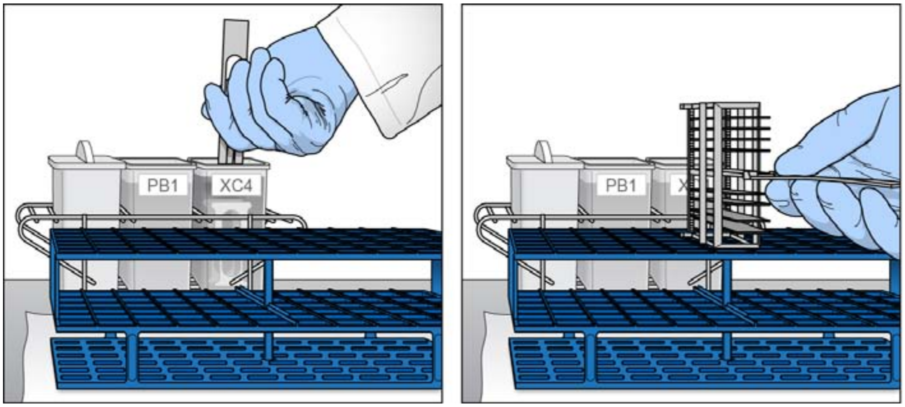

19 For each of the top four BeadChips, working top to bottom:

a Continuing to hold the staining rack handle, carefully grip each BeadChip at its barcode end with self-locking tweezers.

NOTE
The XC4 coat is slippery and makes the BeadChips difficult to hold. The self-locking tweezers grip the BeadChip firmly and help prevent damage.

b Place each BeadChip on a tube rack with the barcode *facing up and towards* you.

Figure 70 BeadChips on Tube Rack

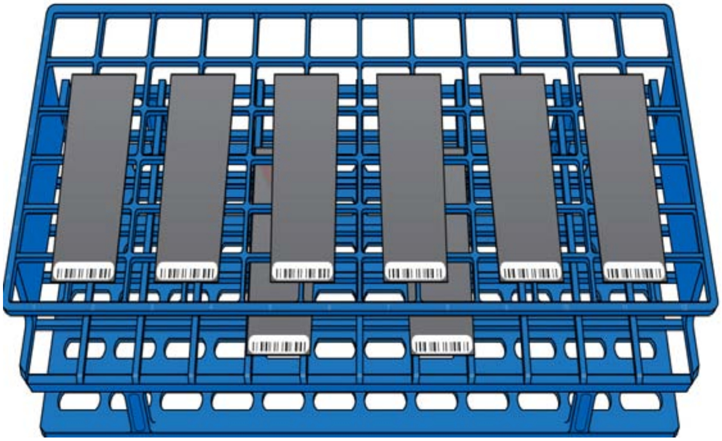

20 Holding the top of the staining rack in position, gently remove the staining rack handle by grasping the handle between the thumb and forefinger. Push the tab up with your thumb and push the handle away from you (unlocking the handle), then pull up the handle and remove.

Figure 71 Removing Staining Rack Handle

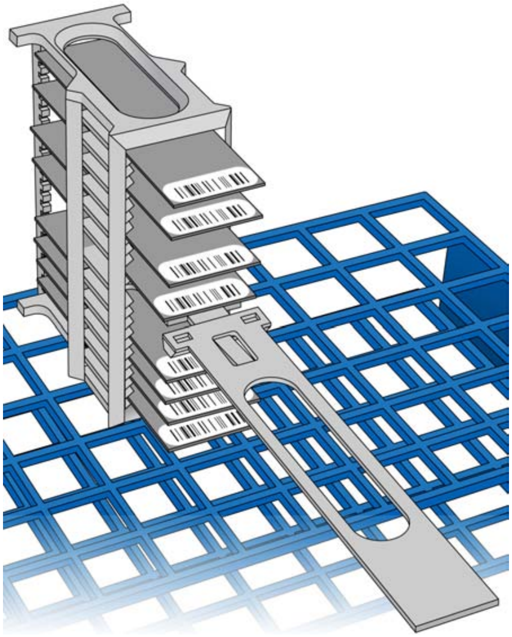

A Tabs
B Handles

21 Remove the remaining BeadChips to the tube rack as shown in the figure above, with six BeadChips on top of the rack and two BeadChips on the bottom. The barcode ends should be towards you, and the BeadChips should be completely horizontal.

CAUTION
To prevent wicking and uneven drying, do not allow the BeadChips to rest on the edge of the tube rack or to touch each other while drying.

22 Place the tube rack in the vacuum desiccator. Each desiccator can hold one tube rack (8 BeadChips).

CAUTION
Ensure the vacuum valve is seated tightly and securely.

23 Remove the red plug from the three-way valve before applying vacuum pressure.

24 Start the vacuum, using at least 508 mm Hg (0.68 bar).

25 To ensure that the desiccator is properly sealed, gently lift the lid of the vacuum desiccator. It should not lift off the desiccator base.

Figure 72 Testing Vacuum Seal

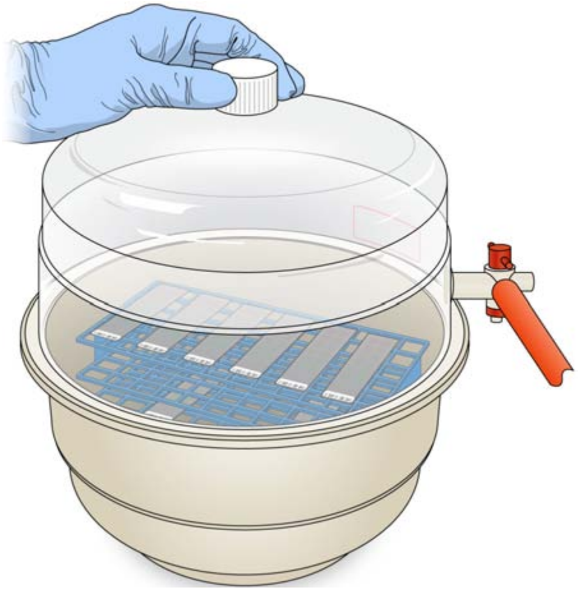

26 Dry under vacuum for 50–55 minutes.
Drying times may vary according to room temperature and humidity.

27 Release the vacuum by turning the handle very slowly.

WARNING
Air should enter the desiccator very slowly to avoid disturbing the contents. Improper use of the vacuum desiccator can result in damage to the BeadChips. This is especially true if you remove the valve plug while a vacuum is applied. For detailed vacuum desiccator instructions, see the documentation included with the desiccator.

28 Store the desiccator with the red valve plug in the desiccator's three-way valve to stop accumulation of dust and lint within the valve port.

29 Touch the borders of the chips (**do not touch the stripes**) to ensure that the etched, barcoded side of the BeadChips are dry to the touch.

30 If the underside feels tacky, manually clean the underside of the BeadChip to remove any excess XC4. The bottom two BeadChips are most likely to have some excess.

   a Hold the BeadChip at a downward angle to prevent excess EtOH from dripping from the wipe onto the stripes.

   b Wipe along the underside of the BeadChip five or six times, until the surface is clean and smooth.

CAUTION
Do not touch the stripes.

31 Clean the glass back plates. For instructions, see the SOP.

32 Clean the Hyb Chambers:

   a Remove the rubber gaskets from the Hyb Chambers.

   b Rinse all Hyb Chamber components with DI water.

   c Thoroughly rinse the eight humidifying buffer reservoirs.

33 Discard unused reagents in accordance with facility standards.

CAUTION
Handle the BeadChips only by the edges or the barcode end. Do not let the BeadChips dry out.

CAUTION
Do not leave the BeadChips in the PB1 for more than 30 minutes.

CAUTION
Do not touch the stripes with the wipe or allow EtOH to drip into the stripes.

34 Do one of the following:

- Proceed to *Illumina GenomeStudio* on page 112, or *Image BeadChip* on page 110.
- Store the BeadChips in the Illumina BeadChip Slide Storage Box inside a vacuum desiccator at room temperature. Image the BeadChips within 72 hours.

Image BeadChip

The Illumina iScan or HiScan Systems scan the BeadChip, using a laser to excite the fluorophore of the single-base extension product on the beads. The scanner records high-resolution images of the light emitted from the fluorophores. See the chapter on imaging BeadChips in the SOP.

Figure 73 Imaging BeadChip

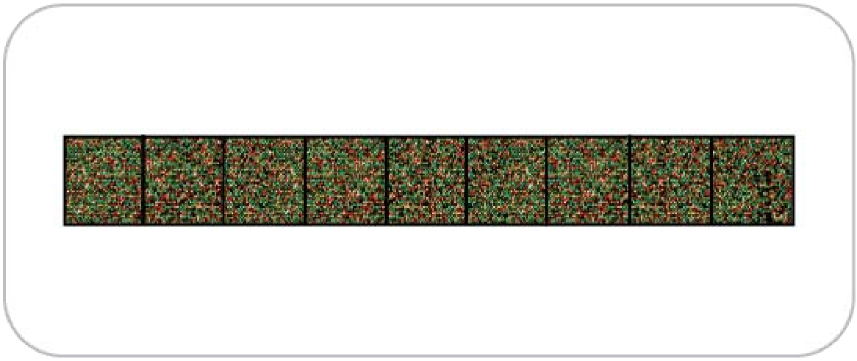

Image BeadChip on the iScan System

The iScan™ Reader is an easy-to-use, laser-based, high-resolution benchtop optical imaging system that can rapidly scan and collect large volumes of data from Illumina DNA analysis and RNA analysis high-density BeadChips.

Image BeadChip on the HiScan System

The HiScan™ System is an easy-to-use, laser-based, high-resolution benchtop optical imaging system that integrates the high-throughput capability of genotyping and gene expression arrays with the power and resolution of next-generation sequencing, delivering unprecedented flexibility for experimental design.

Scanning Settings for Imaging BeadChips on the HiScan and iScan Systems

Use the **Methylation NXT** setting when scanning BeadChips using the HiScan or iScan systems. For general information about scan settings, see the *iScan System User Guide* or

HiScanSQ System User Guide.

Illumina GenomeStudio

Illumina GenomeStudio, Illumina's integrated data analysis software platform, provides a common environment for analyzing data obtained from microarray and sequencing technologies. Within this common environment, or framework, the Illumina GenomeStudio software modules allow you to perform application-specific analyses. The Illumina GenomeStudio Methylation Module, included with your Illumina Infinium Methylation Assay system, is an application for extracting genome-wide DNA methylation data from data files collected from systems such as the Illumina HiScan Reader. Experiment performance is based on built-in controls that accompany each experiment.

Data analysis features of the Illumina GenomeStudio Methylation Module include:

- Choice of assay analysis within a single application
- Data tables for information management and manipulation
- Plotting and graphing tools
- Whole-genome display of sample data in the IGV (Illumina Genome Viewer)
- Data visualization of one or more samples in the ICB (Illumina Chromosome Browser)
- Data normalization
- Custom report file formats
- Differential methylation analysis
- Assay-specific controls dashboards

For feature descriptions and instructions on using the Illumina GenomeStudio platform to visualize and analyze genome-wide DNA methylation data, see the *GenomeStudio Framework User Guide* and the *GenomeStudio Methylation Module User Guide*.

For technical assistance, contact Illumina Technical Support.

Automated Protocol

Chapter 3

Introduction to Infinium HD Methylation Automated Protocol 114
Infinium HD Methylation Automated Workflow 115
Equipment, Materials, and Reagents 117
Quantitate DNA (Optional) 121
Make BCD Plate 131
Make MSA4 Plate 137
Incubate MSA4 Plate 144
Fragment MSA4 Plate 145
Precipitate MSA4 Plate 150
Resuspend MSA4 Plate 157
Hybridize Multi BeadChip 162
Wash BeadChip 180
Single-Base Extension and Stain BeadChip 192
Image BeadChip 227
Illumina GenomeStudio 229

Introduction to Infinium HD Methylation Automated Protocol

This chapter describes pre- and post-amplification automated laboratory protocols for the Illumina Infinium HD Methylation Assay. Follow the protocols in the order shown.

Before performing the protocols in this chapter, use the Zymo EZ DNA Methylation Kit to bisulfite convert the genomic DNA. For the automated Infinium Methylation assay, at least 1 µg of DNA is required. For instructions on bisulfite-converting gDNA, see *Make BCD Plate* on page 131.

Infinium HD Methylation Automated Workflow

The following figure graphically represents the Illumina Infinium HD Methylation Assay automated workflow for 8 BeadChips. These protocols describe the procedure for preparing 96 DNA samples.

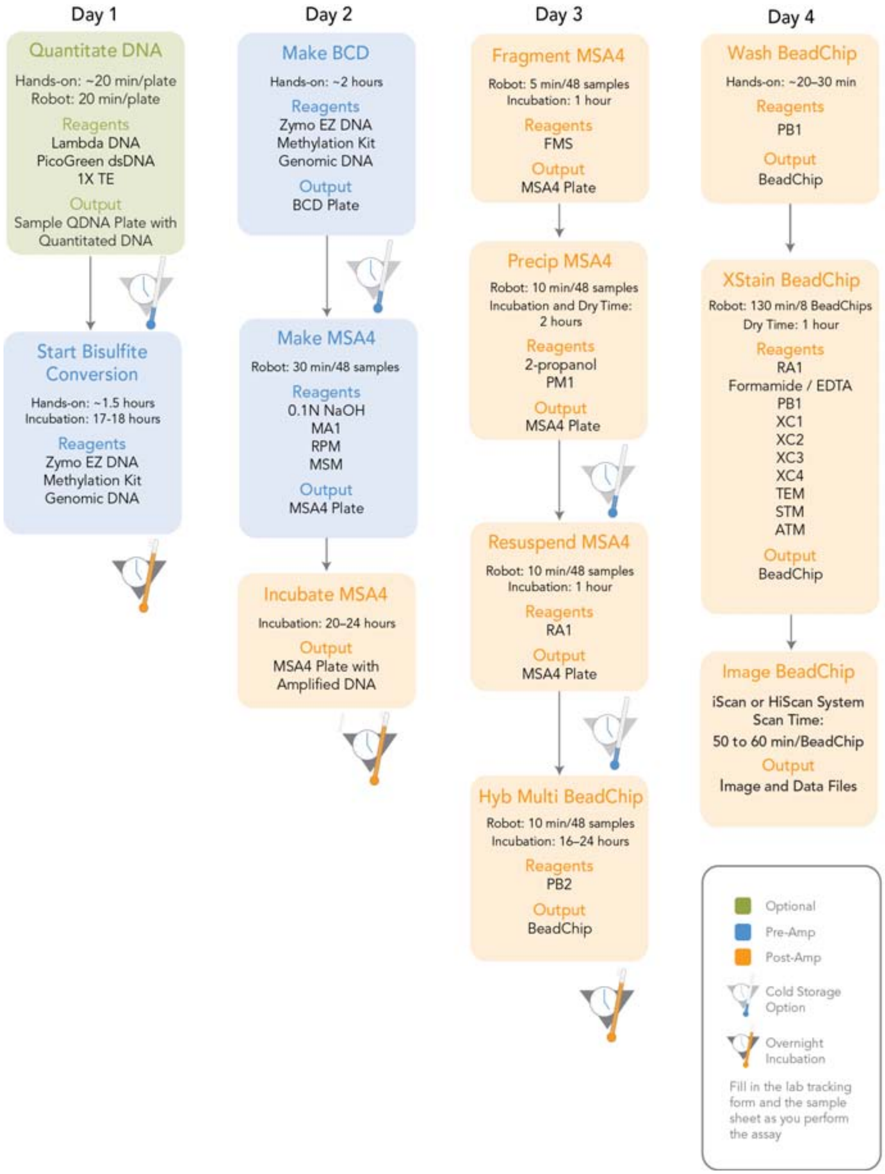

Figure 74 Illumina Infinium HD Methylation Assay Automated Workflow

Equipment, Materials, and Reagents

These materials are specifically required for the automated Illumina Infinium HD Methylation Assay. For a list of other equipment, materials, and reagents needed in an Illumina Infinium HD Methylation Assay lab, see the *Infinium Assay Lab Setup and Procedures Guide*.

User-Supplied Equipment

Table 10 User-Supplied Equipment Methylation Assay (Automated)

Item	Suggested Vendor
Vacuum desiccator (1 per 8 BeadChips processed simultaneously)	VWR International catalog # 24988-197, www.vwr.com
Vacuum tubing	VWR International catalog # 62995-335, www.vwr.com
2 Tecan eight-tip robots (one for pre- and one for post-amplification processes)	Non-LIMS customers<br>• SC-30-401 (110V) - North America and Japan<br>• SC-30-402 (220V) - EU and Asia Pacific (Except Japan)
Carboy > 10 L, 2 per robot, Pre-PCR	
Forceps	VWR International catalog # 25601-008, www.vwr.com
Auto-desiccator cabinet (Optional—allows scanning of BeadChips up to three days after processing)	VWR International, Catalog # 74950-342, www.vwr.com

Illumina-Supplied Equipment

Table 11 Illumina-Supplied Materials

Item	Catalog or Part #
Multi-Sample BeadChip Alignment Fixture	Part # 218528
Robot BeadChip Alignment Fixture (6)	Part # 222691

User-Supplied Materials

Table 12 User-Supplied Materials Methylation Assay (Automated)

Item	Suggested Vendor
96-well, black, flat-bottom Fluotrac 200 plates	Greiner, catalog # 655076 www.gbo.com
Aluminum foil	
Foil adhesive seals (Microseal "F")	MJ Research, Catalog # MSF-1001, www.mjr.com
Reservoir, full, 150 ml	Beckman Coulter, catalog # 372784, www.beckmancoulter.com
Reservoir, half, 75 ml	Beckman Coulter, catalog # 372786, www.beckmancoulter.com
Reservoir, quarter, 40 ml	Beckman Coulter, catalog # 372790, www.beckmancoulter.com
Reservoir frames, 2 (per TECAN)	Beckman Coulter, catalog # 372795, www.becmancoulter.com
Tube racks for vacuum desiccator (1 for every 8 BeadChips to be processed simultaneously; must fit internal dimensions of vacuum desiccator)	VWR catalog # 66023-526, www.vwr.com
Vacuum source (greater than 508 mm Hg (0.68 bar)	
Vacuum gauge for vacuum desiccator (recommended)	

Illumina-Supplied Materials

- WG#-MSA4 barcode labels
- WG#-BCD barcode labels

- WG#-DNA barcode labels
- WG#-QNT barcode labels

Illumina-Supplied Reagents

Table 13 Illumina-Supplied Reagents

Item	Part #
**ATM**—Anti-Stain Two-Color Master Mix	11208317
**FMS**—Fragmentation solution	11203428
**MA1**—Multi-Sample Amplification 1 Mix	11202880
**RPM**—Random Primer Mix	15010230
**MSM**—Multi-Sample Amplification Master Mix	11203410
**PB1**—Reagent used to prepare BeadChips for hybridization	11291245
**PB2**—Humidifying buffer used during hybridization	11191130
**PM1**—Precipitation solution	11292436
**RA1**—Resuspension, hybridization, and wash solution	11292441
**STM**—Superior Two-Color Master Mix	11288046
**TEM**—Two-Color Extension Master Mix	11208309
**XC1**—XStain BeadChip solution 1	11208288
**XC2**—XStain BeadChip solution 2	11208296
**XC3**—XStain BeadChip solution 3	11208392
**XC4**—XStain BeadChip solution 4	11208430

Quantitate DNA (Optional)

This process uses the PicoGreen dsDNA quantitation reagent to quantitate double-stranded DNA samples. You can quantitate up to three plates, each containing up to 96 samples. If you already know the concentration, proceed to *Make BCD Plate* on page 131.

Illumina recommends the Molecular Probes PicoGreen assay to quantitate dsDNA samples. The PicoGreen assay can quantitate small DNA volumes, and measures DNA directly. Other techniques may pick up contamination such as RNA and proteins. Illumina recommends using a spectrofluorometer because fluorometry provides DNA-specific quantification. Spectrophotometry might also measure RNA and yield values that are too high.

Estimated Time

Hands-on time: ~20 minutes per plate

Robot: 20 minutes per plate

Consumables

Item	Quantity	Storage	Supplied By
PicoGreen dsDNA quantitation reagent	See Instructions	-15° to -25° C	User
1X TE (10 mM Tris-HCl pH8.0, 1 mM EDTA (TE))	See Instructions	Room temperature	User
Lambda DNA	See Instructions	2° to 8° C	User
96-well 0.65 ml microtiter plate	1 per 96 samples		User
Fluotrac 200 96-well flat-bottom plate	1 per Std DNA plate<br>1 per Sample DNA plate		User

NOTE
PicoGreen is susceptible to differential contaminants. False positives may occur for whole-genome amplification. Therefore, it is important to quantitate the input into the whole-genome amplification reaction.

Preparation

- Thaw PicoGreen to room temperature in a light-impermeable container.
- Follow the instructions for preparing the robot before each use in the SOP.
- Thaw the sample DNA plates to room temperature.
- Apply a QDNA barcode label to a new Fluotrac plate for each GS#-DNA plate to be quantified.
- Hand-label the microtiter plate “Standard DNA.”
- Hand-label one of the Fluotrac plates “Standard QDNA.”
- In the Sample Sheet, enter the Sample_Name (optional) and Sample_Plate for each Sample_Well.

Make Standard DNA Plate

In this process, you create a Standard DNA plate with serial dilutions of stock Lambda DNA in the wells of column 1.

1. Add stock Lambda DNA to well A1 in the plate labeled “Standard DNA” and dilute it to 75 ng/µl in a final volume of 233.3 µl. Pipette up and down several times.
   a. Use the following formula to calculate the amount of stock Lambda DNA to add to A1:

(233.3 µl) X (75 ng/µl)	=	µl of stock Lambda DNA to add to A1
(stock Lambda DNA concentration)		

   b. Dilute the stock DNA in well A1 using the following formula:
      µl of 1X TE to add to A1 = 233.3 µl - µl of stock Lambda DNA in well A1
2. Add 66.7 µl 1X TE to well B1.
3. Add 100 µl 1X TE to wells C, D, E, F, G, and H of column 1.

Figure 75 Dilution of Stock Lambda DNA Standard

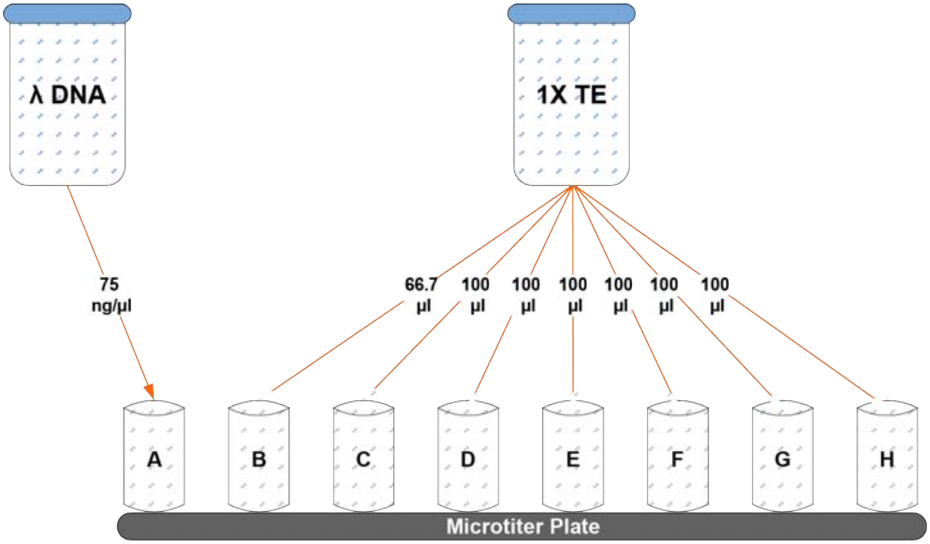

4 Transfer 133.3 µl of Lambda DNA from well A1 into well B1. Pipette up and down several times.

5 Change tips. Transfer 100 µl from well B1 into well C1. Pipette up and down several times.

6 Repeat for wells D1, E1, F1, and G1, changing tips each time. **Do not transfer from well G1 to H1.** Well H1 serves as the blank 0 ng/µl Lambda DNA.

Table 14 Concentrations of Lambda DNA

Row-Column	Concentration (ng/µl)	Final Volume in Well (µl)
A1	75	100
B1	50	100
C1	25	100
D1	12.5	100

Row-Column	Concentration (ng/µl)	Final Volume in Well (µl)
E1	6.25	100
F1	3.125	100
G1	1.5262	200
H1	0	100

Figure 76 Serial Dilutions of Lambda DNA

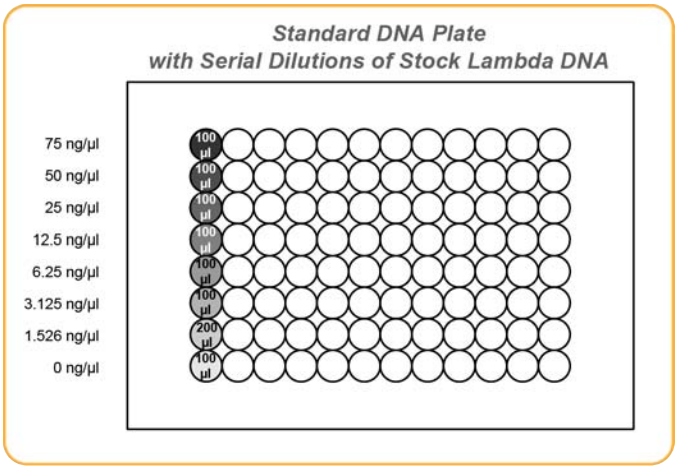

7 Cover the Standard DNA plate with cap mat.

Dilute PicoGreen

The diluted PicoGreen will be added to both the Standard QDNA and Sample QDNA plates, to make the DNA fluoresce when read with the spectrofluorometer.

CAUTION
PicoGreen reagent degrades quickly in the presence of light. Also, do not use glass containers for PicoGreen reagent, because it adheres to glass, thereby lowering its effective concentration in solution and effecting the upper response range accuracy.

1 Prepare a 1:200 dilution of PicoGreen into 1X TE, using a sealed 100 ml or 250 ml Nalgene bottle wrapped in aluminum foil.
Refer to the following table to identify the volumes needed to produce diluted reagent for multiple 96-well QDNA plates. For fewer than 96 DNA samples, scale down the volumes.

Table 15 Volumes for PicoGreen Reagents

# QDNA Plates	PicoGreen Volume (µl)	1X TE Volume (ml)
1	115	23
2	215	43
3	315	63

2 Cap the foil-wrapped bottle and vortex to mix.

Create QDNA Standard and Sample Plates

In this process, PicoGreen is distributed to Standard QDNA and Sample QDNA Fluotrac plates and mixed with aliquots of DNA from the respective DNA plates.

CAUTION
Do not run any other programs or applications while using the Tecan robot. Your computer and the robot may lock up and stop a run.

1 At the robot PC, select **GTS Pre-PCR Tasks | DNA Prep | Make QDNA**.

2 In the DNA Plate Selection dialog box, select the plate type of the Standard DNA and Sample DNA plates. They should all be MIDI plates, TCY plates or ABGN plates. Roll the mouse pointer over each picture to see a description of the plate.

Figure 77 DNA Plate Selection Dialog Box

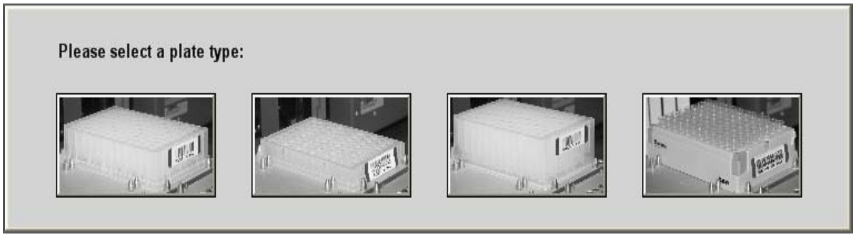

3 In the Basic Run Parameters pane, enter the **Number of DNA/QDNA plates** (1, 2, or 3 pairs) and the **Number of DNA Samples**.
The robot PC updates the Required Run Item(s) and the bed map to show the correct position of items on the robot bed. All barcodes must face to the right.

Figure 78 Make QDNA Screen

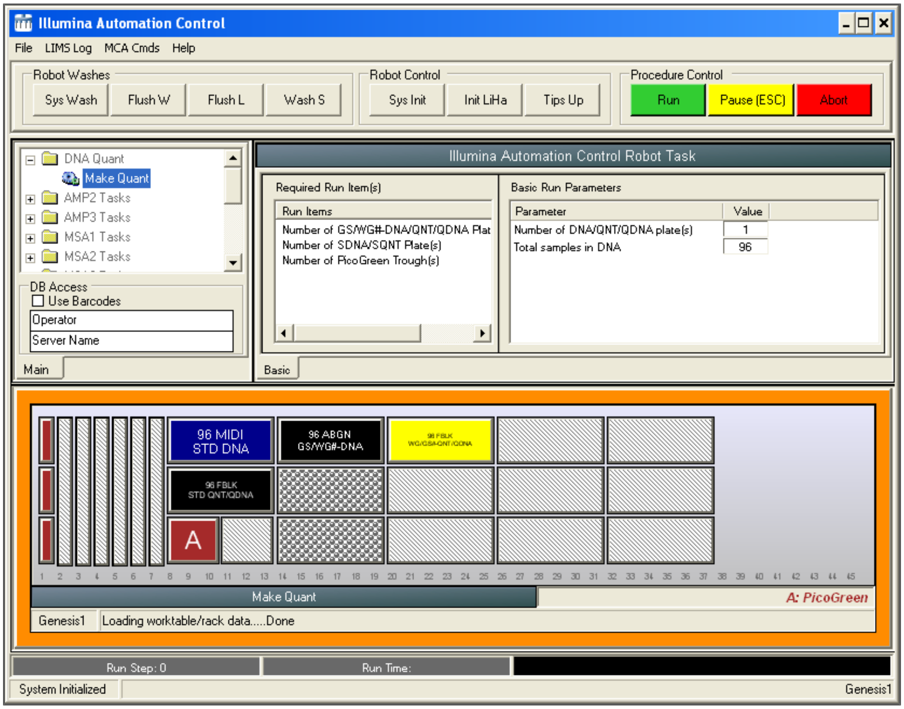

4 Vortex the GS#-DNA Sample plate at 1450 rpm for 1 minute.

5 Centrifuge the GS#-DNA Sample plate to 280 xg for 1 minute.

6 Vortex the Standard DNA plate at 1450 rpm for 1 minute.

7 Centrifuge the Standard DNA plate to 280 xg for 1 minute.

8 Place the GS#-DNA Sample, Standard DNA, Standard QDNA, and QDNA Sample plates on the robot bed according to the robot bed map. Place well A1 at the top-left corner of its robot bed carrier. Remove any plate seals.

9 Pour the PicoGreen dilution into half reservoir A and place it on the robot bed.

10 Make sure that all items are placed properly on the robot bed, that all seals have been removed, and that all the barcodes face to the right.

11 On the Lab Tracking Forms, record the position of the plates on the robot bed.

12 Clear the **Use Barcodes** checkbox.

13 Click **Run**. Observe the beginning of the robot run to ensure there are no problems.
The robot transfers 195 µl of diluted PicoGreen to all Fluotrac plates, then transfers 2 µl aliquots of DNA from Standard DNA plate to Standard QDNA plate and from GS#-DNA plate to sample QDNA plates.
The robot PC sounds an alert and displays a message when the process is complete.

14 Click **OK** in the message box.

15 On the Lab Tracking Forms, record:
- Date/Time
- Operator
- Robot
- The QDNA barcode that corresponds to each GS#-DNA barcode
- The Standard QDNA plate that corresponds to each Standard DNA plate

16 After the robot finishes, immediately seal all plates:
a Place foil adhesive seals over Sample QDNA and Standard QDNA plates.
b Place cap mats on GS#-DNA Sample and Standard DNA plates.

17 Discard unused reagents in accordance with facility requirements.

18 Store the GS#-DNA and Standard DNA plates at 2° to 8° C or -15° to -25° C.

19 Centrifuge the Sample QDNA Plate and Standard QDNA plates to 280 xg for 1 minute.

Read QDNA Plate

In this process, you use the Gemini XS or XPS Spectrofluorometer along with the Illumina Fluorometry Analysis software to read the Standard QDNA and Sample QDNA plates. You use the software to create a standard curve based on the quantities of Standard DNA with PicoGreen. Then you read the Sample QDNA plates to compare

their data against the standard curve to obtain the concentration of sample DNA. For the best performance, Illumina recommends a minimum concentration of 50 ng/µl.

1 Turn on the spectrofluorometer.

2 At the PC, open the Illumina Fluorometry Analysis program.

Figure 79 Illumina Fluorometry Analysis Main Screen

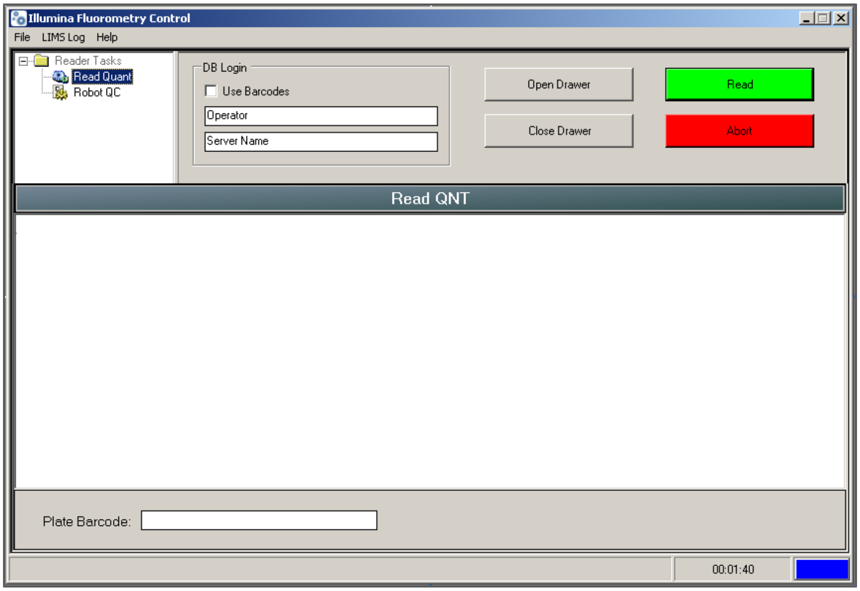

3 Select **Reader Tasks | Read Quant**.

4 Clear the **Use Barcodes** checkbox.

5 Click **Read**.

6 When asked if you want to read a new Standard plate, click **Yes**.

7 Remove the plate seal and load the Standard QDNA plate into the fluorometry tray. Click **OK**. The spectrofluorometer will read the plate data.

8 Review the data from the Standard QDNA plate. Either accept it and go on to the next step, or reject it. Rejecting the data will stop the read Quant process.

9 Remove the Standard QDNA place from the spectrofluorometer tray

10 When prompted, enter the number of plates you want to read (1, 2, or 3). Do not include the Standard QDNA plate in this number. Click **OK**.

11 When prompted, hand-scan the Sample QDNA plate barcode. Click **OK**.

12 When prompted, remove the plate seal from the Sample QDNA plate and load it into the spectrofluorometer tray, with well A1 at the upper left corner. Click **OK**. The spectrofluorometer will read the plate data.

13 Remove the Standard QDNA plate from the spectrofluorometer tray.

14 When prompted, click **Yes** to review the raw Sample QDNA plate data.

Figure 80 Sample QDNA Data

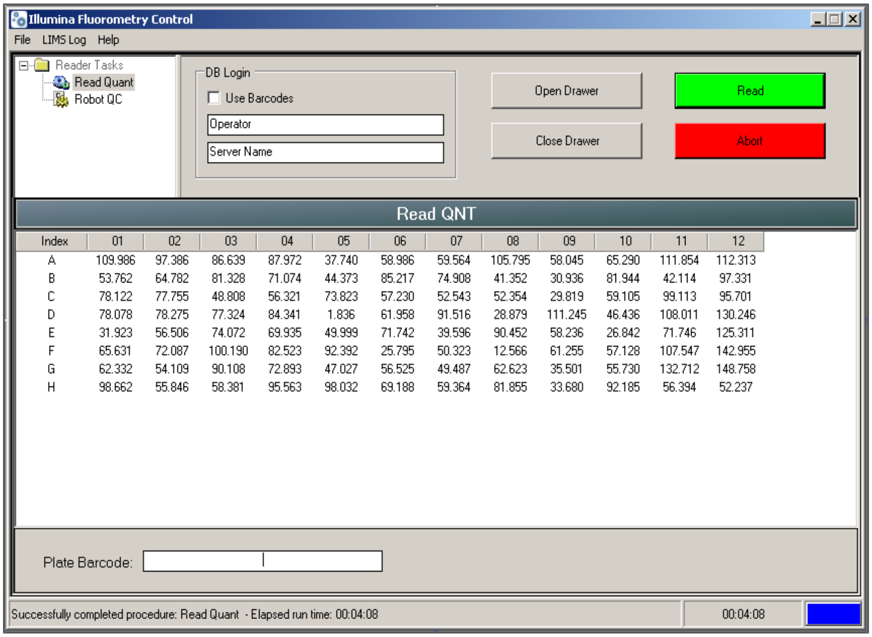

15 Microsoft Excel opens automatically at the same time and displays the quantitation data for the Sample QDNA plate. There are three tabs in the file:

- **SQDNA_STD**—Generates the standard curve by plotting the Relative Fluorescence (RF) values measured in the Standard QDNA plate against assumed concentrations in the Standard DNA Plate.

- **QDNA**—Plots the concentration (ng/µl) of each well of the Sample QDNA Plate as derived from the standard curve.
- **Data**—A readout of the raw data values for the Standard QDNA plate and the Sample QDNA Plate.

16 The Illumina Fluorometry Analysis software will prompt you to indicate whether you wish to save the QDNA data shown in an Excel file. Select the option you prefer:
- Click **Yes**to save.
- Click **No** to delete the quant data. You can read the same plate for quant data repeatedly.

17 If you entered more than one Sample QDNA plate to read, repeat steps 11 to 14 for each additional plate.

18 Discard the QDNA plates and reagents in accordance with facility requirements.

19 Do one of the following:
- Proceed to *Make BCD Plate* on page 131.
- Store the Sample QDNA plate at 2° to 8° C for up to one month.

Make BCD Plate

This process uses the Zymo EZ DNA Methylation Kit to convert unmethylated cytosines (C) in genomic DNA to uracil (U), while leaving methylated cytosines (C) unchanged for methylation analysis.

Figure 81 Bisulfite Conversion

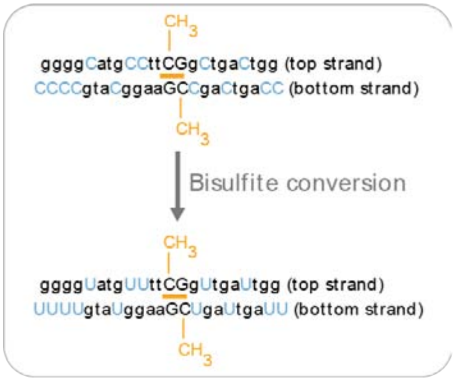

Methylation detection in bisulfite-converted DNA (BCD) is based on the different sensitivity of cytosine and 5-methylcytosine to deamination by bisulfite. Under acidic conditions, cytosine undergoes conversion to uracil, while methylated cytosine remains unreactive. An effective bisulfite-conversion protocol is a necessary prerequisite for a successful Illumina Infinium HD Methylation Assay. Incomplete conversion of cytosine to uracil can result in false-positive methylation signals, and can reduce the overall quality of the assay data.

NOTE
Always perform bisulfite conversion of DNA in the Pre-AMP area.

Figure 82 Bisulfite Conversion Workflow

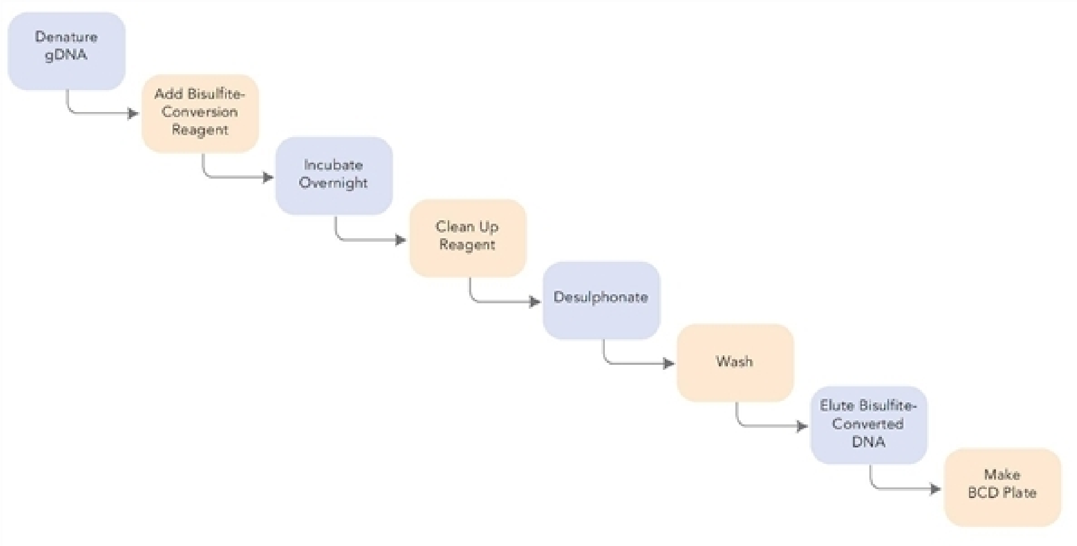

Bisulfite-convert the genomic DNA samples using the Zymo EZ DNA Methylation Kit. Transfer the bisulfite-converted samples to the BCD plate.

Estimated Time

Hands-on time:

- ~1.5 hours on Day 1
- ~2 hours on Day 2

Incubation time: 16–17 hours on Day 1 (Overnight)

Consumables

Item	Quantity	Storage	Supplied By
Zymo EZ DNA Methylation kit (includes bisulfite-conversion reagent, dilution buffer, desulphonation buffer, elution buffer)	1 kit per 50 samples<br>1 kit per 200 samples<br>or<br>1 kit per 2 plates	Room temperature	User
96-well 0.2 ml skirted microplate	1–3 plates		User
Genomic DNA	≥ 500 ng for each bisulfite conversion reaction for manual process.<br><br>≥1000 ng for each bisulfite conversion reaction for the Automated process		User

Illumina recommends using the Zymo Research EZ DNA methylation kit for bisulfite conversion of genomic DNA. Follow Illumina recommended incubation conditions during bisulfite conversion to maximize DNA conversion rate.

For all other steps, follow the manufacturer's instructions for use, because the protocols vary significantly for different kits.

CAUTION
Bisulfite-conversion kits that are not specified in this guide are not supported for use with the Illumina Infinium HD Methylation Assay.

Preparation

Use this table to determine the correct amount of genomic DNA for each bisulfite-conversion process.

Table 16 Genomic DNA Input for Bisulfite Conversion

	Amount of gDNA	Elution Buffer	Number of Activation Reactions Supported
Manual process	> 500 ng	12 µl	Enough for two replicates
Automated process	1000 ng	22 µl	Enough for two replicates

- Prepare the conversion reagent according to the manufacturer's instructions. For best results, use it immediately.

CAUTION
The conversion reagent is photosensitive, so minimize its exposure to light.

- Prepare the wash buffer according to the manufacturer's instructions.
- Apply a BCD barcode label to each new 96-well 0.2 ml skirted microplate.
- On the Lab Tracking Forms, record:
  - Date and time
  - Operator

NOTE
To record information about your assay such as operator information, start and stop times, and barcodes, use the lab tracking form provided at http://www.illumina.com/documentation. This form can be filled out and saved online, or printed and filled in by hand.

Steps to Make BCD Plate

Day 1

The following steps are intended only to provide an overview of the process. For specific instructions, use the manufacturer's documentation.

1. Follow the instructions in the Zymo EZ DNA Methylation Kit to denature the genomic DNA and add conversion reagent.
2. Incubate in a thermocycler using the following conditions for 16 cycles:
   - 95° C for 30 seconds
   - 50° C for 1 hour

NOTE
Denaturation is necessary for bisulfite conversion since the conversion reagent only works on single-stranded DNA.

3 Hold DNA at 4° C for 10 minutes in the thermocycler until you are ready to do the clean-up.

Day 2

1 Follow the instructions in the Zymo EZ DNA Methylation Kit to do the following:
   a Clean the samples using the provided spin columns or filter plate. Wash off the remaining conversion reagent.

NOTE
When using the Zymo EZ DNA Methylation Kit, centrifuge the plate to between 3000 and 5000 xg for optimal performance.

   b Desulphonate the column or plate with desulphonation buffer. Incubate at room temperature for 15 minutes.
   c Clean the samples and wash twice to remove the desulphonation buffer.
   d Add elution buffer:
      — 12 µl for manual process from 500 ng gDNA
      — 22 µl for automated process from 1 µg of gDNA
   e Centrifuge to elute.

NOTE
When using the Zymo EZ DNA Methylation Kit, centrifuge the plate to between 3000 and 5000 xg for 5 minutes for optimal performance.

2 Transfer the bisulfite-converted DNA (BCD) samples to the BCD plate.

3 On the lab tracking form, record the WG#-BCD and BCD plate barcodes.

4 Heat-seal the plate and store it at -15° to -25° C. Thaw the plate completely and vortex to mix contents before using it in an assay.

CAUTION
Do not store BCD for more than one month at -15° to -25° C.

SAFE STOPPING POINT
This is a good stopping point in the process.

Make MSA4 Plate

This process creates a MSA4 plate for BCD amplification. MA1 is first added to the MSA4 plate, followed by the BCD samples. 0.1N NaOH is added to denature the BCD samples. The RPM reagent neutralizes the sample. MSM (Multi-Sample Amplification Master Mix) is then added to the BCD samples.

Figure 83 Denaturing and Neutralizing BCD

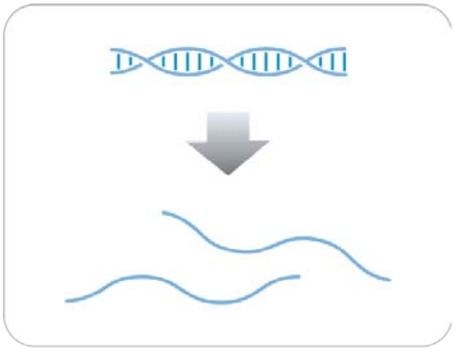

Estimated Time

Robot time:

- 30 minutes for 48 samples
- 45 minutes for 96 samples

Incubation time: ~20–24 hours

Consumables

Item	Quantity	Storage	Supplied By
MA1	2 tubes (per 96 samples)	Room temperature	Illumina
RPM	2 tubes (per 96 samples)	-15° to -25° C	Illumina
MSM	2 tubes (per 96 samples)	-15° to -25° C	Illumina
0.1N NaOH	15 ml (per 96 samples)	2° to 8° C	User
96-well 0.8 ml microtiter plate (MIDI)	1 plate		User
BCD plate with Bisulfite-converted DNA samples	1 plate	-15° to -25° C	User

NOTE
Thaw all reagents completely at room temperature and allow to equilibrate. Once thawed, gently invert each tube several times to thoroughly mix the reagent. Pulse centrifuge each tube to 280 xg to eliminate bubbles and collect reagent at the bottom of the tube.

Preparation

- In preparation for the Incubate MSA4 process (*Incubate MSA4 Plate* on page 144), pre-heat the Illumina Hybridization Oven in the post-amp area to 37° C and allow the temperature to equilibrate
- In the Sample Sheet, enter the Sample_Name and Sample_Plate for each Sample_Well.
- Apply an MSA4 barcode label to a new MIDI.
- Thaw MA1, RPM, and MSM tubes to room temperature.
- Thaw BCD samples to room temperature.
- On the Lab Tracking Forms, record:

- Date/Time
- Operator
- Robot
- Batch number
- Number of samples (48 or 96)
- BCD plate barcode(s)
- MSA4 plate barcode(s)
- MA1 tube barcode(s)
- RPM tube barcode(s)
- MSM tube barcode(s)

NOTE
To record information about your assay such as operator information, start and stop times, and barcodes, use the lab tracking form provided at http://www.illumina.com/documentation. This form can be filled out and saved online, or printed and filled in by hand.

Prepare Robot

For instructions on preparing the robot for use in a protocol see the *Infinium Assay Lab Setup and Procedures Guide*.

Refer to the figure shown below throughout this protocol. Note that all of the barcodes face to the right.

Figure 84 Eight-Tip Robot (Make MSA4 Setup)

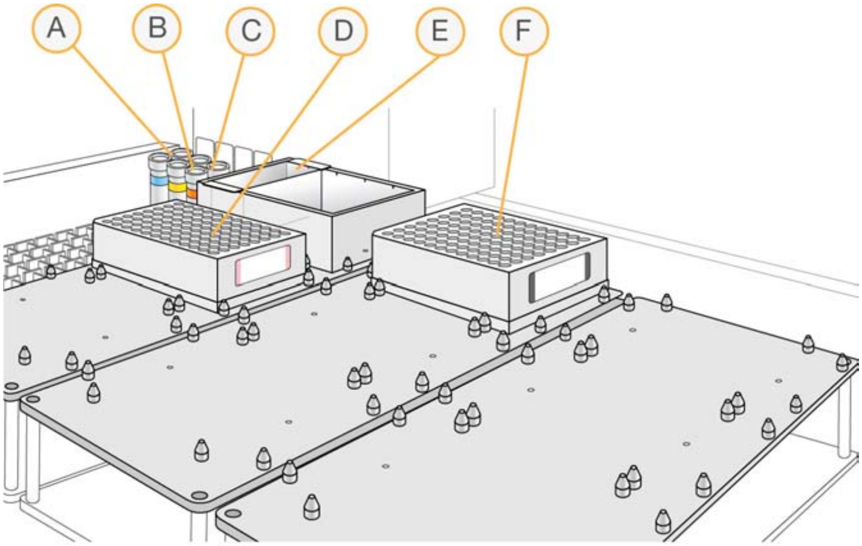

A MA1 Tube
B RPM Tube
C MSM Tube
D MSA4 Plate
E NaOH in Quarter Reservoir
F BCD Plate (TCY or MIDI)

Steps to Make MSA4 Plate

1. If you do not already have a WG#-BCD plate, add BCD into one of the following:
   - MIDI plate: 20 µl to each WG#-BCD plate well
   - TCY plate: 10 µl to each WG#-BCD plate well

   Apply a barcode label to the new WG#-BCD plate.
2. At the robot PC, select **MSA4 Tasks | Make MSA4**.
3. In the Plate Selection dialog box, click on the plate type you wish to use. Roll the mouse pointer over each picture to see a description of the plate.

Figure 85 Selecting the BCD Plate Type

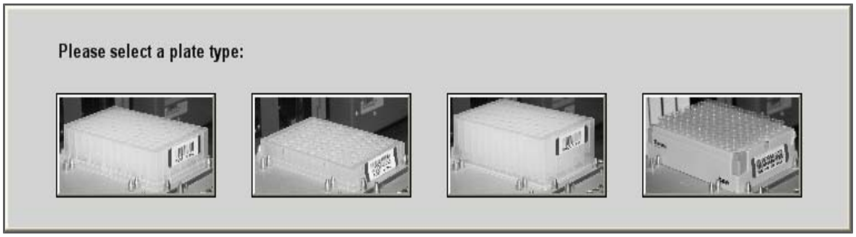

NOTE
Do not mix plate types on the robot bed.

4 Ensure that the **Use Barcodes** check box is cleared. In the Basic Run Parameters pane, enter the **Number of DNA samples** (48 or 96) that are in the plate. This value must match the number of DNAs in the plate and the number of DNAs identified in the DNA manifest.

You can process up to 96 DNA samples per robot run.

The robot PC updates the Required Run Item(s) and the bed map to show the correct position of items on the robot bed. All barcodes must face to the right.

Figure 86 Make MSA4 Screen

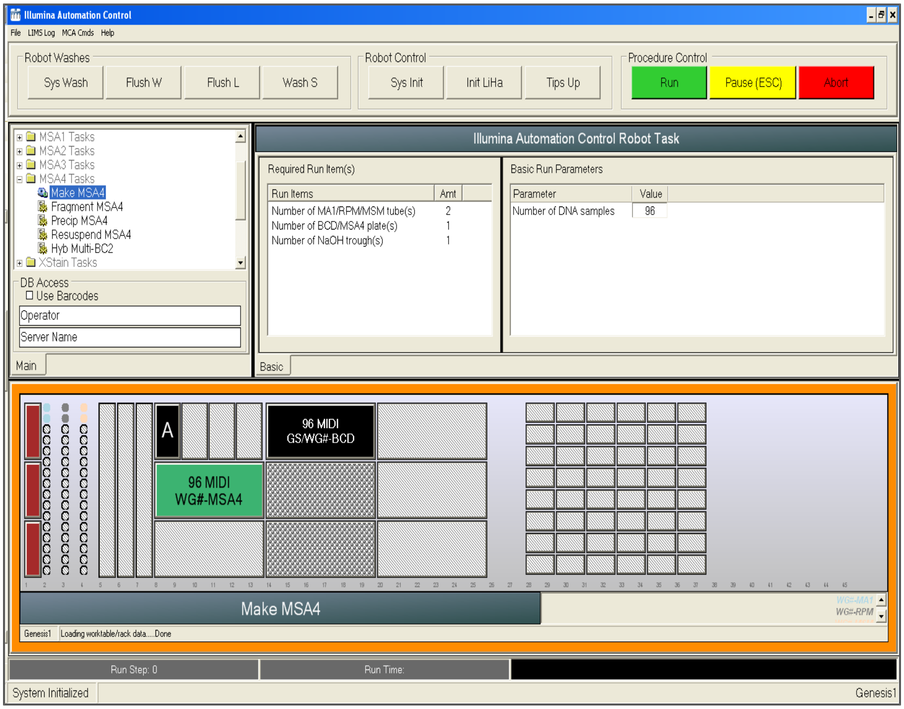

5 Remove caps from MA1, RPM, and MSM tubes, then place the tubes in the robot standoff tube rack according to the bed map.

6 Add 15 ml NaOH to the quarter reservoir, then place the reservoir on the robot bed according to the bed map.

7 Place the WG#-BCD and MSA4 plates on the robot bed according to the bed map.

8 In the Lab Tracking Forms, record the plate positions on the robot bed.

9 Make sure that all items are placed properly on the robot bed, that all caps and seals have been removed, and that all the barcodes face to the right.

10 At the robot PC, click **Run**.

11 After the robot adds the 0.1N NaOH to the DNA in the MSA4 plate, follow the instructions at the prompt.

12 Seal the plate with a cap mat.

13 Vortex the sealed MSA4 plate at 1600 rpm for 1 minute.

14 Centrifuge to 280 xg for 1 minute at 22° C.

15 Remove the cap mat.

NOTE
When you remove a cap mat, set it aside, upside down, in a safe location for use later in the protocol. When you place the cap mat back on the plate, be sure to match it to its original plate and orient it correctly.

16 Place the MSA4 plate back on the robot bed in its original position, and then click **OK**.
The Wait for reaction time message appears. The wait time for this reaction is 10 minutes.
The robot PC sounds an alert and displays a message when the process is complete. Click **OK** in the message box. Remove the MSA4 plate from the robot bed and seal with the 96-well cap mat.

17 Invert the sealed MSA4 plate at least 10 times to mix contents.

18 Centrifuge to 280 xg for 1 minute.

19 Discard unused reagents in accordance with facility standards.

20 Proceed immediately to the next step.

Incubate MSA4 Plate

This process incubates the MSA4 plate for 20–24 hours at 37° C in the Illumina Hybridization Oven. It generates a sufficient quantity of each individual DNA sample to be used twice in the Illumina Infinium HD Methylation Assay.

Figure 87 Incubating DNA to Amplify

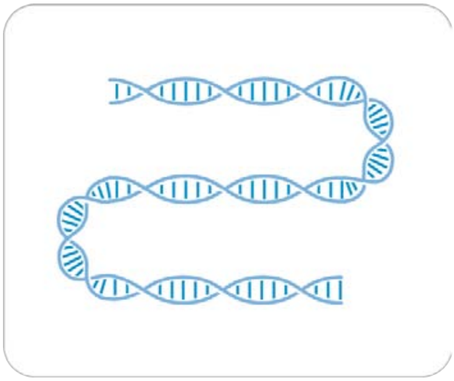

Estimated Incubation Time

20–24 hours

Steps to Incubate MSA4 Plate

1 Incubate MSA4 plate in the Illumina Hybridization Oven for at least 20 but no more than 24 hours at 37° C.

2 On the lab tracking form, record the start and stop times.

NOTE
To record information about your assay such as operator information, start and stop times, and barcodes, use the lab tracking form provided at http://www.illumina.com/documentation. This form can be filled out and saved online, or printed and filled in by hand.

Fragment MSA4 Plate

This process enzymatically fragments the amplified DNA samples. An end-point fragmentation is used to prevent over-fragmentation.

Figure 88 Fragmenting DNA

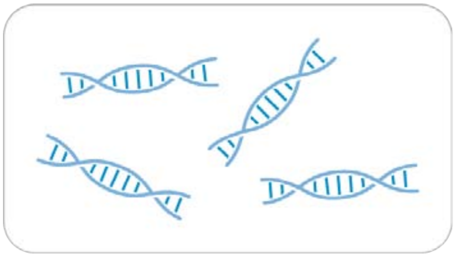

Estimated Time

Robot time:

- 5 minutes for 48 samples
- 10 minutes for 96 samples

Incubation time: 1 hour

Consumables

Item	Quantity	Storage	Supplied By
FMS	2 tubes<br>(per 96 samples)	-15° to -25° C	Illumina

NOTE
Thaw all reagents completely at room temperature and allow to equilibrate. Once thawed, gently invert each tube several times to thoroughly mix the reagent. Pulse centrifuge each tube to 280 xg to eliminate bubbles and collect reagent at the bottom of the tube.

Preparation

- Preheat the heat block with the MIDI plate insert to 37° C.
- On the Lab Tracking Forms, record:
  - Date/Time
  - Operator
  - Robot
  - FMS tube barcode(s)

NOTE
To record information about your assay such as operator information, start and stop times, and barcodes, use the lab tracking form provided at http://www.illumina.com/documentation. This form can be filled out and saved online, or printed and filled in by hand.

Prepare Robot

For instructions on preparing the robot for use in a protocol see the *Infinium Assay Lab Setup and Procedures Guide*.

CAUTION
Do not run any other programs or applications while using the Tecan robot. Your computer and the robot may lock up and stop a run.

Refer to the figure shown below throughout this protocol.

Figure 89 Tecan Eight-Tip Robot (Fragment MSA4 Setup) with FMS tubes and MSA4 Plate

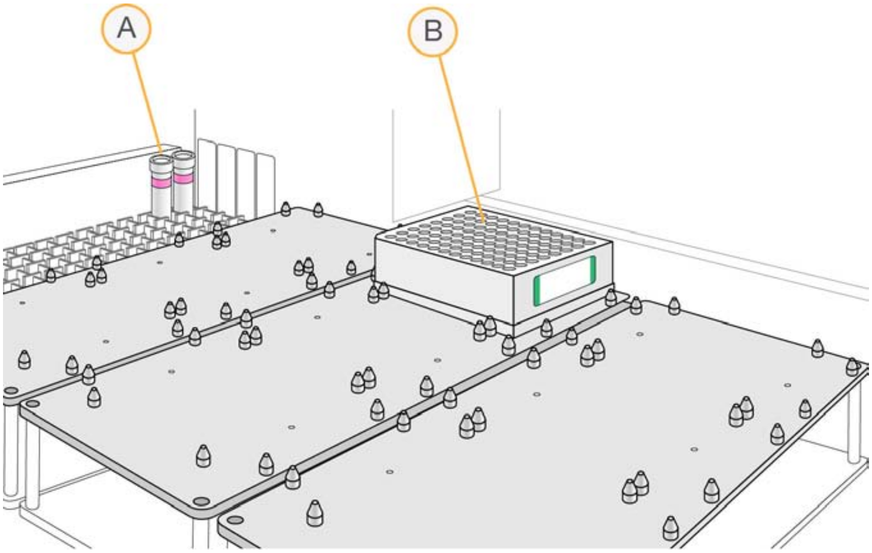

Steps to Fragment MSA4 Plate

1 Centrifuge the MSA4 plate to 50 xg for 1 minute.

2 Remove the cap mat.

3 At the robot PC, select **MSA4 Tasks | Fragment MSA4**.

4 In the Basic Run Parameters pane, change the value for **Number of DNA samples** to reflect the number of samples being processed.
The robot PC updates the Required Run Item(s) and the bed map to show the correct position of items on the robot bed. All barcodes must face to the right.

Figure 90 Fragment MSA4 Screen

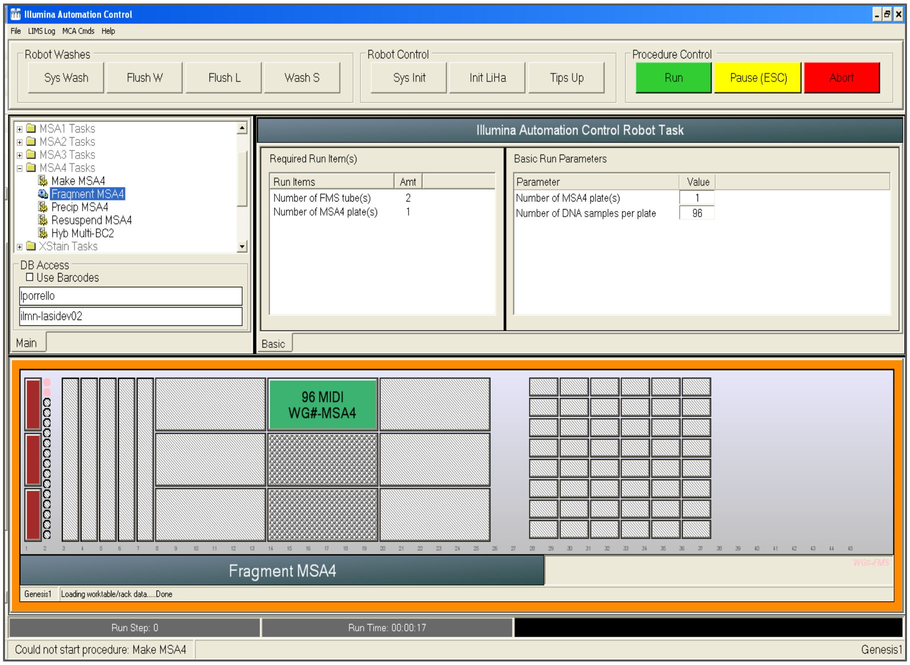

5 Place the MSA4 plate on the robot bed according to the bed map. Remove the plate seal.

6 Place FMS tubes in the robot tube rack according to the bed map. Remove the cap.

7 In the Lab Tracking Forms, record the plate positions on the robot bed.

8 Do one of the following:

- Proceed to *Precipitate MSA4 Plate* on page 150. Leave plate in 37° C heat block until you have completed the preparatory steps.
- Store the sealed MSA4 plate at -15° to -25° C if you do not plan to proceed to the next step immediately

Start Robot

1 At the robot PC:

   a Click **Run** to start the process.
   b Ensure that there are no problems.

   The robot PC sounds an alert and displays a message when the process is done.

2. When the robot finishes, click **OK** in the message box. Remove the MSA4 plate from the robot bed and seal it with a cap mat.
3. Vortex at 1600 rpm for 1 minute.
4. Centrifuge to 50 xg for 1 minute at 22° C.
5. Place the sealed plate on the 37° C heat block for 1 hour.
6. On the Lab Tracking Forms, record the start and stop times.
7. Discard unused reagents in accordance with facility standards.
8. Do one of the following:
   - Proceed to *Precipitate MSA4 Plate* on page 150. Leave plate in 37° C heat block until you have completed the preparatory steps.
   - Store the sealed MSA4 plate at -15° to -25° C if you do not plan to proceed to the next step immediately.

SAFE STOPPING POINT
This is a good stopping point in the process.

Precipitate MSA4 Plate

PM1 and 2-propanol are added to the MSA4 plate to precipitate the DNA samples.

Figure 91 Precipitating DNA

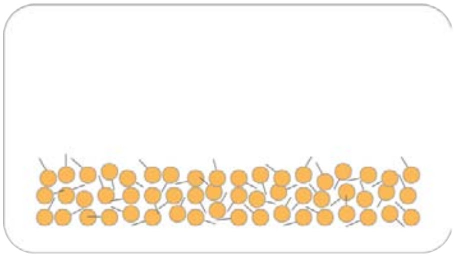

Estimated Time

Robot time:

- 10 minutes for 48 samples
- 15 minutes for 96 samples

Incubation and dry time: 2 hours

Consumables

Item	Quantity	Storage	Supplied By
PM1	2 tubes (per 96 samples)	-15° to -25° C	Illumina
100% 2-propanol	40 ml (per 96 samples)	Room temperature	User

NOTE
Thaw all reagents completely at room temperature and allow to equilibrate. Once thawed, gently invert each tube several times to thoroughly mix the reagent. Pulse centrifuge each tube to 280 xg to eliminate bubbles and collect reagent at the bottom of the tube.

Preparation

- Preheat the heat sealer. Allow 20 minutes.
- MSA4 plate:
  - If you froze the MSA4 plate after fragmentation, thaw it to room temperature.
  - If you continued immediately from Fragment MSA4, leave the plate in the 37° C heat block until setup is complete.
- On the Lab Tracking Forms, record:
  - Date/Time
  - Operator
  - Robot
  - PM1 tube barcode(s)
  - 2-propanol lot number and date opened

NOTE
To record information about your assay such as operator information, start and stop times, and barcodes, use the lab tracking form provided at http://www.illumina.com/documentation. This form can be filled out and saved online, or printed and filled in by hand.

Prepare Robot

For instructions on preparing the robot for use in a protocol see the *Infinium Assay Lab Setup and Procedures Guide*.

Refer to the figure shown below throughout this protocol. Note that barcodes face to the right.

Figure 92 Tecan Eight-Tip Robot (Precip MSA4 Setup)

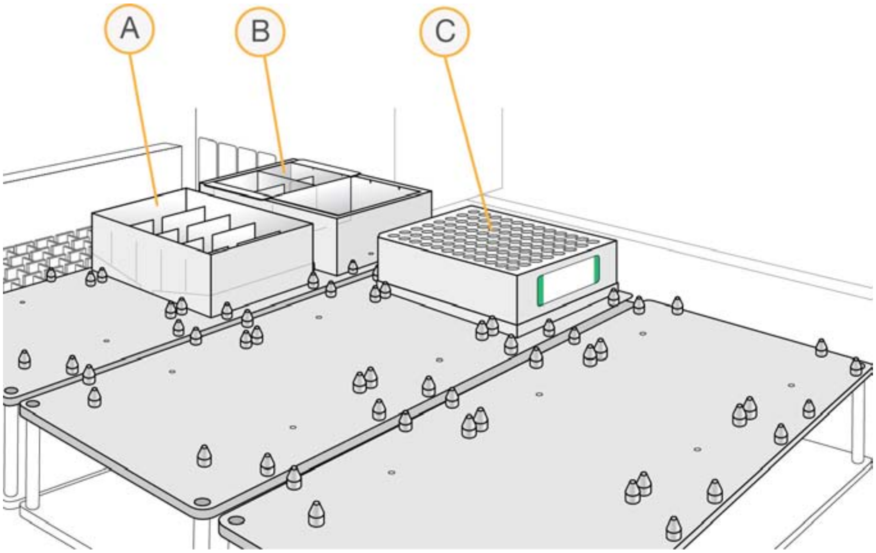

A 2-propanol in full Reservoir
B PM1 in Half Reservoir
C MSA4 Plate

Steps to Precipitate MSA4 Plate

1. Centrifuge the sealed MSA4 plate to 50 xg for 1 minute at 22° C.
2. At the robot PC, select **MSA4 Tasks | Precip MSA4**.
3. In the Basic Run Parameters pane, change the value for **Number of DNA samples** to reflect the number of DNAs being processed.
   The robot PC updates the Required Run Item(s) and the bed map to show the correct position of items on the robot bed. All barcodes must face to the right.

Figure 93 Precip MSA4 Screen

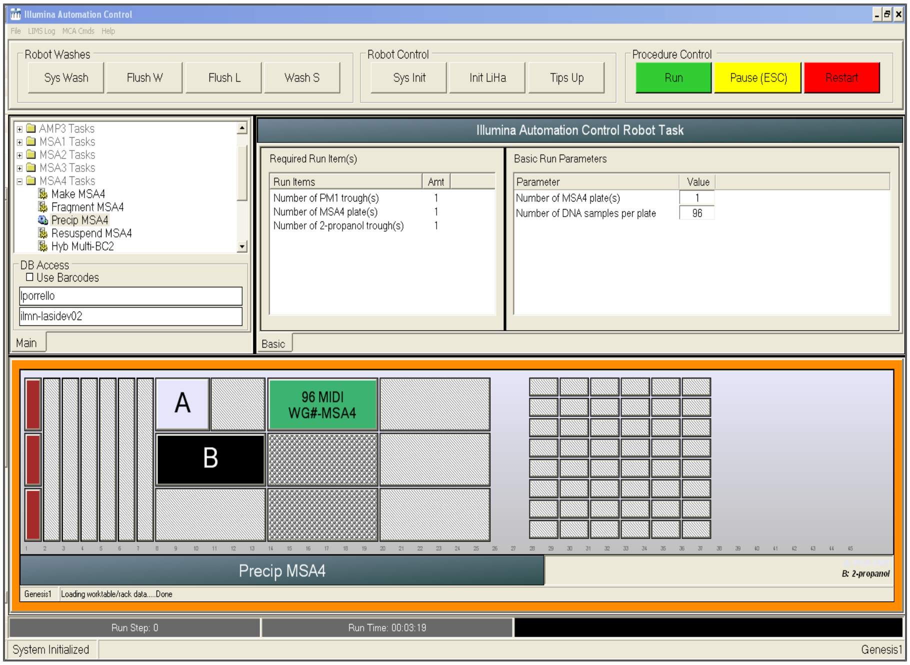

4 Remove the cap mat and place the MSA4 plate on the robot bed according to the bed map.

5 Place a half reservoir in the reservoir frame, according to the robot bed map, and add PM1 as follows:
- For 48 samples, dispense 1 tube PM1
- For 96 samples, dispense 2 tubes PM1

6 Place a full reservoir in the reservoir frame, according to the robot bed map, and add 2-propanol as follows:
- For 48 samples, dispense 20 ml 2-propanol
- For 96 samples, dispense 40 ml 2-propanol

7 In the Lab Tracking Forms, record the plate positions on the robot bed.

8 Make sure that all items are placed properly on the robot bed, that all caps and seals have been removed, and that all the barcodes face to the right.

Start Robot

1. At the robot PC:
   a. Click **Run** to start the process.
   b. Ensure that there are no problems.
2. When prompted, remove the MSA4 plate from the robot bed. Do not click **OK** in the message box yet.
3. Seal the MSA4 plate with the same cap mat removed earlier.
4. Vortex the sealed plate at 1600 rpm for 1 minute.
5. Incubate at 37° C for 5 minutes.
6. Centrifuge 50 xg at room temperature for 1 minute.

> NOTE
> Set centrifuge to 4° C in preparation for the next centrifuge step.

7. Remove the cap mat and place the MSA4 plate back on the robot bed according to the bed map (*Steps to Precipitate MSA4 Plate* on page 152).
8. Click **OK** in the Please Remove MSA4 Plate message box. The robot PC sounds an alert and displays a message when the process is complete.
9. Click **OK** in the message box. Remove the MSA4 plate from the robot bed and carefully seal with a ***new, dry*** cap mat, taking care not to shake the plate in any way until the cap mat is fully seated.
10. Invert the plate at least 10 times to mix contents thoroughly.
11. Incubate at 4° C for 30 minutes.
12. Place the sealed MSA4 plate in the centrifuge opposite another plate of equal weight.

Figure 94 Sealed MSA4 Plate and Plate of Equal Balance in Centrifuge

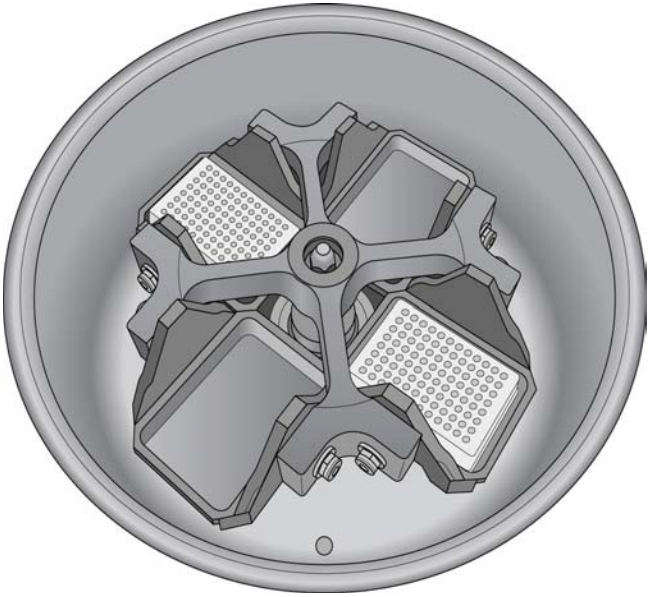

13 Centrifuge to 3000 xg at 4° C for 20 minutes.

14 Immediately remove MSA4 plate from centrifuge. Remove the cap mat and discard it.
Perform the next step immediately to avoid dislodging the blue pellet. If any delay occurs, repeat 11 through 12 before proceeding.

15 Over an absorbent pad appropriate for 2-propanol disposal, decant supernatant by quickly inverting the MSA4 plate and smacking it down.

16 Tap the plate firmly on the pad several times over a period of 1 minute or until all wells are completely devoid of liquid.

CAUTION
Keep the plate inverted. To ensure optimal performance, do not allow supernatant in wells to pour into other wells.

17 Leave the uncovered plate inverted on the tube rack for 1 hour at room temperature to air dry the pellet.
At this point, blue pellets should be present at the bottoms of the wells.

Figure 95 Uncovered MSA4 Plate Inverted for Air Drying

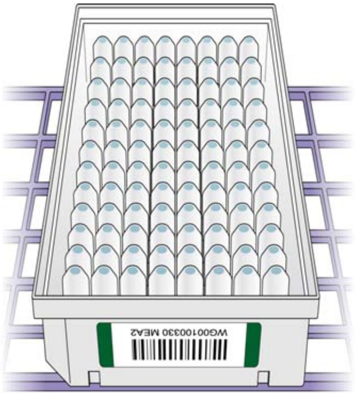

CAUTION
Do not over-dry the pellet. Pellets that are over-dried will be difficult to resuspend. Poorly resuspended samples will lead to poor data quality.

18 On the Lab Tracking Forms, record the start and stop times.

19 Discard unused reagents in accordance with facility standards.

20 Do one of the following:
- Proceed to *Resuspend MSA4 Plate* on page 157.
- If you do not plan to proceed to the next step immediately, seal the MSA4 plate with a new cap mat and store at -15° to -25° C.

SAFE STOPPING POINT
This is a good stopping point in the process.

Resuspend MSA4 Plate

RA1 is added to the MSA4 plate to resuspend the precipitated DNA samples.

Figure 96 Resuspending DNA

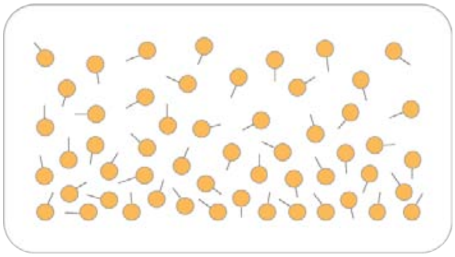

Estimated Time

Robot time:

- 10 minutes for 48 samples
- 15 minutes for 96 samples

Incubation time: 1 hour

Consumables

Item	Quantity (Per 96 samples)	Storage	Supplied By
RA1	Bottle (9 ml)	-15° to -25° C	Illumina

NOTE
Pour out only the recommended volume of RA1 needed for the suggested number of samples listed in the consumables table. Additional RA1 is used later in the XStain HD BeadChip step

WARNING
This protocol involves the use of an aliphatic amide that is a probable reproductive toxin. Personal injury can occur through inhalation,

ingestion, skin contact, and eye contact. For more information, consult the material data safety sheet for this assay at http://www.illumina.com/msds. Dispose of containers and any unused contents in accordance with the governmental safety standards for your region.

Preparation

- RA1 is shipped frozen. Gradually warm the reagent to room temperature, preferably in a 20°–25° C water bath. Gently mix to dissolve any crystals that may be present.
- If you stored the MSA4 plate at -15° to -25° C, thaw it to room temperature. Remove the cap mat and discard it.
- Preheat the Illumina Hybridization Oven to 48° C.
- Preheat the heat sealer. Allow 20 minutes.
- On the Lab Tracking Forms, record:
  - Date/Time
  - Operator
  - Robot
  - RA1 bottle barcode(s)

NOTE
To record information about your assay such as operator information, start and stop times, and barcodes, use the lab tracking form provided at http://www.illumina.com/documentation. This form can be filled out and saved online, or printed and filled in by hand.

Prepare Robot

For instructions on preparing the robot for use in a protocol and ensuring that the Chamber Rack is properly installed on the post-amplification robot bed, see the *Infinium Assay Lab Setup and Procedures Guide*.

Refer to the figure shown below throughout this protocol. Note that all of the barcodes face to the right.

Figure 97 Tecan Eight-Tip Robot (Resuspend MSA4 Setup)

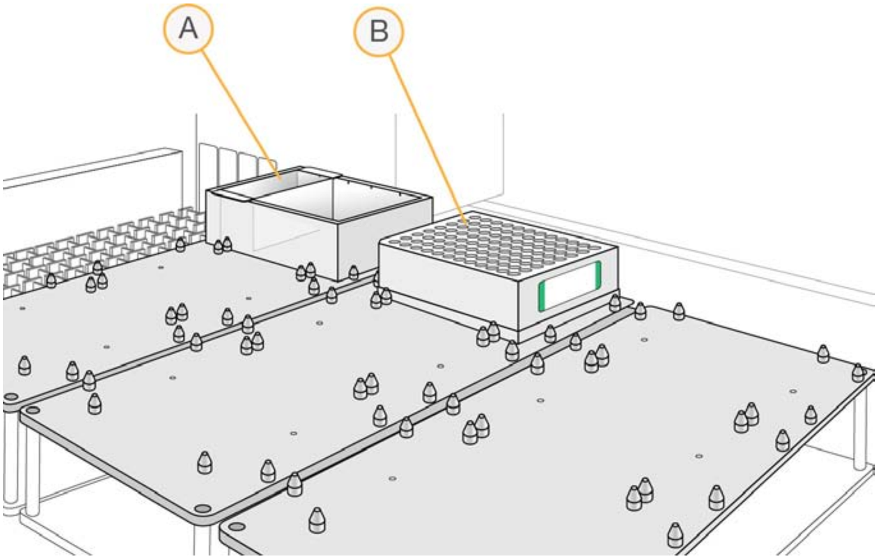

A RA1 in Quarter Reservoir
B MSA4 Plate

Steps to Resuspend MSA4 Plate

1. At the robot PC, select **MSA4 Tasks | Resuspend MSA4**.
2. In the Basic Run Parameters pane, change the value for **Number of DNA samples** to reflect the number of DNAs being processed.
   The robot PC updates the Required Run Item(s) and the bed map to show the correct position of items on the robot bed. All barcodes must face to the right.

Figure 98 Resuspend MSA4 Screen

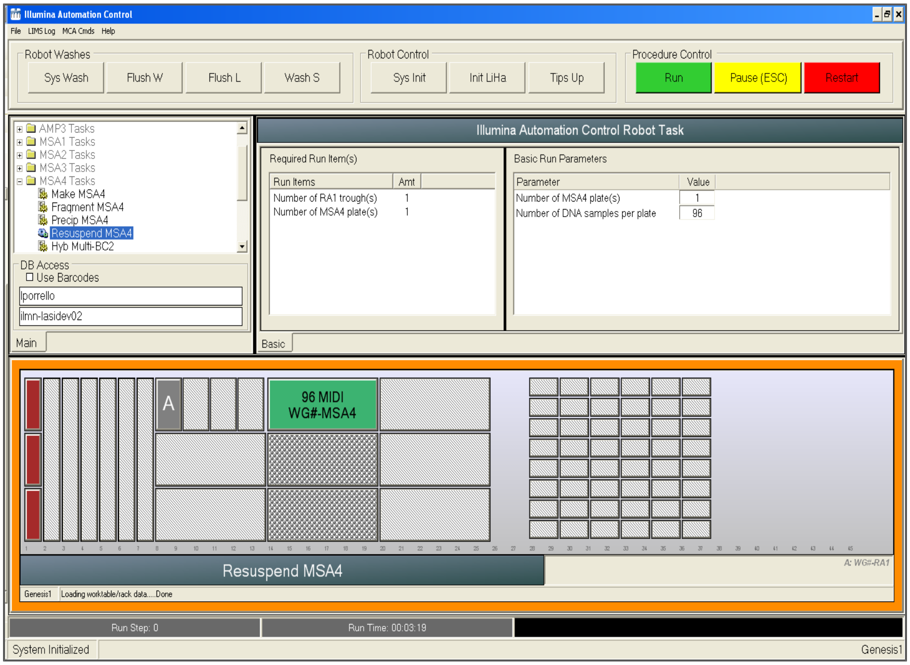

3 Place the MSA4 plate on the robot bed according to the bed map.

4 Place a quarter reservoir in the reservoir frame, according to the robot bed map, and add RA1 as follows:

- 4.5 ml for 48 samples
- 9 ml for 96 samples

5 In the Lab Tracking Forms, record the plate positions on the robot bed and RA1 barcodes on the Lab Tracking Form.

6 Make sure that all items are placed properly on the robot bed, that all caps and seals have been removed, and that all the barcodes face to the right.

Start Robot

1 At the robot PC:

a Click **Run** to start the process.

The robot PC sounds an alert and displays a message when the process is complete.

2 Click **OK** in the message box. Remove the MSA4 plate from the robot bed.

3 Apply a foil seal to the MSA4 plate by firmly holding the heat sealer block down for 5 seconds.

4 Place the sealed plate in the Illumina Hybridization Oven and incubate for 1 hour at 48° C.

5 In the Lab Tracking Forms, record the start and stop times.

6 Vortex the plate at 1800 rpm for 1 minute.

7 Pulse centrifuge to 280 xg.

8 Discard unused reagents in accordance with facility standards.

NOTE

If you stored the DNA pellets at -15° to -25° C for more than 72 hours, you may need to re-vortex and centrifuge until the pellets are completely resuspended.

SAFE STOPPING POINT

This is a good stopping point in the process.

NOTE

If you do not plan to proceed to the next step immediately, store the sealed MSA4 plate at -15° to -25° C (-80° C if storing for more than 24 hours). Store RA1 at -15° to -25° C.

Hybridize Multi BeadChip

In this process, the fragmented and resuspended DNA samples are dispensed onto the BeadChips. DNA-loaded BeadChips are placed into Hyb Chamber Inserts that are placed inside the Hyb Chambers.

Once the DNA samples are loaded into the Flow-Through Chambers, incubate the chambers for 16–24 hours at 48° C in the Illumina Hybridization Oven. Hybridization occurs during the incubation period. Each sample will be hybridized to an individual section of the BeadChip.

Figure 99 Hybridizing DNA to BeadChip

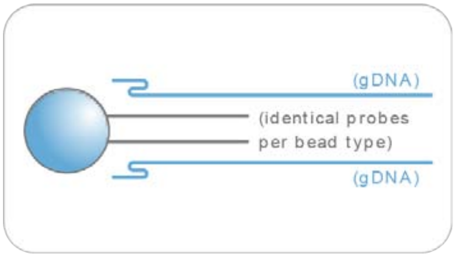

Estimated Time

Robot time:

- 10 minutes for 48 samples
- 15 minutes for 96 samples

Incubation time: 16–24 hours

Consumables

Item	Quantity (per 96 Samples)	Storage	Supplied By
PB2	2 tubes	Room temperature	Illumina
BeadChips	8	4° C	Illumina
Hyb Chambers	2		Illumina
Hyb Chamber gaskets	2		Illumina
Hyb Chamber inserts	8		Illumina
Robot BeadChip Alignment Fixtures	4		Illumina
EtOH	500 ml		User

NOTE
Thaw all reagents completely at room temperature and allow to equilibrate. Once thawed, gently invert each tube several times to thoroughly mix the reagent. Pulse centrifuge each tube to 280 xg to eliminate bubbles and collect reagent at the bottom of the tube.

Preparation

- Preheat the heat block to 95° C.
- Preheat the Illumina Hybridization Oven to 48° C.
- On the Lab Tracking Forms, record:
  - Date/Time
  - Operator
  - Robot
  - PB2 tube barcode

NOTE
To record information about your assay such as operator information,

start and stop times, and barcodes, use the lab tracking form provided at http://www.illumina.com/documentation. This form can be filled out and saved online, or printed and filled in by hand.

NOTE
Perform the Hyb Chamber assembly near the Hyb Oven to minimize the distance you need to move the BeadChip loaded Hyb Chamber. Take care to keep the Hyb Chamber steady and level when lifting and moving. Avoid shaking and keep the Hyb Chamber parallel to the lab bench at all times.

Assemble Hybridization Chambers

1 Place the following items on the bench top:
- BeadChip Hyb Chambers (2)
- Hyb Chamber Gaskets (2)
- Robot BeadChip Alignment Fixtures (4)
- BeadChip Hyb Chamber Inserts (8)

Figure 100 BeadChip Hyb Cartridge Components

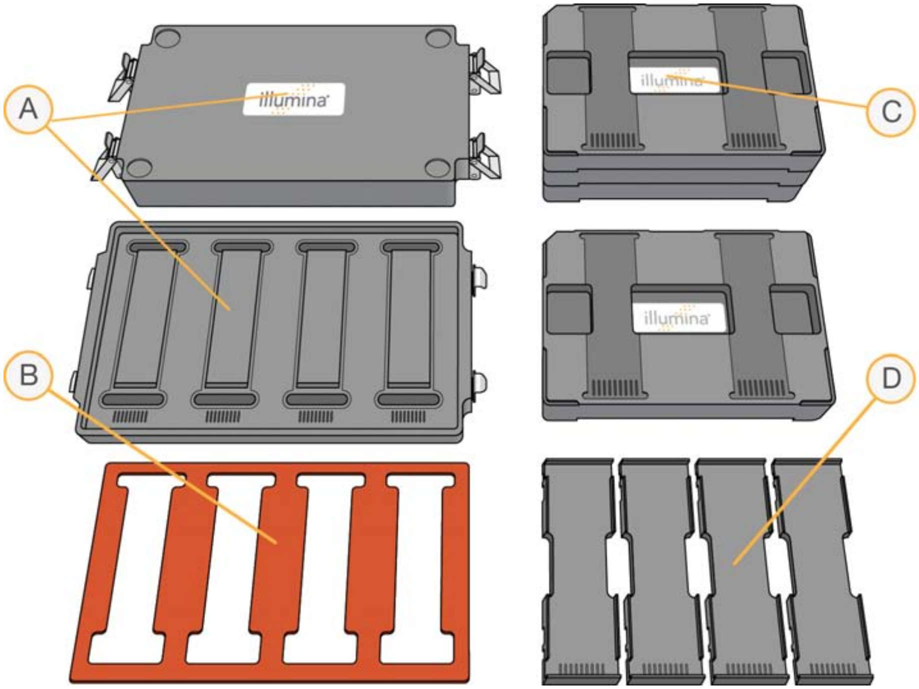

A Hyb Chamber
B Hyb Chamber Gasket
C Robot Bead Chip Alignment Fixture
D Hyb Chamber Insert

NOTE
To ensure optimal results from Hyb Chambers keep the Hyb Chamber lids and bases together. Adopt a labeling convention that keeps each Hyb Chamber base paired with its original lid. Check Hyb Chamber lid-base pairs regularly to ensure that the fit remains secure. Check hinges regularly for any signs of abnormal wear or loose fittings. It is important that the hinges provide adequate clamping strength to ensure an airtight seal between the lid and the base. Record the Hyb Chamber that was used for each BeadChip, so that Hyb Chambers can be investigated and evaluated in the event of sample evaporation or other lab processing anomalies.

2 Place the BeadChip Hyb Chamber gaskets into the BeadChip Hyb Chambers.

a Match the wider edge of the Hyb Chamber gasket to the barcode-ridge side of the Hyb Chamber.

Figure 101 Hyb Chamber and Gasket

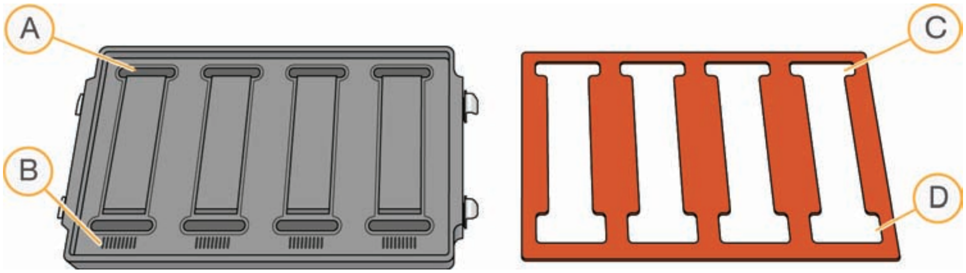

A Reservoirs
B Barcode Ridges
C Narrower Edges
D Wider Edges

b Lay the gasket into the Hyb Chamber, and then press it down all around.

Figure 102 Placing Gasket into Hyb Chamber

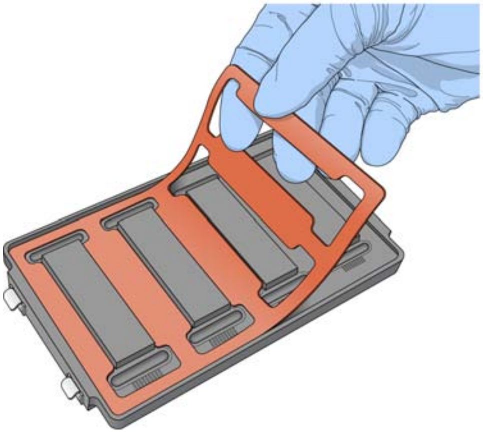

c Make sure the Hyb Chamber gaskets are properly seated.

Figure 103 Hyb Chamber with Gasket in Place

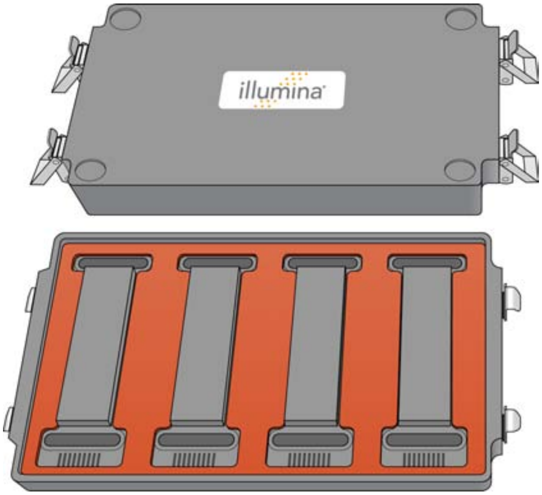

3 Dispense 400 µl PB2 into the eight humidifying buffer reservoirs in the Hyb Chambers.

Figure 104 Dispensing PB2 into Hyb Chamber Reservoirs

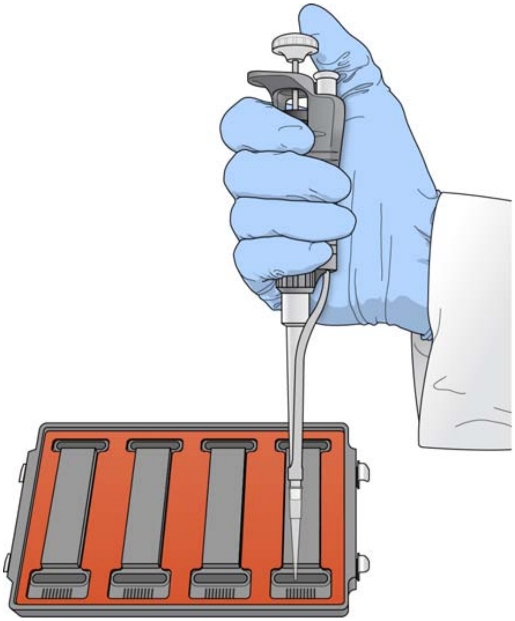

4 Close and lock the BeadChip Hyb Chamber lid.
   a Seat the lid securely on the bottom plate.
   b Snap two clamps shut, kitty-corner, across from each other.
   c Snap the other two clamps.

Figure 105 Sealing the Hyb Chamber

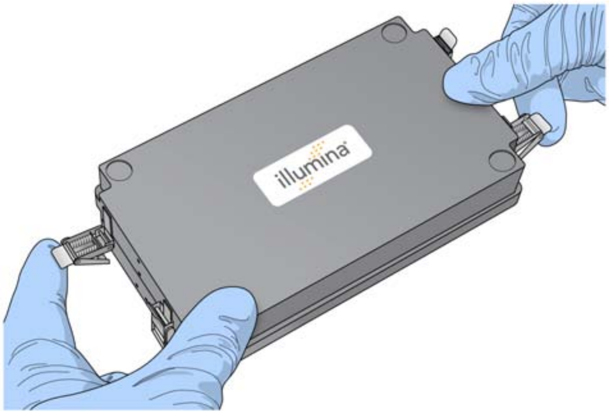

5 Leave the closed Hyb Chambers on the bench at room temperature until the BeadChips are loaded with DNA sample.

Prepare the Robot

For instructions on preparing the robot for use in a protocol and ensuring that the Chamber Rack is properly installed on the post-amplification robot bed, see the *Infinium Assay Lab Setup and Procedures Guide*.

Refer to the figure below throughout this protocol. Note that all of the plate barcodes face to the right.

Figure 106 Placing Alignment Fixtures and MSA4 Plate onto Robot Bed

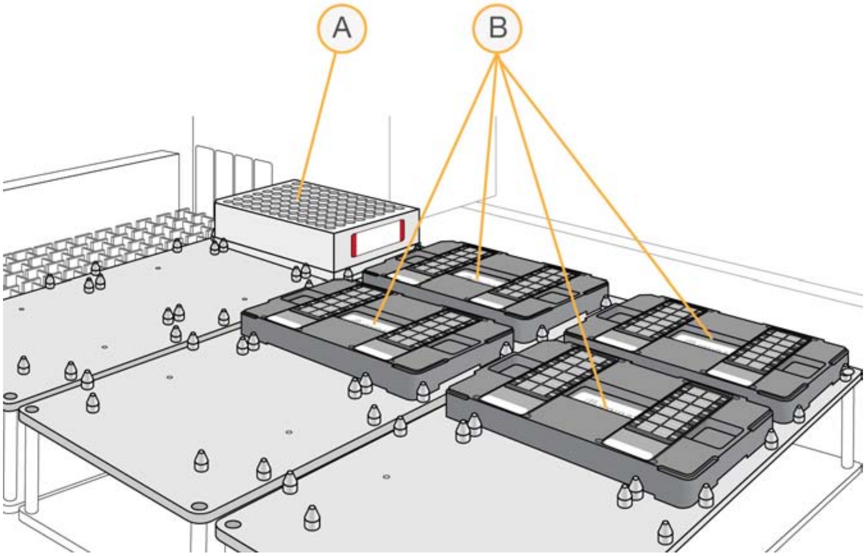

A MSA4 Plate
B Robot BeadChip Alignment Fixtures

Load BeadChips

1. Place the resuspended MSA4 plate on the heat block to denature the samples at 95° C for 20 minutes.
2. After the 20-minutes incubation, remove the MSA4 plate from the heat block and place it on the benchtop at room temperature for 30 minutes, and continue with the following steps.

CAUTION
Do not unpackage BeadChips until you are ready to begin hybridization.

3. Remove all BeadChips from their packages.
4. Place BeadChips into the Robot BeadChip Alignment Fixtures with the barcode end aligned to the ridges on the fixture.

Figure 107

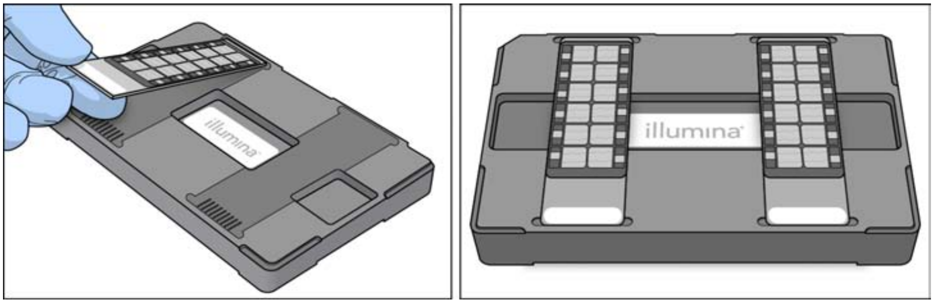

5 Place the appropriate number of BeadChips per sample number (4 BeadChips for 48 samples, 8 BeadChips for 96 samples) into the Robot BeadChip Alignment Fixtures.

6 Stack the Robot BeadChip Alignment Fixtures and carry them to the robot.

Figure 108 Four Stacked Robot BeadChip Alignment Fixtures

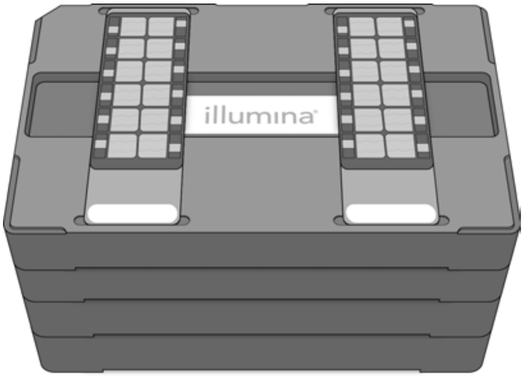

7 At the robot PC, select **MSA4 Tasks | Hyb Multi BeadChip**.

8 Choose the 12 x 1 BeadChip from the Bead Chip Selection dialog box.

9 In the Basic Run Parameters pane, change the value for the **Number of DNA Samples** to reflect the number of DNAs being processed.
The robot PC updates the Required Run Item(s) and the bed map to show the correct position of items on the robot bed. All barcodes must face to the right.

Figure 109 Hyb Multi BeadChip Screen

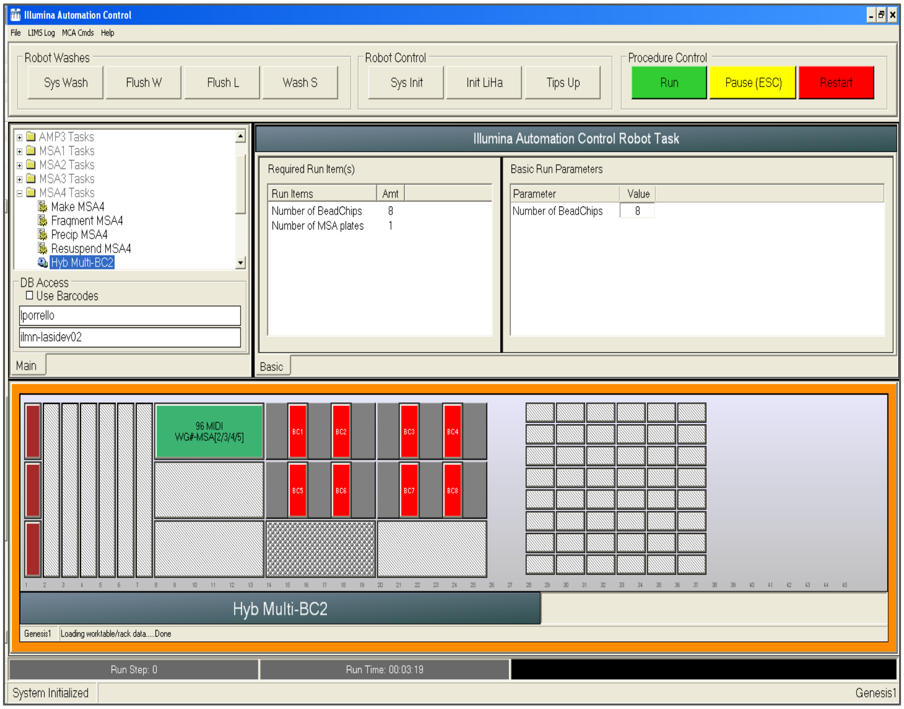

10 Place the Robot BeadChip Alignment Fixtures onto the robot bed according to the bed map.

11 On the Lab Tracking Forms, record the plate position on the robot bed, BeadChip serial numbers, and BeadChip positions on the Lab Tracking Form.

12 Pulse centrifuge the MSA4 plate to 280 xg.

13 Place the MSA4 plate onto the robot bed according to the bed map. Remove the foil seal.

Start Robot

1 At the robot PC:

a Click **Run** to start the process.

The robot dispenses the sample onto the BeadChips, following the layout shown in the Lab Tracking Forms.
The robot PC sounds an alert and displays a message when the process is complete.

2 Click **OK** in the message box.

3 Carefully remove the Robot BeadChip Alignment Fixtures from the robot bed and visually inspect all sections of the BeadChips. Ensure DNA sample covers all of the sections of each bead stripe. Record any sections that are not completely covered.

4 After the MSA4 hyb plate is done, apply a foil seal to the MSA4 plate by firmly holding the heat sealer block down for 5 seconds.

5 Store MSA4 plate at -15° to -25° C. Store at -80° C if you do not plan to use it again within 24 hours.

Set up Multi BeadChip for Hybridization

1 Ensure the Illumina Hybridization Oven is set to 48° C.

CAUTION
Hold the BeadChip by the ends with your thumb and forefinger (thumb at the barcode end). Do not hold the BeadChip by the sides near the sample inlets. Avoid contacting the beadstripe area and sample inlets.

WARNING
Chambers should be at room temperature when you load the BeadChips. They should not be preconditioned in the Illumina Hybridization Oven prior to loading the BeadChips. Heating the PB2 and then opening the Hyb Chamber to add BeadChips causes some of the PB2° to evaporate, leading to a change in the osmolality of PB2 and an imbalance in the vapor pressure between PB2 and RA1 (sample hyb buffer).

2 Carefully remove each BeadChip from the Robot BeadChip Alignment Fixtures when the robot finishes.

3 Calibrate the Illumina Hybridization Oven with the Full-Scale Plus digital thermometer supplied with your system.

4 Carefully place each BeadChip in a Hyb Chamber insert, orienting the barcode end so that it matches the barcode symbol on the insert.

Figure 110 Matching the Barcode End to the Insert Fixture

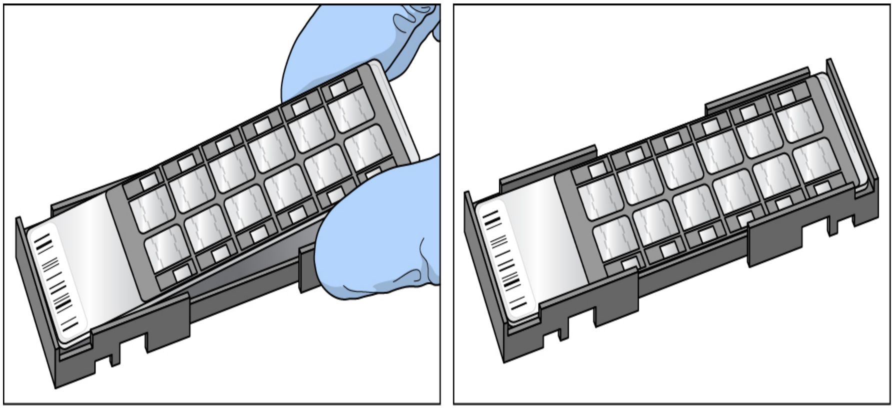

5 Load the Hyb Chamber inserts containing loaded BeadChips inside the Illumina Hyb Chamber. Position the barcode over the ridges indicated on the Hyb Chamber.

CAUTION
For optimal performance, take care to keep the Hyb Chamber inserts containing BeadChips steady and level when lifting or moving. Avoid shaking and keep parallel to the lab bench at all times. Do not hold by the sides near the sample inlets.

Figure 111 Placing Hyb Chamber Inserts into Hyb Chamber

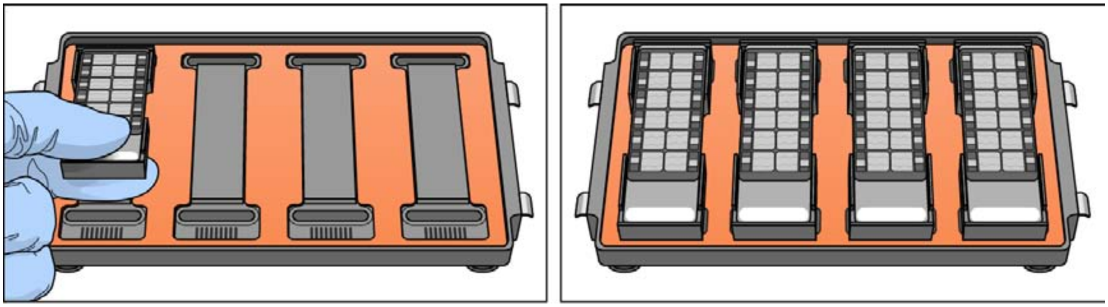

6 Ensure Hyb Chamber inserts are seated properly.

7 Position the lid onto the Hyb Chamber by applying the backside of the lid first and then slowly bringing down the front end to avoid dislodging the Hyb Chamber inserts.

Figure 112 Seating Lid onto Hyb Chamber

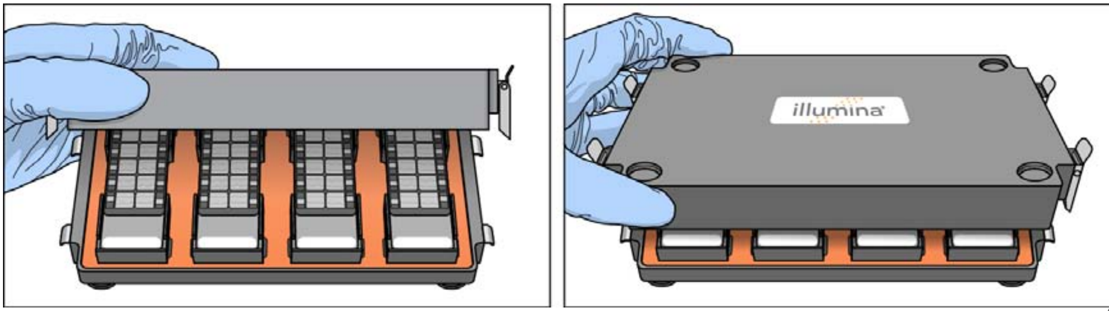

8 Secure the lid by closing down the clamps on both sides of the Hyb Chamber. It is best to close them in a kitty-corner fashion, closing first the top left clamp, then the bottom right, then the top right followed by the bottom left.

NOTE
For optimal performance, take care to keep the Hyb Chamber steady and level when lifting or moving. Avoid shaking the Hyb Chamber, and keep the Hyb Chamber parallel to the lab bench while you transfer it to the Illumina Hybridization Oven.

9 Place the Hyb Chamber in the 48° C Illumina Hybridization Oven so that the clamps of the Hyb Chamber face the left and right side of the oven. The Illumina logo on top of the Hyb Chamber should be facing you.

Figure 113 Hyb Chamber Correctly Placed in Hyb Oven

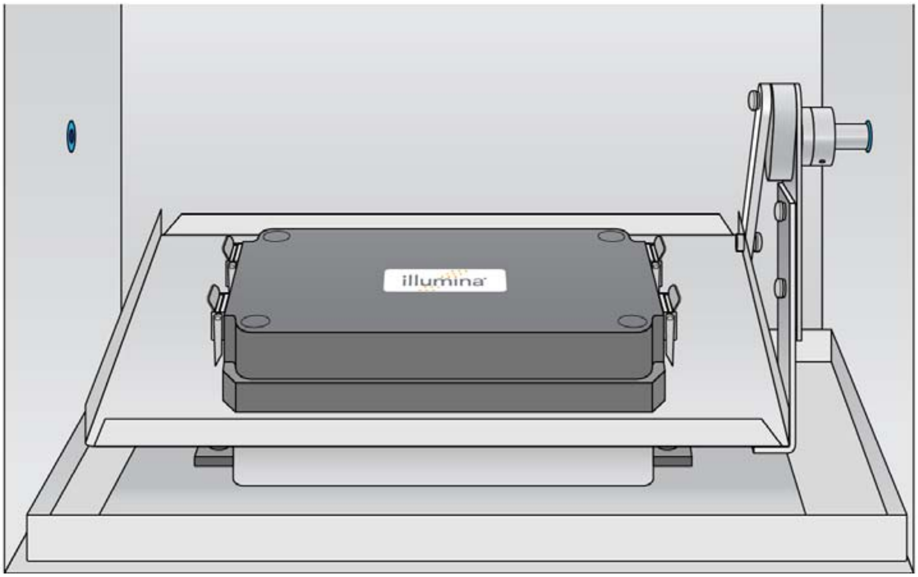

10 If you are loading multiple Hyb Chambers, you may stack them on top of each other. You can stack up to 4 Hyb Chambers.

NOTE
If you are stacking multiple Hyb Chambers in the Illumina Hybridization Oven, make sure the feet of the top Hyb Chamber fit into the matching indents on top of the bottom Hyb Chamber. This will hold the Hyb Chambers in place while they are rocking.

Figure 114 Two Hyb Chambers Correctly Placed in Hyb Oven

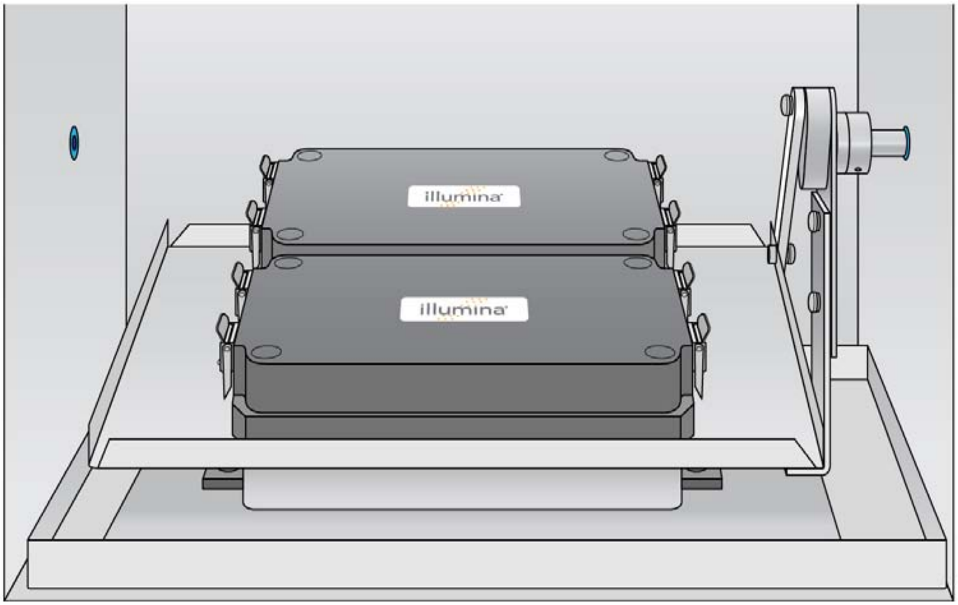

Figure 115 Incorrectly Placed Hyb Chamber

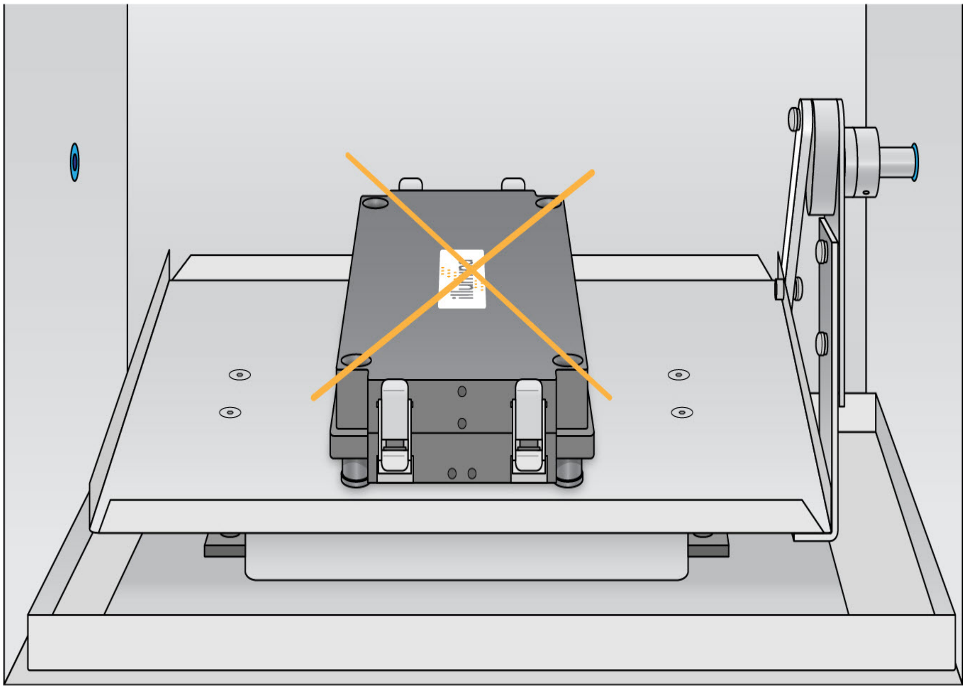

11 Start the rocker, setting the speed to 5 (optional).

12 Incubate at 48° C for at least 16 hours but no more than 24 hours.

13 On the Lab Tracking Forms, record the start and stop times.

Resuspend XC4 Reagent for XStain BeadChip

Keep the XC4 in the bottle in which it was shipped until you are ready to use it. In preparation for the XStain protocol, follow these steps to resuspend the XC4 reagent:

1 Add 330 ml 100% EtOH to the XC4 bottle. The final volume will be 350 ml. Each XC4 bottle (350 ml) has enough solution to process up to 24 BeadChips.

2 Shake vigorously for 15 seconds.

3 Leave the bottle upright on the lab bench overnight.

4 Shake again to ensure that the pellet is completely resuspended. If any coating is visible, vortex at 1625 rpm until it is in complete suspension. Once resuspended with 330 ml 100% EtOH, bring XC4 to room temperature before use.

NOTE
If the XC4 was not left to resuspend overnight, you can still proceed with the assay. Add the EtOH and put the XC4 on its side on a rocker to resuspend. Leave it there until the BeadChips are ready for coating.

Wash BeadChip

In this process, the BeadChips are prepared for the XStain BeadChip process. Coverseals are removed from BeadChips and the BeadChips are washed in PB1 reagent. BeadChips are then assembled into Flow-Through Chambers under the PB1 buffer.

Figure 116 Washing BeadChip

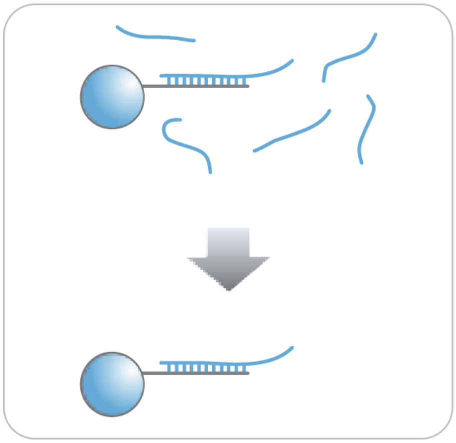

Estimated Time

- 20 minutes for 4 BeadChips
- 30 minutes for 8 BeadChips

Consumables

Item	Quantity (per 4 BeadChips)	Storage	Supplied By
PB1	550 ml (up to 8 BeadChips)	Room temperature	Illumina
Multi-Sample BeadChip Alignment Fixture	1 (per 8 BeadChips)		Illumina
Te-Flow -Through Chambers (with Black Frames, Spacers, Glass Back Plates, and Clamps)	4		Illumina
Wash Dish	2 (up to 8 BeadChips)		Illumina
Wash Rack	1 (up to 8 BeadChips)		Illumina

NOTE
Pour out only the recommended reagent volume needed for the suggested number of samples listed in the Consumables table of each section. Some reagents are used later in the protocol.

WARNING
This protocol involves the use of an aliphatic amide that is a probable reproductive toxin. Personal injury can occur through inhalation, ingestion, skin contact, and eye contact. For more information, consult the material data safety sheet for this assay at http://www.illumina.com/msds. Dispose of containers and any unused contents in accordance with the governmental safety standards for your region.

NOTE
Thaw all reagents completely at room temperature and allow to equilibrate. Once thawed, gently invert each tube several times to thoroughly mix the reagent. Pulse centrifuge each tube to 280 xg to eliminate bubbles and collect reagent at the bottom of the tube.

Preparation

- Remove each Hyb Chamber from the Illumina Hybridization Oven. Let cool on the benchtop for 25 minutes prior to opening.
- Have ready on the lab bench:
  - Two wash dishes:
    - Containing 200 ml PB1, and labeled as such
  - Multi-Sample BeadChip Alignment Fixture
    - Using a graduated cylinder, fill with 150 ml PB1
  - Te-Flow -Through Chamber components:
    - Black frames
    - Spacers (separated for ease of handling)
    - Clean glass back plates (Clean as directed in the SOP)
    - Clamps
- On the Lab Tracking Forms, record:
  - Date/Time
  - Operator
  - Robot
  - PB1 bottle barcode

NOTE
You can download and print copies of the lab tracking worksheet from http://www.illumina.com/documentation.

Steps to Wash BeadChip

1. Attach the wire handle to the rack and submerge the wash rack in the wash dish containing 200 ml PB1.
2. Remove the Hyb Chamber(s) from the Illumina Hybridization Oven.
3. Wait 30 minutes for the Hyb Chambers to cool down, and then remove BeadChips from the Hyb Chamber inserts one at a time.

Figure 117

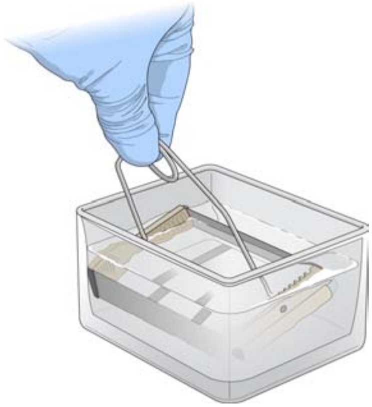

4 Remove the coverseal from the BeadChip as follows:

a Using powder-free gloved hands, hold the BeadChip in one hand with your thumb and forefinger on the long edges of the BeadChip. The BeadChip may also be held with the thumb and forefinger on the short edges of the BeadChip. In either case avoid contact with the sample inlets. The barcode should be facing up and be closest to you, and the top side of the BeadChip should be angled slightly away from you.

b Remove the entire seal in a single, slow, consistent motion by pulling it off in a diagonal direction away from yourself. Start with a corner on the barcode end and pull with a continuous upward motion away from you and towards the opposite corner on the top side of the BeadChip. Do not stop and start the pulling action. Do not touch the exposed active areas.

NOTE
To ensure no solution splatters on you, be sure to pull the coverseal away from yourself. Illumina recommends removing the coverseal over an absorbent cloth or paper towels, preferably in a hood.

c Discard the coverseal.

Figure 118 Removing the Coverseal

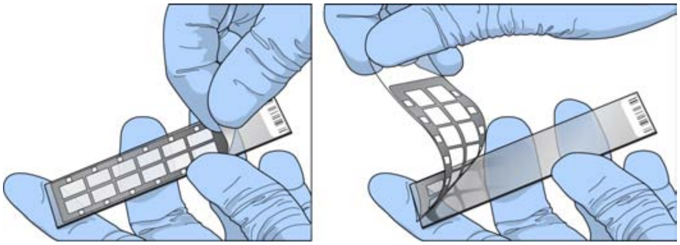

CAUTION
Do not touch the arrays!

5 Immediately and carefully slide the BeadChips into the wash rack, making sure that they are completely submerged in the PB1.

Figure 119 Submerging BeadChips in Wash Dish Containing PB1

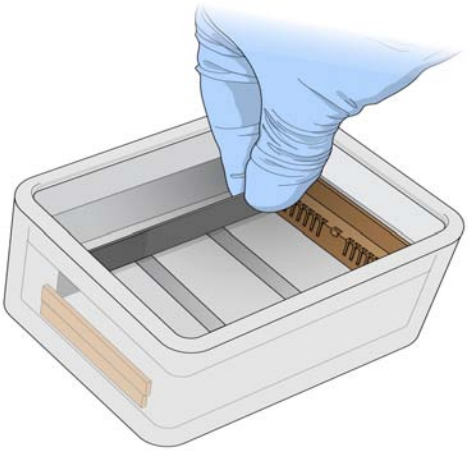

6 Repeat steps 4 through 5 for each BeadChip to be processed. The wash rack holds up to 8 BeadChips.

7 Once all BeadChips are in the wash rack, move the wash rack up and down for 1 minute, breaking the surface of the PB1 with gentle, slow agitation.

8 Move the wash rack to the other wash dish containing clean PB1. Make sure the BeadChips are completely submerged.

9 Move the wash rack up and down for 1 minute, breaking the surface of the PB1 with gentle, slow agitation.

10 When you remove the BeadChips from the wash rack, inspect them for remaining residue.

NOTE
Residue that can adversely affect results is sometimes left on BeadChips after seals are removed. If there is residue left on the BeadChips after the second PB1 wash, use a 200 ul pipette tip for each BeadChip and slowly and carefully scrape off the residues outward (away) from the bead-sections under PB1. Use a new pipette tip for each BeadChip. Then, continue with the protocol.

11 If you are processing more than 8 BeadChips.

a Complete the steps in the next section, Assemble Flow-Through Chambers, for the first eight BeadChips.

b Place the assembled Flow-Through Chambers of the first eight BeadChips on the lab bench in a horizontal position.

NOTE
Keep the Flow-Through Chambers in a horizontal position on the lab bench until all assembled Flow-Through Chambers are ready to be loaded into the Chamber Rack. Do not place the Flow-Through Chambers in the Chamber Rack until all BeadChips are prepared in Flow-Through Chambers.

c Repeat steps 2 through 10 from this section for any additional BeadChips. Use new PB1 for each set of eight BeadChips.

12 ***Immediately*** wash the Hyb Chamber reservoirs with dH_2O and scrub them with a small cleaning brush, ensuring that no PB2 remains in the Hyb Chamber reservoir.

CAUTION
It is important to wash the Hybridization Chamber reservoirs immediately and thoroughly to ensure that no traces of PB2 remain in the wells.

Assemble Flow-Through Chambers

NOTE
The 150 ml used in the BeadChip alignment fixture can be used for up to eight BeadChips. You must used 150 ml of fresh PB1 for every additional set of eight BeadChips.

1 For each BeadChip to be processed, place a black frame into the Multi-Sample BeadChip Alignment Fixture pre-filled with PB1.

Figure 120 Placing Black Frames into Multi-Sample BeadChip Alignment Fixture

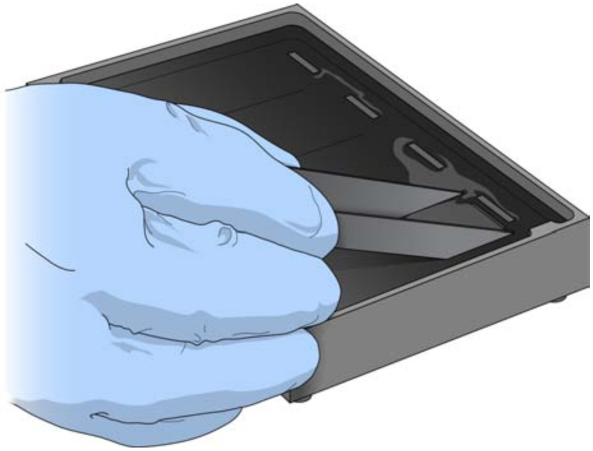

2 Place each BeadChip to be processed into a black frame, aligning its barcode with the ridges stamped onto the Alignment Fixture.

NOTE
Inspect the surface of each BeadChip for residue left by the seal. Use a pipette tip to remove any residue under buffer and be careful not to scratch the bead area.

Figure 121 Placing BeadChip into Black Frame on Alignment Fixture

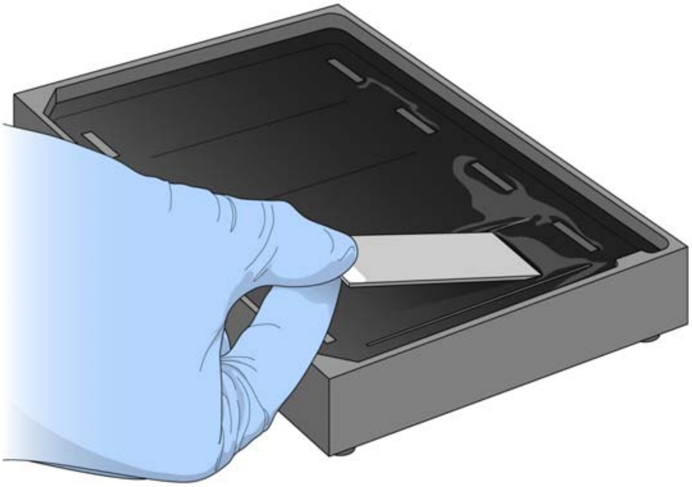

3 Place a clear spacer onto the top of each BeadChip. Use the Alignment Fixture grooves to guide the spacers into proper position.

NOTE
Be sure to use the clear plastic spacers, not the white ones.

Figure 122 Placing Clear Plastic Spacer onto BeadChip

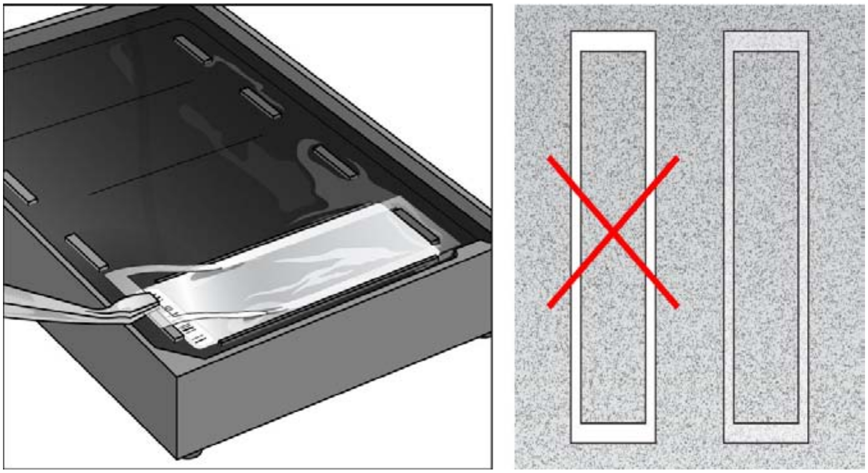

4 Place the Alignment Bar onto the Alignment Fixture.

Figure 123 Placing Alignment Bar onto Alignment Fixture

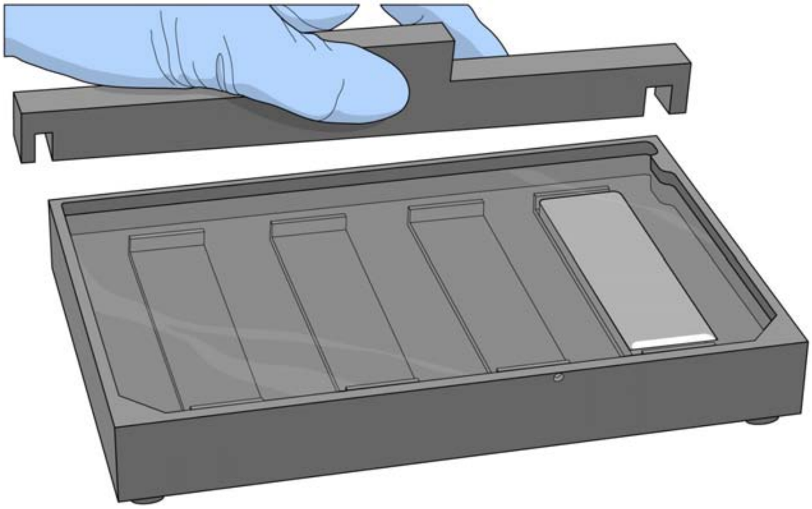

5 Place a clean glass back plate on top of the clear spacer covering each BeadChip. The plate reservoir should be at the barcode end of the BeadChip, facing inward to create a reservoir against the BeadChip surface.

Figure 124 Placing Glass Back Plate onto BeadChip

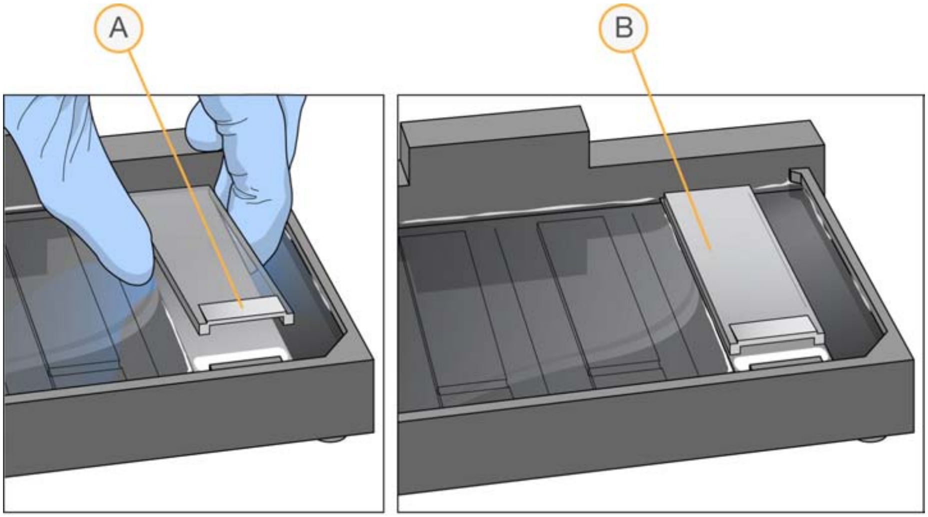

A Reservoir at Barcode End of Glass Back Plate
B Glass Plate Back in Position

6 Attach the metal clamps to the Flow-Through Chambers as follows:
   a Gently push the glass back plate up against the Alignment Bar with one finger.
   b Place the first metal clamp around the Flow-Through Chamber so that one stripe shows between it and the Alignment Bar.
   c Place the second metal clamp around the Flow-Through Chamber at the barcode end, just below the reagent reservoir, so that no stripes show between the clamp and the barcode.

Figure 125 Securing Flow-Through Chamber Assembly with Metal Clamps

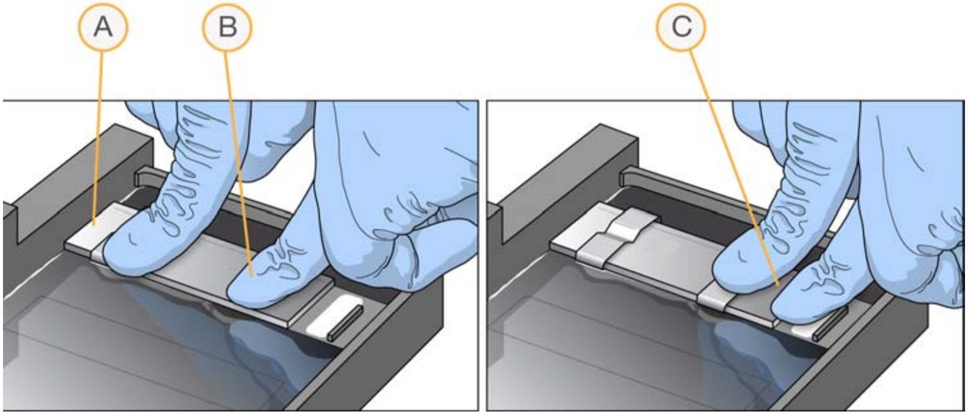

A One Stripe Shows Between First Clamp and Alignment Bar
B Glass Back Plate Pressed Against Alignment Bar
C No Stripes Show Between Second Clamp and Barcode

7 Using scissors, trim the ends of the clear plastic spacers from the Flow-Through Chamber assembly. Slip scissors up over the barcode to trim the other end:
   a Trim spacer ends at the non-barcode end of the assembly.
   b On the barcode end of the assembly, slip scissors up over the barcode to trim spacer ends.

Figure 126 Trimming Spacer Ends from Flow-Through Chamber Assembly

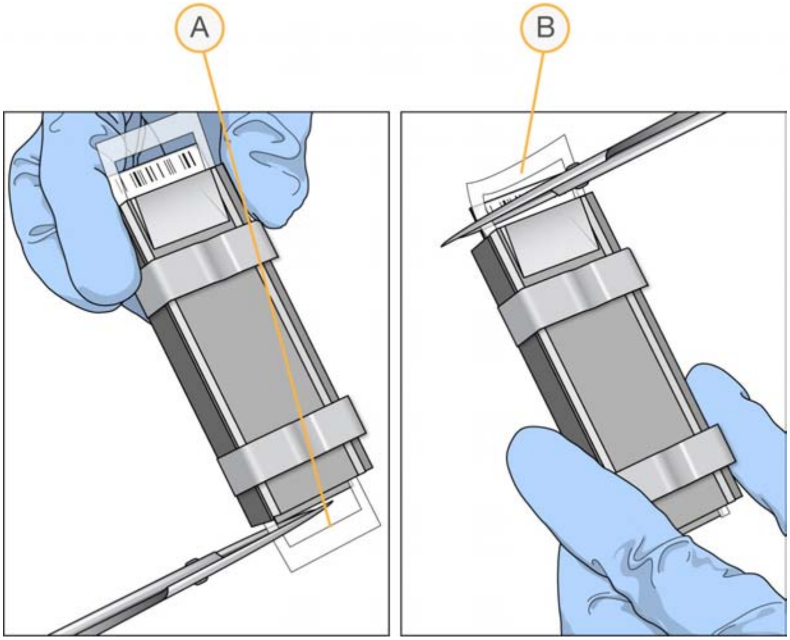

A Trim Spacer at Non-Barcode End of Flow-Through Chamber
B Trim Spacer at Barcode End of Flow-Through Chamber

8 Discard unused reagents in accordance with facility standards.

CAUTION
Place all assembled Flow-Through Chambers on the lab bench in a horizontal position while you perform the preparation steps for XStain BeadChip. Do not place the Flow-Through Chambers in the Chamber Rack until all necessary steps are completed.

Single-Base Extension and Stain BeadChip

Following hybridization, RA1 reagent is used to wash away unhybridized and non-specifically hybridized DNA sample. XC1 and XC2 are added to condition the BeadChip surface for the extension reaction. TEM reagents are dispensed into the Flow-Through Chambers to perform single-base extension of primers hybridized to DNA on the BeadChip. This reaction incorporates labeled nucleotides into the extended primers. 95% formamide/1 mM EDTA is added to remove the hybridized DNA. After neutralization using the XC3 reagent, the labeled extended primers undergo a multi-layer staining process on the Chamber Rack. Next, the Flow-Through Chambers are disassembled. The BeadChips are washed in the PB1 reagent, and then coated with XC4 reagent and dried.

Figure 127 Extending and Staining BeadChip

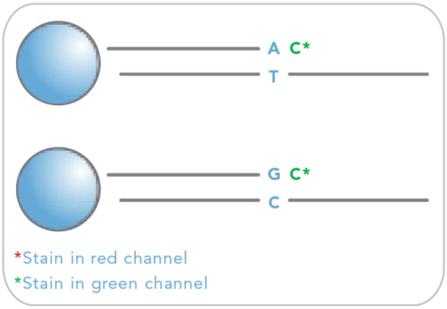

Estimated Time

Robot time:

- 2 hours and 10 minutes for 8 BeadChips
- 2 hours and 25 minutes for 16 BeadChips
- 2 hours and 40 minutes for 24 BeadChips

Dry time: 55 minutes

Consumables

Item	Quantity (Per 8 BeadChips)	Storage	Supplied By
RA1	10 ml (see *Setup* for special instructions)	-15° to -25° C	Illumina
XC1	2 tubes	-15° to -25° C	Illumina
XC2	2 tubes	-15° to -25° C	Illumina
TEM	2 tubes	-15° to -25° C	Illumina
XC3	75 ml	Room temperature	Illumina
STM (Make sure that all STM tubes indicate the same stain temperature on the label)	2 tubes	-15° to -25° C	Illumina
ATM	2 tubes	-15° to -25° C	Illumina
PB1	310 ml	Room temperature	Illumina
XC4	310 ml	-15° to -25° C	Illumina
Alconox Powder Detergent	as needed		Illumina
EtOH	as needed	Room temperature	User
95% formamide/1 mM EDTA	15 ml	-15° to -25° C	User

CAUTION
Pour out only the recommended reagent volume needed for the suggested number of beachships listed in the consumables table of each section. Some of the reagents are used later in the ptotocol.

NOTE
It is important to use fresh RA1 for each protocol step in the assay where it is required. RA1 that has been stored properly and has not been dispensed for use in either the XStain or Resuspension step is considered fresh RA1. After RA1 has been poured out into a reservoir and exposed to room temperature air for extended periods of time, it is no longer fresh.

WARNING
This protocol involves the use of an aliphatic amide that is a probable reproductive toxin. Personal injury can occur through inhalation, ingestion, skin contact, and eye contact. For more information, consult the material data safety sheet for this assay at http://www.illumina.com/msds. Dispose of containers and any unused contents in accordance with the governmental safety standards for your region.

Preparation

- RA1 is shipped frozen. Gradually warm the reagent to room temperature, preferably in a 20–25° C water bath. Gently mix to dissolve any crystals that may be present.
- Place all reagent tubes in a rack in the order in which they will be used. If frozen, allow them to thaw to room temperature, and then gently invert and centrifuge to 3000 xg for 3 minutes.
- On the Lab Tracking Forms, record:
  - Date/Time
  - Operator
  - Robot
  - RA1 barcode
  - XC3 barcode
  - XC1 barcode(s)
  - XC2 barcode(s)
  - TEM barcode(s)
  - STM barcode(s)
  - ATM barcode(s)
  - PB1 barcode
  - XC4 barcode(s)

NOTE
To record information about your assay such as operator information, start and stop times, and barcodes, use the lab tracking form provided at http://www.illumina.com/documentation. This form can be filled out and saved online, or printed and filled in by hand.

Set Up Chamber Rack

1. Ensure the water circulator reservoir is filled with water to the appropriate level. See the *VWR Operator's Manual*, VWR part # 110-229.
2. Turn on the water circulator and set it to a temperature that brings the Chamber Rack to 44° C at equilibrium.
   This temperature may vary depending on facility ambient conditions.

Figure 128 Water Circulator Connected to Chamber Rack

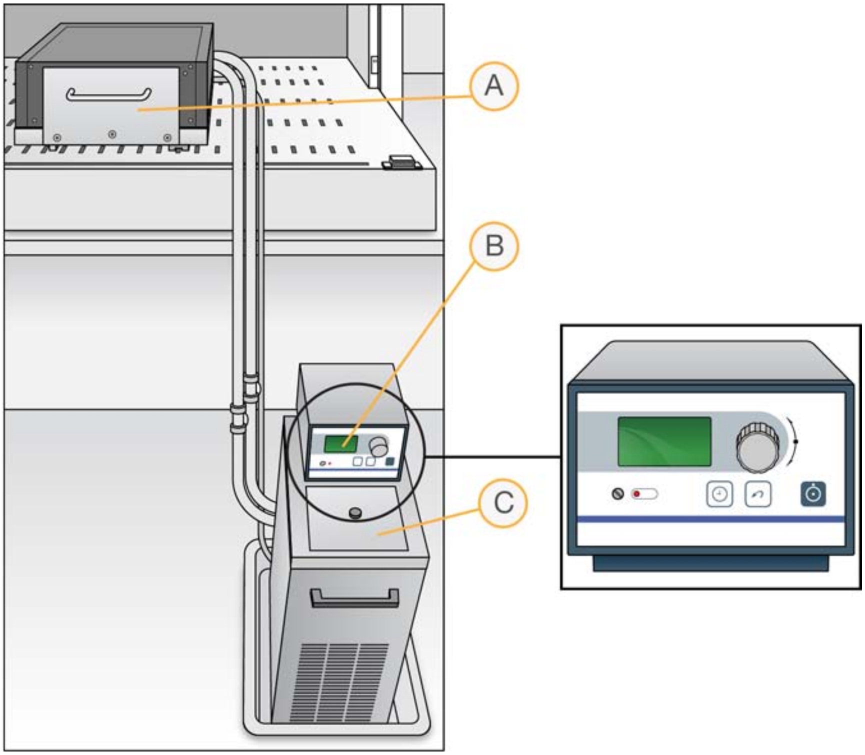

A Chamber Rack on robot Bed
B Water Circulator with Programmable Temperature Controls
C Reservoir Cover

3 The temperature displayed on the water circulator LCD screen may differ from the actual temperature on the Chamber Rack. Confirm the actual temperature using the temperature probe for the Chamber Rack.

4 You must remove bubbles trapped in the Chamber Rack ***each time*** you run this process. Follow instructions in the *Te-Flow (Tecan Flow-Through Module) Operating Manual*, Tecan Doc ID 391584.

5 Use the Illumina Temperature Probe in several locations to ensure that the Chamber Rack is at 44° C.

NOTE
Do not leave the temperature probe in the first three rows of the Chamber Rack. Reserve this space for BeadChips.

Figure 129 IlluminaTemperature Probe and Temperature Probe in Chamber Rack

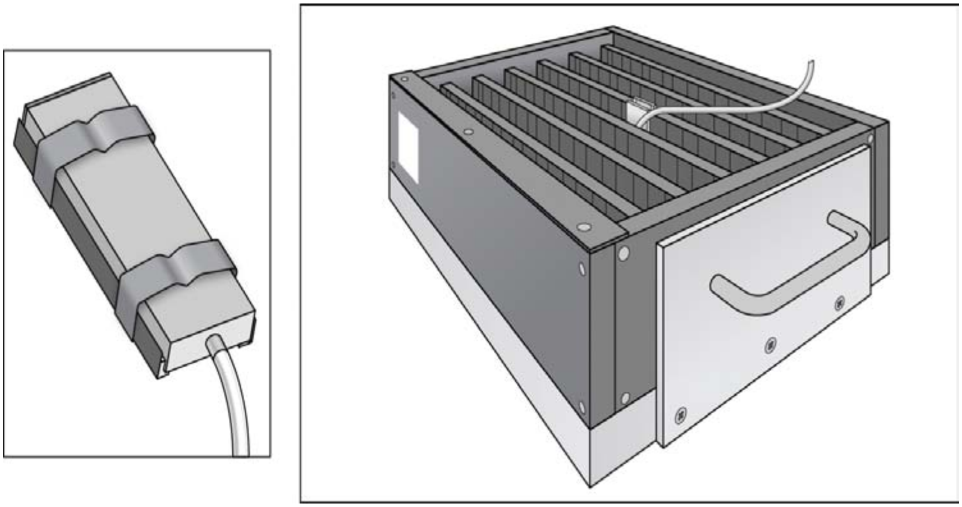

6 For accurate temperature measurement, ensure the Temperature Probe is touching the base of the Chamber Rack.

7 Confirm the Chamber Rack is seated in column 36 on the robot bed.

8 Slide the Chamber Rack back to ensure it is firmly seated.

NOTE
The remaining steps in this protocol must be performed without interruption.

Prepare Robot

For instructions on preparing the robot for use in a protocol and ensuring that the Chamber Rack is properly installed on the post-amplification robot bed, see the *Infinium Assay Lab Setup and Procedures Guide*.

Refer to the figure shown below throughout this protocol.

Figure 130 Tecan Eight-Tip Robot (XStain BeadChip Setup)

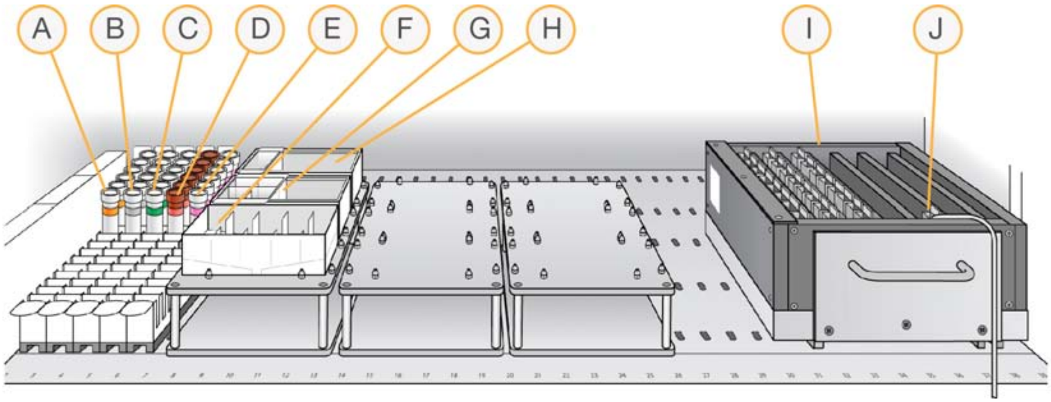

A XC1
B XC2
C TEM
D STM
E ATM
F XC3 in Full Reservoir
G RA1 in Half Reservoir
H 95% Formamide / 1 mM EDTA in Quarter Reservoir
I 24 BeadChips in Chamber Rack
J Temperature Probe

Single-Base Extension and Stain

CAUTION
The remaining steps must be performed without interruption.

1 At the robot PC, select **XStain Tasks | Infinium HD Chemistry | XStain BeadChip**.

2 In the Basic Run Parameters pane, enter the number of BeadChips.
You can process up to 24 BeadChips in the XStain BeadChip process.
The robot PC updates the Required Run Item(s) and the bed map to show the correct position of items on the robot bed. All barcodes must face to the left.

Figure 131 XStain BeadChip Screen

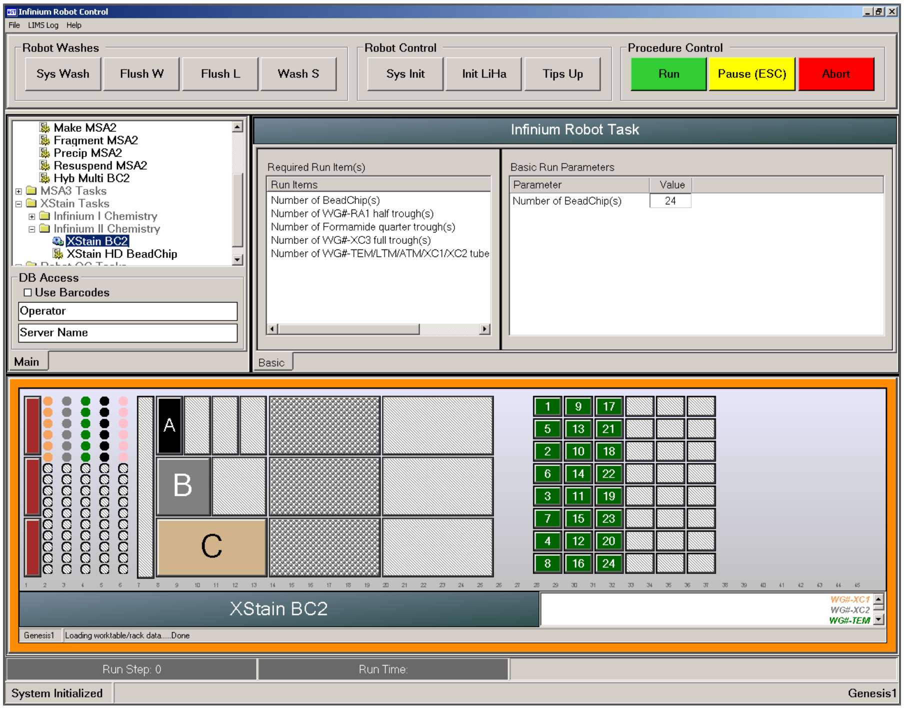

3 If you plan on imaging the BeadChip immediately after the staining process, turn on the iScan or HiScan now to allow the lasers to stabilize.

4 Place a quarter reservoir in the reservoir frame, according to the robot bed map (*Prepare Robot* on page 197), and add 95% formamide/1 mM EDTA as follows:

- 15 ml to process 8 BeadChips
- 17 ml to process 16 BeadChips
- 25 ml to process 24 BeadChips

5 Place a half reservoir in the reservoir frame, according to the robot bed map, and add RA1 in the following volumes:

- 10 ml to process 8 BeadChips
- 20 ml to process 16 BeadChips
- 30 ml to process 24 BeadChips

6 Place a full reservoir in the reservoir frame, according to the robot bed map, and add XC3 in the following volumes:
- 49 ml to process 8 BeadChips
- 97 ml to process 16 BeadChips
- 145 ml to process 24 BeadChips

7 Place each reagent tube (XC1, XC2, TEM, STM, ATM) in the robot tube rack according to the bed map, and remove their caps.

8 Ensure that all items are placed properly on the robot bed, that all caps and seals have been removed, and that all the barcodes face to the right.

Start Robot

1 At the robot PC:

a Click **Run** to start the process.

b Log in if prompted.

c At the prompt, enter the staining temperature. The correct temperature is listed on the STM reagent label. If no temperature is listed, enter 32° C.

Figure 132 Entering XStain Temperature

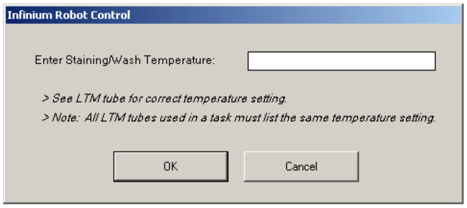

2 When the prompt appears, wait for the Chamber Rack to reach 44° C. Do not load the BeadChips or click **OK** yet.

Figure 133 Adjusting Chamber Rack to 44° C Message

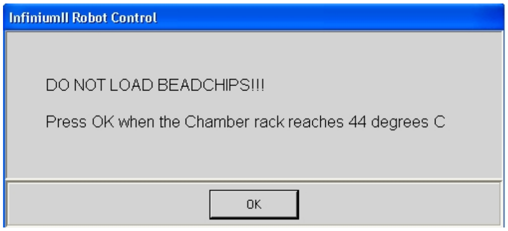

3 Once the temperature probe registers 44° C, click **OK**.

4 When prompted, load the BeadChips and click **OK**.

Figure 134 Load BeadChips Message

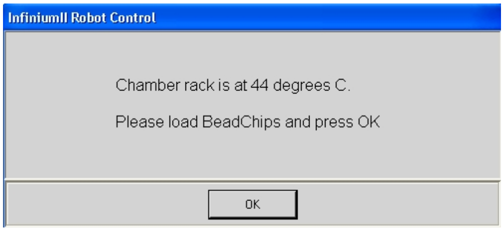

5 Place each assembled Flow-Through Chamber in the first row of the Chamber Rack. Refer to the robot bed map for the correct layout.

6 Ensure each Flow-Through Chamber is properly seated on its rack to allow adequate heat exchange between the rack and the chamber.

7 On the Lab Tracking Forms, record the chamber rack position associated with each BeadChip.

8 Click **OK**. A series of reactions begins, each with a wait time. Message boxes on the robot PC tell you which reaction is occurring and how long the wait time is. The total wait time is 1 hour and 25 minutes.

Table 17 List of Reactions

#	Reagent	Wait Time
1	RA1	3 minutes

#	Reagent	Wait Time
2	XC1	10 minutes
3	XC2	10 minutes
4	TEM	15 minutes
5	Formamide/EDTA	7 minutes
6	XC3	2 minutes
7	STM	10 minutes
8	XC3	7 minutes
9	ATM	10 minutes
10	XC3	7 minutes
11	STM	10 minutes
12	XC3	7 minutes
13	ATM	10 minutes
14	XC3	7 minutes
15	STM	10 minutes
16	XC3	7 minutes

9 When prompted, remove the BeadChips from the Chamber Rack immediately and place them horizontally on the lab bench at room temperature. Click **OK** in the message box.

10 The robot PC sounds an alert and displays a message when the process is complete. Click **OK** to finish the process.

Wash and Coat 8 BeadChips

Follow either the 8 BeadChips Process (shown below) or the 16–24 BeadChips Process (see *Wash and Coat 16–24 BeadChips* on page 214).

Preparation

Before starting the Wash and Coat process, please read these important notes:

Take the utmost care to minimize the chance of lint or dust entering the wash dishes, which could transfer to the BeadChips. Place wash dish covers on wash dishes when stored or not in use. Clean wash dishes with low-pressure air to remove particulates before use.

In preparation for XC4 BeadChip coating, wash the tube racks and wash dishes thoroughly before and after use. Rinse with DI water. Immediately following wash, place racks and wash dishes upside down on a wash rack to dry.

Place Kimwipes in three layers on the lab bench. Place a tube rack on top of these Kimwipe layers. Do not place on absorbent lab pads. You will place the staining rack containing BeadChips on this tube rack after removing it from the XC4 wash dish.

Prepare an additional clean tube rack that fits the internal dimensions of vacuum desiccator for removal of the BeadChips. Allow one rack per 8 BeadChips. No Kimwipes are required under this tube rack.

Equipment Needed

Place the following items on the bench:

- 1 staining rack
- 1 vacuum desiccator
- 1 tube rack
- Self-locking tweezers
- Large Kimwipes
- Vacuum hose

Steps

1 Set up two top-loading wash dishes, labeled "PB1" and "XC4".

2 To indicate the fill volume before filling wash dishes with PB1 and XC4, pour 310 ml water into the wash dishes and mark the water level on the side. Empty the water from the wash dish. This enables you to pour reagent directly from the PB1 and XC4 bottles into the wash dishes, minimizing contaminant transfer from labware to wash dishes.

Figure 135 PB1 and XC4 Wash Dishes with BeadChip Rack

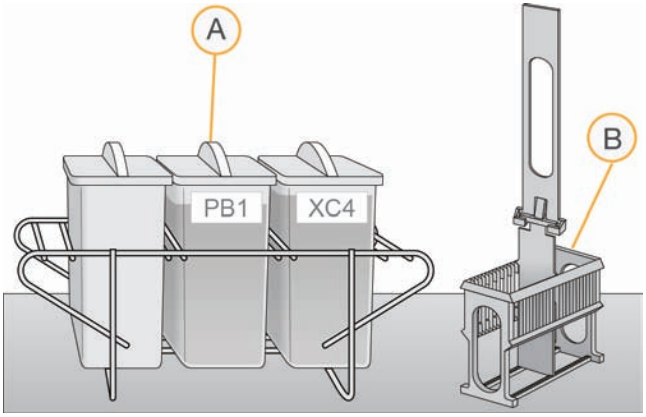

A Wash Dishes
B Staining Rack

3 Pour 310 ml PB1 into the wash dish labeled "PB1."

4 Submerge the unloaded staining rack into the wash dish with the locking arms and tab ***facing toward*** you. This orients the staining rack so that you can safely remove the BeadChips. Let the staining rack sit in the wash dish. You will use it to carry the BeadChips after disassembling the Flow-Through Chambers.

CAUTION
Handle the BeadChips only by the edges or the barcode end. Do not let the BeadChips dry out.

Figure 136 Staining Rack Locking Arms and Tabs

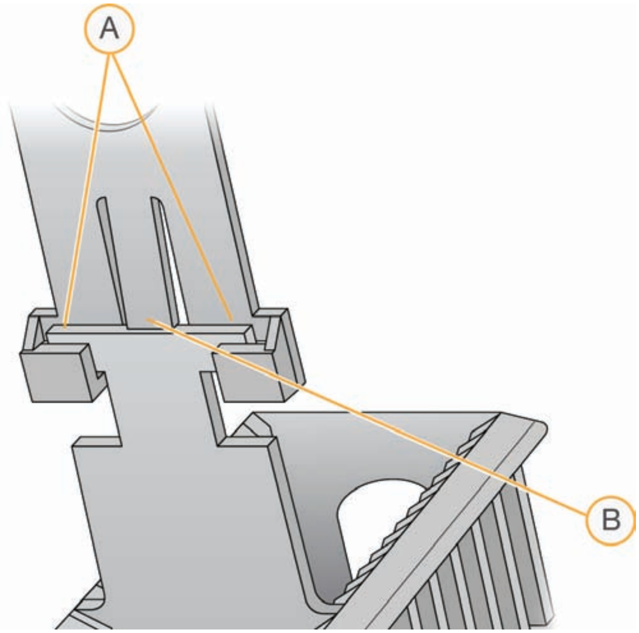

A Locking Arms
B Tabs

CAUTION
If the staining rack handle is not correctly oriented, the BeadChips may be damaged when you remove the staining rack handle before removing the BeadChips.

CAUTION
Do not leave the BeadChips in the PB1 for more than 30 minutes.

CAUTION
Do not let the XC4 sit for more than 10 minutes.

CAUTION
Do not touch the stripes with the wipe or allow EtOH to drip onto the stripes.

5 One at a time, disassemble each Flow-Through Chamber:
   a Use the dismantling tool to remove the two metal clamps.

CAUTION
It is important to use the dismantling tool to avoid chipping the glass back plates.

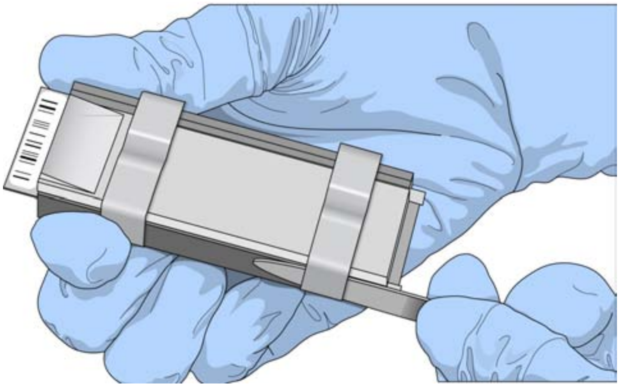

Figure 137 Removing the Metal Clamps from Flow-Through Chamber

   b Remove the glass back plate.
   c Set the glass back plate aside. When you finish the XStain BeadChip protocol, clean the glass back plates as described in the SOP.
   d Remove the spacer. To avoid damaging the stripes on the BeadChip, pull the spacer out so that the long sides slide along the sides of the BeadChip.
   e Remove the BeadChip.

CAUTION
Do not touch the face of the BeadChips. Handle them by the barcode end or by the edges.

6 Place the BeadChips in the staining rack while it is submerged in PB1. Put four BeadChips above the staining rack handle and four below. The BeadChip barcodes should ***face away*** from you; the locking arms on the handle should ***face towards*** you.
If necessary, briefly lift the staining rack out of the wash dish to seat the BeadChip. Replace it immediately after inserting each BeadChip.

7 Ensure that the BeadChips are completely submerged.

CAUTION
Do not allow the BeadChips to dry. Submerge each BeadChip in the wash dish as soon as possible.

8 Move the staining rack up and down 10 times, breaking the surface of the PB1.

NOTE
If the top edges of the BeadChips begin to touch during either PB1 or XC4 washes, gently move the staining rack back and forth to separate the slides. It is important for the solution to circulate freely between all BeadChips.

Figure 138 Washing BeadChips in PB1

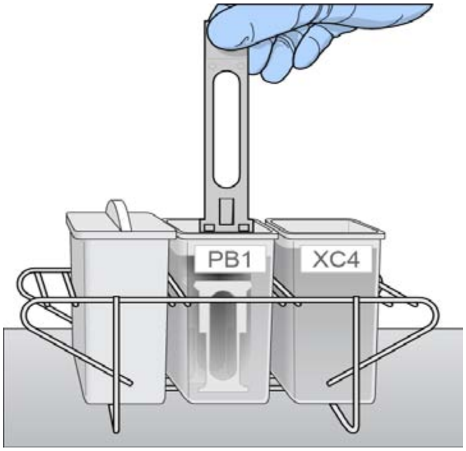

9 Allow the BeadChips to soak for an additional 5 minutes.

NOTE
Do not leave the BeadChips submerged in PB1 for longer than 30 minutes.

10 Shake the XC4 bottle vigorously to ensure complete resuspension.

11 Pour 310 ml XC4 into the dish labeled "XC4," and cover the dish to prevent any lint or dust from falling into the solution.

NOTE
Use the XC4 within 10 minutes after filling the wash dish.

12 Remove the staining rack from the PB1 dish and place it directly into the wash dish containing XC4. For proper handling and coating, The barcode labels on the BeadChips must ***face away*** from you; the locking arms on the handle must ***face towards*** you.

Figure 139 Moving BeadChips from PB1 to XC4

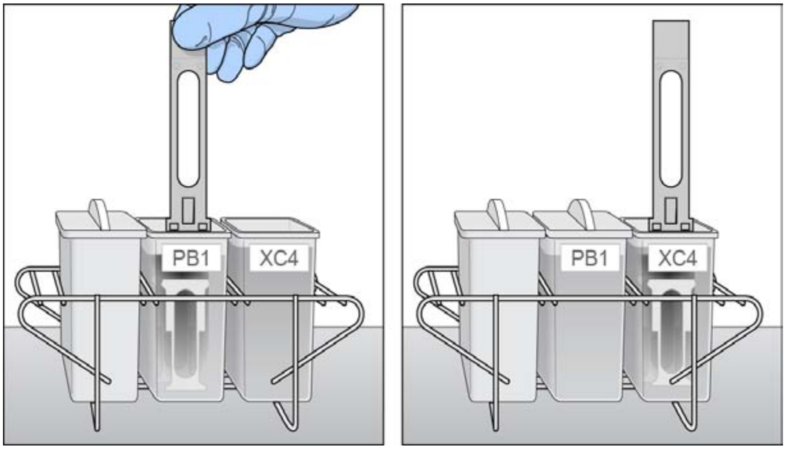

13 Move the staining rack up and down 10 times, breaking the surface of the XC4.

NOTE
If the top edges of the BeadChips begin to touch during either PB1 or XC4 washes, gently move the staining rack back and forth to separate the slides. It is important for the solution to circulate freely between all BeadChips.

14 Allow the BeadChips to soak for an additional 5 minutes.

CAUTION
Use XC4 only once. To process subsequent BeadChips, use a new, clean wash dish with fresh XC4.

15 Prepare a clean tube rack for the staining rack by placing two folded Kimwipes under the tube rack.

16 Prepare one additional tube rack per 8 BeadChips (Illumina-provided from VWR catalog # 60916-748) that fits the internal dimensions of vacuum desiccator

17 Remove the staining rack in one smooth, rapid motion and place it directly on the prepared tube rack, making sure the barcodes *face up* and the locking arms and tabs *face down*.

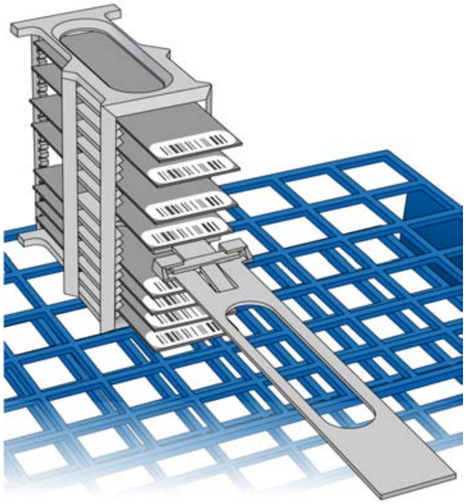

Figure 140 Staining Rack in Correct Orientation

18 To ensure uniform coating, place the staining rack on the center of the tube rack, avoiding the raised edges.

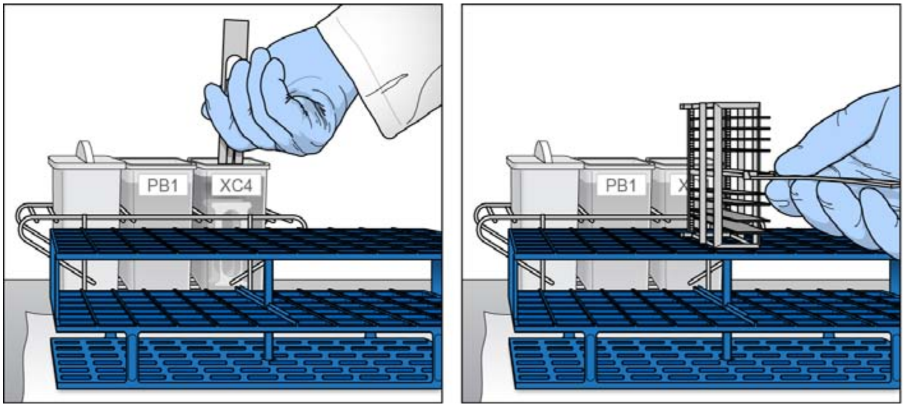

Figure 141 Moving the BeadChip Carrier from XC4 to Tube Rack

19 For each of the top four BeadChips, working top to bottom:

a Continuing to hold the staining rack handle, carefully grip each BeadChip at its barcode end with self-locking tweezers.

NOTE
The XC4 coat is slippery and makes the BeadChips difficult to hold. The self-locking tweezers grip the BeadChip firmly and help prevent damage.

b Place each BeadChip on a tube rack with the barcode *facing up and towards* you.

Figure 142 BeadChips on Tube Rack

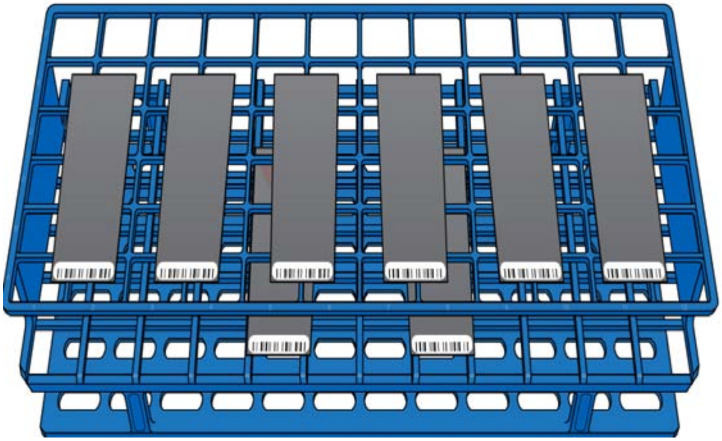

20 Holding the top of the staining rack in position, gently remove the staining rack handle by grasping the handle between the thumb and forefinger. Push the tab up with your thumb and push the handle away from you (unlocking the handle), then pull up the handle and remove.

Figure 143 Removing Staining Rack Handle

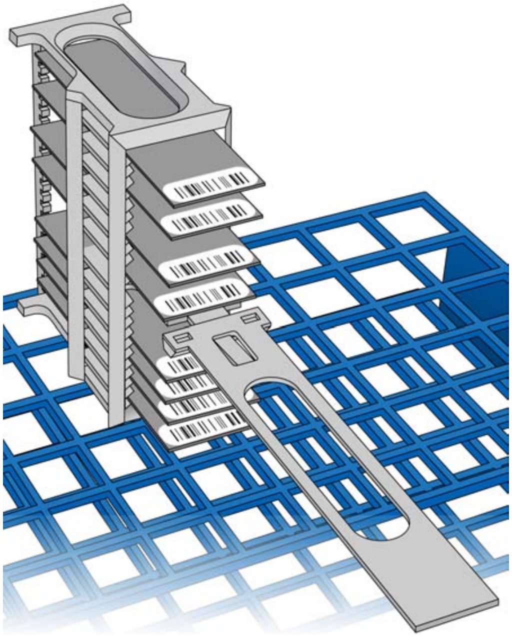

A Tabs
B Handles

21 Remove the remaining BeadChips to the tube rack as shown in the figure above, with six BeadChips on top of the rack and two BeadChips on the bottom. The barcode ends should be towards you, and the BeadChips should be completely horizontal.

CAUTION
To prevent wicking and uneven drying, do not allow the BeadChips to rest on the edge of the tube rack or to touch each other while drying.

22 Place the tube rack in the vacuum desiccator. Each desiccator can hold one tube rack (8 BeadChips).

CAUTION
Ensure the vacuum valve is seated tightly and securely.

23 Remove the red plug from the three-way valve before applying vacuum pressure.

24 Start the vacuum, using at least 508 mm Hg (0.68 bar).

25 To ensure that the desiccator is properly sealed, gently lift the lid of the vacuum desiccator. It should not lift off the desiccator base.

Figure 144 Testing Vacuum Seal

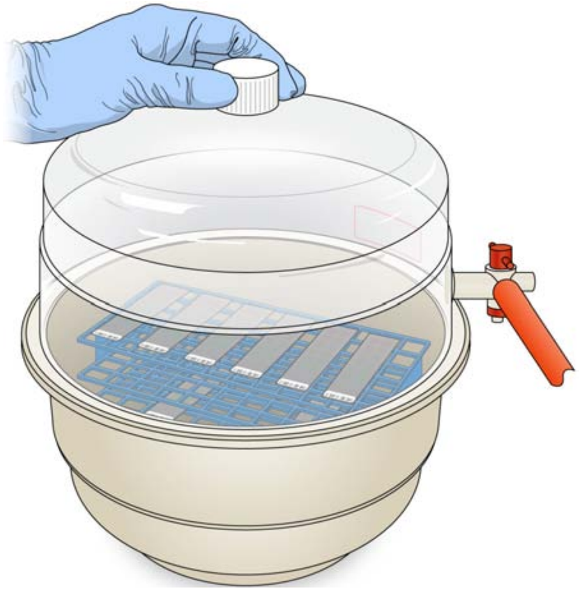

26 Dry under vacuum for 50–55 minutes.
Drying times may vary according to room temperature and humidity.

27 Release the vacuum by turning the handle very slowly.

WARNING
Air should enter the desiccator very slowly to avoid disturbing the contents. Improper use of the vacuum desiccator can result in damage to the BeadChips. This is especially true if you remove the valve plug while a vacuum is applied. For detailed vacuum desiccator instructions, see the documentation included with the desiccator.

28 Store the desiccator with the red valve plug in the desiccator's three-way valve to stop accumulation of dust and lint within the valve port.

29 Touch the borders of the chips (**do not touch the stripes**) to ensure that the etched, barcoded side of the BeadChips are dry to the touch.

30 If the underside feels tacky, manually clean the underside of the BeadChip to remove any excess XC4. The bottom two BeadChips are most likely to have some excess.

a Hold the BeadChip at a downward angle to prevent excess EtOH from dripping from the wipe onto the stripes.

b Wipe along the underside of the BeadChip five or six times, until the surface is clean and smooth.

CAUTION
Do not touch the stripes.

31 Clean the glass back plates. For instructions, see the SOP.

32 Clean the Hyb Chambers:

a Remove the rubber gaskets from the Hyb Chambers.

b Rinse all Hyb Chamber components with DI water.

c Thoroughly rinse the eight humidifying buffer reservoirs.

33 Discard unused reagents in accordance with facility standards.

CAUTION
Handle the BeadChips only by the edges or the barcode end. Do not let the BeadChips dry out.

CAUTION
Do not leave the BeadChips in the PB1 for more than 30 minutes.

CAUTION
Do not touch the stripes with the wipe or allow EtOH to drip into the stripes.

34 Do one of the following:

- Proceed to *Illumina GenomeStudio* on page 229 or *Image BeadChip* on page 227.
- Store the BeadChips in the Illumina BeadChip Slide Storage Box inside a vacuum desiccator at room temperature. Be sure to image the BeadChips within 72 hours.

Wash and Coat 16–24 BeadChips

Follow either the 16–24 BeadChips Process (shown below) or the 8 BeadChips Process (see *Wash and Coat 8 BeadChips* on page 203).

Preparation

Before starting the Wash and Coat process, please read these important notes:

Take the utmost care to minimize the chance of lint or dust entering the wash dishes, which could transfer to the BeadChips. Place wash dish covers on wash dishes when stored or not in use. Clean wash dishes with low-pressure air to remove particulates before use.

In preparation for XC4 BeadChip coating, wash the tube racks and wash dishes thoroughly before and after use. Rinse with DI water. Immediately following wash, place racks and wash dishes upside down on a wash rack to dry.

Place Kimwipes in three layers on the lab bench. Place a tube rack on top of these Kimwipe layers. Do not place on absorbent lab pads. You will place the staining rack containing BeadChips on this tube rack after removing it from the XC4 wash dish.

Prepare an additional clean tube rack that fits the internal dimensions of vacuum desiccator for removal of the BeadChips. Allow one rack per 8 BeadChips. No Kimwipes are required under this tube rack.

Equipment Needed

- 1 staining rack
- 3 vacuum desiccators (1 per 8 samples)
- 3 tube racks (1 per 8 samples)
- Self-locking tweezers
- Large Kimwipes
- Vacuum hose

Steps

1 Dispense 285 ml PB1 into a wash dish, and then cover the dish.

2 Set up two top-loading wash dishes, labeled “PB1” and “XC4”.

3 To indicate the fill volume before filling wash dishes with PB1 and XC4, pour 285 ml water into the wash dishes and mark the water level on the side. Empty the water from the wash dish. This enables you to pour reagent directly from the PB1 and XC4 bottles into the wash dishes, minimizing contaminant transfer from labware to wash dishes.

Figure 145 PB1 and XC4 Wash Dishes with Staining Rack

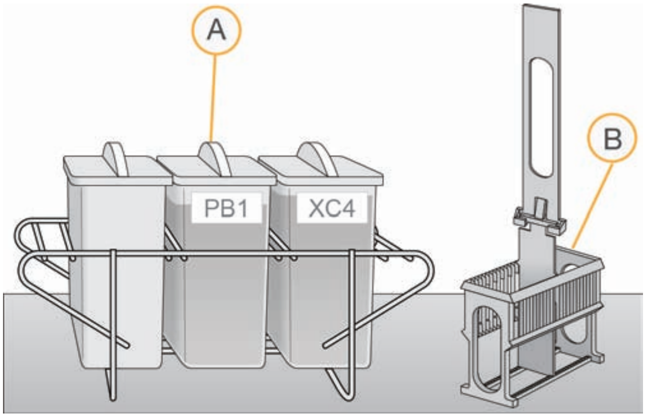

A Wash Dishes
B Staining Rack

4 Pour 285 ml PB1 into the wash dish labeled "PB1."

5 Submerge the unloaded staining rack into the wash dish with the locking arms and tab ***facing you***. This orients the staining rack so that you can safely remove the BeadChips.

Figure 146 Staining Rack Locking Arms and Tabs

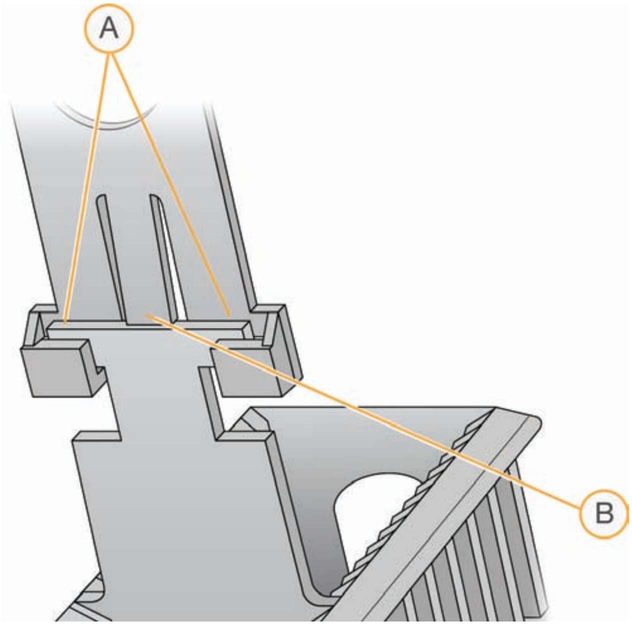

A Locking Arms
B Tabs

CAUTION
If the staining rack handle is not correctly oriented, the BeadChips may be damaged when you remove the staining rack handle before removing the BeadChips.

6 Let the staining rack sit in the wash dish. You will use it to carry the BeadChips after disassembling the Flow-Through Chambers.

7 One at a time, disassemble each Flow-Through Chamber:
  a Using the dismantling tool, remove the two metal clamps.

CAUTION
It is important to use the dismantling tool to avoid chipping the glass back plates.

Figure 147 Removing Metal Clamps from Flow-Through Chamber

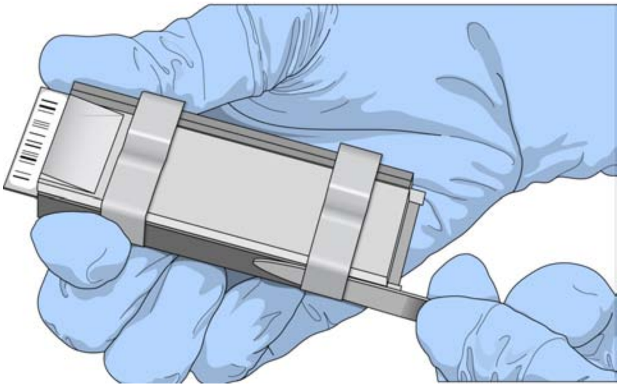

b Remove the glass back plate.

c Set the glass back plates aside. When you finish the XStain BeadChip protocol, clean the glass back plates as described in the *Infinium Assay Lab Setup and Procedures Guide*.

d Remove the spacer.

e Remove the BeadChip.

CAUTION
Do not touch the face of the BeadChips. Handle them by the barcode end or by the edges.

8 Place BeadChips in the staining rack while it is submerged in PB1.

- For 16 BeadChips, place 8 above the handle and 8 below.
- For 24 BeadChips, place 12 above the handle and 12 below.

The BeadChip barcodes should ***face away*** from you, while the locking arms and tab ***face towards*** you.

If necessary, briefly lift the staining rack out of the wash dish to seat the BeadChip. Replace it immediately after inserting the BeadChip.

9 Ensure that the BeadChips are completely submerged.

CAUTION
Do not allow the BeadChips to dry. Submerge each BeadChip in the wash dish as soon as possible.

10 Move the staining rack up and down 10 times, breaking the surface of the PB1.

NOTE
If the top edges of the BeadChips begin to touch during either PB1 or XC4 washes, gently move the staining rack back and forth to separate the slides. It is important for the solution to circulate freely between all BeadChips.

11 Allow the BeadChips to soak for an additional 5 minutes.

Figure 148 Washing BeadChips in PB1

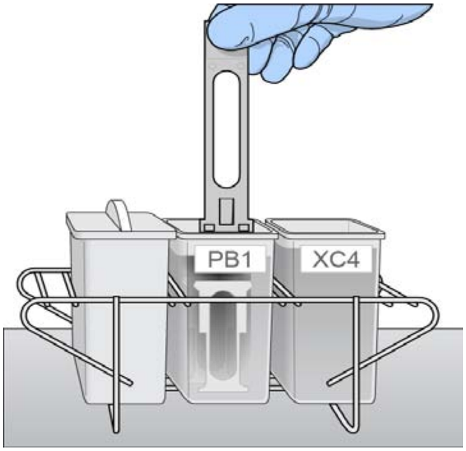

NOTE
Do not leave the BeadChips submerged in PB1 for longer than 30 minutes.

12 Pour 285 ml XC4 into the dish labeled "XC4," and cover the dish to prevent any lint or dust from falling into the solution. Place the bottle with excess XC4 in a readily available location for topping off the 'XC4' wash dish during the coating procedure.

NOTE
Use the XC4 within 10 minutes after filling the wash dish.

13 Remove the staining rack from the dish containing PB1 and place it directly into the wash dish containing XC4. The barcode labels on the BeadChips must ***face away*** from you, while the locking arms on the handle ***face towards*** you, for proper handling and coating.

14 Move the staining rack up and down 10 times, breaking the surface of the XC4.

Figure 149 Moving BeadChips from PB1 to XC4

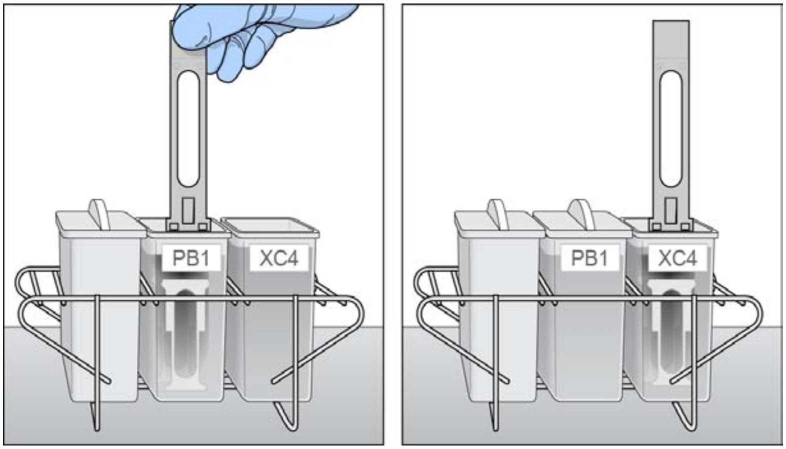

NOTE
If the top edges of the BeadChips begin to touch during either PB1 or XC4 washes, gently move the staining rack back and forth to separate the slides. It is important for the solution to circulate freely between all BeadChips.

15 Allow the BeadChips to soak for an additional 5 minutes.

CAUTION
Use XC4 only once. To process subsequent BeadChips, use a new, clean wash dish with fresh XC4.

16 Prepare a clean tube rack for the staining rack by placing two folded Kimwipes under the tube rack.

17 Prepare one additional tube rack per 8 BeadChips (Illumina-provided from VWR catalog # 60916-748) that fits the internal dimensions of the vacuum desiccator.

18 Remove the staining rack in one smooth, rapid motion and place it directly on the prepared tube rack, making sure the barcodes face ***up*** and the locking arms and tab face ***down***.

Figure 150 Staining Rack in Correct Orientation

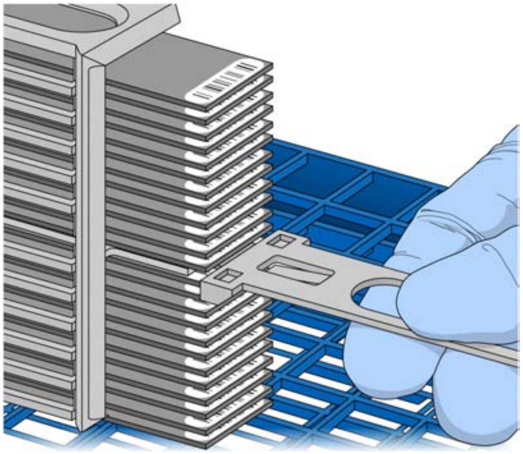

19 To ensure uniform coating, place the staining rack on the center of the tube rack, avoiding the raised edges.

Figure 151 Moving the Staining Rack from XC4 to Tube Rack

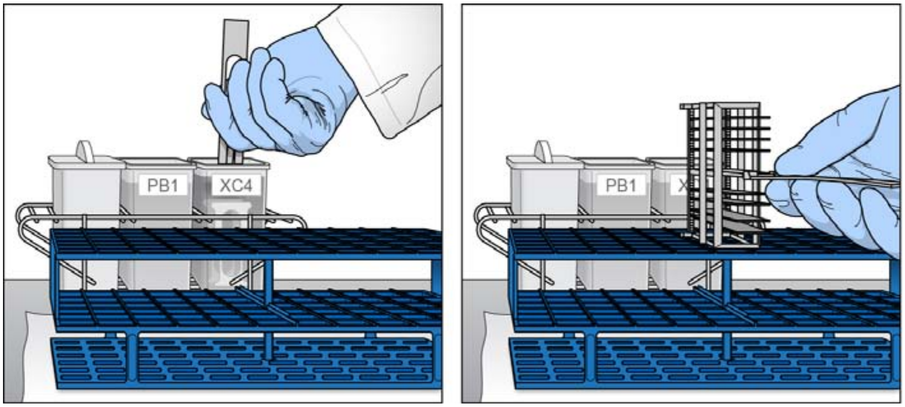

20 For the **top eight** BeadChips, working top to bottom:

a Continuing to hold the staining rack handle, carefully grip each BeadChip at its barcode end with self-locking tweezers.

NOTE
The XC4 coat is slippery and makes the BeadChips difficult to hold. The self-locking tweezers grip the BeadChip firmly and help prevent damage.

b Put the eight BeadChips on the tube rack as shown in Figure 152, with six BeadChips on top of the rack and two BeadChips on the bottom. The barcode ends should be towards you, and the BeadChips should be completely horizontal.

CAUTION
To prevent wicking and uneven drying, do not allow the BeadChips to rest on the edge of the tube rack or to touch each other while drying.

Figure 152 BeadChips on Tube Rack

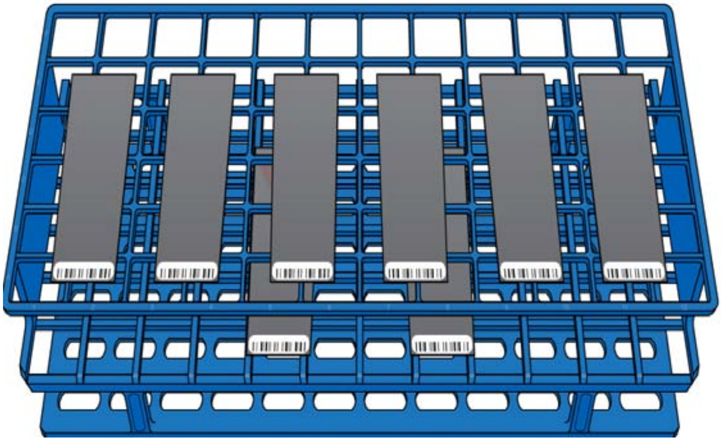

If you are processing 16 BeadChips, 8 remain below the staining rack handle. If you are processing 24 BeadChips, 4 remain above the staining rack handle and 12 below.

21 Return the staining rack to the XC4 wash dish and top off wash dish until BeadChips are completely covered with remaining XC4 reagent.

22 Soak the BeadChips for 10 seconds.

23 Dry the **first 8** BeadChips:

a Place the tube rack with the first 8 BeadChips into the desiccator. Check the vacuum pressure and make sure that the valve is securely attached.

b Start the vacuum, using at least 508 mm Hg (0.68 bar).

c To ensure that the des sic at or is properly sealed, gently lift the lid of the vacuum desiccator. It should not lift off the desiccator base.

d Dry under vacuum for 50–55 minutes.

Drying times may vary according to room temperature and humidity.

24 Remove the staining rack with the remaining BeadChips in one rapid motion from the XC4 wash dish and place it directly on the tube rack. Ensure that the BeadChips are horizontal with the barcodes facing up.

25 If you are processing 24 BeadChips, remove the 4 BeadChips that remain above the staining rack handle and place them on the tube rack.

26 (For both 16- and 24-BeadChip processes) Holding the top of the staining rack in position, grasp the handle between your thumb and forefinger. Push the tab up with your thumb and push the handle away from you to unlock it. Pull up the handle and remove.

Figure 153 Removing Staining Rack Handle

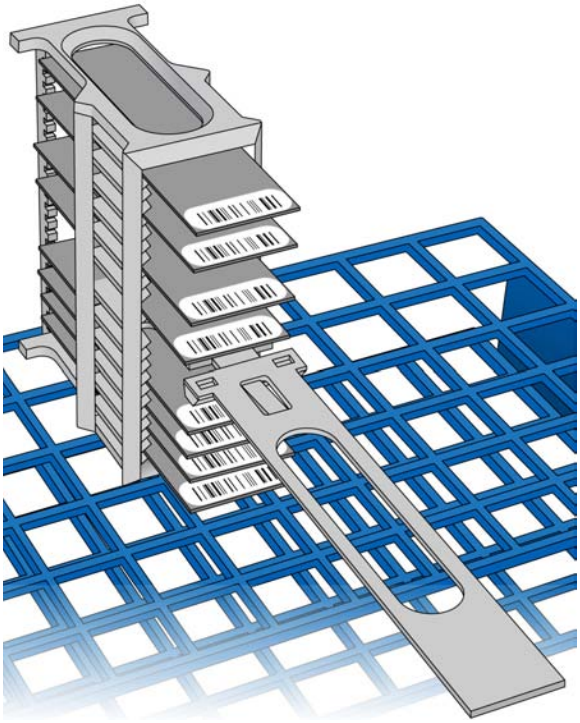

A Tabs
B Handles

27 Place BeadChips on the tube rack until there are six BeadChips on top of the rack and two BeadChips on the bottom. The barcode ends should be towards you, and the BeadChips should be completely horizontal.

   If you are processing 24 BeadChips, 8 remain in the staining rack.

28 If you are processing 24 BeadChips:

   a Return the staining rack with the last 8 BeadChips to the XC4 wash dish and top off the wash dish until BeadChips are completely covered with remaining XC4 reagent.

   b Soak the BeadChips for 10 seconds.

29 Dry the **second set of 8** BeadChips:

a Place the tube rack with the second set of 8 BeadChips into the desiccator. Check the vacuum pressure and make sure that the valve is securely attached.

b Start the vacuum, using at least 508 mm Hg (0.68 bar).

c To ensure that the des sic at or is properly sealed, gently lift the lid of the vacuum desiccator. It should not lift off the desiccator base.

d Dry under vacuum for 50–55 minutes.

30 If you are processing 24 BeadChips:

a Remove staining rack with the remaining 8 BeadChips in one rapid motion from the ‘XC4’ wash dish and place it directly on tube rack. Ensure that the BeadChips are horizontal with the barcodes facing up.

b Place BeadChips on the tube rack as shown in Figure 152 until there are six BeadChips on top of the rack and two BeadChips on the bottom. The barcode ends should be towards you, and the BeadChips should be completely horizontal.

a Place the tube rack with the **third set of** 8 BeadChips into the desiccator. Check the vacuum pressure and make sure that the valve is securely attached.

b Start the vacuum, using at least 508 mm Hg (0.68 bar).

c To ensure that the des sic at or is properly sealed, gently lift the lid of the vacuum desiccator. It should not lift off the desiccator base.

Figure 154 Testing Vacuum Seal

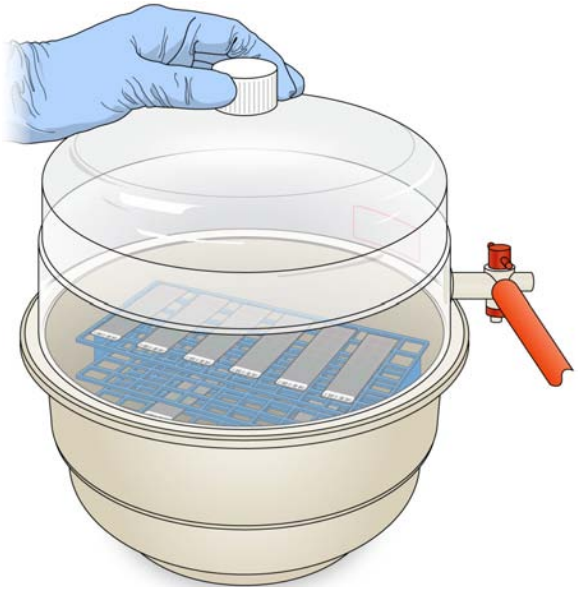

d Dry under vacuum for 50–55 minutes.

31 Release the vacuum by turning the handle very slowly.

WARNING
Air should enter the desiccator very slowly to avoid disturbing the contents. Improper use of the vacuum desiccator can result in damage to the BeadChips. This is especially true if you remove the valve plug while a vacuum is applied. For detailed vacuum desiccator instructions, see the documentation included with the desiccator.

32 Store the desiccator with the red valve plug in the desiccator's three-way valve to stop accumulation of dust and lint within the valve port. Remove the red plug from the three-way valve before applying vacuum pressure.

33 Touch the borders of the chips (**do not touch the stripes**) to ensure that the etched, bar-coded side of the BeadChips are dry to the touch.

34 If the underside feels tacky, manually clean the underside of the BeadChip to remove any excess XC4. The bottom two BeadChips are the most likely to have some excess.

a Hold the BeadChip at a downward angle to prevent excess EtOH from dripping from the wipe onto the stripes.
a Wrap a pre-saturated ProStat EtOH Wipe around your index finger.
b Wipe along the underside of the BeadChip five or six times, until the surface is clean and smooth.

CAUTION
Do not touch the stripes.

35 Clean the glass back plates. For instructions, see the *Infinium Assay Lab Setup and Procedures Guide*.

36 Clean the Hyb Chambers:
a Remove the rubber gaskets from the Hyb Chambers.
b Rinse all Hyb Chamber components with DI water.
c Thoroughly rinse the eight humidifying buffer reservoirs.

37 Discard unused reagents in accordance with facility standards.

38 Do one of the following:
- Proceed to *Image BeadChip* on page 227
- Store the BeadChips in the Illumina BeadChip Slide Storage Box inside a vacuum desiccator at room temperature. Image the BeadChips within 72 hours.

Image BeadChip

The Illumina iScan or HiScan Systems scan the BeadChip, using a laser to excite the fluorophore of the single-base extension product on the beads. The scanner records high-resolution images of the light emitted from the fluorophores. See the chapter on imaging BeadChips in the SOP.

Figure 155 Imaging BeadChip

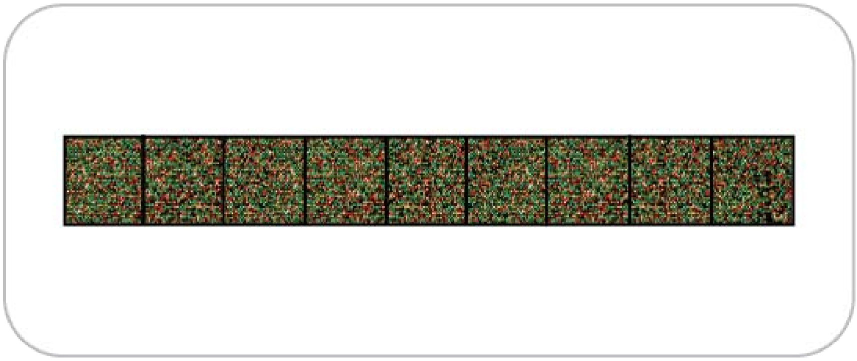

Image BeadChip on the iScan System

The iScan™ Reader is an easy-to-use, laser-based, high-resolution benchtop optical imaging system that can rapidly scan and collect large volumes of data from Illumina DNA analysis and RNA analysis high-density BeadChips.

Image BeadChip on the HiScan System

The HiScan™ System is an easy-to-use, laser-based, high-resolution benchtop optical imaging system that integrates the high-throughput capability of genotyping and gene expression arrays with the power and resolution of next-generation sequencing, delivering unprecedented flexibility for experimental design.

Scanning Settings for Imaging BeadChips on the HiScan and iScan Systems

Use the **Methylation NXT** setting when scanning BeadChips using the HiScan or iScan systems. For general information about scan settings, see the *iScan System User Guide* or

HiScanSQ System User Guide.

Illumina GenomeStudio

Illumina GenomeStudio, Illumina's integrated data analysis software platform, provides a common environment for analyzing data obtained from microarray and sequencing technologies. Within this common environment, or framework, the Illumina GenomeStudio software modules allow you to perform application-specific analyses. The Illumina GenomeStudio Methylation Module, included with your Illumina Infinium Methylation Assay system, is an application for extracting genome-wide DNA methylation data from data files collected from systems such as the Illumina HiScan Reader. Experiment performance is based on built-in controls that accompany each experiment.

Data analysis features of the Illumina GenomeStudio Methylation Module include:

- Choice of assay analysis within a single application
- Data tables for information management and manipulation
- Plotting and graphing tools
- Whole-genome display of sample data in the IGV (Illumina Genome Viewer)
- Data visualization of one or more samples in the ICB (Illumina Chromosome Browser)
- Data normalization
- Custom report file formats
- Differential methylation analysis
- Assay-specific controls dashboards

For feature descriptions and instructions on using the Illumina GenomeStudio platform to visualize and analyze genome-wide DNA methylation data, see the *GenomeStudio Framework User Guide* and the *GenomeStudio Methylation Module User Guide*.

For technical assistance, contact Illumina Technical Support.

System Controls

Chapter 4

Introduction 232
Control Dashboard 233
Controls Table 234
Control Diagrams 236

Introduction

This appendix describes the controls used in the Illumina Infinium HD Methylation Assay, including expected outcomes. Diagrams are included with descriptions for sample-independent and sample-dependent controls as well as controls that are specific to the green channel or red channel. The controls are useful both by themselves and in combination with each other.

Sample-Independent Controls

The sample-independent controls let you evaluate the quality of specific steps in the process flow, and include:

- Staining controls
- Extension controls
- Target removal controls
- Hybridization controls

Sample-Dependent Controls

The sample-dependent controls let you evaluate performance across samples, and include:

- Bisulfite conversion I controls
- Bisulfite conversion II controls
- Specificity I controls
- Specificity II controls
- Non-polymorphic (NP) controls
- Negative controls

Several key steps in the Illumina Infinium HD Methylation Assay require evaluation of both the red and green color channels. For these instances, both red and green channel controls are included.

Control Dashboard

To view the controls, create an Infinium Methylation analysis workspace using the GenomeStudio Wizard, as described in the *GenomeStudio Framework User Guide* and its *GenomeStudio Methylation Module User Guide*. You can then view the controls performance from GenomeStudio's **Analysis | View Controls Dashboard** menu.

Controls Table

Table 18 Controls

Purpose	Name	Number on the Array	Evaluate Green (GRN)	Evaluate Red (RED)	Expected Intensity
**Sample-Independent Controls**					
Staining	DNP (High)	1	-	+	High
Staining	DNP (Bgnd)	1	-	+	Background
Staining	Biotin (Med)	1	+	-	High
Staining	Biotin (Bgnd)	1	+	-	Background
Extension	Extension (A), (T)	2	-	+	High
Extension	Extension (C), (G)	2	+	-	High
Hybridization	Hyb (Low)	1	+	-	Low
Hybridization	Hyb (Medium)	1	+	-	Medium
Hybridization	Hyb (High)	1	+	-	High
Target Removal	Target Removal 1, 2	2	+	-	Low
**Sample-Dependent Controls**					
Bisulfite conversion I	BC conversion I C1, C2, C3	3	+	-	High
Bisulfite conversion I	BC conversion I U1, U2, U3	3	+	-	Background
Bisulfite conversion I	BC conversion I C4, C5, C6	3	-	+	High

Purpose	Name	Number on the Array	Evaluate Green (GRN)	Evaluate Red (RED)	Expected Intensity
Bisulfite conversion I	BC conversion I U4, U5, U6	3	-	+	Background
Bisulfite conversion II	BC conversion II 1, 2, 3, 4	4	-	+	High
Specificity I	GT mismatch 1, 2, 3 (PM)	3	+	-	High
Specificity I	GT mismatch 1, 2, 3 (MM)	3	+	-	Background
Specificity II	Specificity 1, 2, 3	3	-	+	High
Non-Polymorphic	NP (A), (T)	2	-	+	High
Non-Polymorphic	NP (C), (G)	2	+	-	High
Negative	Average*	600	+	+	Background
Negative	StdDev**		+	+	Background

* Average intensity of 600 negative control probes

** Standard deviation of intensities of 600 negative control probes

Control Diagrams

The diagrams in this section illustrate control structure and function.

Sample-Independent Controls

Sample-independent controls evaluate the performance of specific steps in the process flow.

Staining Controls

Staining controls are used to examine the efficiency of the staining step in both the red and green channels. Staining controls have dinitrophenyl (DNP) or biotin attached to the beads. *Controls Table* on page 234 lists the control names, relevant color channel, and expected intensity of biotin and DNP labeling controls. These controls are independent of the hybridization and extension step. Both red and green channels can be evaluated using the Staining Controls.

Figure 156 Staining Controls (Sample-Independent)

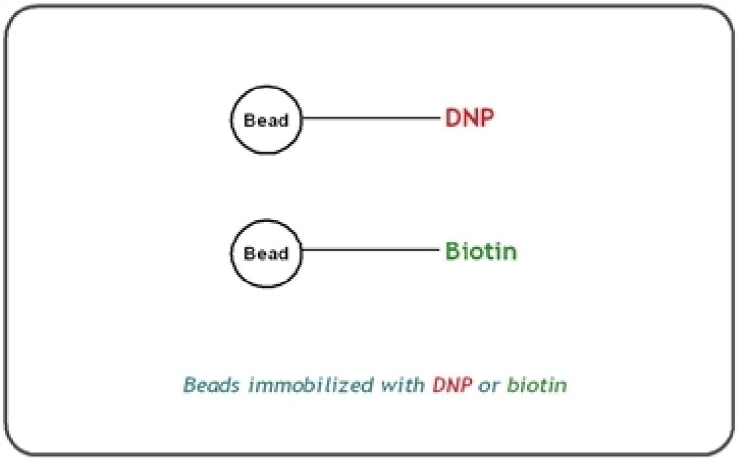

Extension Controls

Extension controls test the extension efficiency of A, T, C, and G nucleotides from a hairpin probe, and are therefore sample-independent. Both red (A,T) and green (C,G) channels are monitored. *Controls Table* on page 234 lists the control names, the relevant color channel, and the expected intensity of the extension controls.

Figure 157 Extension Controls (Sample-Independent)

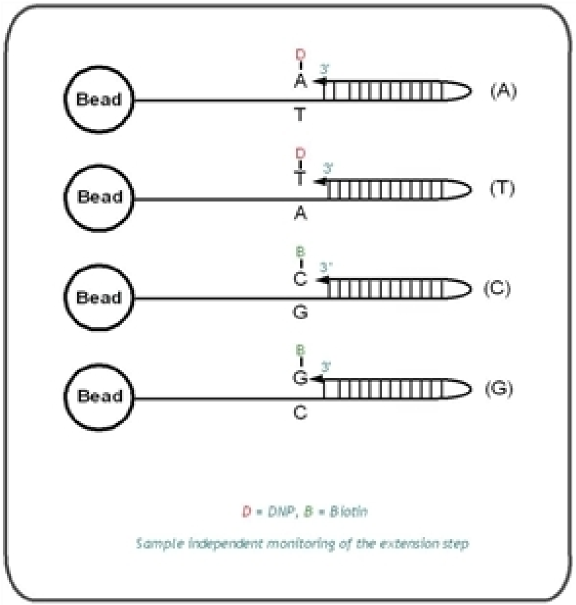

Hybridization Controls

The hybridization controls test the overall performance of the entire assay using synthetic targets instead of amplified DNA. These synthetic targets complement the sequence on the array perfectly, allowing the probe to extend on the synthetic target as template.

The synthetic targets are present in the hybridization buffer (RA1) at three levels, monitoring the response from high-concentration (5 pM), medium-concentration (1 pM), and low-concentration (0.2 pM) targets. All bead type IDs should result in signal with

various intensities, corresponding to the concentrations of the initial synthetic targets. Performance of hybridization controls should only be monitored in the green channel. *Controls Table* on page 234 lists control names for the hybridization controls and expected intensities.

Figure 158 Hybridization Controls (Sample-Independent)

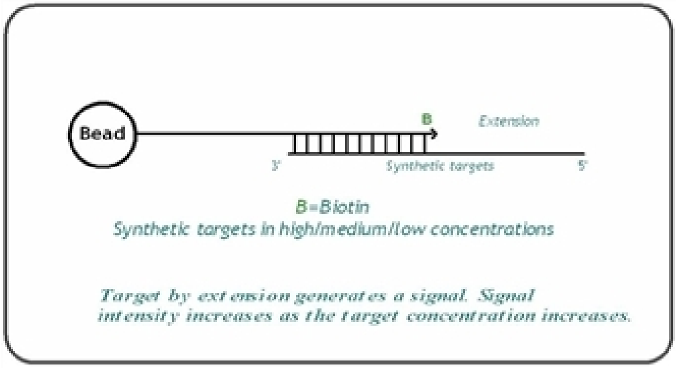

Target Removal Controls

Target removal controls test the efficiency of the stripping step after the extension reaction. In contrast to allele-specific extension, the control oligos are extended using the probe sequence as template. This process generates labeled targets. The probe sequences are designed such that extension from the probe does not occur.

All target removal controls should result in low signal compared to the hybridization controls, indicating that the targets were removed efficiently after extension. The target removal controls are present in the hybridization buffer RA1. Performance of target removal controls should only be monitored in the green channel. *Controls Table* on page 234 lists control names and expected signal.

Figure 159 Target Removal Controls (Sample-Independent)

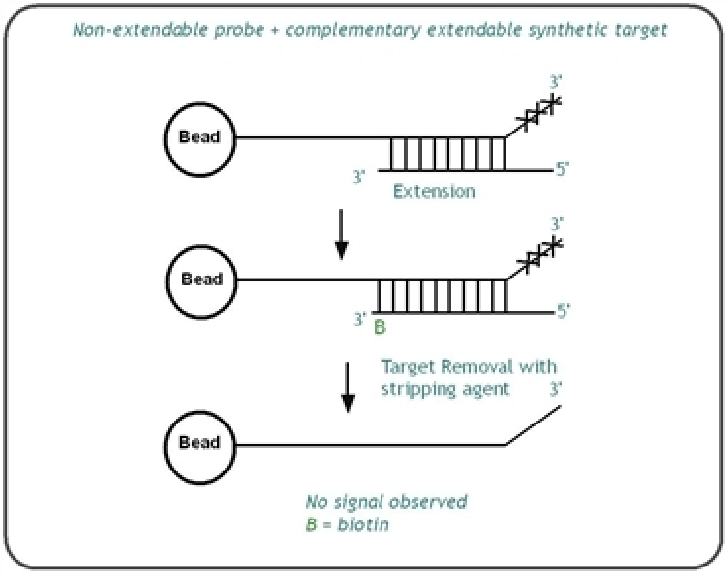

Sample-Dependent Controls

The sample-dependent controls can be used to evaluate performance across samples. These control oligos are designed for bisulfite-converted human genomic DNA sequences. Because target sequences do not contain CpG dinucleotides, the performance of the control oligos does not depend on the methylation status of the template DNA.

Bisulfite-Conversion Controls

These controls assess the efficiency of bisulfite conversion of the genomic DNA. The Infinium Methylation probes query a [C/T] polymorphism created by bisulfite conversion of non-CpG cytosines in the genome.

Bisulfite-Conversion I

These controls use the Infinium I probe design and allele-specific single base extension to monitor efficiency of bisulfite conversion. If the bisulfite conversion reaction was successful, the "C" (Converted) probes will match the converted sequence and be

extended. If the sample has unconverted DNA, the "U" (Unconverted) probes will be extended. There are no underlying C bases in the primer landing sites, except for the query site itself. Performance of bisulfite conversion controls C1, C2, and C3 should be monitored in the Green channel, and controls C4, C5, and C6 should be monitored in Red channel.

Figure 160 Bisulfite-conversion I Controls (Sample-Dependent)

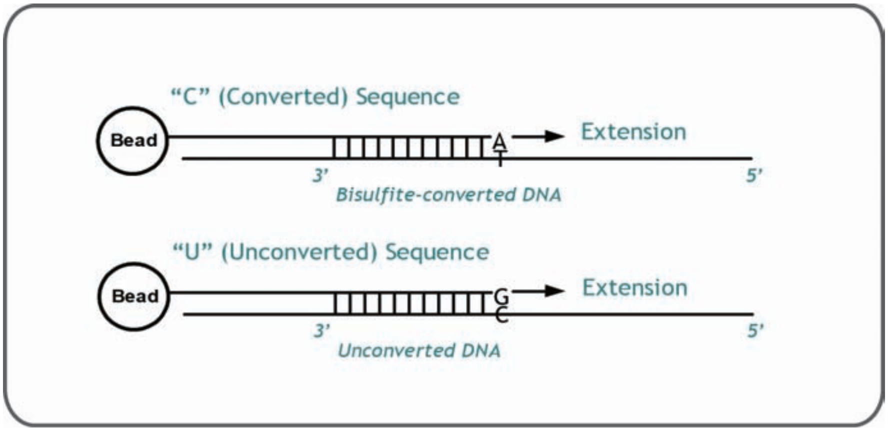

Bisulfite-Conversion II

These controls use Infinium II probe design and single base extension to monitor the efficiency of bisulfite conversion. If the bisulfite conversion reaction was successful, the "A" base will be incorporated and the probe will have intensity in the Red channel. If the sample has unconverted DNA, the "G" base will be incorporated across the unconverted cytosine, and the probe will have elevated signal in the Green channel.

Figure 161 Bisulfite-conversion II Controls (Sample-Dependent)

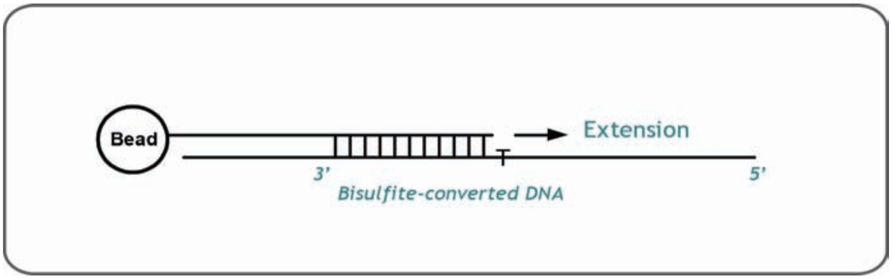

Specificity Controls

Specificity controls are designed to monitor potential non-specific primer extension for Infinium I and Infinium II assay probes. Specificity controls are designed against non-polymorphic T sites.

Specificity I

These controls are designed to monitor allele-specific extension for Infinium I probes. The methylation status of a particular cytosine is carried out following bisulfite treatment of DNA through the use of query probes for unmethylated and methylated state of each CpG locus. In assay oligo design, the A/T match corresponds to the unmethylated status of the interrogated C, and the G/C match corresponds to the methylated status of C. G/T mismatch controls check for non-specific detection of methylation signal over unmethylated background. PM controls correspond to A/T perfect match and should give high signal. MM controls correspond to G/T mismatch and should give low signal. Performance of GT Mismatch controls should be monitored in both green and red channels. the Controls dashboard table lists expected outcome for controls probes.

Figure 162 Specificity I Controls (Sample-Dependent)

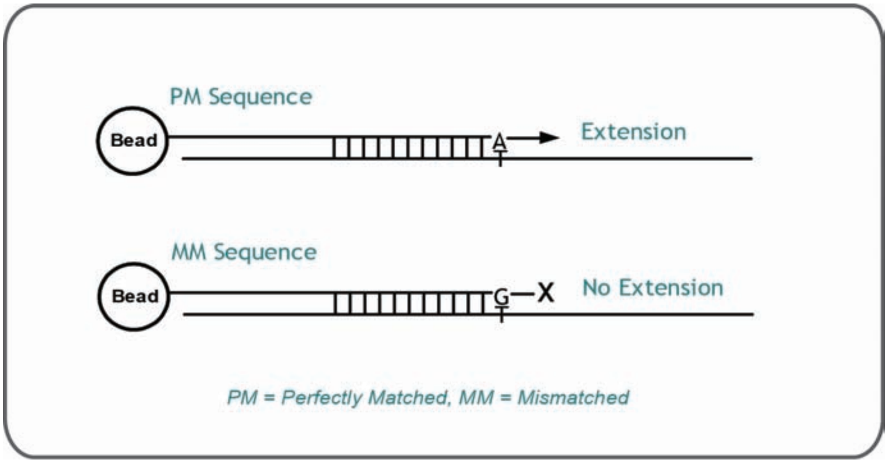

Specificity II

These controls are designed to monitor extension specificity for Infinium II probes and check for potential non-specific detection of methylation signal over unmethylated background. Specificity II probes should incorporate the "A" base across the non-polymorphic T and have intensity in the Red channel. In case of non-specific incorporation of the "G" base, the probe will have elevated signal in the Green channel.

Figure 163 Specificity II Controls (Sample-Dependent)

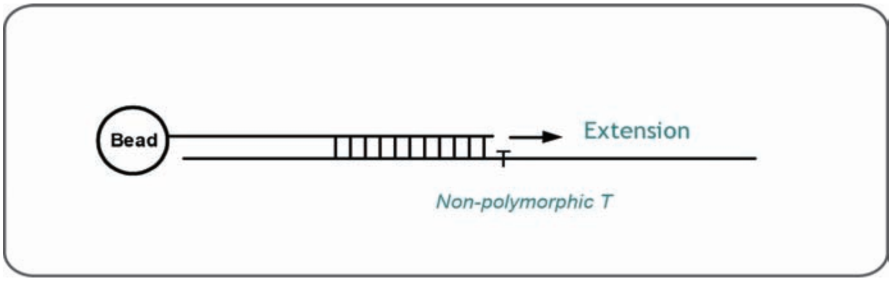

Negative Controls

Negative control probes are randomly permutated sequences that should not hybridize to the DNA template. Negative controls are particularly important for methylation studies because of a decrease in sequence complexity after bisulfite treatment. The mean signal of these probes defines the system background. This is a comprehensive measurement of background, including signal resulting from cross-hybridization, as well as non-specific extension and imaging system background. The GenomeStudio platform uses the Average signal and standard deviation of 600 negative controls to establish detection limits for the methylation probes. Performance of negative controls should be monitored in both green and red channels.

Non-Polymorphic Controls

Non-polymorphic controls test the overall performance of the assay, from amplification to detection, by querying a particular base in a non-polymorphic region of the bisulfite genome. They let you compare assay performance across different samples. One non-polymorphic control has been designed to query each of the four nucleotides (A,T, C, and G). *Controls Table* on page 234 lists the bead type IDs. The target with the C base

results from querying the opposite whole genome amplified strand generated from the converted strand.

Figure 164 Non-Polymorphic Controls (Sample-Dependent)

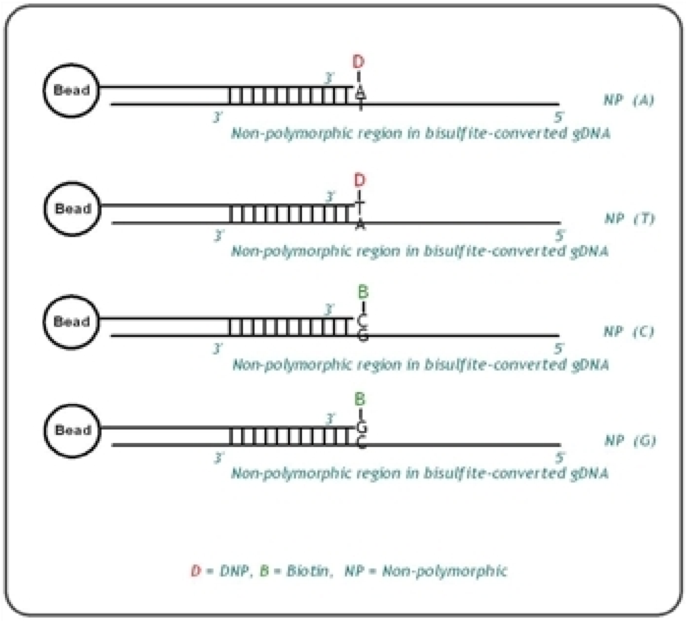

Index

A

arrays 11
AutoLoader2 17

B

BCD plate 35, 131
bead type IDs 234
BeadChips
  description 11
biotin controls 236
bisulfite conversion 35, 131

C

color channels
  evaluate 236
  monitor extension 237
control oligos
  See controls 232
controls 243
  bead type IDs 234
  Control Dashboard 233
  extension 237
  hybridization 237
  non-polymorphic 242
  sample-dependent 232, 239, 243
  sample-independent 232, 236, 238
  staining controls 236
  target removal 238
customer support 247

D

data analysis 112, 229
differential analysis 112, 229
DNA 27
  bisulfite conversion 35, 131
    input amount 38, 134
  track 13
DNP 236
documentation 247

E

equipment
  automated protocol
    Illumina-supplied 118
    user-supplied 117
  manual protocol
    Illumina-supplied 23
    user-supplied 23
experienced user cards 13
extension controls 237

F

Flow-Through Chambers
  single-base extension 96
  stain 98

G

gDNA input amounts 38, 134
gene analysis 112, 229
GenomeScan 17
GenomeStudio
  Controls Dashboard 233
  integrated informatics 112, 229

H

help, technical 247
HiScan 17
hybridization
  controls 237

I

IllumiCode Sequence IDs 234
Infinium Assay
  overview 2
Infinium HD Assay
  controls
    See controls 232
iScan System 17

L

lab tracking form 43

M

materials
  automated protocol
    Illumina-supplied 119-120
    user-supplied 24, 119
  manual protocol
    Illumina-supplied 25
methylation
  detection 35, 131
multiplex 2

N

non-polymorphic controls 242

P

PicoGreen DNA quantitation kit 27
plates
  BCD 35, 131
protocols
  experienced user cards 13

Q

quantitate dNA 27

R

reagents
  manual protocol
    Illumina-supplied 26

S

sample-dependent controls 239, 243
  non-polymorphic 242
sample-independent controls 236, 238
  extension 237
  hybridization 237
  staining controls 236
  target removal 238
Sample QDNA plate 27, 122
sample sheet 13
SNPs
  number per sample 2
staining controls 236
Standard QDNA plate 27, 122
system controls
  See controls 232

T

target removal controls 238
Tecan GenePaint 16
technical assistance 247
track sample DNA 13

U

unmethylated cytosines 35, 131

X

XStain BeadChips
  single-base extension 96

stain 98

Z

Zymo Research
EZ DNA methylation kit 37, 133

Technical Assistance

For technical assistance, contact Illumina Customer Support.

Table 19 Illumina General Contact Information

**Illumina Website**	http://www.illumina.com
**Email**	techsupport@illumina.com

Table 20 Illumina Customer Support Telephone Numbers

Region	Contact Number
North America toll-free	1.800.809.ILMN (1.800.809.4566)
United Kingdom toll-free	0800.917.0041
Germany toll-free	0800.180.8994
Netherlands toll-free	0800.0223859
France toll-free	0800.911850
Other European time zones	+44.1799.534000
Other regions and locations	1.858.202.ILMN (1.858.202.4566)

MSDSs

Material safety data sheets (MSDSs) are available on the Illumina website at http://www.illumina.com/msds.

Product Documentation

If you require additional product documentation, you can obtain PDFs from the Illumina website. Go to http://www.illumina.com/support/documentation.ilmn. When you click on a link, you will be asked to log in to iCom. After you log in, you can view or save the PDF. To register for an iCom account, please visit https://icom.illumina.com/Account/Register.
